# A bioinformatics insight to rhizobial globins: gene identification and mapping, polypeptide sequence and phenetic analysis, and protein modeling.

**DOI:** 10.12688/f1000research.6392.1

**Published:** 2015-05-13

**Authors:** Reinier Gesto-Borroto, Miriam Sánchez-Sánchez, Raúl Arredondo-Peter

**Affiliations:** 1 Laboratorio de Biofísica y Biología Molecular, Centro de Investigación en Dinámica Celular, Instituto de Investigación en Ciencias Básicas y Aplicadas, Universidad Autónoma del Estado de Morelos, Colonia Chamilpa, Morelos, 62210, Mexico

**Keywords:** Burkholderia, Cupriavidus, flavohemoglobin, globin-coupled sensor, Rhizobium, single-domain globin, truncated (2/2) hemoglobin

## Abstract

Globins (Glbs) are proteins widely distributed in organisms. Three evolutionary families have been identified in Glbs: the M, S and T Glb families. The M Glbs include flavohemoglobins (fHbs) and single-domain Glbs (SDgbs); the S Glbs include globin-coupled sensors (GCSs), protoglobins and sensor single domain globins, and the T Glbs include truncated Glbs (tHbs). Structurally, the M and S Glbs exhibit 3/3-folding whereas the T Glbs exhibit 2/2-folding. Glbs are widespread in bacteria, including several rhizobial genomes. However, only few rhizobial Glbs have been characterized. Hence, we characterized Glbs from 62 rhizobial genomes using bioinformatics methods such as data mining in databases, sequence alignment, phenogram construction and protein modeling. Also, we analyzed soluble extracts from *Bradyrhizobium* *japonicum* USDA38 and USDA58 by (reduced + carbon monoxide (CO) *minus* reduced) differential spectroscopy. Database searching showed that only *fhb*, *sdgb*, *gcs* and *thb* genes exist in the rhizobia analyzed in this work. Promoter analysis revealed that apparently several rhizobial *glb* genes are not regulated by a -10 promoter but might be regulated by -35 and Fnr (fumarate-nitrate reduction regulator)-like promoters. Mapping analysis revealed that rhizobial *fhb*s and *thb*s are flanked by a variety of genes whereas several rhizobial *sdgb*s and *gcs*s are flanked by genes coding for proteins involved in the metabolism of nitrates and nitrites and chemotaxis, respectively. Phenetic analysis showed that rhizobial Glbs segregate into the M, S and T Glb families, while structural analysis showed that predicted rhizobial SDgbs and fHbs and GCSs globin domain and tHbs fold into the 3/3- and 2/2-folding, respectively. Spectra from *B*. *japonicum* USDA38 and USDA58 soluble extracts exhibited peaks and troughs characteristic of bacterial and vertebrate Glbs thus indicating that putative Glbs are synthesized in *B*. *japonicum* USDA38 and USDA58.

## Introduction

Globins (Glbs) are proteins widely distributed in organisms from the three kingdoms of life, *i*. *e*. in Archaea, Eubacteria and Eukarya ^1^. Structurally, Glbs fold into a tertiary structure known as the globin fold. This protein folding consists of six to eight α-helices (designated with letters A to H) that form a hydrophobic pocket where a heme prosthetic group is located ^2^. Two structural types of the globin fold have been identified in Glbs: the 2/2- and 3/3-fold. In the 2/2-Glbs, helices B and E overlap to helices G and H ^3^ and in the 3/3-Glbs helices A, E and F overlap to helices B, G and H ^4, 5^. Likewise, three evolutionary families have been identified in Glbs ^6, 7^: the M, S and T Glb families. The M Glbs include flavohemoglobins (fHbs) and single-domain Glbs (SDgbs), the S Glbs include globin-coupled sensors (GCSs), protoglobins and sensor single domain globins, and the T Glbs include truncated Glbs (tHbs) (which are further classified into class 1, class 2 and class 3 tHbs). Canonical tHbs are ~20 to 40 amino acids shorter than the globin fold, resulting in an almost absent helix A and a helix F that is reduced to a single turn ^8, 9^. The M and S Glbs fold into the 3/3-fold whereas the T Glbs fold into the 2/2-fold.

A variety of gaseous ligands bind to the heme Fe of Glbs, most notably O _2_ and nitric oxide (NO). The reversible binding of O _2_ is associated with the major function of Glbs in organisms: the transport of O _2_. Binding of NO by oxygenated Glbs is essential to NO-detoxification *via* NO-dioxygenase activity ^10, 11^. Several additional functions have been reported for Glbs, including dehaloperoxidase activity and reaction with free radicals, binding and transport of sulfide and lipids, and O _2_-sensing (reviewed by Giardina *et al.* ^12^ and Vinogradov *et al.* ^13^). This indicates that *in vivo*, Glbs might be multifunctional proteins.

Glbs are widespread in bacteria. A comprehensive genomic analysis revealed that *glb* genes belonging to the M, S and T Glb families exist in the genomes of 1185 Eubacteria, including several rhizobial genomes ^7^. However, only few rhizobial *glb* genes have been characterized. Characterizing rhizobial Glbs is of interest because rhizobia establish symbiotic relationships with leguminous plants. A result of this plant-microbe interaction is the symbiotic fixation of atmospheric N _2_, which occurs within specialized plant organs called nodules ^14^. Symbiotic N _2_-fixation is a process modulated by a variety of factors, such as the O _2_^15^ and NO ^16, 17^ levels in the surrounding environment. Glbs bind O _2_ and NO and thus may function in some aspects of the N _2_-fixation, *e*. *g*. by transporting O _2_ and detoxifying NO. Modulation of O _2_ levels in the plant cell cytoplasm from nodules is well characterized ^18, 19^. A plant Glb (leghemoglobin (Lb)) that is synthesized at high (~3 to 5 mM) concentrations in nodules apparently facilitates O _2_-diffusion to the symbiotic rhizobia and maintains low (submicromolar) concentrations of O _2 _within nodules. This is essential for sustaining the (micro) aerobic respiration of symbiotic rhizobia and preventing the inactivation of nitrogenase (which fixes the atmospheric N _2_ into NH _4_^+^) by O _2_. The binding and metabolizing of NO by Lb and other Glbs is also well documented ^11, 20^. Thus, a likely function for Lb in nodules is to detoxify the NO that is generated during the plant infection by rhizobia ^21^. However, little is known about the properties and functions of Glbs either within the symbiotic or free-living rhizobia.

Forty-six years ago Appleby ^22^ was the first to propose the existence of Glbs in rhizobia. This author detected absorption peaks and troughs that are characteristic of Glbs in differential (dithionite reduced + CO *minus* dithionite reduced) spectra of soluble extracts from *Bradyrhizobium japonicum* 505 (Wisconsin). Subsequent spectroscopic analyses suggested the existence of soluble Glbs in *Rhizobium leguminosarum* bv. viciae ^23^, *B. japonicum* NPK63 ^24^ and *R. etli* CE3 ^25^. The first rhizobial *glb* gene was identified in the pSymA megaplasmid of *Sinorhizobium meliloti* 1021 ^26^. BLAST analysis revealed that this gene corresponded to an *fhb* gene and thus was named *smfhb*. A bioinformatics analysis showed that *smfhb* is flanked by *nos* and *fix* genes (which code for denitrification enzymes and high O _2_-affinity terminal oxidases and an O _2_-sensor, respectively) and that apparently it is regulated by an Fnr-like promoter. These observations suggested that *smfhb* is regulated by the concentration of O _2_ and that SmfHb functions in some aspects of nitrogen metabolism. A transcriptomic analysis of the *S. meliloti* response to NO in culture showed that *smfhb* (also designated as a *S. meliloti hmp*) is upregulated by NO and the analysis of a *smfhb ^-^* mutant exhibited a high sensitivity to NO in culture and led to a reduced N _2_-fixation efficiency *in planta*. These observations suggested that SmfHb functions in some aspects of NO metabolism, possibly by detoxifying NO ^27^.

Genomic analysis reported by Vinogradov *et al*. ^7^ revealed that Glb sequences exist in several rhizobia. However, in spite of the above reports knowledge on the rhizobial Glbs is quite limited. Hence, in order to obtain information on the properties of rhizobial Glbs we characterized Glb sequences from selected rhizobial genomes by using bioinformatics methods. These included gene characterization, polypeptide sequence and phenetic analysis, as well as protein modeling. Also, we analyzed soluble extracts from *B. japonicum* USDA38 and USDA58 by differential spectroscopy. Our main results showed that only *fhb*, *sdgb*, *gcs* and *thb* genes exist in the rhizobia analyzed in this work; that several rhizobial *glb* genes are not regulated by a -10 promoter but might be regulated by -35 and Fnr-like promoters; that rhizobial *fhb*s and *thb*s are flanked by a variety of genes whereas several rhizobial *sdgb*s and *gcs*s are flanked by genes coding for proteins involved in the metabolism of nitrates and nitrites and chemotaxis, respectively; that rhizobial Glbs segregate into the M, S and T Glb families; that predicted rhizobial SDgbs and fHbs and GCSs globin domain and tHbs fold into the 3/3- and 2/2-fold, respectively, and that spectra from *B. japonicum* USDA38 and USDA58 soluble extracts exhibit peaks and troughs characteristic of bacterial and vertebrate Glbs.

## Methods

### Database search

Putative Glb sequences and Glb domains were identified in databases ( Table S1) containing the genomes of rhizobial species and strains using the query sequences *S. meliloti* fHb; *Vitreoscilla* SDgb; *Agrobacterium tumefaciens* GCS; *Methanosarcina acetivorans* protoglobin; *Methylacidiphilum infernorum* sensor single domain globin; *Mycobacterium tuberculosis* tHb class 1; *A. tumefaciens* tHb class 2, and *M. avium* tHb class 3 (Genbank accession numbers AY328026, AAA75506, NP_354049, 2VEB_A, YP_001939425, NP_216058, WP_020813663 and BAN32501, respectively) and the SUPERFAMILY database ( http://supfam.mrc-lmb.cam.ac.uk) ^28^. Resulting sequences were subjected to a FUGUE analysis ( http://tardis.nibio.go.jp/fugue/prfsearch.html) ^29^ to determine the most similar Glb structure and presence of proximal H at the myoglobin-fold position F8. Putative Glbs had to satisfy the following criteria: length higher than or ~100 amino acids, a FUGUE Z score higher than 6 (which corresponds to 99% specificity ^29^) with known Glb structures, and the presence of proximal H at position F8.

### Gene mapping and detection of promoter sequences

Scaffolds containing copies of the *glb* gene were used for mapping *glb*s. This included the detection of open reading frames (ORFs) ~5 kb up- and downstream to *glb*s and ORF length, transcription direction and localization in the +/- strand. Canonical (-10 and -35) and Fnr ^30^ promoter sequences and Shine-Dalgarno sequences were searched within 130 nucleotides upstream to the rhizobial *glb* genes either by using the search tool of MS Word ^®^ or by pairwise sequence alignments using the ClustalX program ( http://www.clustal.org/clustal2/) ^31^.

### Protein sequence alignments and phenetic analysis

Pairwise and multiple sequence alignments were performed using the ClustalX program ^31^. Multiple sequence alignment was manually verified using the procedure described by Kapp *et al*. ^32^ based on the myoglobin-fold ^33^. A phenogram was constructed from the aligned sequences using the UPGMA method from the ClustalX program. The resulting phenogram was edited using the iTOL program ( http://itol.embl.de/) ^34^.

### Modeling and analysis of the predicted proteins tertiary structure

The tertiary structure of rhizobial Glbs was modeled using the automated mode of the I-TASSER server ( http://zhanglab.ccmb.med.umich.edu/I-TASSER/) ^35– 37^, which also provided the best structural homologs to the query sequences. Models were edited using the VMD program ( http://www.ks.uiuc.edu/Research/vmd/) ^38^ and Adobe Photoshop ^®^ software. Distance and dihedral angles of amino acids at the heme prosthetic group were calculated using the distance and dihedral tools of the SwissPDBViewer program ( http://spdbv.vital-it.ch/) as described by Gopalasubramaniam *et al*. ^39^ and Sáenz-Rivera *et al*. ^40^, respectively.

### Bacterial growth, cell rupture and spectral analysis

*Bradyrhizobium japonicum* USDA38 and USDA58 were kindly provided by Drs. Donald Keister and Douglas Jones (United States Department of Agriculture, USA). All reagents were purchased from Sigma-Aldrich (St. Louis MO, USA). *B. japonicum* cells were grown in YM (Yeast Mannitol) broth (per 100 ml: KH _2_PO _4_, 50 mg; MgSO _4_, 20 mg; NaCl, 10 mg; mannitol, 1 g; yeast extract, 50 mg, pH 7.0) for 3 to 5 days at 30°C with shaking at 200 rpm. Cells were harvested by centrifugation at 11,000 *× g*, pellets were resuspended in 50 mM Na-phosphate buffer (pH 7.2) containing 1 mM EDTA and 1 mM phenylmethylsulfonyl fluoride (PMSF). Cells were disrupted by sonication at maximum power (three cycles of 1 min each in ice) and incubation at 4°C overnight with gentle agitation after the addition of DNAse I (40 U/ml), RNAse A (3 U/ml) and lysozyme (2 mg/ml). The resulting solution was cleared by centrifugation at 22,000 *× g* for 40 min at 4°C, and the supernatant was fractionated with solid ammonium sulphate between 35 and 65% saturation. The resulting pellet was resuspended in 5 ml of 50 mM Na-phosphate buffer (pH 7.2) containing 1 mM EDTA and 1 mM PMSF and dialyzed for 18 h against the same buffer to remove the excess of salts. 0.5 to 1 ml aliquots of the dialyzed solution were used to obtain the dithionite reduced + CO *minus* dithionite reduced differential spectra in a Beckman DU6 spectrophotometer. Control spectra were obtained from commercial (Sigma-Aldrich) preparations of the sperm whale myoglobin and bovine blood hemoglobin.

Globin genes detected in the genomes of rhizobial bacteria Globin nomenclature corresponds to the first three binomial (genus and species) letters followed by the strain name, globin type and gene copy number. URLs indicate links to individual *glb* gene sequences ^56^. Click here for additional data file. Copyright: © 2015 Gesto-Borroto R et al. 2015 Data associated with the article are available under the terms of the Creative Commons Zero "No rights reserved" data waiver (CC0 1.0 Public domain dedication).

Predicted Glb polypeptides detected in the genomes of rhizobial bacteria Globin nomenclature corresponds to the first three binomial (genus and species) letters followed by the strain name, globin type and globin copy number. URLs indicate links to individual Glb polypeptide sequences ^57^. Click here for additional data file. Copyright: © 2015 Gesto-Borroto R et al. 2015 Data associated with the article are available under the terms of the Creative Commons Zero "No rights reserved" data waiver (CC0 1.0 Public domain dedication).

Distance to the heme Fe and orientation of distal, proximal, B10 and CD1 amino acids in the predicted structure of selected rhizobial Glbs (Table S2) Structural homologs (including the PDB ID number), amino acids from the structural homologs and values for the structural homologs amino acids to individual rhizobial Glbs are indicated in parenthesis for comparison ^58^. Click here for additional data file. Copyright: © 2015 Gesto-Borroto R et al. 2015 Data associated with the article are available under the terms of the Creative Commons Zero "No rights reserved" data waiver (CC0 1.0 Public domain dedication).

## Results and discussion

### Detection of Glb sequences in the genomes of α- and β-rhizobia

Recently, Vinogradov *et al*. ^7^ reported that Glb sequences exist in the genomes of 96 rhizobia. However, this report did not provide the rhizobial Glb sequences or links to rhizobial scaffolds containing the Glb sequences. Hence, we searched in databases (see the Methods section and Table S1) in order to obtain rhizobial Glb sequences for analysis. We selected 62 out of the 96 rhizobial genomes reported by the above authors representing the major rhizobial genera, species and strains, which included α- and β-rhizobia ( *i*. *e*. those classified within the α- and β-proteobacteria, respectively). A total of 197 *glb* sequences were detected in the 62 rhizobial genomes, corresponding to 7 *fhb*s, 47 *sdgb*s, 40 *gcs*s and 103 *thb*s (4 *thb*s class 1, 56 *thb*s class 2 and 43 *thb*s class 3). Individual Glb nucleotide and polypeptide sequences and links to rhizobial scaffolds containing the Glb sequences are provided in Dataset 1 and Dataset 2, respectively. All the rhizobial genomes analyzed in this work contained *glb* sequences, thus indicating that *glb*s are widespread in rhizobia. However, protoglobin and sensor single domain globin sequences were not detected in the rhizobial genomes. This observation indicates that apparently only the *fhb*, *sdgb*, *gcs* and *thb* lineages evolved within rhizobia.

**Figure 1.**
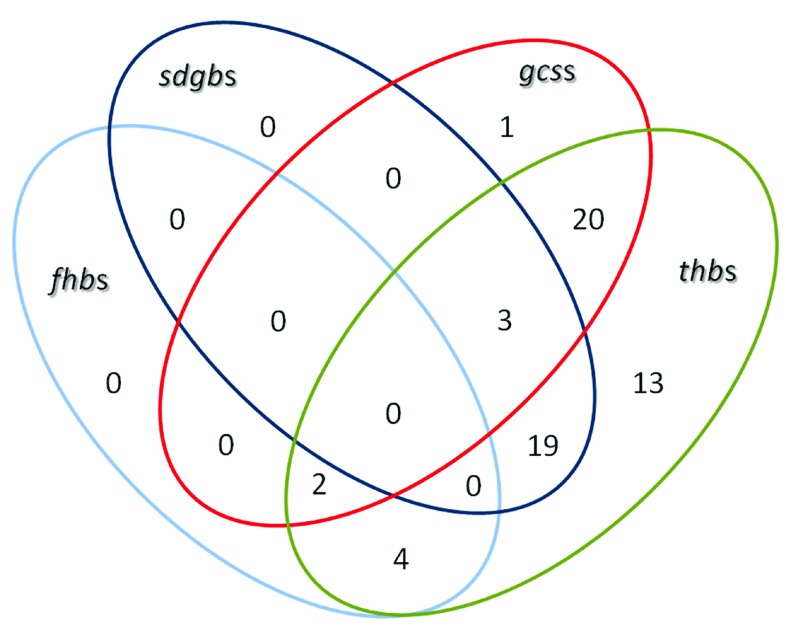
Venn diagram illustrating the distribution of *glb* genes in the rhizobial bacteria analyzed in this work. Numbers correspond to rhizobial genomes containing *glb*s.

A distribution analysis showed that most (61) of the rhizobial genomes analyzed in this work contain *thb*s, either as single *thb*s (13) or in combination with *fhb*s, *sdgb*s and/or *gcs*s (48). Furthermore, one rhizobial genome contained only a *gcs* and none contained only *fhb*s and *sdgb*s and the combinations *fhb*s + *sdgb*s, *fhb*s + *gcs*s and *sdgb*s + *gcs*s ( Figure 1). These observations indicate that in the rhizobia analyzed in this work *thb*s predominate over other *glb*s and that in these bacteria *fhb*s, *sdgb*s and *gcs*s mostly exist in combination with *thb*s. Also, analysis of the *glb* copy number showed that in the rhizobia analyzed in this work *fhb*s mostly exist as single copy (ranging from one to two copies), *sdgb*s mostly exist as two copies (ranging from one to four copies), *gcs*s exist as either single or two copies (ranging from one to two copies) and *thb*s mostly exist as two copies (ranging from one to three copies) although quite a few *thb*s exist as single copy ( Table 1). Thus, apparently rhizobial *glb*s mostly exist as either single or two copies.

**Table 1.** Number of *glb* copies detected in the rhizobial genomes analyzed in this work.

*glb*/no. of copies	No. of genomes
*fhb*s	
1	5
2	1
*sdgb*s	
1	5
2	11
3	4
4	2
*gcs*s	
1	12
2	14
*thb*s	
1	22
2	36
3	3

### Mapping of *glb* genes in the rhizobial genomes

The *glb* genes detected in this work were mapped within the rhizobial genomes in order to identify genes that flank nearby to and could coexpress with *glb*s. Mapping analysis showed that rhizobial *glb* copies are located in different scaffolds and that they are not tandemly arrayed. Figure S1A shows that either no ORFs or ORFs coding for hypothetical or non-identified proteins are located nearby most of the rhizobial *fhb* genes. However, genes coding for the transcriptional regulator NsrR, 2-nitropropane dioxygenase and NosR, Z, D, F, Y and X are located nearby *cupnecN1fhb1*, *rhilegUPM1137fhb* and *sinmel1021fhb*, respectively. Figure S1B shows that *B. elkanii* and *B. japonicum sdgb*s are mostly flanked by genes coding for proteins that function in nitrate/nitrite metabolism and sugar transport. Figure S1C shows that genes coding for proteins that function in chemotaxis are located nearby several rhizobial *gcs*s, although genes coding for a peptide deformylase, sugar and nitrate transport proteins and NAD(P)H nitrate reductase are located nearby some other rhizobial *gcs*s. Figure S1D shows that genes flanking the rhizobial *thb*s are rather variable. However, *B. japonicum thb*s are often flanked by genes coding for the transcriptional regulator Rieske Fe-S, shikimate kinase and alcohol dehydrogenase; mesorhizobia *thb*s are often flanked by genes coding for permeases and tRNA-Trp, and *R. leguminosarum thb*s are often flanked by genes coding for membrane proteins. Thus, if *glb* and flanking genes coexpress in rhizobia, and proteins coded by these genes function within the same metabolic pathways, the above observations suggest that rhizobial Glbs could play a variety of roles in rhizobial physiology, including nitrate/nitrite metabolism, transport processes, gene regulation and chemotaxis. Interestingly, with the exception of *sinmel1021fhb* which is flanked by *nos* and *fix* genes ( Figure S1A) ^26^, *nif* and *fix* genes coding for proteins that function in N _2_-fixation were not detected nearby the rhizobial *glb* genes. This observation suggests that rhizobial Glbs might not directly function in N _2_-fixation.

### Detection of promoter sequences upstream to the rhizobial *glb* genes

**Figure 2.**
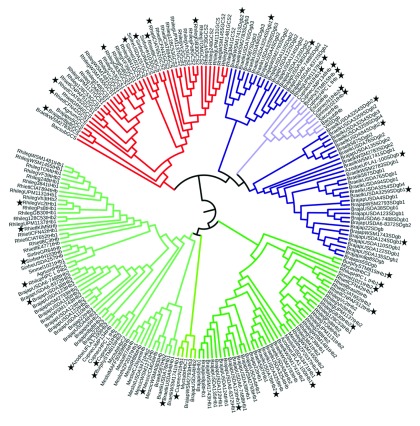
Phenetic relationships among Glbs detected in the genomes of rhizobial bacteria. Phenogram was obtained from the Glbs sequence alignment shown in Figure S2. The fHb, SDgb, GCS, tHb class 1, tHb class 2 and tHb class 3 clusters are indicated with light blue, dark blue, red, light green, bright green and dark green, respectively. Stars indicate Glbs selected for the detection of promoter sequences upstream to the *glb* genes and Glb protein modeling.

Identification of promoter sequences is crucial to an understanding of gene regulation and ultimately protein function within the cell's physiology. Hence, we searched for canonical (-10 and -35) promoters and the O _2_- and NO-regulated Fnr promoter ^30, 41, 42^ within 130 nucleotides upstream to 44 selected rhizobial *glb* genes ( *i*. *e*. those representative of major rhizobial Glb clades identified in this work (see Figure 2)). Also, we searched for Shine-Dalgarno sequences within the same region, which indicate that Glb transcripts could be translated into proteins. Results showed that, with the exception of *burphySTM815thb1*, *burphySTM815thb2* and *rhilupHPC(L)thb1*, a -10 promoter is absent upstream of the selected rhizobial *glb*s. In contrast, with the exception of *cupnecN1thb1* and *rhilupHPC(L)thb2*, a -35 promoter exists upstream of the selected rhizobial *glb*s. Searching for Fnr promoter sequences revealed that Fnr-like promoters exist upstream to 30 out of the 44 selected rhizobial *glb*s, including *fhb*, *sdgb*, *gcs* and *thb* genes. A Shine-Dalgarno sequence was detected upstream to most of the selected rhizobial *glb*s ( Table 2). These observations suggest that the -35 promoter is a major canonical promoter that regulates most of the rhizobial *glb*s, that it is likely that several rhizobial *glb*s are regulated by levels of O _2_ and NO throughout an FNR mechanism ^41– 44^ and that rhizobial Glb transcripts are translated into proteins.

**Table 2.** Position of canonical and Fnr-like promoter sequences and Shine-Dalgarno sequence within 130 nucleotides upstream to selected rhizobial *glb* genes. Consensus sequences are indicated in parenthesis. Identical and non-identical nucleotides into the Fnr-like promoter sequences to the consensus Fnr promoter sequence are indicated with upper- and lowercase letters, respectively. N.D., non-detected.

*glb* gene	Canonical promoters		Fnr promoter		Shine-Dalgarno sequence (AGGAGG)
	-10 promoter	-35 promoter	Sequence	Position	
	(TATAAT)	(TTGACA)	(TTTAAGAGGCCAAT)		
***fhb*s**					
*burphySTM815fhb*	N.D.	-36 to -41	TcTAAGcGaCtgAT	-102 to -115	-10 to -13
*cupnecHPC(L)fhb*	N.D.	-43 to -48	N.D.		-8 to -12
*cupnecJMP134fhb*	N.D.	-46 to -54	TTTAAaAcGgagcc	-5 to -18	-10 to -15
*cupnecN1fhb1*	N.D.	-46 to -52	N.D.		-10 to -15
*cupnecN1fhb2*	N.D.	-29 to -36	aTcAAGgcGgCgAg	-64 to -77	-8 to -12
*rhilegUPM1137fhb*	N.D.	-32 to -37	N.D.		-9 to -12
*sinmel1021fhb*	N.D.	-48 to -56	gTcAAGgaGCCAAa	-12 to -25	-8 to -12
			ggTtgGgGtCCAcT	-61 to -74	
***sdgb*s**					
*azodoeUFLA1-100sdgb*	N.D.	-38 to -42	gccAgGAGtCCgAT	-2 to -15	-8 to -12
*braelkUSDA94sdgb2*	N.D.	-36 to -43	TaTAAGgacatcAT	-114 to -127	-7 to -11
*braelkWSM1741sdgb2*	N.D.	-34 to -40	N.D.		-7 to -12
*braelkUSDA3254sdgb1*	N.D.	-61 to -66	TTTttGgGGCaAAT	-71 to -84	N.D.
*braelkUSDA3254sdgb2*	N.D.	-40 to -44	TTTAcGAGGCtgcT	-11 to -24	-16 to -22
*braelkUSDA3259sdgb1*	N.D.	-41 to -46	TTTcAGAactCAtT	-22 to -35	-8 to -12
			cTTcgGttaCCAAT	-56 to -69	
*brajapUSDA38sdgb2*	N.D.	-53 to -58	N.D.		-6 to -9
*brajapUSDA124sdgb1*	N.D.	-60 to -65	N.D.		-7 to -11
***gcs*s**					
*brajapin8p8gcs*	N.D.	-55 to -60	gTTtcGcctCCgAT	-21 to -34	-6 to -11
*rhietlCIAT652gcs1*	N.D.	-44 to -50	N.D.		-7 to -9
*rhietlCIAT652gcs2*	N.D.	-31 to -36	gTggAGAGGaCcgT	-91 to -104	-2 to -5
*rhietl8c3gcs*	N.D.	-44 to -51	TTTAAccaGgCAtc	-80 to -93	-5 to -11
*rhietlCFN42gcs1*	N.D.	-65 to -70	N.D. **		N.D.
*rhilegGB30gcs1*	N.D.	-51 to -55	TgatcGAGGCaAgg	-33 to -46	-8 to -10
*rhilegGB30gcs2*	N.D.	-47 to -52	gTggtGAGGaCcgT	-90 to -103	-3 to -8
*sinfreGR64gcs*	N.D.	-23 to -29	TTcAgcgGGCCAca	-47 to -60	-6 to -8
*sinmel1021gcs*	N.D.	-31 to -36	N.D.		-6 to -8
***thb*s**					
*azodoeUFLA1-100thb1*	N.D.	-54 to -59	TgctgGAcGCCAAc	-95 to -108	-5 to -7
*azodoeUFLA1-100thb2*	N.D.	-61 to -67	N.D.		-7 to -12
*braelkUSDA76thb2*	N.D.	-56 to -61	TTTgAGAtaCCtAT	-15 to -28	-3 to -5
*braelkUSDA94thb1*	N.D.	-34 to -39	gTTgAGAGcCgcca	-59 to -72	-2 to -4
*brajapUSDA38thb2*	N.D.	-38 to -42	TaTAtcAGGgCAca	-23 to -36	-7 to -9
*brajapUSDA123thb1*	N.D.	-32 to -37	N.D.		-11 to -14
*burphySTM815thb1*	-8 to -13	-31 to 35	TaTAAacGGtaAcT	-90 to -103	-2 to -4
*burphySTM815thb2*	-23 to -28	-43 to -47	cTgAtGcGGCCAgc	-70 to -83 **	N.D.
*cupnecN1thb1*	N.D.	N.D.	TcgctaAGGCCgcT	47 to -60	-7 to -11
*cupnecN1thb2*	N.D.	-43 to -47	N.D.		-7 to -9
*mescicWSM1271thb*	N.D.	-61 to -66	TTgtAGtGGgCgAc	-95 to -108	-8 to -12
*meslotNZP2037thb2*	N.D.	-39 to -43	TgcAAGccGCCAtc	-47 to -60	-9 to -12
*rhietlCNPAF512thb*	N.D.	-32 to -37	TaTAtGAGGagcgg	-28 to -41	N.D.
*rhietlKIM5thb*	N.D.	-59 to -64	N.D.		-8 to -10
*rhilegGB30thb2*	N.D.	-54 to -58	TTggAatGGaCAAT	-58 to -71	-6 to -10
*rhilegVc2thb1*	N.D.	-31 to -35	TTcgAcAtGCaAAT	-90 to -103	N.D.
*rhilupHPC(L)thb1*	-17 to -22	-40 to -45	N.D.		-4 to -7
*rhilupHPC(L)thb2*	N.D.	N.D.	N.D.		-7 to -11
*sinfreHH103thb*	N.D.	-42 to -49	TTTgtcAaGCCctg	-102 to -115	-3 to -6 and -8 to -11
*sinmel1021thb2*	N.D.	-57 to -63	cTTgtcgGGCagAT	-87 to -100	-5 to -7

### Sequence alignments and phenetic analysis of rhizobial Glbs

Pairwise sequence alignments showed that the rhizobial fHbs, SDgbs, GCSs and tHbs analyzed in this work are 34.6 to 85.4%, 6.7 to 100%, 10.9 to 100% and 3.5 to 100% identical, respectively. This indicates that variability among the rhizobial Glb sequences is high. Moreover, identity values for the fHbs globin and flavin domains were 39.1 to 93.7% and 26.5 to 81.1%, respectively, and identity values for the GCSs globin and transmitter domains were 17.5 to 100% and 5.9 to 100%, respectively. Thus, apparently in the rhizobial fHbs and GCSs analyzed in this work the globin domain is more conserved than the flavin and transmitter domains.

The average length and molecular mass for the rhizobial fHbs, SDgbs, GCSs and tHbs analyzed in this work are 400 amino acids and 44 kDa, 141 amino acids and 15 kDa, 510 amino acids and 55 kDa and 149 amino acids and 17 kDa, respectively. However, sequence analysis revealed that globin domain from BraelkUSDA76tHb1, BraelkUSDA94tHb1 and Braelk587tHb2 contains 119 to 237 extra amino acids at the N-terminal and 131 extra amino acids at the C-terminal, and that the globin domain from BrajapUSDA123tHb1, BrajapUSDA135tHb1, BraelkWSM1741SDgb2, RhietlCFN42GCS1, BrajapUSDA4tHb2 and BrajapWSM2793tHb3 contains 27 to 73 extra amino acids at the N-terminal. In contrast, a large deletion comprising helices A and B, CD loop and part of helix E was detected in the BraelkUSDA94SDgb2 sequence indicating that BraelkUSDA94SDgb2 is 89 amino acids in length ( Figure S2).

Multiple sequence alignment showed that, with the exception of 21 GCSs, in the rhizobial Glbs analyzed in this work, the proximal (F8, located at position 322/323 in Figure S2) amino acid to the heme Fe is H. Apparently, in the above rhizobial GCSs, F8 is E. Amino acids other than H occupying the F8 position in bacterial Glbs were previously reported by Vinogradov *et al*. ^7^. However, because H F8 is absolutely conserved in Glbs ( *i*. *e*. from bacteria to mammals) ^1, 32, 45– 47^, assigning E F8 to rhizobial (and other bacterial) GCSs should be taken with caution as this assignment might result from a sequence alignment artifact. Ideally, F8 from rhizobial GCSs should be identified by experimental methods, such as x-ray crystallography. Multiple sequence alignment also showed that in the rhizobial Glbs analyzed in this work, the distal (E7, located at position 285/289/290 in Figure S2) amino acid to the heme Fe is Q in fHbs, can be Q/R/K/M/L in SDgbs, Q in GCSs and can be H/F/L/V/R in tHbs. This indicates that distal Q is conserved in rhizobial fHbs and GCSs and that amino acids occupying the distal position in rhizobial SDgbs and tHbs are variable. The B10 and CD1 amino acids (located at positions 257 and 270/271/273 in Figure S2, respectively), which also participate in binding of ligands to the heme Fe ^48– 50^, are Y and F in most of the rhizobial Glbs analyzed in this work followed by (in order of abundance) F, S and V and H, I, S and Y, respectively.

A phenogram was constructed from the above multiple sequence alignment. Figure 2 shows that the rhizobial Glbs analyzed in this work segregate into two main lineages: one containing fHbs, SDgbs and GCSs, and the other containing tHbs (the fHb/SDgb/GCS and tHb lineages, respectively). This is consistent with the main evolutionary lineages identified in bacterial Glbs ^1, 51, 52^ thus indicating that major evolutionary patterns for rhizobial Glbs were identical to those for other bacterial Glbs. Rhizobial fHbs and GCSs cluster with rhizobial SDgbs within the fHb/SDgb/GCS lineage owing to the similarity between the fHb and GCS globin domains and SDgbs. This has been postulated to be the result of an early divergence from a common ancestor to the bacterial fHb and GCS globin domains and SDgbs ^1, 6^. The tHb lineage segregates into rhizobial tHbs class 1, tHbs class 2 and tHbs class 3. Within this lineage the rhizobial tHbs class 3 segregate in ancestral position to the rhizobial tHbs class 1 and tHbs class 2. Also, the bradyrhizobial, azorhizobial, mesorhizobial, rhizobial and burkholderial tHbs class 3 segregate from each other; the segregation within rhizobial, sinorhizobial, mesorhizobial and β-rhizobial tHbs class 2 is rather conserved, and bradyrhizobial tHbs class 2 and class 3 segregate into the *B. elkanii* and *B. japonicum* tHb sublineages. These observations indicate that rhizobial tHbs evolved similarly to other bacterial tHbs ^7, 8, 52^ and that evolution of rhizobial tHb sublineages was rather conserved.

### Modeling and analysis of the predicted rhizobial Glbs tertiary structure

Structure elucidation is essential to a full understand of a protein´s function within the cell´s physiology. The structure of a considerable number of bacterial and non-bacterial Glbs has been elucidated by x-ray crystallography. However, with the exception of a *S. meliloti* fHb whose tertiary structure was predicted using bioinformatics methods ^26^, the structure of rhizobial Glbs is not known. Hence, we used bioinformatics methods to predict and analyze the tertiary structure of 44 selected rhizobial Glbs ( *i*. *e*. those representative of major rhizobial Glb clades identified in this work (see Figure 2 and Table S2)) using the best structural homologs as templates ( Dataset 3).

**Figure 3.**
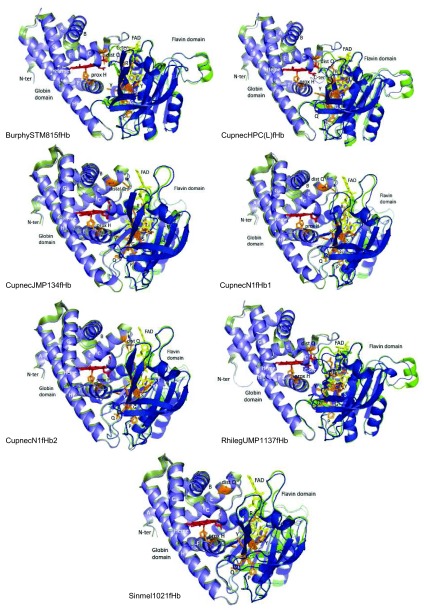
Predicted structure of rhizobial fHbs (blue) overlapped to structural homologues (green). Structural homologues are indicated in Dataset 3. Distal and proximal amino acids to the heme Fe and amino acids that interact with the FAD cofactor are shown in brown. Heme and FAD are shown in red and yellow, respectively. Helices within the globin domain are indicated with letters A to H. All structures are displayed in the same orientation.

Predicted structures for selected rhizobial SDgbs and fHbs and GCSs globin domain and tHbs fold into the 3/3- and 2/2-globin fold, respectively ( Figure 3 to Figure 8). Figure 3 shows that structures among the predicted rhizobial fHbs are highly similar. Yet major differences were detected in the BurphySTM815fHb, CupnecHPC(L)fHb and RhilegUMP1137fHb flavin domains, which exhibited two additional helices. Dataset 3 shows that among globin domains from predicted rhizobial fHbs the distance of the proximal H and distal Q to the heme Fe is 1.44 to 2.47 Å and 6.71 to 15.35 Å, respectively. This observation suggests that the heme Fe in rhizobial fHbs is pentacoordinate.

**Figure 4.**
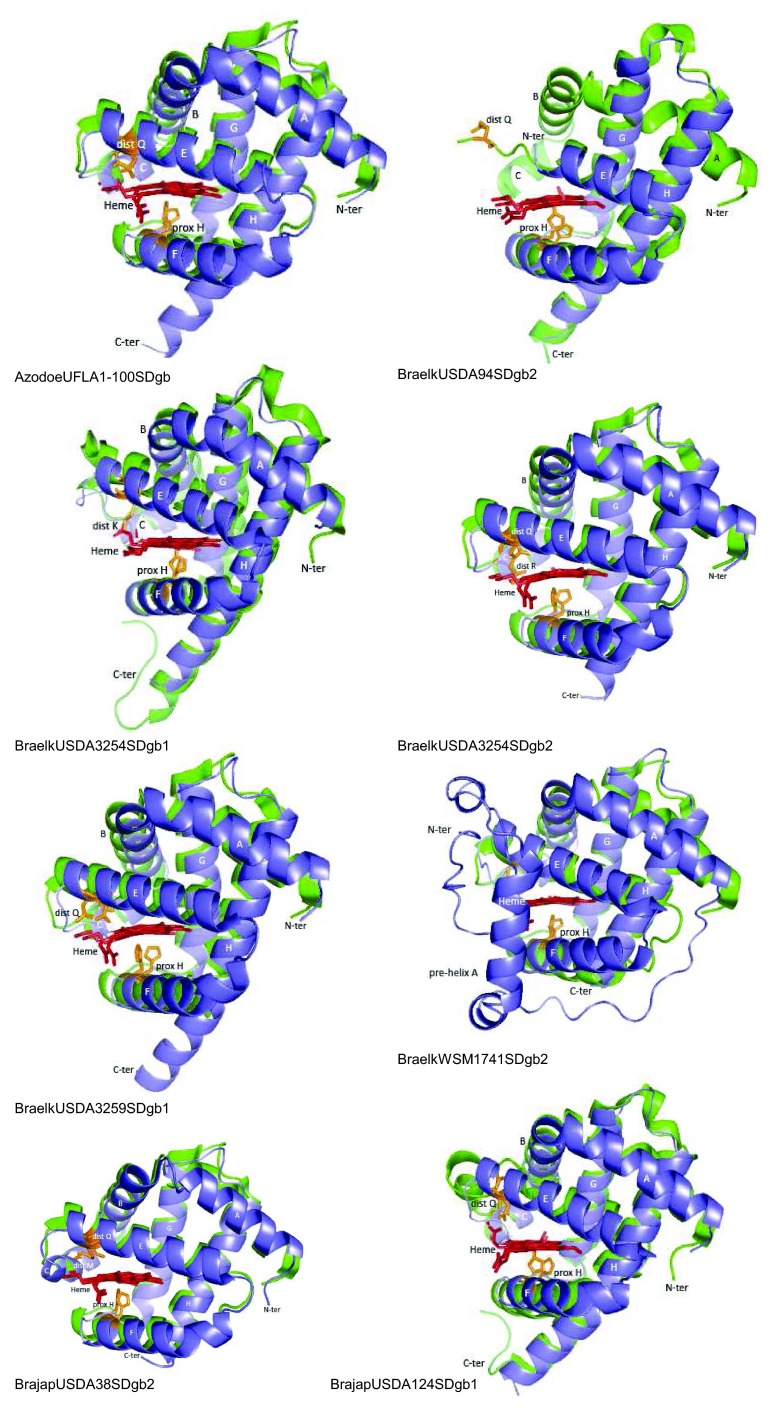
Predicted structure of selected rhizobial SDgbs (blue) overlapped to structural homologues (green). Structural homologues are indicated in Dataset 3. Distal and proximal amino acids to the heme Fe are shown in brown. Heme is shown in red. Helices are indicated with letters A to H. All structures are displayed in the same orientation.

**Figure 5.**
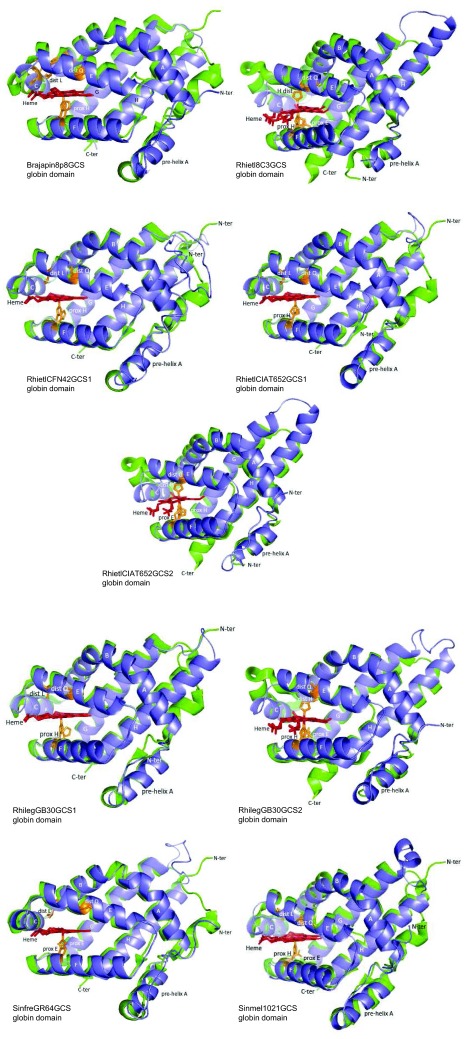
Predicted structure of selected rhizobial GCSs globin domain (blue) overlapped to structural homologues (green). Structural homologues are indicated in Dataset 3. Distal and proximal amino acids to the heme Fe are shown in brown. Heme is shown in red. Helices are indicated with letters A to H. All structures are displayed in the same orientation.

**Figure 6.**
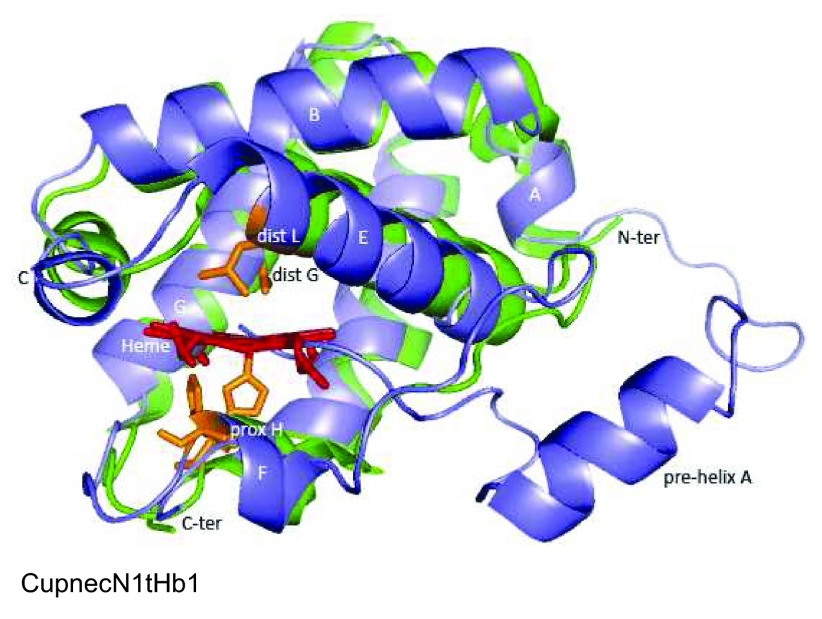
Predicted structure of class 1 CupnecN1tHb1 (blue) overlapped to the structural homologue *Tetrahymena pyriformis* tHb (PDB ID 3AQ5) (green). Distal and proximal amino acids to the heme Fe are shown in brown; only potential distal E11 is shown in the CupnecN1tHb1 structure. Heme is shown in red. Helices are indicated with letters A to H.

**Figure 7.**
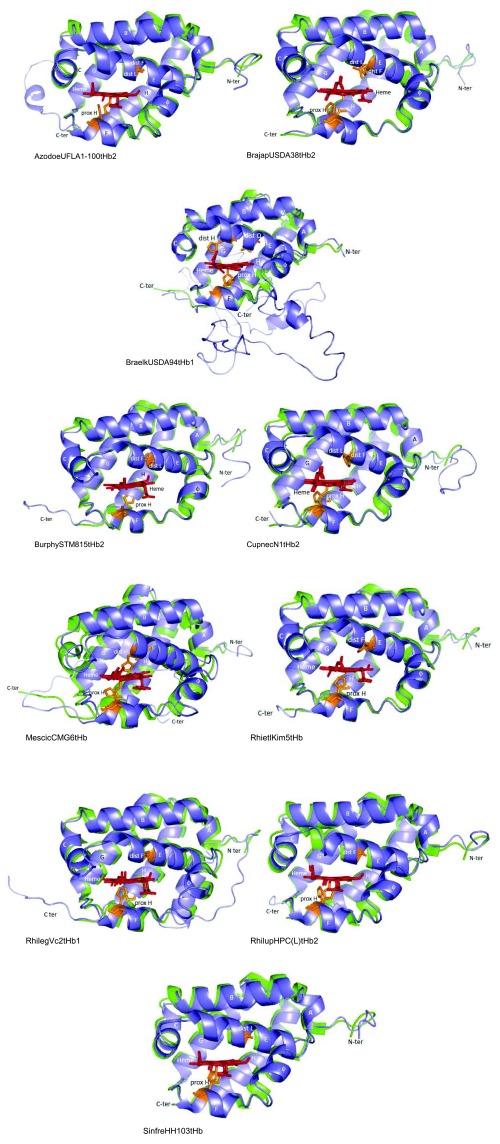
Predicted structure of selected rhizobial tHbs class 2 (blue) overlapped to structural homologues (green). Structural homologues are indicated in Dataset 3. Distal and proximal amino acids to the heme Fe are shown in brown; only potential distal E11 is shown in the tHbs structure. Heme is shown in red. Helices are indicated with letters A to H. Pre-helix F is indicated with the Greek letter φ. All structures are displayed in the same orientation.

**Figure 8.**
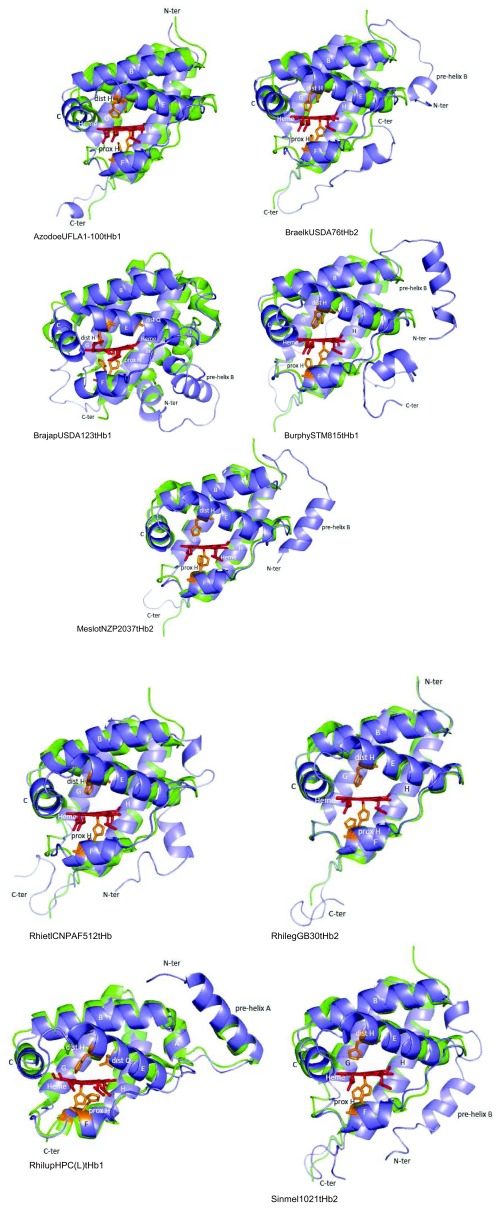
Predicted structure of selected rhizobial tHbs class 3 (blue) overlapped to structural homologues (green). Structural homologues are indicated in Dataset 3. Distal and proximal amino acids to the heme Fe are shown in brown; only potential distal E11 is shown in the tHbs structure. Heme is shown in red. Helices are indicated with letters A to H. All structures are displayed in the same orientation.

Figure 4 shows that 3/3-globin folding is highly conserved in the predicted structure of the rhizobial SDgbs AzodoeUFLA1-100SDgb, BraelkUSDA3254SDgb2, BraelkUSDA3259SDgb1 and BrajapUSDA38SDgb2. Major variations to 3/3-globin folding from predicted rhizobial SDgbs consisted of the existence of an unusually short helix E in BraelkUSDA94SDgb2, a long helix H in BraelkUSDA3254SDgb1 and BrajapUSDA124SDgb1, and the existence of a pre-helix A followed by a long loop at the N-terminal of BraelkWSM1741SDgb2. Dataset 3 shows that among the predicted rhizobial SDgbs the distance of proximal H and distal Q/R/K/M to the heme Fe is 2.11 to 4.44 Å and 5.08 to 6.63 Å, respectively. This observation suggests that the heme Fe in rhizobial SDgbs is either penta- or hexacoordinate.

Only the globin domain from bacterial GCSs has been crystalized and analyzed by x-ray crystallography ^53, 54^ ( Dataset 3). Crystal structure for the bacterial GCSs transmitter domain has not been elucidated. Hence, we only predicted and analyzed the tertiary structure of globin domains from the selected rhizobial GCSs. Figure 5 shows that the predicted rhizobial GCSs globin domain exhibits a 1.5- to 3-turn pre-helix A, that (with the exception of SinfreGR64GCS) no loop exists between helices A and B, and that helix H is unusually long in Rhietl8C3GCS, RhietlCIAT652GCS2 and RhilegGB30GCS2. Dataset 3 shows that among the predicted rhizobial GCSs globin domain distance of proximal H/E and distal Q to the heme Fe is 1.77 to 5.56 Å and 4.09 to 9.04 Å, respectively. This observation suggests that the heme Fe in the rhizobial GCSs globin domain is either penta- or hexacoordinate.

Figure 6 to Figure 8 show that 2/2-globin folding is highly conserved in the predicted rhizobial tHbs class 1, class 2 and class 3. Major variations to 2/2-globin folding from predicted rhizobial tHbs consisted of the existence of a 2.5-turn pre-helix A followed by a long loop at the N-terminal of (class 1) CupnecN1tHb1 ( Figure 6); the existence of a one-turn pre-helix F (designated as φ in Figure 7 ^8^) in the rhizobial tHbs class 2; the existence of a long and extended C-terminal region in (class 2) BraelkUSDA94tHb1 ( Figure 7), and the substitution of helix A by a long loop that connects to helix B through a 1- to 2.5-turn pre-helix B in (class 3) BraelkUSDA76tHb2, BrajapUSDA123tHb1, BurphySTM815tHb1, MeslotNZP2037tHb2 and Sinmel1021tHb2 ( Figure 8). Dataset 3 shows that among the predicted rhizobial tHbs, the distance of proximal H and distal H/L/F to the heme Fe is 1.77 to 7.51 Å and 4.09 to 8.25 Å, respectively. This observation suggests that the heme Fe in the rhizobial tHbs is either penta- or hexacoordinate.

The above observations suggest that in spite of sequence variability (see the *Sequence alignments and phenetic analysis of rhizobial Glbs* subsection) the structure of rhizobial Glbs is similar to the canonical 3/3- or 2/2-globin folding of bacterial and non-bacterial Glbs. However, a number of predicted rhizobial Glbs exhibited variations at the N- and C-terminal regions suggesting that their structural properties could be different to those of canonical Glbs.

Data also shows that (with few exceptions) in addition to proximal and distal amino acids the distance of B10 and CD1 amino acids to the heme Fe and the orientation of proximal, distal, B10 and CD1 amino acids are similar within and among the predicted rhizobial SDgbs, fHbs and GCSs globin domain and tHbs. These amino acids participate in the binding of ligands to the heme Fe. Thus, these observations suggest that the mechanisms and chemistry for ligand binding are similar among the rhizobial Glbs.

### Spectroscopic identification of putative Glbs in soluble extracts from *Bradyrhizobium japonicum* USDA38 and USDA58

The prerequisites for being able to infer a protein’s function are isolating and characterizing either native or recombinant proteins and detecting protein synthesis *in vivo*. No rhizobial Glb has been isolated and characterized thus far. However, spectroscopic evidence indicates that putative Glbs exist in soluble extracts from *B. japonicum* 505 (Wisconsin), *R. leguminosarum* bv. viciae, *B. japonicum* NPK63 and *R. etli* CE3 (see the *Introduction* section). In order to extend these analyses to other rhizobia, we analyzed soluble extracts from *B. japonicum* USDA38 and USDA58 by (dithionite reduced + CO *minus* dithionite reduced) differential spectroscopy using as controls the sperm whale myoglobin and bovine blood hemoglobin. Table 3 shows that absorption peaks and troughs in the Soret and Q regions for the *B. japonicum* USDA38 and USDA58, *B. japonicum* 505 (Wisconsin), *R. leguminosarum* bv. viciae, *B. japonicum* NPK63 and *R. etli* CE3 soluble extracts, *Vitreoscilla* VHb, *E. coli* K12 Hmp, sperm whale myoglobin and bovine blood hemoglobin are nearly identical. This preliminary evidence indicates that putative soluble Glbs are synthesized in *B. japonicum* USDA38 and USDA58. Interestingly, genes coding for SDgbs ( *brajapUSDA38SDgb1* and *brajapUSDA38SDgb2*) and tHbs ( *brajapUSDA38tHb1* and *brajapUSDA38tHb2*) were identified in the *B. japonicum* USDA38 genome ( Dataset 1). Thus, it is likely that putative *B. japonicum* USDA38 Glbs corresponds to a combination of SDgbs and tHbs. Inferences from the preliminary results reported here should be confirmed by Glb detection, isolation and unequivocal identification after protein sequencing. This may open the possibility to carry out further experimental analyses on rhizobial Glbs.

**Table 3.** Absorption peaks and troughs in the Soret and Q regions from the (dithionite reduced + CO *minus* dithionite reduced) differential spectra of rhizobial soluble extracts and other bacterial and vertebrate Glbs.

Rhizobial soluble extract/Glb	Soret region		Q region				Reference
	Peak (nm)	Trough (nm)	Peak (nm)		Trough (nm)		
**Rhizobial soluble extracts**							
*B. japonicum* USDA38	425	448	535	573	549	600	This work
*B. japonicum* USDA58	416	437	535	573	554	601	This work
*B. japonicum* NPK63	422	443	529	574	558	598	24
*B. japonicum* 505 (Wisconsin)	417	434	540	569	556	n.i.	22
*R. etli* CE3	421	439	539	563	547	590	25
*R. leguminosarum* bv. viciae 96	424	443	535	574	555	n.i.	23
**Bacterial Glbs**							
*Vitreoscilla* VHb	418	436	534	567	551	590	25
*E. coli* K12 Hmp	420	437	530	570	555	592	55
**Vertebrate Glbs**							
Sperm whale myoglobin	419	436	538	578	558	596	This work
Bovine blood hemoglobin	417	432	533	570	554	588	This work

n.i., non-identified

## Conclusions

Rhizobial Glbs have been poorly studied. However, results reported in this work provide molecular and biochemical data from a bioinformatics perspective that contribute to a better understanding of these proteins. For example, the distribution and outline for the evolution of *glb* genes and Glb proteins among rhizobia was clarified, genes that could coexpress with the rhizobial *glb*s were identified and the predicted tertiary structure for rhizobial Glbs was elucidated. Also, spectroscopic analysis suggested that soluble Glbs are synthesized in free-living *B. japonicum* USDA38 and USDA58. This information will be useful in designing future experimental work focused on clarifying Glb functions within the physiology of free-living and symbiotic rhizobia.

## Data availability

The data referenced by this article are under copyright with the following copyright statement: Copyright: © 2015 Gesto-Borroto R et al.

Data associated with the article are available under the terms of the Creative Commons Zero "No rights reserved" data waiver (CC0 1.0 Public domain dedication). 

*F1000Research: Dataset 1. Globin genes detected in the genomes of rhizobial bacteria. 10.5256/f1000research.6392.d46189*

*F1000Research: Dataset 2. Predicted Glb polypeptides detected in the genomes of rhizobial bacteria. 10.5256/f1000research.6392.d46190*

*F1000Research: Dataset 3. Distance to the heme Fe and orientation of distal, proximal, B10 and CD1 amino acids in the predicted structure of selected rhizobial Glbs ( Table S2). 10.5256/f1000research.6392.d46191*

## Figures and Tables

**Table S1.** Classification of the α- and β-rhizobia and rhizobial genomes analyzed in this work.

α-rhizobia Domain Eubacteria Phylum Proteobacteria Class Alphaproteobacteria Order Rhizobiales			
Family	Genus	Species/strain	Database
Bradyrhizobiaceae	*Bradyrhizobium*	*B. elkanii* USDA3254, 587, USDA3259, USDA76, USDA94, WSM1741, WSM2783 *B. japonicum* USDA110, 22, USDA122, USDA123, USDA124, USDA 135, USDA38, USDA4, USDA6-7488, USDA6-8372, WSM1743, WSM2793, in8p8, is5	JGI Genome Portal ( http://genome.jgi.doe.gov/) Rhizobase ( http://genome.microbedb. jp/rhizobase)
Phyllobacteriaceae	*Mesorhizobium*	*M. ciceri* CMG6, WSM4083, bv. biserrulae WSM1271 *M. loti* CJ3sym, MAFF303099, NZP2037, R7A, R88b, USDA3471	JGI Genome Portal ( http://genome.jgi.doe.gov/) Rhizobase ( http://genome.microbedb. jp/rhizobase)
Rhizobiaceae	*Rhizobium*	*R. etli* 8C-3, Brasil 5, CFN42, CIAT652, CIAT894, CNPAF512, GR56, IE4771, Kim5 *R. leguminosarum* bv. viciae UPM1131, bv. viciae UPM1137, bv. viciae 128C53, bv. viciae GB30, bv. viciae VF39, bv. viciae WSM1455, bv. viciae WSM1481, bv. viciae 248, bv. viciae 3841, bv. viciae Ps8, bv. viciae TOM, bv. viciae Vh3, bv. viciae Vc2 *R. lupini* HPC(L)	CCG-UNAM ( http://www.cifn.unam.mx/) Sanger Institute ( http://www.sanger.ac.uk) NEERI ( http://www.neeri.res.in/)
	*Sinorhizobium*	*S. fredii* GR64, HH103, USDA257 *S. meliloti* 1021	GGL ( http://appmibio.uni-goettingen.de/) INRA ( https://iant.toulouse.inra.fr/)
Xanthobacteraceae	*Azorhizobium*	*A. doebereinerae* UFLA1-100	JGI Genome Portal ( http://genome.jgi.doe.gov/)
β-rhizobia Domain Eubacteria Phylum Proteobacteria Class Betaproteobacteria Order Burkholderiales			
Family	Genus	Species/strain	Database
Burkholderiaceae	*Burkholderia*	*B. phymatum* STM815	JGI Genome Portal ( http://genome.jgi.doe.gov/)
	*Cupriavidus*	C. necator* HPC(L), JMP134, N-1 ATCC43291	Goettingen Genomics Laboratory ( http:// appmibio.uni-goettingen.de/)

*Formerly classified as *Alcaligenes eutrophus* and *Ralstonia eutropha*.

**Table S2.** Predicted structures of rhizobial Glbs deposited in the Caspur protein model dataBase ( http://bioinformatics.cineca.it/PMDB/).

Glb	ID number
**fHbs**	
BurphySTM815fHb	PM0079658
CupnecHPC(L)fHb	PM0079659
CupnecJMP134fHb	PM0079660
CupnecN-1 ATCC43291fHb1	PM0079661
CupnecN-1 ATCC43291fHb2	PM0079662
RhilegUPM1137fHb	PM0079663
Sinmel1021fHb	PM0079672
**SDgbs**	
AzodoeUFLA1-100SDgb	PM0079664
BraelkUSDA94SDgb2	PM0079665
BraelkUSDA3254SDgb1	PM0079666
BraelkUSDA3254SDgb2	PM0079667
BraelkUSDA3259SDgb1	PM0079668
BraelkWSM1741SDgb2	PM0079669
BrajapUSDA38SDgb2	PM0079670
BrajapUSDA124SDgb1	PM0079671
**GCSs**	
Brajapin8p8GCS globin domain	PM0079673
RhietlCIAT652GCS1 globin domain	PM0079674
RhietlCIAT652GCS2 globin domain	PM0079675
Rhietl8C-3GCS globin domain	PM0079676
RhietlCFN42DSM 11541GCS1 globin domain	PM0079677
RhilegGB30GCS1 globin domain	PM0079678
RhilegGB30GCS2 globin domain	PM0079679
SinfreGR64GCS globin domain	PM0079680
Sinmel1021GCS globin domain	PM0079681
**tHbs**	
AzodoeUFLA1-100tHb1	PM0079682
AzodoeUFLA1-100tHb2	PM0079683
BraelkUSDA76tHb2	PM0079684
BraelkUSDA94tHb1 (globin domain + C-terminal extension)	PM0079701
BrajapUSDA38tHb2	PM0079685
BrajapUSDA123tHb1	PM0079686
BurphySTM815tHb1	PM0079687
BurphySTM815tHb2	PM0079688
CupnecN-1 ATCC43291tHb1	PM0079689
CupnecN-1 ATCC43291tHb2	PM0079690
MescicCMG6tHb	PM0079691
MeslotNZP2037tHb2	PM0079692
RhietlCNPAF512tHb	PM0079693
RhietlKim5tHb	PM0079694
RhilegGB30tHb2	PM0079695
RhilegVc2tHb1	PM0079696
RhilupHPC(L)tHb1	PM0079697
RhilupHPC(L)tHb2	PM0079698
SinfreHH103tHb	PM0079699
Sinmel1021tHb2	PM0079700

## Supplementary materials

DNA fragments correspond to ~5 kb up- and downstream to *glb* genes. Arrows indicate the transcription orientation. The *glb* genes are shown in red, predicted polypeptides functioning in nitrogen metabolism are shown in blue and predicted polypeptides functioning in chemotaxis are shown in green. The ORF sizes and distances between ORFs are shown at an approximate scale. Abbreviations for the predicted polypeptides are indicated at the end of the figure. Location of DNA fragments in the rhizobial genome: Chr, chromosome; Pmd, plasmid; n.i., non-identified.

The tHb, fHb, SDgb and GCS sequences are shown in green, light blue, dark blue and red, respectively. Distal and proximal amino acids located in helices E and F, respectively, are indicated within black boxes; potential distal E7 and E11 are indicated in the tHb sequences. Amino acids that interact with FAD and NAD(P) ^+^ cofactors in the fHb flavin domain are indicated within gray boxes. Limits for the globin domain are indicated with right- and left-oriented arrows within black circles. Helices are indicated with letters A to H within the 2/2- and 3/3-fold of the tHb and SDgb and fHb and GCS globin domains, respectively. Outgroups for fHbs correspond to BacsubfHb, EsccolfHb and SaccerfHb (Genbank accession number YP_003865693, NP_289108 and NP_011750, respectively); outgroup for SDgbs corresponds to VitSDgb (Genbank accession number AAA75506); outgroups for GCSs correspond to AgrtumGCS and BacsubGCS (Genbank accession number NP_354049 and NP_388919, respectively); outgroups for tHbs correspond to MyctubtHb class1, MyctubtHb class 2, AgrtumtHb class 2 and MycavitHb class 3 (Genbank accession number NP_216058, NP_216986, WP_020813663 and BAN32501, respectively).
