# Supplementary material for: A bioinformatics insight to rhizobial globins: gene identification and mapping, polypeptide sequence and phenetic analysis, and protein modeling
Source: F1000Res. 2015 May 13;4:117. [Version 1] doi: 10.12688/f1000research.6392.1 (PMC4648194; doi:10.12688/f1000research.6392.1)
Supplement: Supplementary file 4 [file f1000research-4-6858-s0003.tgz › 9115a3bb-4d31-4698-ba26-bb25b55f4ae0.pdf]

Figure S1
Gesto-Borroto et al.

(A)

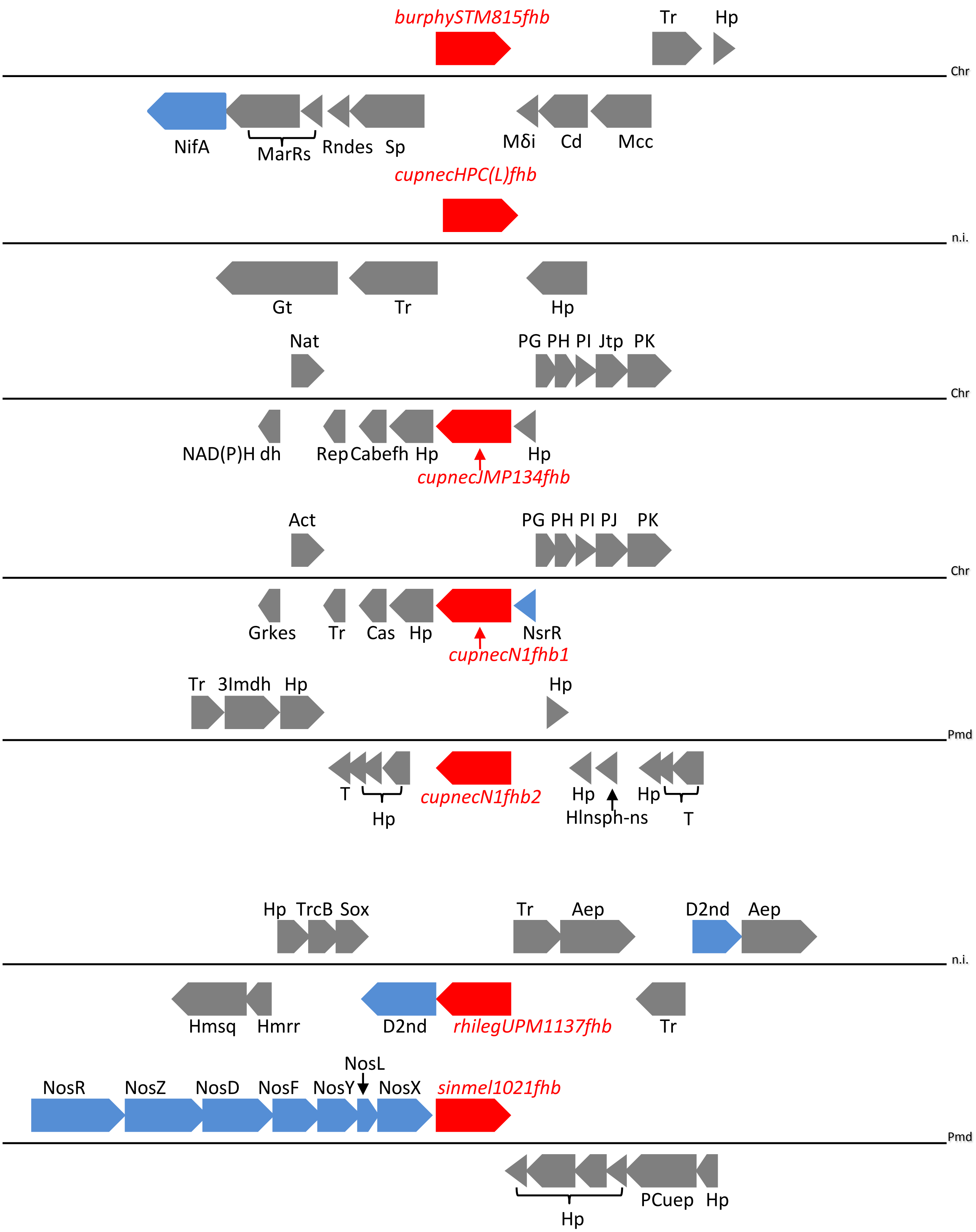

(B)

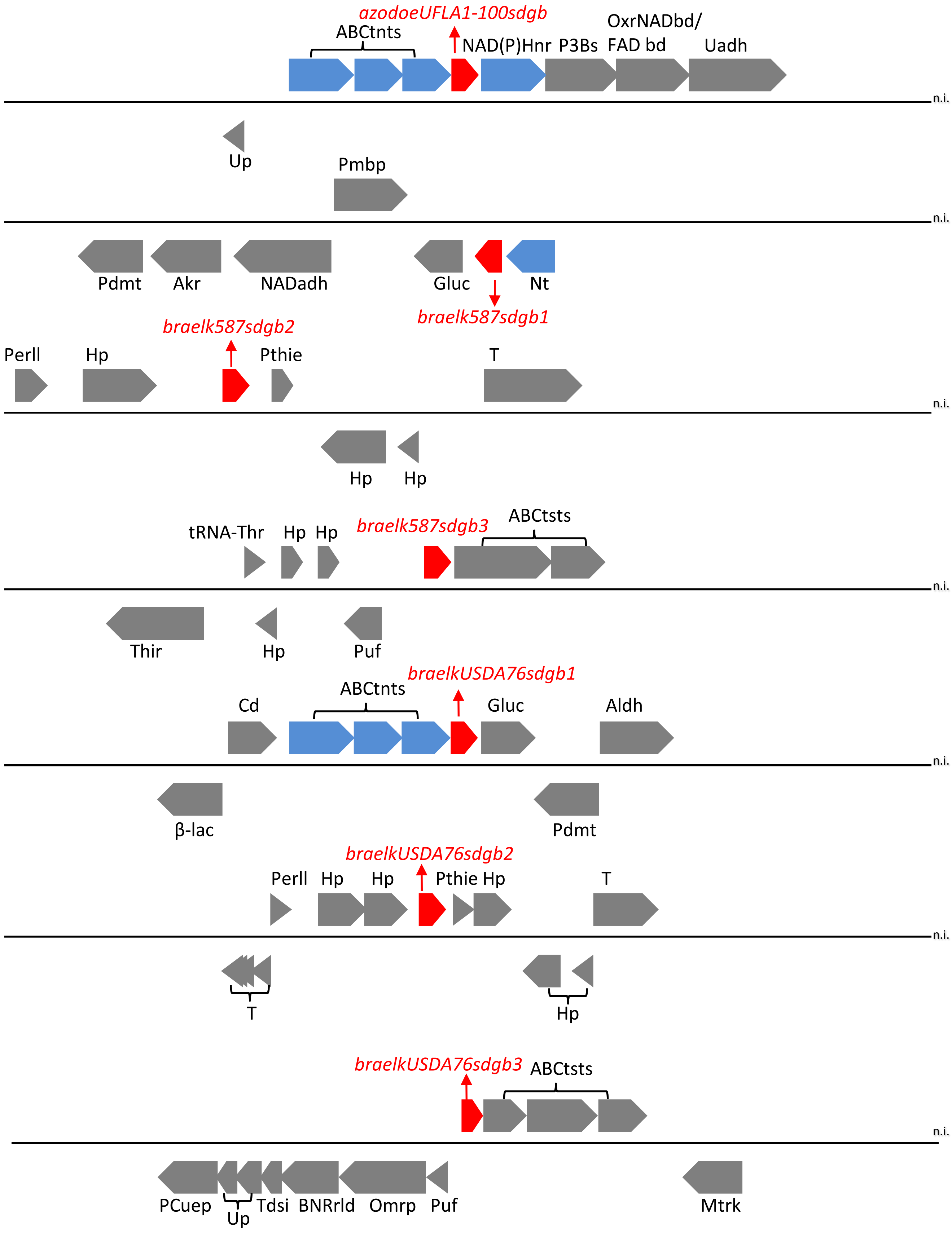
azodoeUFLA1-100sdgb
ABCtnts
NAD(P)Hnr
P3Bs
OxrNADbd/
FAD bd
Uadh
n.i.
Up
Pmbp
n.i.
Pdmt
Akr
NADadh
Gluc
Nt
braelk587sdgb1
braelk587sdgb2
Perll
Hp
Pthie
T
n.i.
Hp
Hp
tRNA-Thr
Hp
Hp
braelk587sdgb3
ABCtsts
n.i.
Thir
Hp
Puf
braelkUSDA76sdgb1
Cd
ABCtnts
Gluc
Aldh
n.i.
β-lac
Pdmt
braelkUSDA76sdgb2
Perll
Hp
Hp
Pthie Hp
T
n.i.
T
Hp
braelkUSDA76sdgb3
ABCtsts
n.i.
PCuep
Up
Tdsi
BNRrld
Omrp
Puf
Mtrk

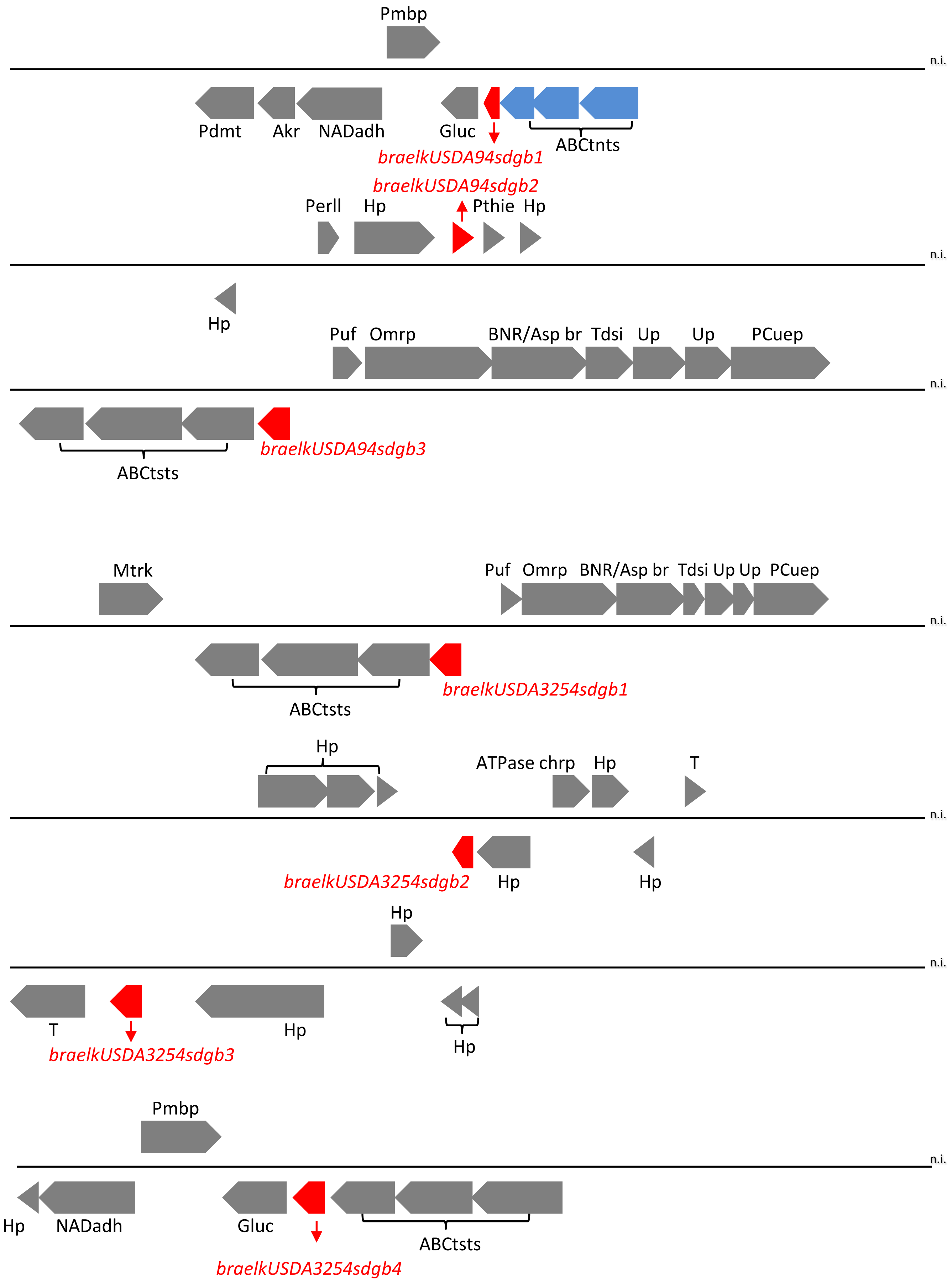
Pmbp
n.i.
Pdmt
Akr
NADadh
Gluc
braelkUSDA94sdgb1
ABCtnts
braelkUSDA94sdgb2
Perll
Hp
Pthie
Hp
n.i.
Hp
Puf
Omrp
BNR/Asp br
Tdsi
Up
Up
PCuep
n.i.
braelkUSDA94sdgb3
ABCtsts
Mtrk
Puf
Omrp
BNR/Asp br
Tdsi
Up
Up
PCuep
n.i.
braelkUSDA3254sdgb1
ABCtsts
Hp
ATPase chrp
Hp
T
n.i.
braelkUSDA3254sdgb2
Hp
Hp
Hp
n.i.
T
braelkUSDA3254sdgb3
Hp
Hp
Pmbp
n.i.
Hp
NADadh
Gluc
ABCtsts
braelkUSDA3254sdgb4

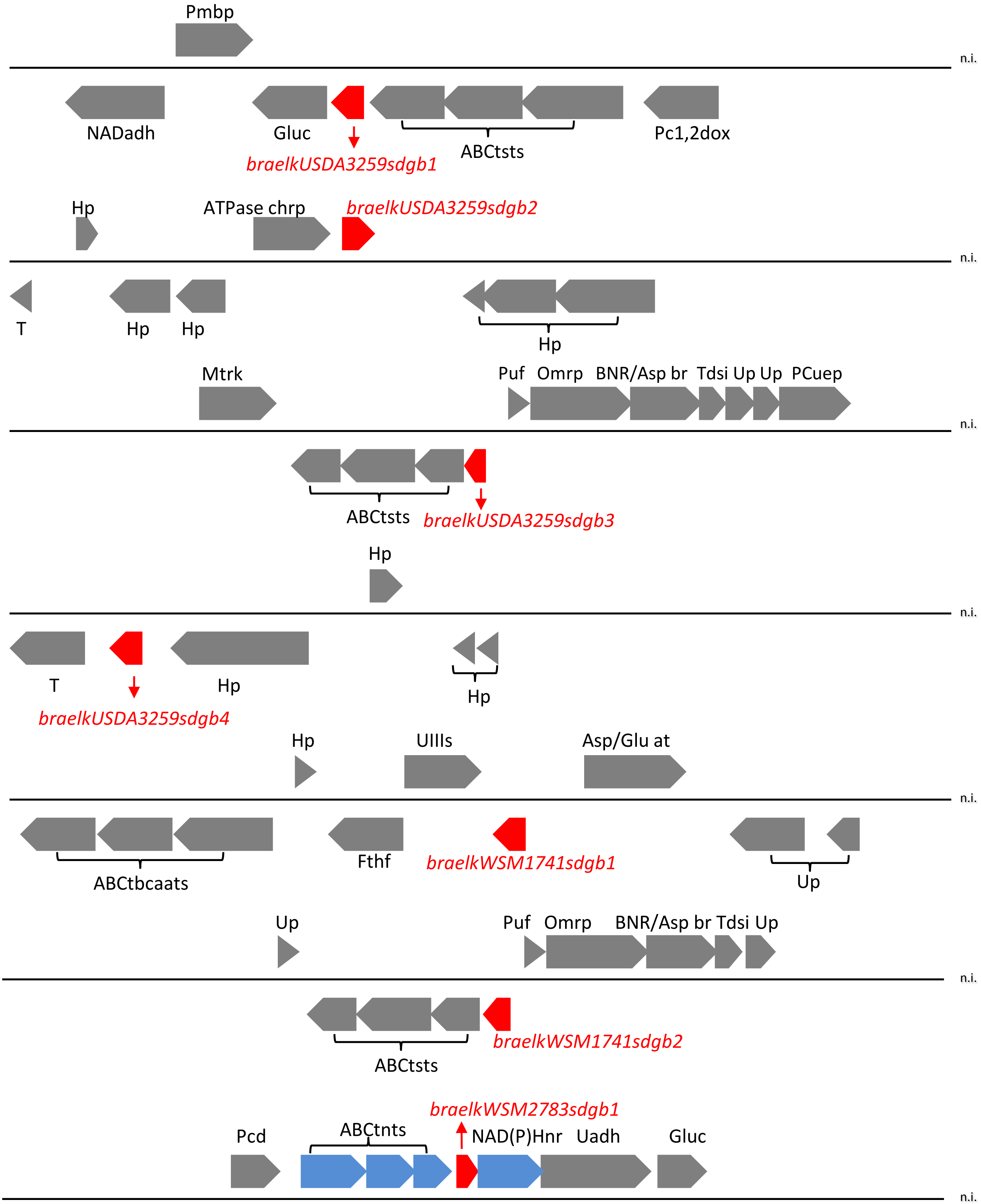

Pmbp
n.i.
NADadh
Gluc
braelkUSDA3259sdgb1
ABCtsts
Pc1,2dox
Hp
ATPase chrp
braelkUSDA3259sdgb2
n.i.
T
Hp
Hp
Hp
Mtrk
Puf
Omrp
BNR/Asp br
Tdsi
Up
Up
PCuep
n.i.
ABCtsts
braelkUSDA3259sdgb3
Hp
n.i.
T
Hp
braelkUSDA3259sdgb4
Hp
Hp
UIIIs
Asp/Glu at
n.i.
ABCtbcaats
Fthf
braelkWSM1741sdgb1
Up
Up
Puf
Omrp
BNR/Asp br
Tdsi
Up
n.i.
ABCtsts
braelkWSM1741sdgb2
braelkWSM2783sdgb1
Pcd
ABCtnts
NAD(P)Hnr
Uadh
Gluc
n.i.

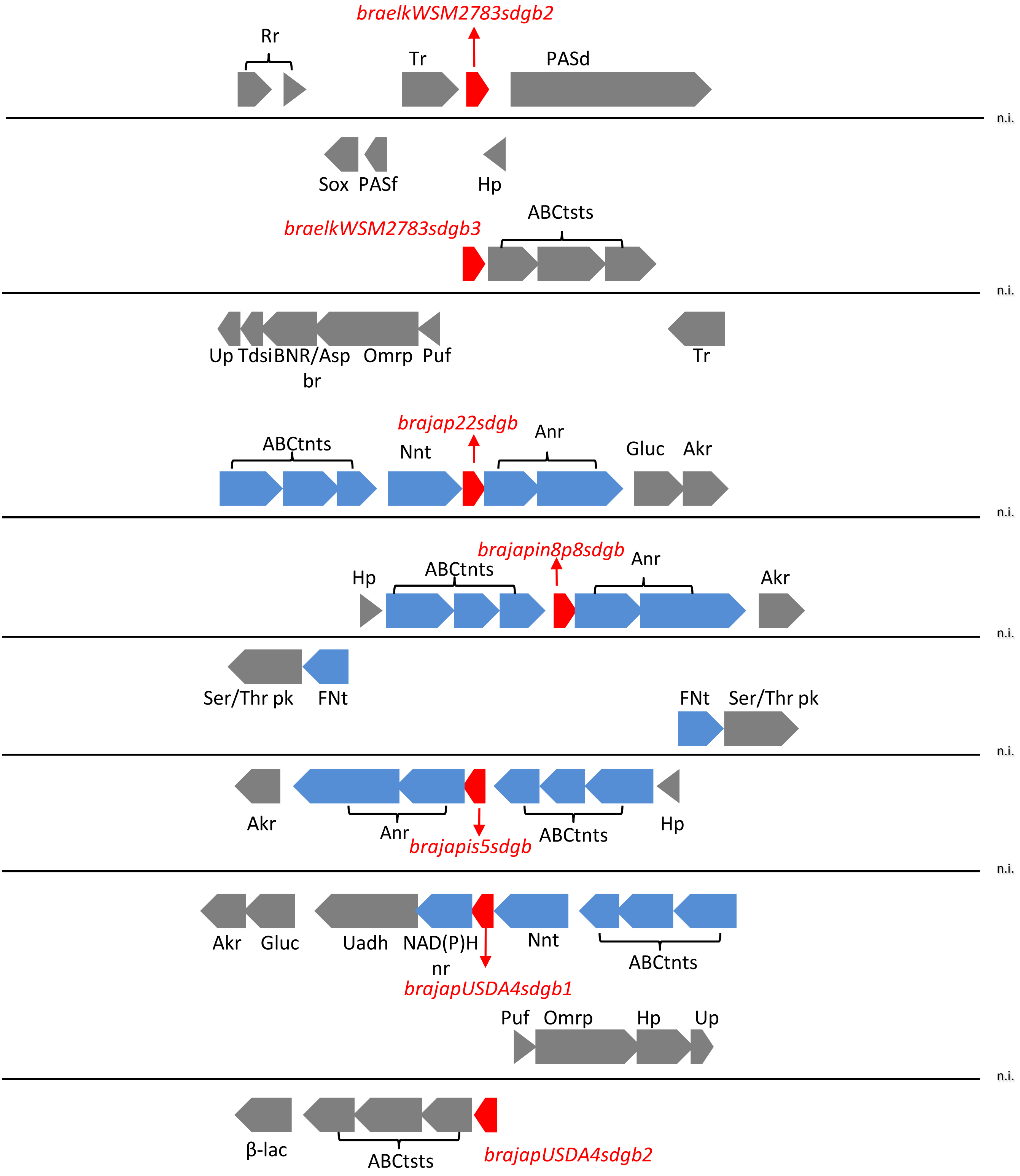
braelkWSM2783sdgb2
Rr
Tr
PASd
n.i.
Sox
PASf
Hp
ABCtsts
braelkWSM2783sdgb3
n.i.
Up
TdsiBNR/Asp
br
Omrp
Puf
Tr
brajap22sdgb
Anr
ABCtnts
Nnt
Gluc
Akr
n.i.
brajapin8p8sdgb
Anr
Hp
ABCtnts
Akr
n.i.
Ser/Thr pk
FNt
FNt
Ser/Thr pk
n.i.
Akr
Anr
brajapis5sdgb
ABCtnts
Hp
n.i.
Akr
Gluc
Uadh
NAD(P)H
nr
Nnt
ABCtnts
brajapUSDA4sdgb1
Puf
Omrp
Hp
Up
n.i.
β-lac
ABCtsts
brajapUSDA4sdgb2

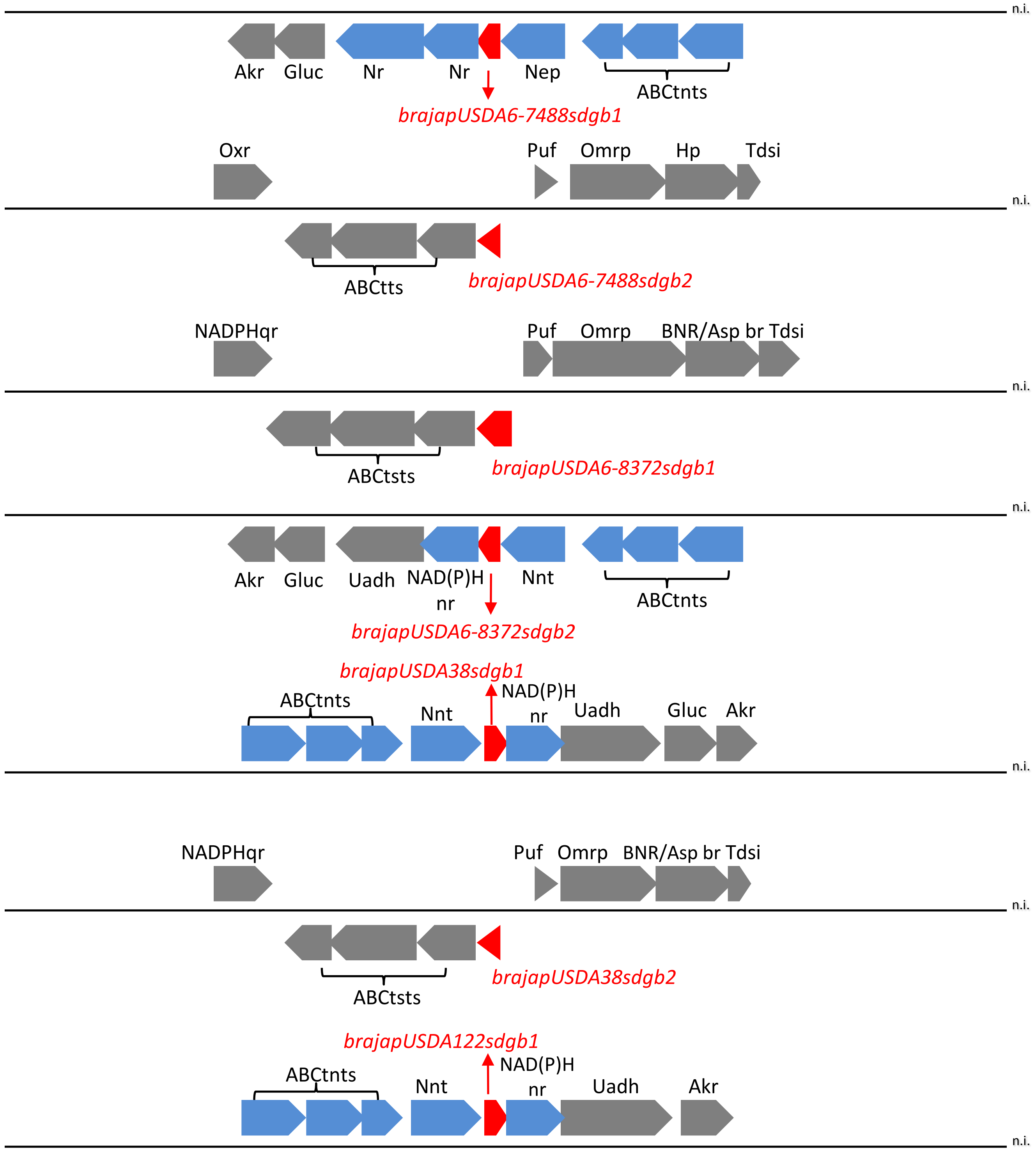
n.i.
Akr
Gluc
Nr
Nr
Nep
ABCtnts
brajapUSDA6-7488sdgb1
Oxr
Puf
Omrp
Hp
Tdsi
n.i.
ABCtts
brajapUSDA6-7488sdgb2
NADPHqr
Puf
Omrp
BNR/Asp br Tdsi
n.i.
ABCtsts
brajapUSDA6-8372sdgb1
n.i.
Akr
Gluc
Uadh
NAD(P)H nr
Nnt
ABCtnts
brajapUSDA6-8372sdgb2
brajapUSDA38sdgb1
ABCtnts
Nnt
NAD(P)H nr
Uadh
Gluc
Akr
n.i.
NADPHqr
Puf
Omrp
BNR/Asp br Tdsi
n.i.
ABCtsts
brajapUSDA38sdgb2
brajapUSDA122sdgb1
ABCtnts
Nnt
NAD(P)H nr
Uadh
Akr
n.i.

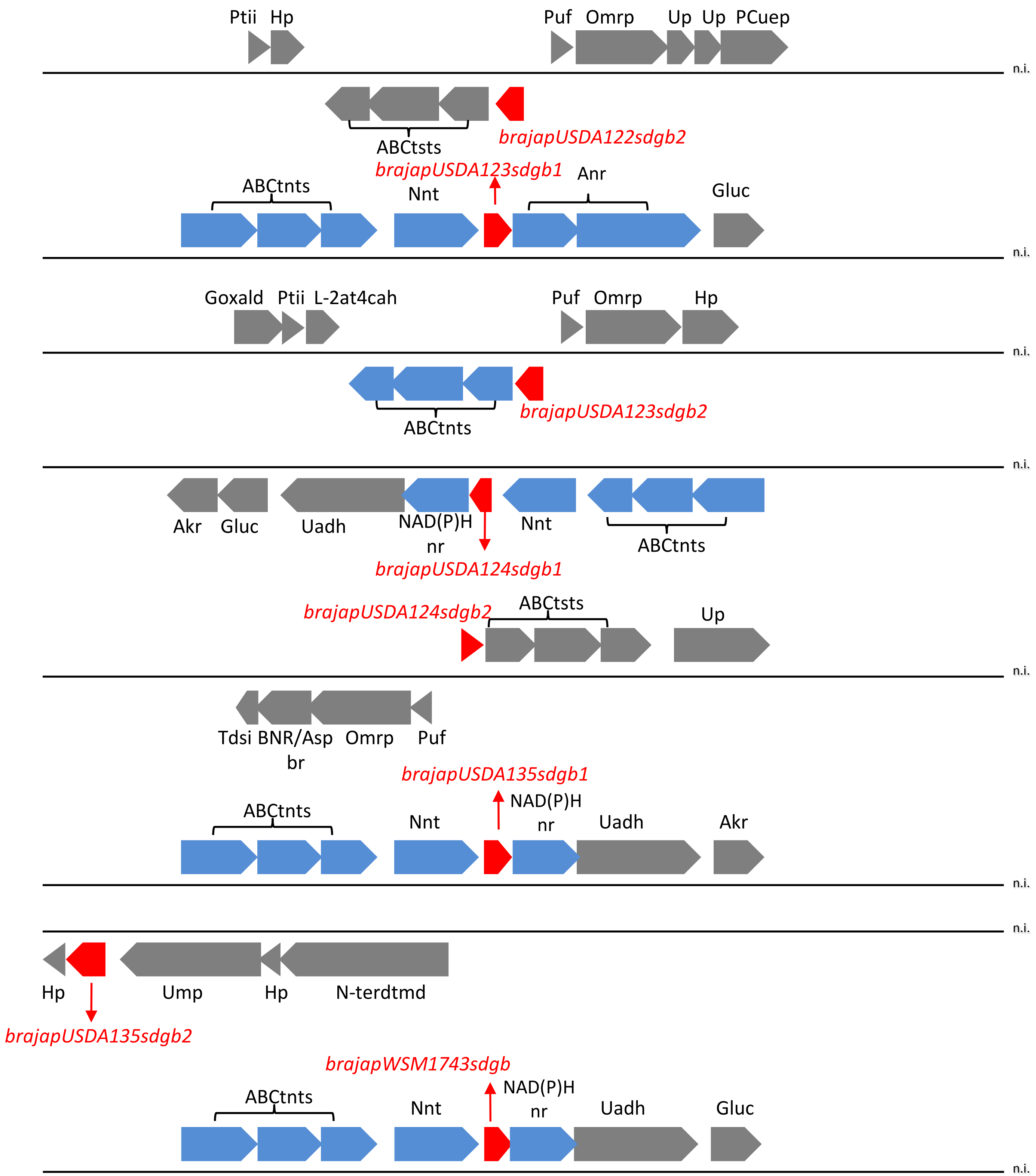

Ptii Hp
Puf Omrp Up Up PCuep
n.i.
ABCtsts
brajapUSDA122sdgb2
brajapUSDA123sdgb1
ABCtnts
Nnt
Anr
Gluc
n.i.
Goxald Ptii L-2at4cah
Puf Omrp Hp
n.i.
ABCtnts
brajapUSDA123sdgb2
n.i.
Akr Gluc Uadh
NAD(P)H
nr
Nnt
ABCtnts
brajapUSDA124sdgb1
brajapUSDA124sdgb2
ABCtsts
Up
n.i.
Tdsi BNR/Asp Omrp Puf
br
brajapUSDA135sdgb1
ABCtnts
Nnt
NAD(P)H
nr
Uadh
Akr
n.i.
n.i.
Hp Ump Hp N-terdtmd
brajapUSDA135sdgb2
brajapWSM1743sdgb
ABCtnts
Nnt
NAD(P)H
nr
Uadh
Gluc
n.i.

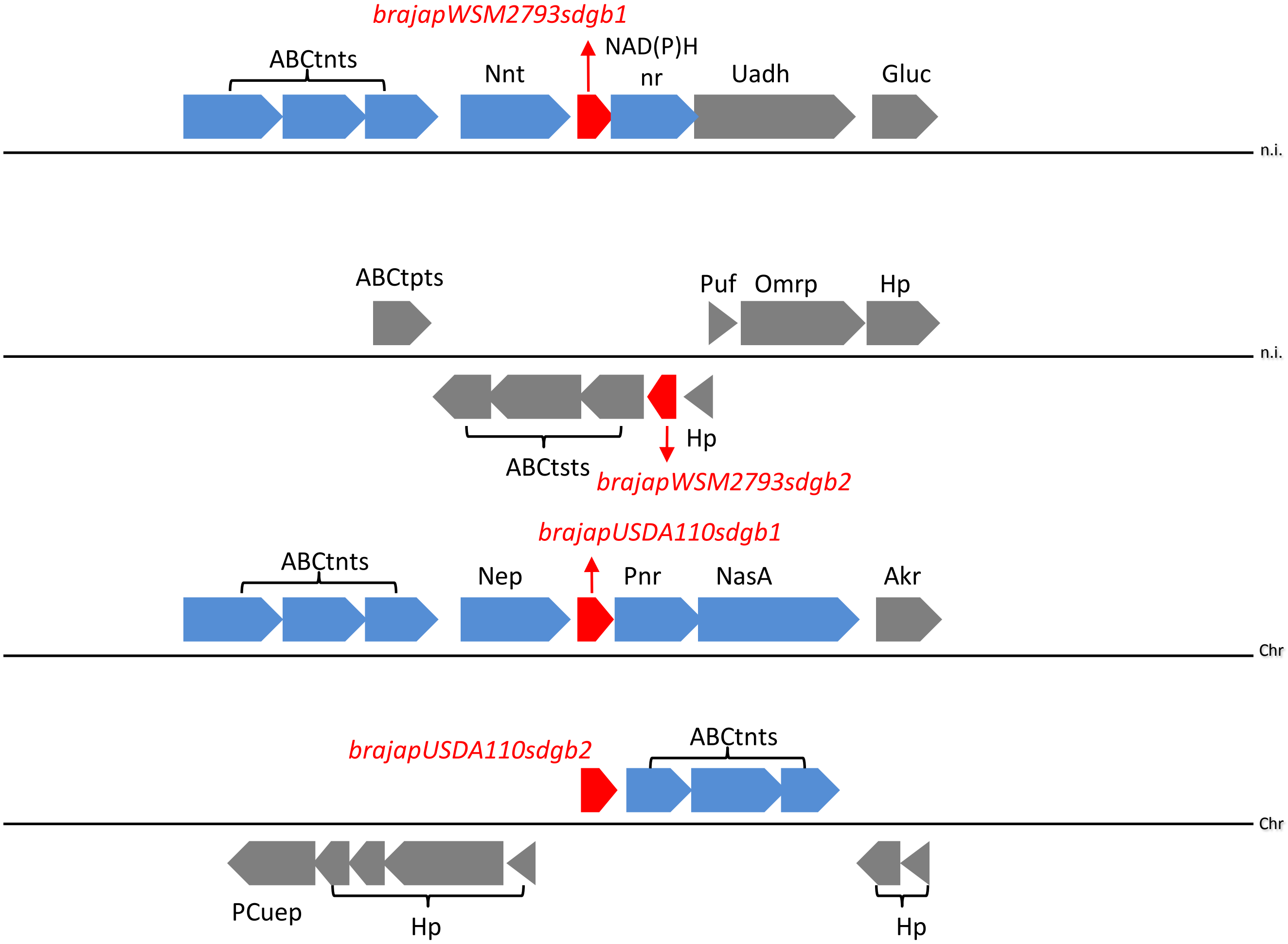
brajapWSM2793sdgb1
ABCtnts
Nnt
NAD(P)H
nr
Uadh
Gluc
n.i.
ABCtpts
Puf
Omrp
Hp
n.i.
Hp
ABCtsts
brajapWSM2793sdgb2
brajapUSDA110sdgb1
ABCtnts
Nep
Pnr
NasA
Akr
Chr
brajapUSDA110sdgb2
ABCtnts
Chr
PCuep
Hp
Hp

(C)

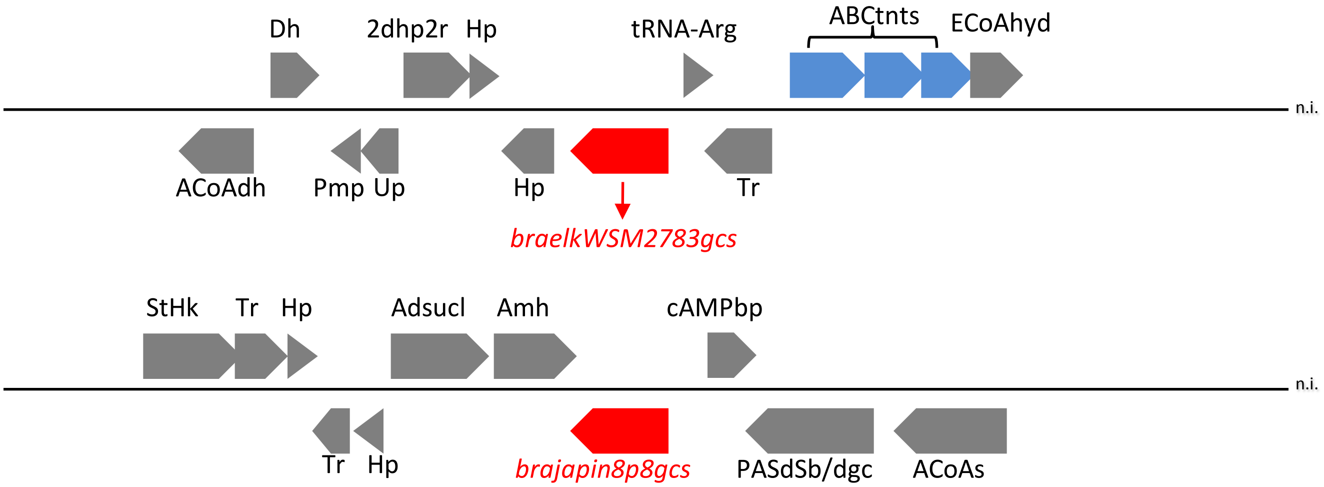
Dh
2dhp2r
Hp
tRNA-Arg
ABCtnts
ECoAhyd
n.i.
ACoAdh
Pmp
Up
Hp
Tr
braelkWSM2783gcs
StHk
Tr
Hp
Adsucl
Amh
cAMPbp
n.i.
Tr
Hp
brajapin8p8gcs
PASdSb/dgc
ACoAs

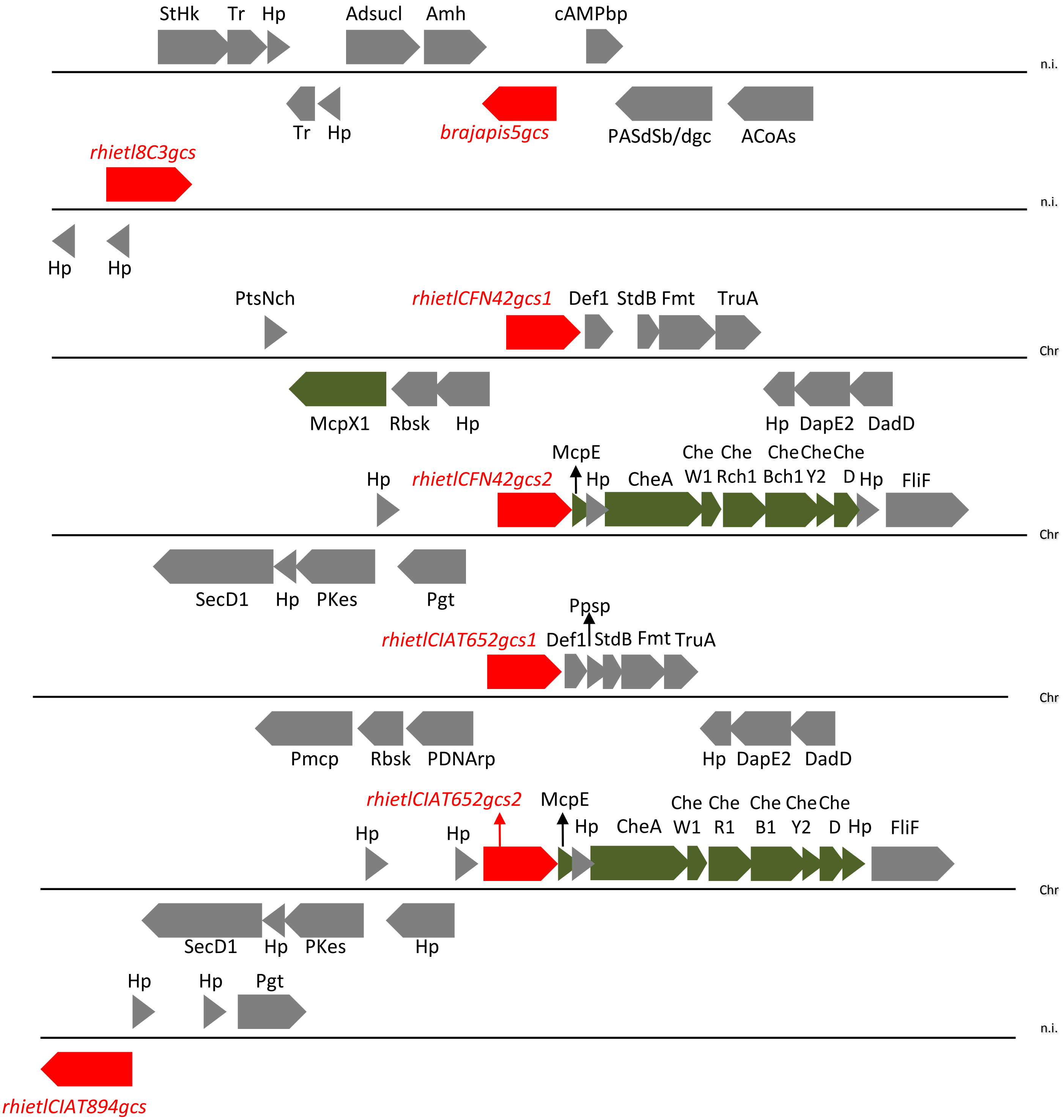

StHk Tr Hp Adsucl Amh cAMPbp
n.i.
Tr Hp brajapis5gcs PASdSb/dgc ACoAs
rhietl8C3gcs
n.i.
Hp Hp
PtsNch rhietlCFN42gcs1 Def1 StdB Fmt TruA
Chr
McpX1 Rbsk Hp Hp DapE2 DadD
Hp rhietlCFN42gcs2 McpE Hp CheA Che W1 Che Rch1 Che Bch1 Che Y2 Che D Hp FliF
Chr
SecD1 Hp PKes Pgt
rhietlCIAT652gcs1 Def1 Ppsp StdB Fmt TruA
Chr
Pmcp Rbsk PDNArp Hp DapE2 DadD
Hp Hp rhietlCIAT652gcs2 McpE Hp CheA Che W1 Che R1 Che B1 Che Y2 Che D Hp FliF
Chr
SecD1 Hp PKes Hp
Hp Hp Pgt
n.i.
rhietlCIAT894gcs

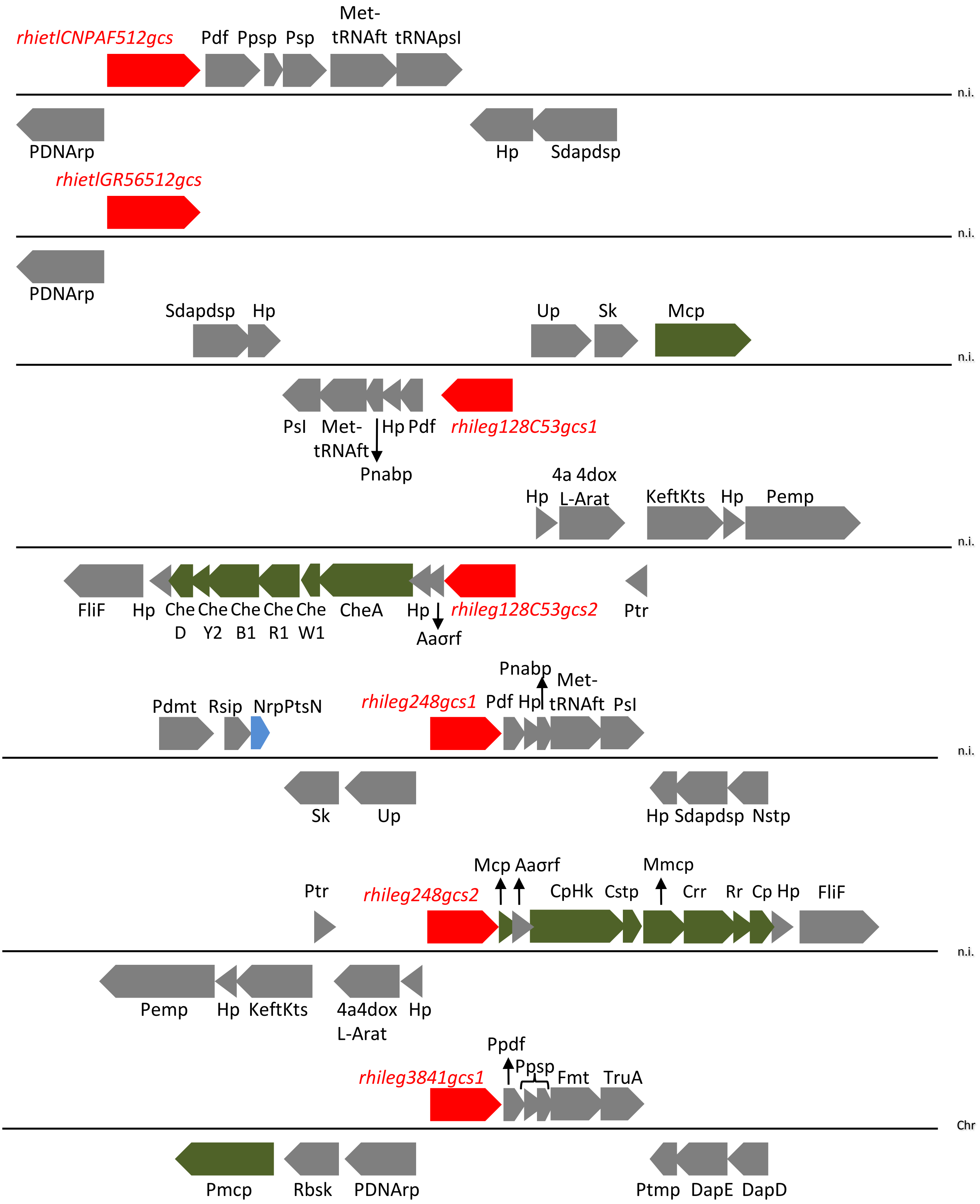
rhietlCNPAF512gcs
Pdf Ppsp Psp Met-tRNAft tRNApsI
n.i.
PDNArp
Hp Sdapdsp
rhietlGR56512gcs
n.i.
PDNArp
Sdapdsp Hp
Up Sk Mcp
n.i.
PsI Met-tRNAft Hp Pdf
Pnabp
rhileg128C53gcs1
4a 4dox
Hp L-Arat
KeftKts Hp Pemp
n.i.
FliF Hp Che D Che Y2 Che B1 Che R1 Che W1 CheA Hp
Aaσrf
rhileg128C53gcs2
Ptr
Pnabp
Pdmt Rsip NrpPtsN
rhileg248gcs1
Pdf Hp
Met-tRNAft PsI
n.i.
Sk Up
Hp Sdapdsp Nstp
Mcp Aaσrf
Mmcp
Ptr
rhileg248gcs2
CpHk Cstp Crr Rr Cp Hp FliF
n.i.
Pemp Hp KeftKts 4a4dox L-Arat Hp
Ppdf
Ppsp
rhileg3841gcs1
Fmt TruA
Chr
Pmcp Rbsk PDNArp
Ptmp DapE DapD

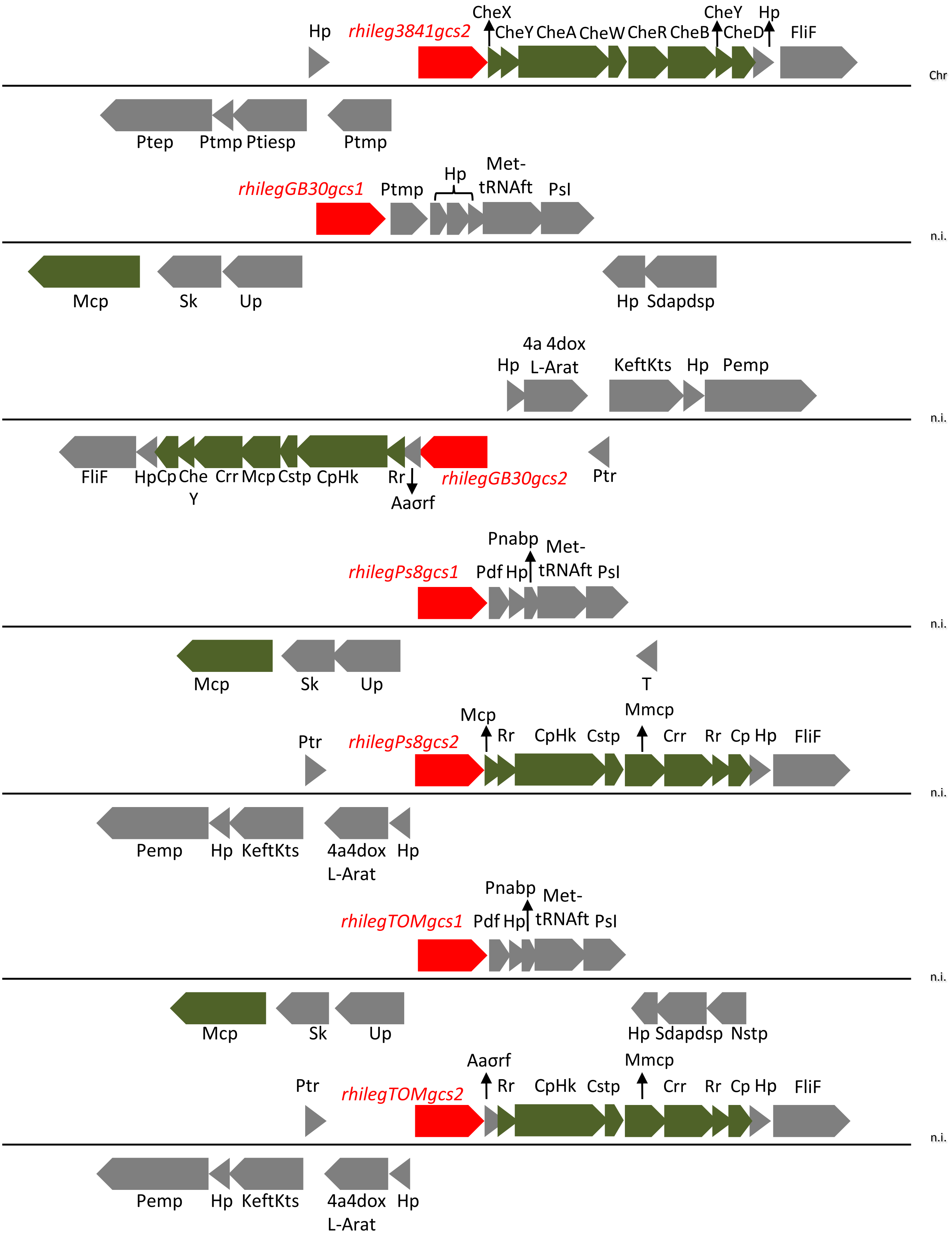

CheX
CheY
Hp
Hp
rhileg3841gcs2
CheY CheA CheW CheR CheB
CheD
FliF
Chr
Ptep
Ptmp Ptiesp
Ptmp
Met-
tRNAft
Hp
rhilegGB30gcs1
Ptmp
PsI
n.i.
Mcp
Sk
Up
Hp
Sdapdsp
4a 4dox
L-Arat
Hp
KeftKts
Hp
Pemp
n.i.
FliF
Hp Cp Che Y
Crr Mcp Cstp CpHk
Rr
Aaorf
rhilegGB30gcs2
Ptr
Pnabp
Met-
tRNAft
rhilegPs8gcs1
Pdf Hp
PsI
n.i.
Mcp
Sk
Up
T
Mcp
Mmcp
Ptr
rhilegPs8gcs2
Rr
CpHk
Cstp
Crr
Rr
Cp Hp
FliF
n.i.
Pemp
Hp
KeftKts
4a4dox
L-Arat
Hp
Pnabp
Met-
tRNAft
rhilegTOMgcs1
Pdf Hp
PsI
n.i.
Mcp
Sk
Up
Hp Sdapdsp Nstp
Aaorf
Mmcp
Ptr
rhilegTOMgcs2
Rr
CpHk
Cstp
Crr
Rr
Cp Hp
FliF
n.i.
Pemp
Hp
KeftKts
4a4dox
L-Arat
Hp

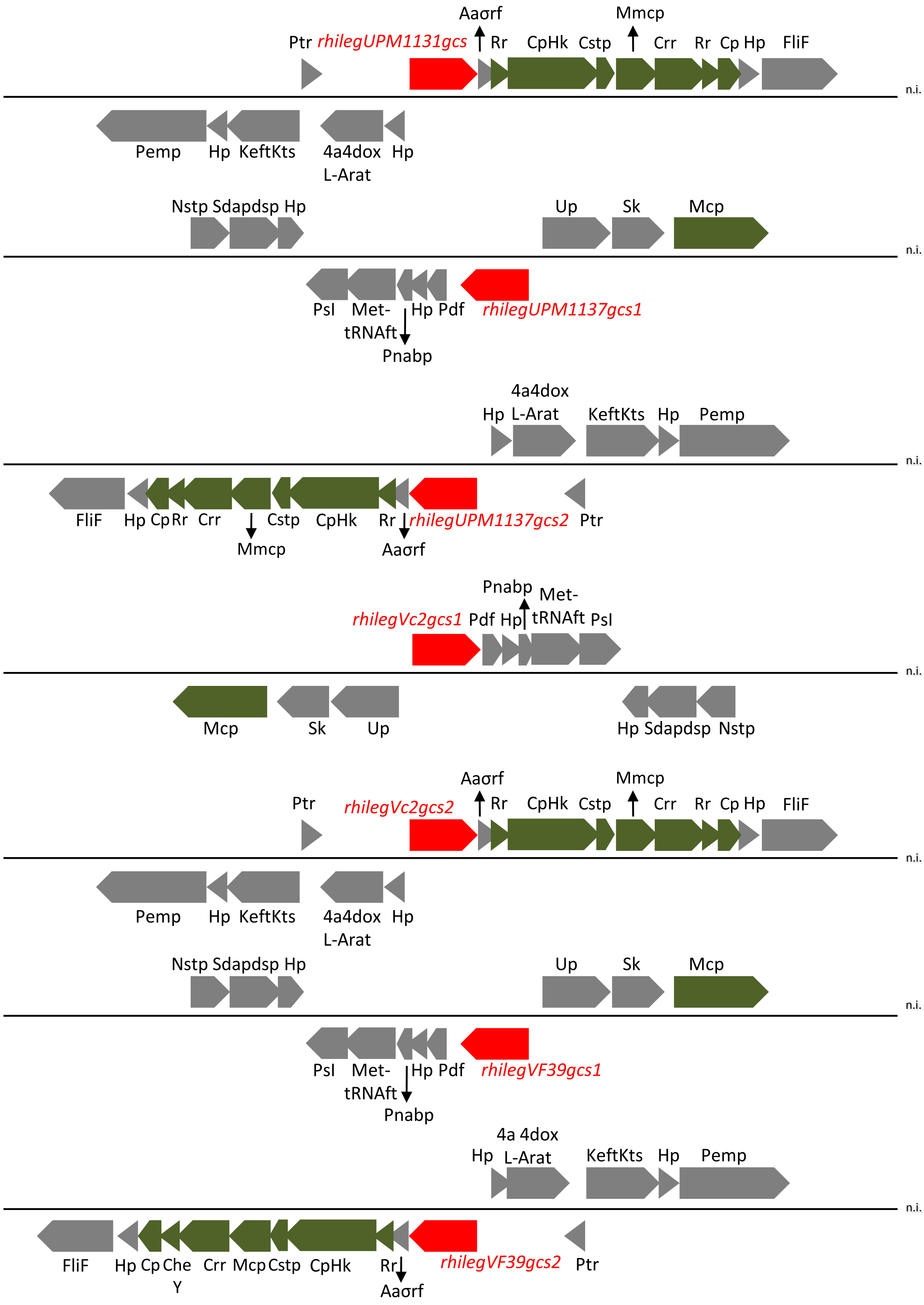
Aaσrf
Mmcp
Ptr rhilegUPM1131gcs
Rr
CpHk
Cstp
Crr
Rr
Cp
Hp
FliF
n.i.
Pemp
Hp
KeftKts
4a4dox
L-Arat
Hp
Nstp Sdapdsp Hp
Up
Sk
Mcp
n.i.
PsI
Met-
tRNAft
Hp Pdf
rhilegUPM1137gcs1
Pnabp
4a4dox
Hp L-Arat
KeftKts
Hp
Pemp
n.i.
FliF
Hp Cp Rr
Crr
Cstp
CpHk
Rr
rhilegUPM1137gcs2
Ptr
Mmcp
Aaσrf
Pnabp
Met-
rhilegVc2gcs1
Pdf Hp
tRNAft
PsI
n.i.
Mcp
Sk
Up
Hp Sdapdsp Nstp
Aaσrf
Mmcp
Ptr
rhilegVc2gcs2
Rr
CpHk
Cstp
Crr
Rr
Cp
Hp
FliF
n.i.
Pemp
Hp
KeftKts
4a4dox
L-Arat
Hp
Nstp Sdapdsp Hp
Up
Sk
Mcp
n.i.
PsI
Met-
tRNAft
Hp Pdf
rhilegVF39gcs1
Pnabp
4a 4dox
Hp L-Arat
KeftKts
Hp
Pemp
n.i.
FliF
Hp Cp Che
Y
Crr
Mcp
Cstp
CpHk
Rr
Aaσrf
rhilegVF39gcs2
Ptr

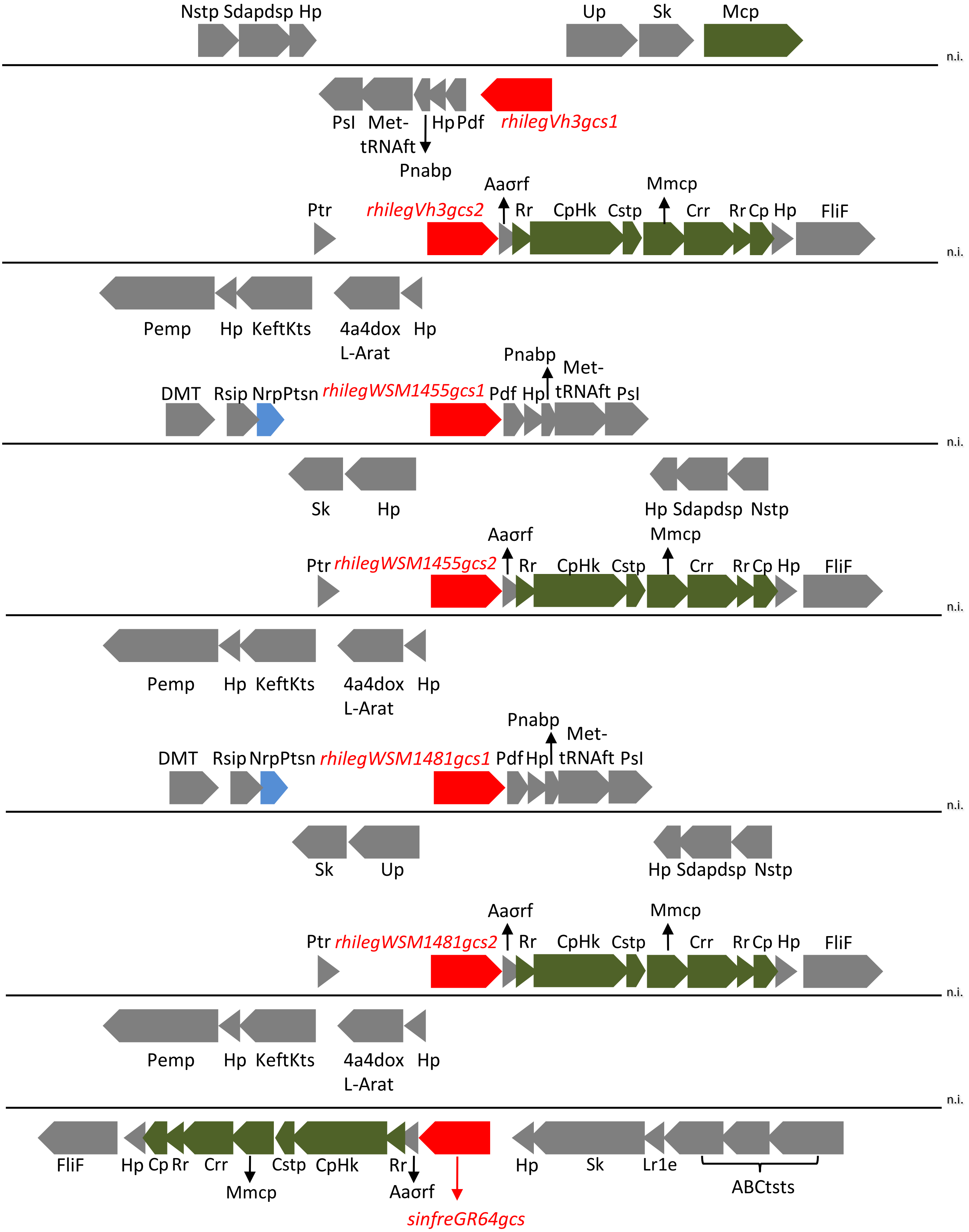
Nstp Sdapdsp Hp
Up
Sk
Mcp
n.i.
PsI
Met-
tRNAft
Hp Pdf
Pnabp
rhilegVh3gcs1
Aaσrf
Mmcp
Ptr
rhilegVh3gcs2
Rr
CpHk
Cstp
Crr
Rr Cp Hp
FliF
n.i.
Pemp
Hp
KeftKts
4a4dox
L-Arat
Hp
Pnabp
Met-
DMT
Rsip NrpPtsn
rhilegWSM1455gcs1
Pdf Hp
tRNAft
PsI
n.i.
Sk
Hp
Hp Sdapdsp Nstp
Aaσrf
Mmcp
Ptr
rhilegWSM1455gcs2
Rr
CpHk
Cstp
Crr
Rr Cp Hp
FliF
n.i.
Pemp
Hp
KeftKts
4a4dox
L-Arat
Hp
Pnabp
Met-
DMT
Rsip NrpPtsn
rhilegWSM1481gcs1
Pdf Hp
tRNAft
PsI
n.i.
Sk
Up
Hp Sdapdsp Nstp
Aaσrf
Mmcp
Ptr
rhilegWSM1481gcs2
Rr
CpHk
Cstp
Crr
Rr Cp Hp
FliF
n.i.
Pemp
Hp
KeftKts
4a4dox
L-Arat
Hp
n.i.
FliF
Hp Cp Rr
Crr
Mmcp
Cstp
CpHk
Rr
Aaσrf
sinfreGR64gcs
Hp
Sk
Lr1e
ABCtsts

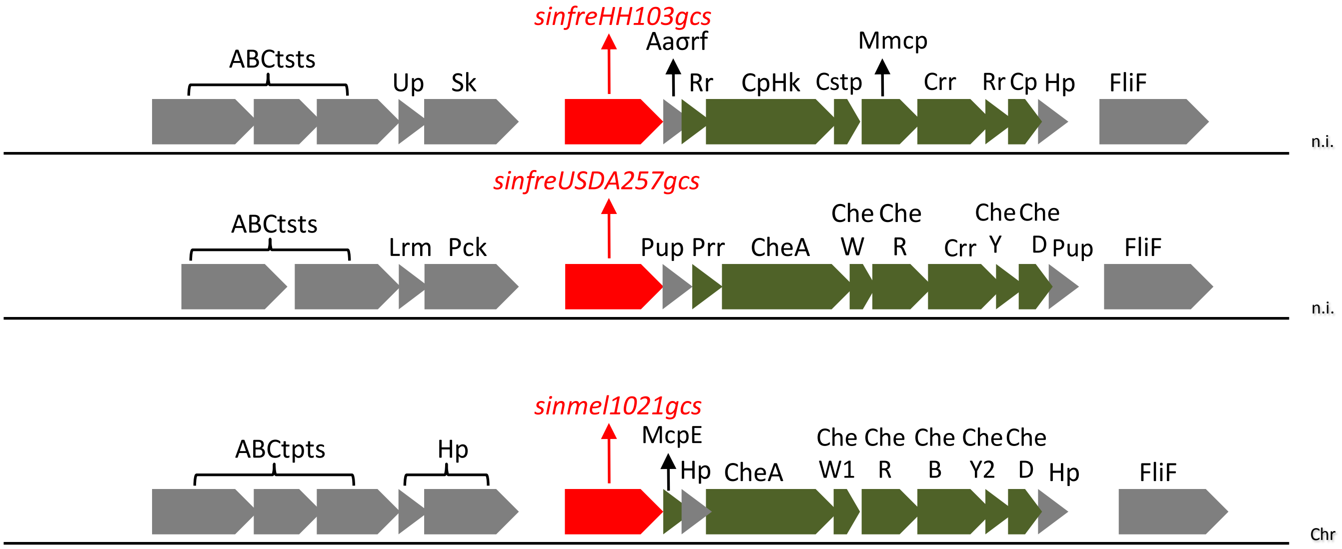
sinfreHH103gcs
ABCtsts
Up
Sk
Aaorf
Rr
CpHk
Cstp
Mmcp
Crr
Rr
Cp
Hp
FliF
n.i.
sinfreUSDA257gcs
ABCtsts
Lrm
Pck
Pup
Prr
CheA
Che
W
Che
R
Crr
Che
Y
Che
D
Pup
FliF
n.i.
sinmel1021gcs
ABCtpts
Hp
McpE
Hp
CheA
Che
W1
Che
R
Che
B
Che
Y2
Che
D
Hp
FliF
Chr

(D)

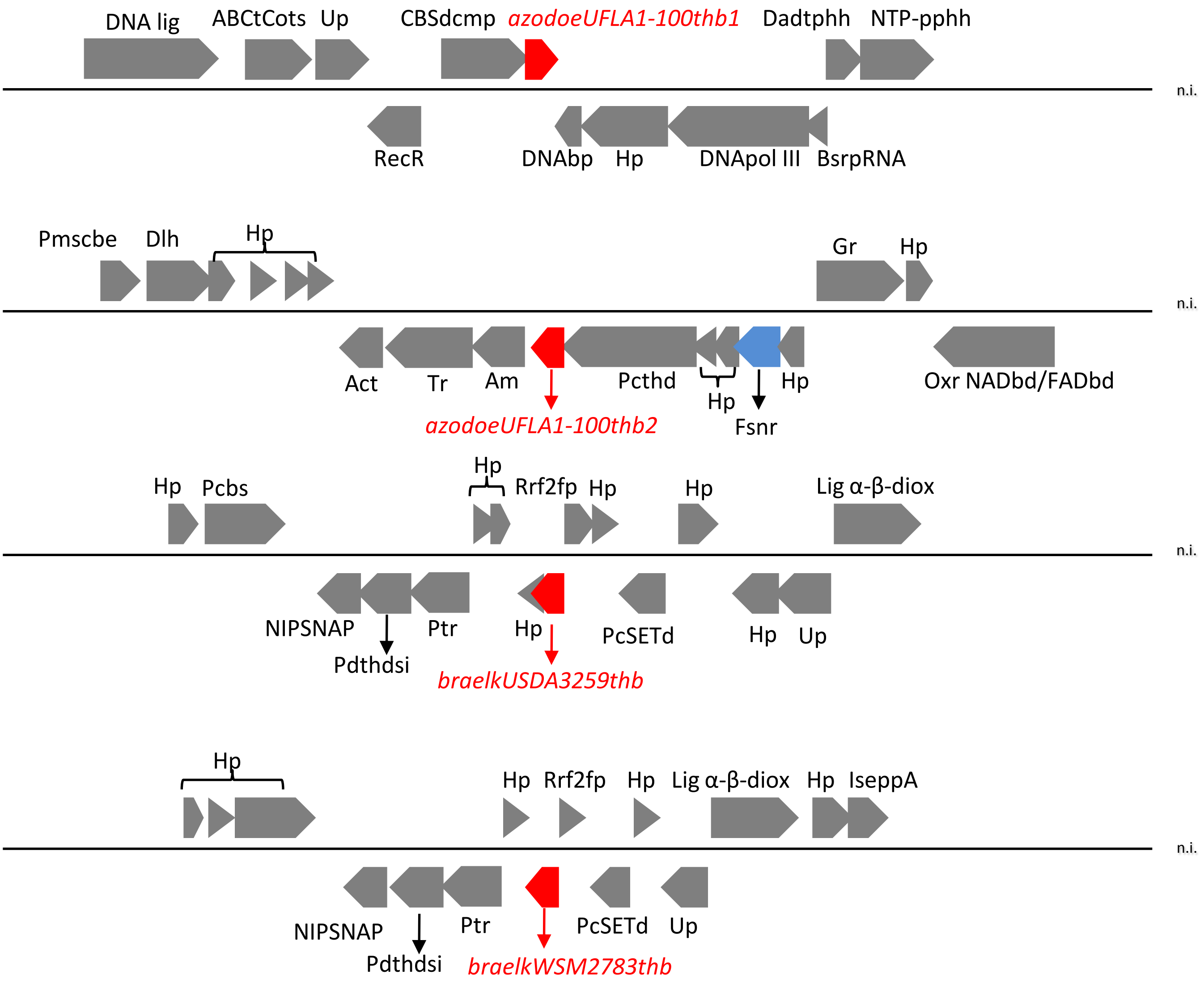
DNA lig
ABCtCots
Up
CBSdcmp
azodoeUFLA1-100thb1
Dadtphh
NTP-pphh
n.i.
RecR
DNAbp
Hp
DNApol III
BsrpRNA
Pmscbe
Dlh
Hp
Gr
Hp
n.i.
Act
Tr
Am
Pcthd
Hp
Hp
Fsnr
Oxr NADbd/FADbd
azodoeUFLA1-100thb2
Hp
Pcbs
Hp
Rrf2fp
Hp
Hp
Lig α-β-diox
n.i.
NIPSNAP
Pdthdsi
Ptr
Hp
PcSETd
Hp
Up
braelkUSDA3259thb
Hp
Hp
Rrf2fp
Hp
Lig α-β-diox
Hp
IseppA
n.i.
NIPSNAP
Pdthdsi
Ptr
PcSETd
Up
braelkWSM2783thb

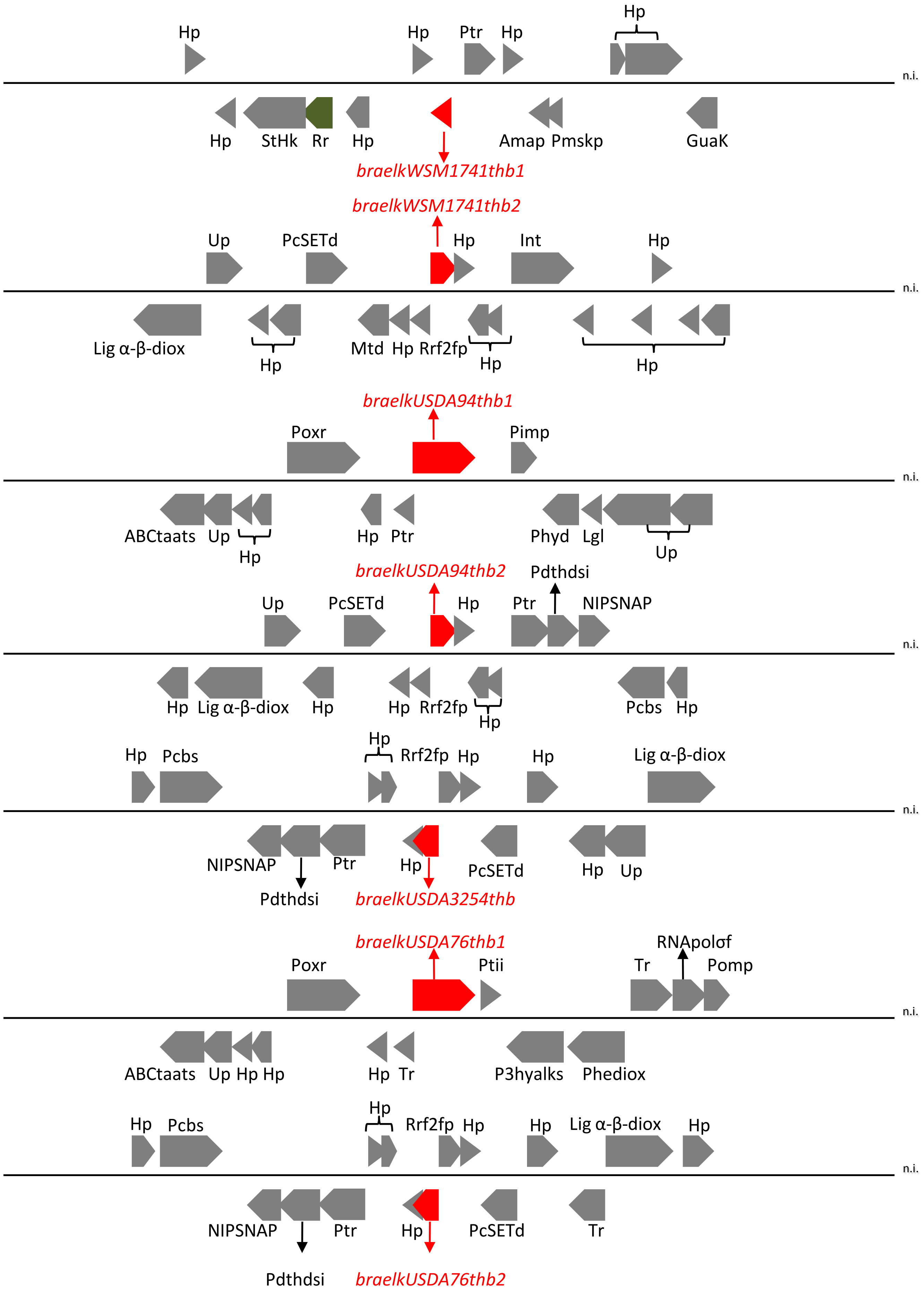
Hp
Hp
Ptr
Hp
Hp
n.i.
Hp
StHk
Rr
Hp
Amap
Pmskp
GuaK
braelkWSM1741thb1
braelkWSM1741thb2
Up
PcSETd
Hp
Int
Hp
n.i.
Lig α-β-diox
Hp
Mtd
Hp
Rrf2fp
Hp
Hp
braelkUSDA94thb1
Poxr
Pimp
n.i.
ABCtaats
Up
Hp
Hp
Ptr
Phyd
Lgl
Up
braelkUSDA94thb2
Pdthdsi
Up
PcSETd
Hp
Ptr
NIPSNAP
n.i.
Hp
Lig α-β-diox
Hp
Hp
Rrf2fp
Hp
Pcbs
Hp
Hp
Pcbs
Hp
Rrf2fp
Hp
Hp
Lig α-β-diox
n.i.
NIPSNAP
Ptr
Hp
PcSETd
Hp
Up
Pdthdsi
braelkUSDA3254thb
braelkUSDA76thb1
RNApolσf
Poxr
Ptii
Tr
Pomp
n.i.
ABCtaats
Up
Hp
Hp
Hp
Tr
P3hyalks
Phediox
Hp
Pcbs
Hp
Rrf2fp
Hp
Hp
Lig α-β-diox
Hp
n.i.
NIPSNAP
Ptr
Hp
PcSETd
Tr
Pdthdsi
braelkUSDA76thb2

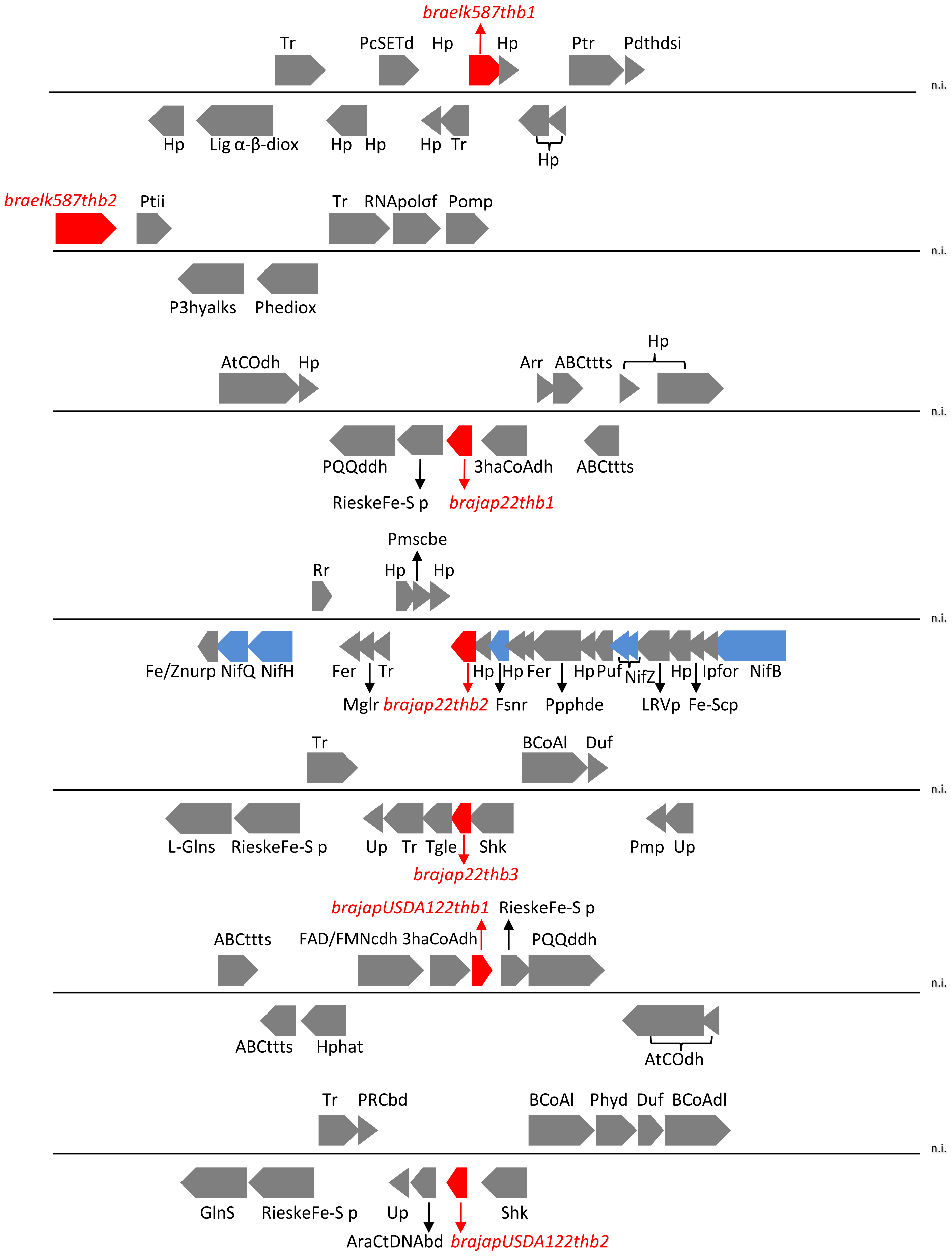
braelk587thb1
Tr
PcSETd
Hp
Hp
Ptr
Pdthdsi
n.i.
Hp
Lig α-β-diox
Hp
Hp
Hp
Tr
Hp
braelk587thb2
Ptii
Tr
RNApolσf
Pomp
n.i.
P3hyalks
Phediox
Hp
AtCOdh
Hp
Arr
ABCttts
n.i.
PQQddh
3haCoAdh
ABCttts
RieskeFe-S p
brajap22thb1
Pmscbe
Rr
Hp
Hp
n.i.
Fe/Znurp
NifQ
NifH
Fer
Tr
Hp
Hp
Fer
Hp
Puf
NifZ
Hp
Ipfor
NifB
Mglr
brajap22thb2
Fsnr
Ppphde
LRVp
Fe-Scp
Tr
BCoAl
Duf
n.i.
L-Glns
RieskeFe-S p
Up
Tr
Tgle
Shk
Pmp
Up
brajap22thb3
brajapUSDA122thb1
RieskeFe-S p
ABCttts
FAD/FMNcdh
3haCoAdh
PQQddh
n.i.
ABCttts
Hphat
AtCOdh
Tr
PRCbd
BCoAl
Phyd
Duf
BCoAdl
n.i.
GlnS
RieskeFe-S p
Up
Shk
AraCtDNAbd
brajapUSDA122thb2

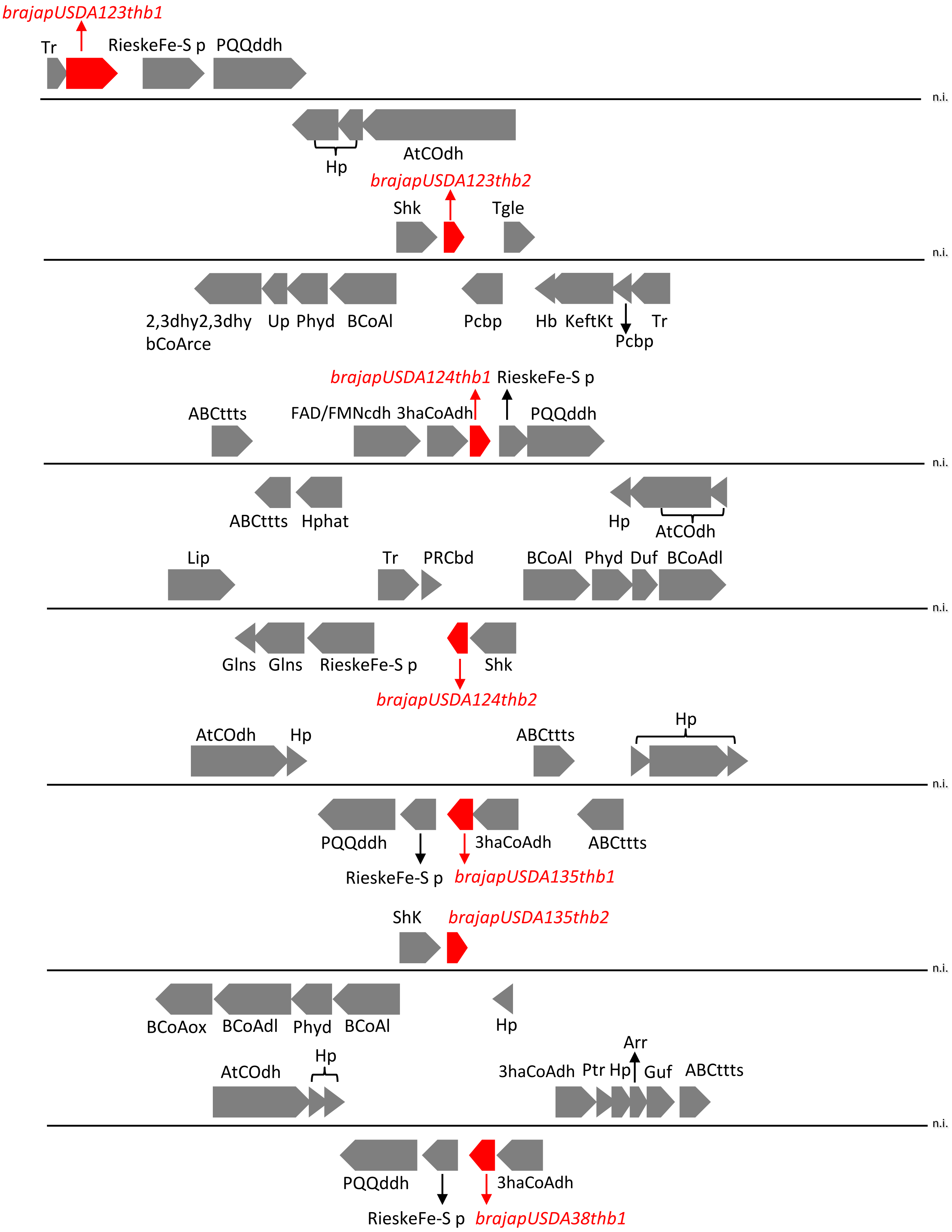
brajapUSDA123thb1
Tr
RieskeFe-S p
PQQddh
n.i.
Hp
AtCOdh
brajapUSDA123thb2
Shk
Tgle
n.i.
2,3dhy2,3dhy
bCoArce
Up
Phyd
BCoAl
Pcbp
Hb
KeftKt
Tr
Pcbp
brajapUSDA124thb1
RieskeFe-S p
ABCttts
FAD/FMNcdh
3haCoAdh
PQQddh
n.i.
ABCttts
Hphat
Hp
AtCOdh
Lip
Tr
PRCbd
BCoAl
Phyd
Duf
BCoAdl
n.i.
Glns
Glns
RieskeFe-S p
Shk
brajapUSDA124thb2
AtCOdh
Hp
ABCttts
Hp
n.i.
PQQddh
3haCoAdh
ABCttts
RieskeFe-S p
brajapUSDA135thb1
ShK
brajapUSDA135thb2
n.i.
BCoAox
BCoAdl
Phyd
BCoAl
Hp
AtCOdh
Hp
Arr
3haCoAdh
Ptr
Hp
Guf
ABCttts
n.i.
PQQddh
3haCoAdh
RieskeFe-S p
brajapUSDA38thb1

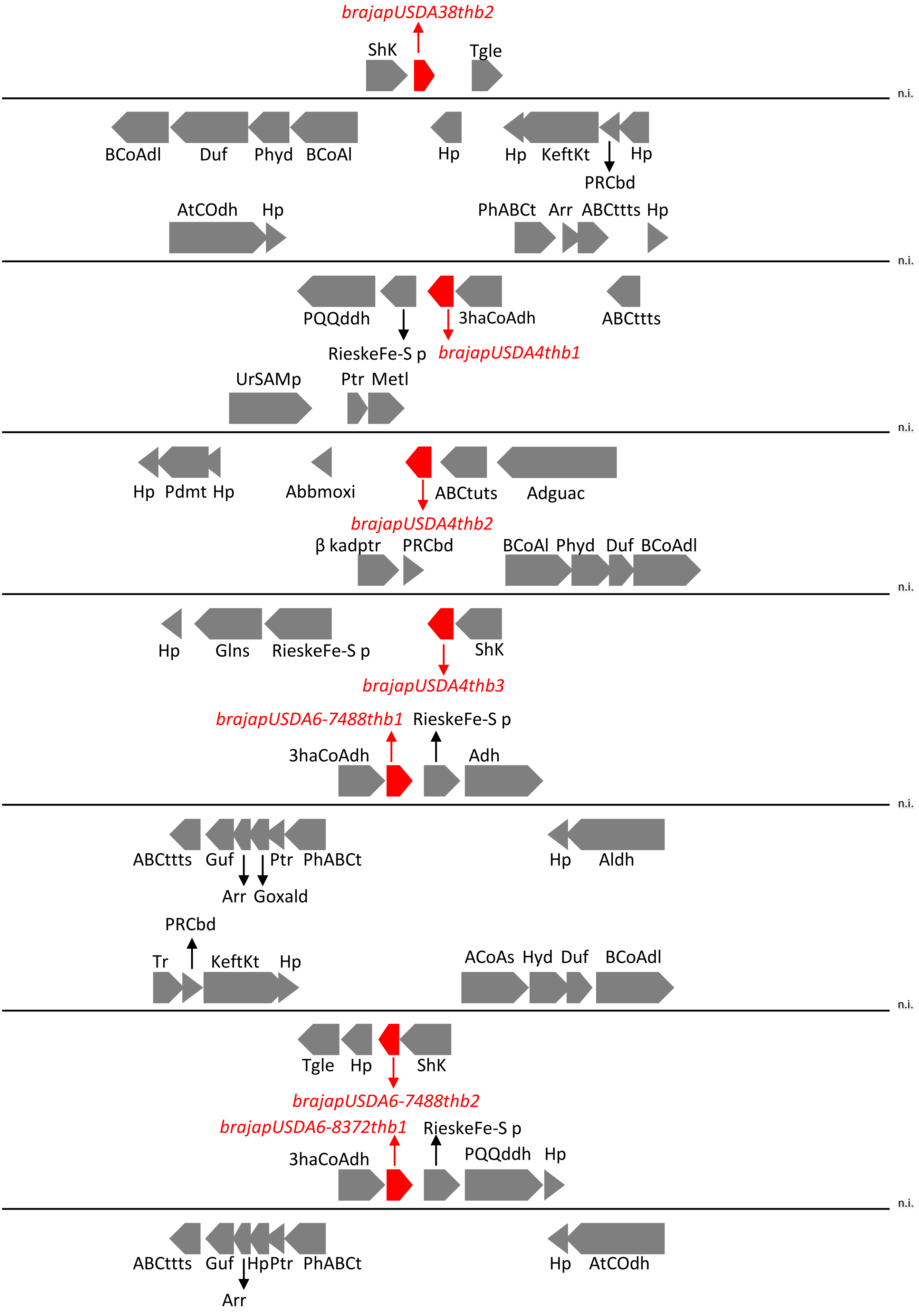
brajapUSDA38thb2
ShK
Tgle
n.i.
BCoAdl
Duf
Phyd
BCoAl
Hp
Hp
KeftKt
Hp
PRCbd
AtCOdh
Hp
PhABCt
Arr
ABCttts
Hp
n.i.
PQQddh
3haCoAdh
ABCttts
RieskeFe-S p
brajapUSDA4thb1
UrSAMp
Ptr
Metl
n.i.
Hp
Pdmt
Hp
Abbmoxi
ABCtuts
Adguac
brajapUSDA4thb2
β kadptr
PRCbd
BCoAl
Phyd
Duf
BCoAdl
n.i.
Hp
Glns
RieskeFe-S p
ShK
brajapUSDA4thb3
brajapUSDA6-7488thb1
RieskeFe-S p
3haCoAdh
Adh
n.i.
ABCttts
Guf
Ptr
PhABCt
Hp
Aldh
Arr
Goxald
PRCbd
Tr
KeftKt
Hp
ACoAs
Hyd
Duf
BCoAdl
n.i.
Tgle
Hp
ShK
brajapUSDA6-7488thb2
brajapUSDA6-8372thb1
RieskeFe-S p
3haCoAdh
PQQddh
Hp
n.i.
ABCttts
Guf
Hp
Ptr
PhABCt
Hp
AtCOdh
Arr

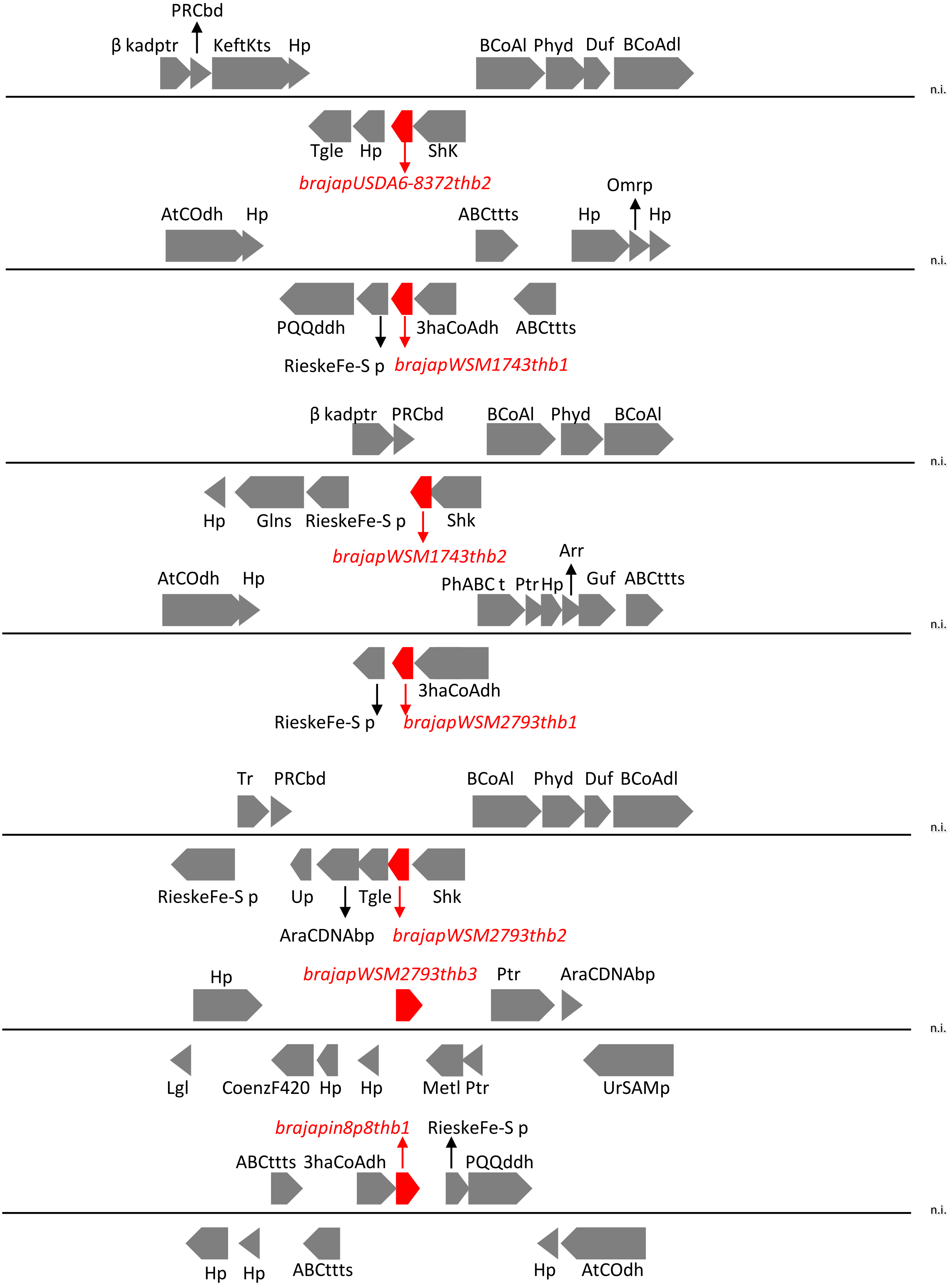
PRCbd
β kadptr
KeftKts
Hp
BCoAl
Phyd
Duf
BCoAdl
n.i.
Tgle
Hp
ShK
brajapUSDA6-8372thb2
Omrp
AtCOdh
Hp
ABCttts
Hp
Hp
n.i.
PQQddh
3haCoAdh
ABCttts
RieskeFe-S p
brajapWSM1743thb1
β kadptr
PRCbd
BCoAl
Phyd
BCoAl
n.i.
Hp
Glns
RieskeFe-S p
Shk
brajapWSM1743thb2
Arr
AtCOdh
Hp
PhABC t
Ptr
Hp
Guf
ABCttts
n.i.
3haCoAdh
RieskeFe-S p
brajapWSM2793thb1
Tr
PRCbd
BCoAl
Phyd
Duf
BCoAdl
n.i.
RieskeFe-S p
Up
Tgle
Shk
AraCDNAbp
brajapWSM2793thb2
Hp
brajapWSM2793thb3
Ptr
AraCDNAbp
n.i.
Lgl
CoenzF420
Hp
Hp
Metl
Ptr
UrSAMp
brajapin8p8thb1
RieskeFe-S p
ABCttts
3haCoAdh
PQQddh
n.i.
Hp
Hp
ABCttts
Hp
AtCOdh

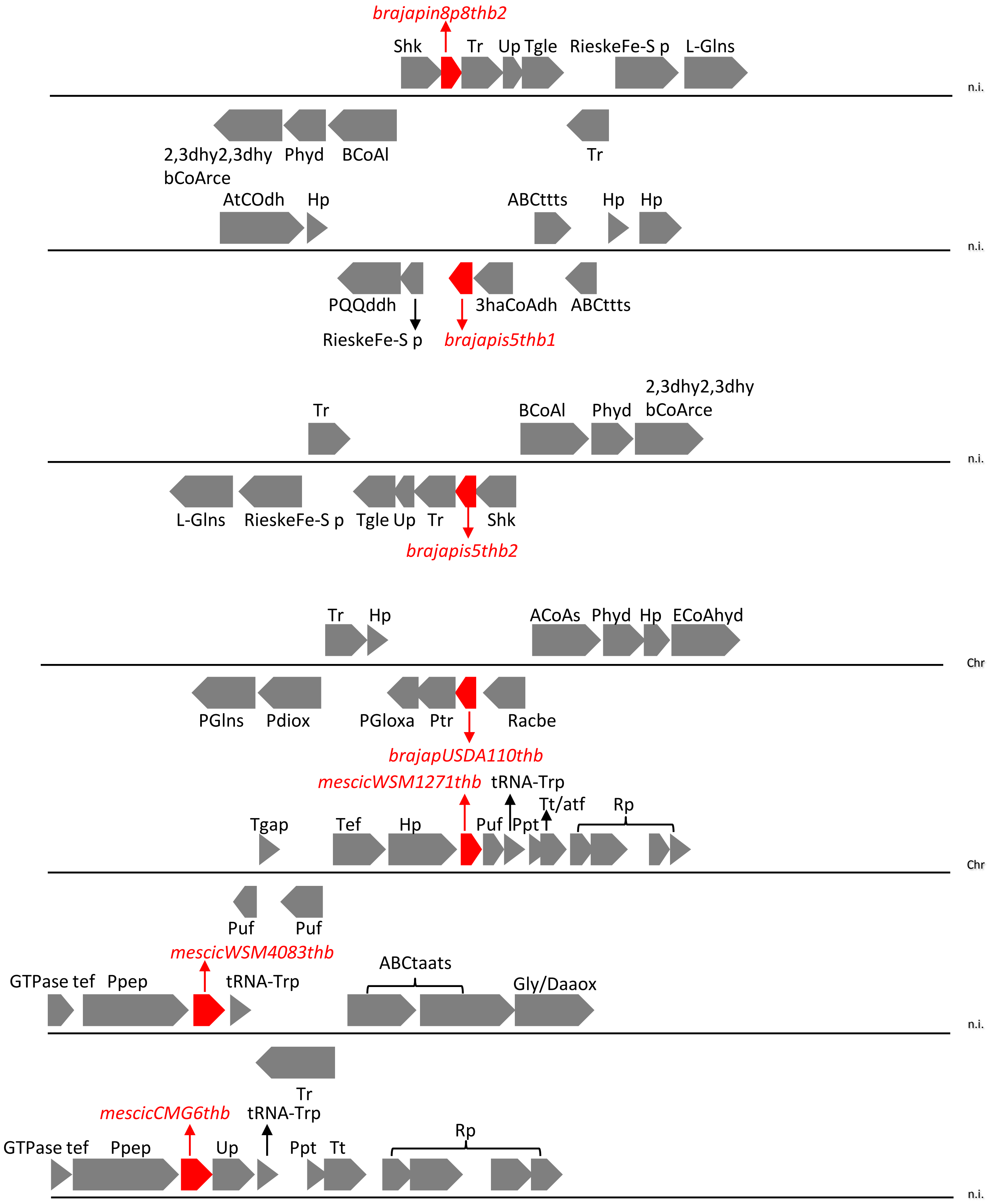
brajapin8p8thb2
Shk
Tr
Up
Tgle
RieskeFe-S p
L-Glns
n.i.
2,3dhy2,3dhy bCoArce
Phyd
BCoAl
Tr
AtCOdh
Hp
ABCttts
Hp
Hp
n.i.
PQQddh
RieskeFe-S p
brajapis5thb1
3haCoAdh
ABCttts
2,3dhy2,3dhy bCoArce
Tr
BCoAl
Phyd
n.i.
L-Glns
RieskeFe-S p
Tgle
Up
Tr
Shk
brajapis5thb2
Tr
Hp
ACoAs
Phyd
Hp
ECoAhyd
Chr
PGlns
Pdiox
PGloxa
Ptr
Racbe
brajapUSDA110thb
mescicWSM1271thb
tRNA-Trp
Tt/atf
Rp
Tgap
Tef
Hp
Puf
Ppt
Chr
Puf
Puf
mescicWSM4083thb
GTPase tef
Ppep
tRNA-Trp
ABCtaats
Gly/Daaox
n.i.
Tr
mescicCMG6thb
tRNA-Trp
Rp
GTPase tef
Ppep
Up
Ppt
Tt
n.i.

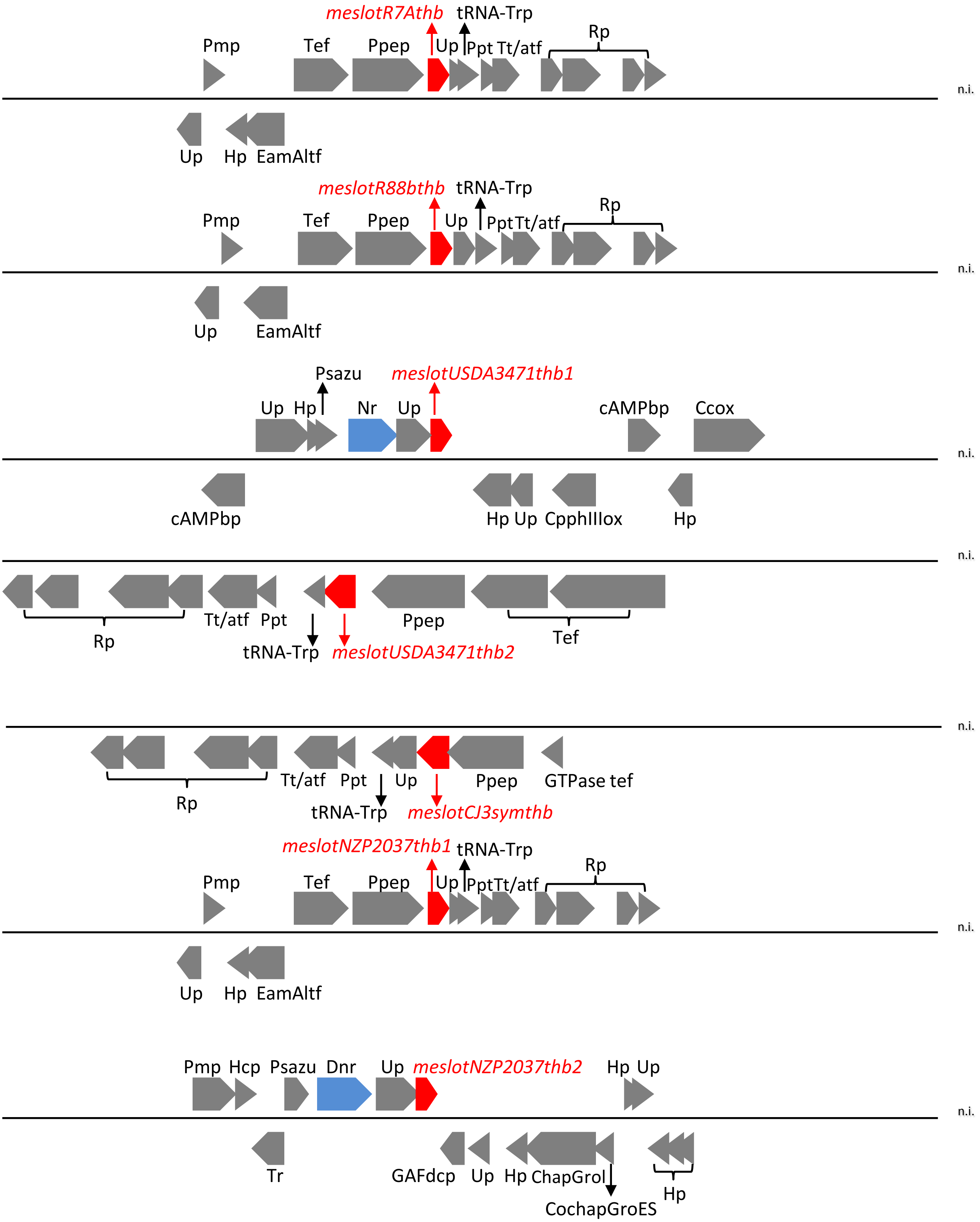

meslotR7Athb
tRNA-Trp
Pmp
Tef
Ppep
Up
Ppt
Tt/atf
Rp
n.i.
Up
Hp
EamAltf
meslotR88bthb
tRNA-Trp
Pmp
Tef
Ppep
Up
Ppt
Tt/atf
Rp
n.i.
Up
EamAltf
Psazu
meslotUSDA3471thb1
Up
Hp
Nr
Up
cAMPbp
Ccox
n.i.
cAMPbp
Hp
Up
CpphIIIox
Hp
n.i.
Rp
Tt/atf
Ppt
tRNA-Trp
meslotUSDA3471thb2
Ppep
Tef
n.i.
Rp
Tt/atf
Ppt
Up
tRNA-Trp
meslotCJ3symthb
Ppep
GTPase tef
meslotNZP2037thb1
tRNA-Trp
Pmp
Tef
Ppep
Up
Ppt
Tt/atf
Rp
n.i.
Up
Hp
EamAltf
Pmp
Hcp
Psazu
Dnr
Up
meslotNZP2037thb2
Hp
Up
n.i.
Tr
GAFdcp
Up
Hp
ChapGroI
CochapGroES
Hp

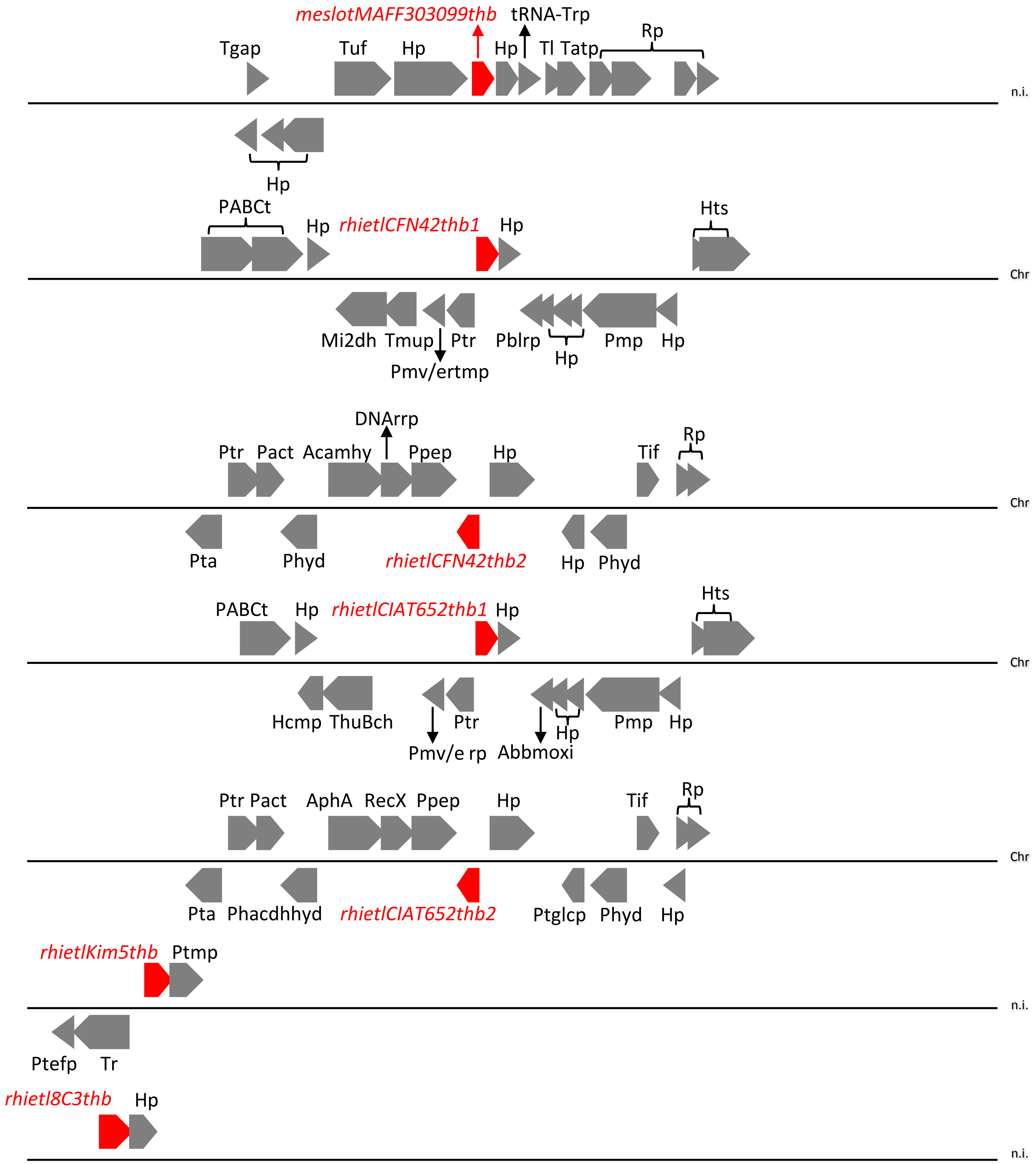

meslotMAFF303099thb
tRNA-Trp
Tgap
Tuf
Hp
Hp
Tl
Tatp
Rp
n.i.
Hp
PABCt
Hp
rhietlCFN42thb1
Hp
Hts
Chr
Mi2dh
Tmup
Ptr
Pblrp
Hp
Pmp
Hp
Pmv/ertmp
DNArrp
Ptr
Pact
Acamhy
Ppep
Hp
Tif
Rp
Chr
Pta
Phyd
rhietlCFN42thb2
Hp
Phyd
PABCt
Hp
rhietlCIAT652thb1
Hp
Hts
Chr
Hcmp
ThuBch
Ptr
Hp
Pmp
Hp
Pmv/e rp
Abbmoxi
Ptr Pact
AphA
RecX
Ppep
Hp
Tif
Rp
Chr
Pta
Phacdhhyd
rhietlCIAT652thb2
Ptglcp
Phyd
Hp
rhietlKim5thb
Ptmp
n.i.
Ptefp
Tr
rhietl8C3thb
Hp
n.i.

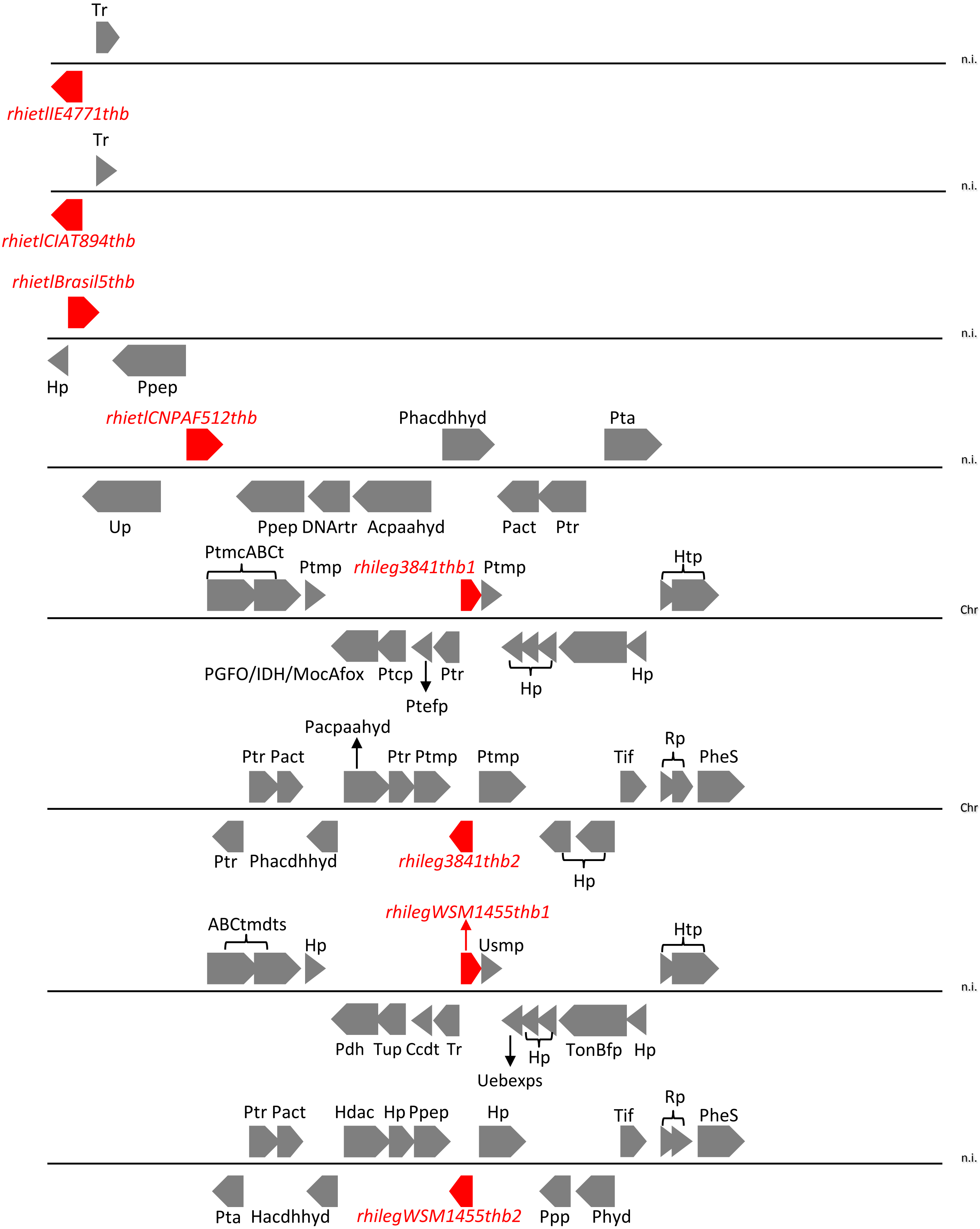

Tr
n.i.
rhietlIE4771thb
Tr
n.i.
rhietlCIAT894thb
rhietlBrasil5thb
n.i.
Hp
Ppep
rhietlCNPAF512thb
Phacdhhyd
Pta
n.i.
Up
Ppep DNArtr Acpaahyd
Pact Ptr
PtmcABCt
Ptmp
rhileg3841thb1
Ptmp
Htp
Chr
PGFO/IDH/MocAfox Ptcp Ptr
Hp
Hp
Ptefp
Pacpaahyd
Ptr Pact
Ptr Ptmp
Ptmp
Tif
Rp
PheS
Chr
Ptr Phacdhhyd
rhileg3841thb2
Hp
rhilegWSM1455thb1
ABCtmdts
Hp
Usmp
Htp
n.i.
Pdh Tup Ccdt Tr
Hp
TonBfp Hp
Uebexps
Ptr Pact
Hdac Hp Ppep
Hp
Tif
Rp
PheS
n.i.
Pta Hacdhhyd
rhilegWSM1455thb2
Ppp
Phyd

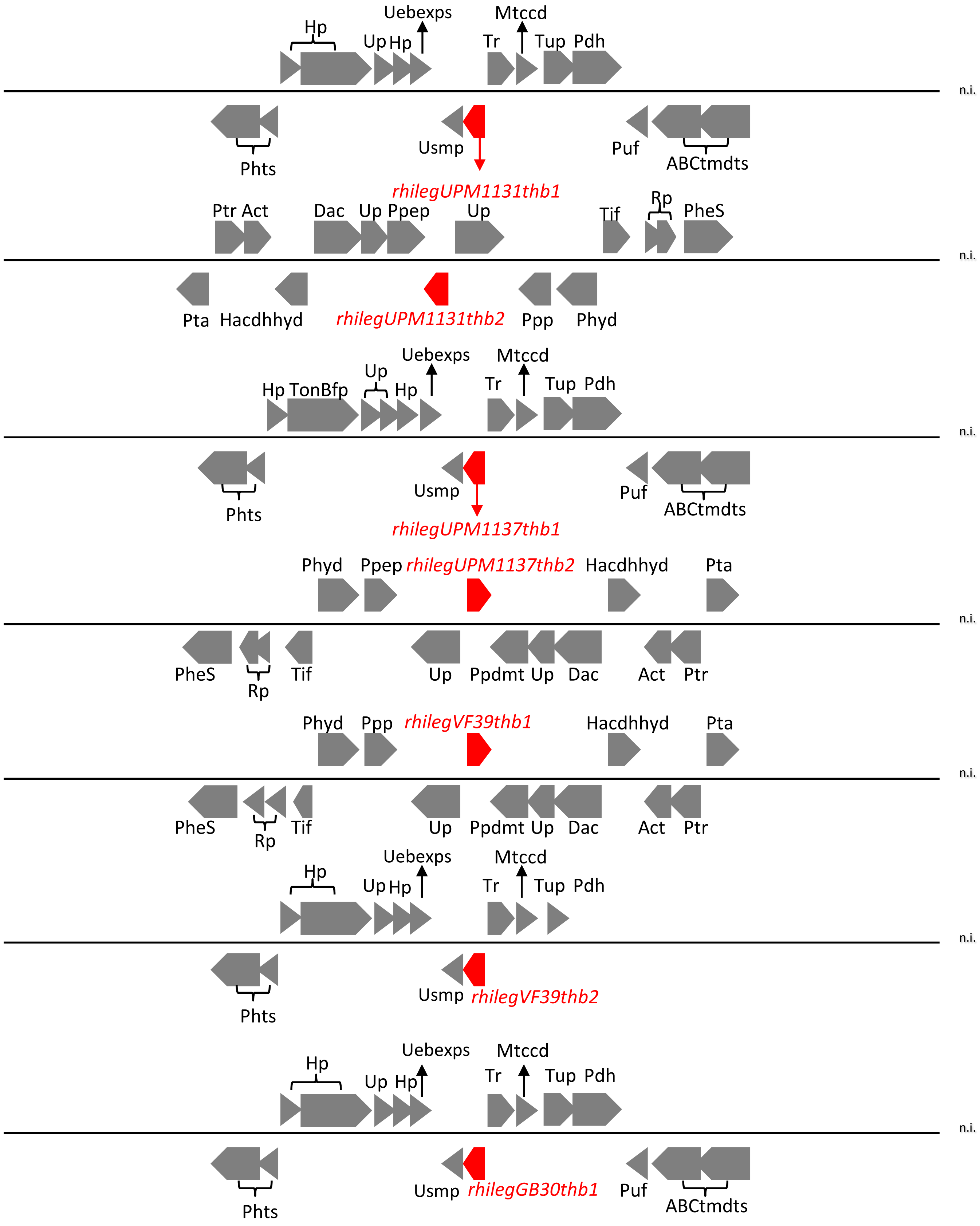
Hp
Uebexps
Up Hp
Mtccd
Tr
Tup Pdh
n.i.
Phts
Usmp
Puf
ABCtmdts
rhilegUPM1131thb1
Ptr Act
Dac Up Ppep
Up
Tif
Rp
PheS
n.i.
Pta Hacdhhyd
rhilegUPM1131thb2
Ppp Phyd
Uebexps
Mtccd
Up
Hp TonBfp
Hp
Tr
Tup Pdh
n.i.
Phts
Usmp
Puf
ABCtmdts
rhilegUPM1137thb1
Phyd Ppep rhilegUPM1137thb2 Hacdhhyd Pta
n.i.
PheS
Rp
Tif
Up Ppdmt Up Dac
Act Ptr
Phyd Ppp rhilegVF39thb1 Hacdhhyd Pta
n.i.
PheS
Rp
Tif
Up Ppdmt Up Dac
Act Ptr
Uebexps
Mtccd
Hp
Up Hp
Tr
Tup Pdh
n.i.
Phts
Usmp rhilegVF39thb2
Uebexps
Mtccd
Hp
Up Hp
Tr
Tup Pdh
n.i.
Phts
Usmp rhilegGB30thb1
Puf
ABCtmdts

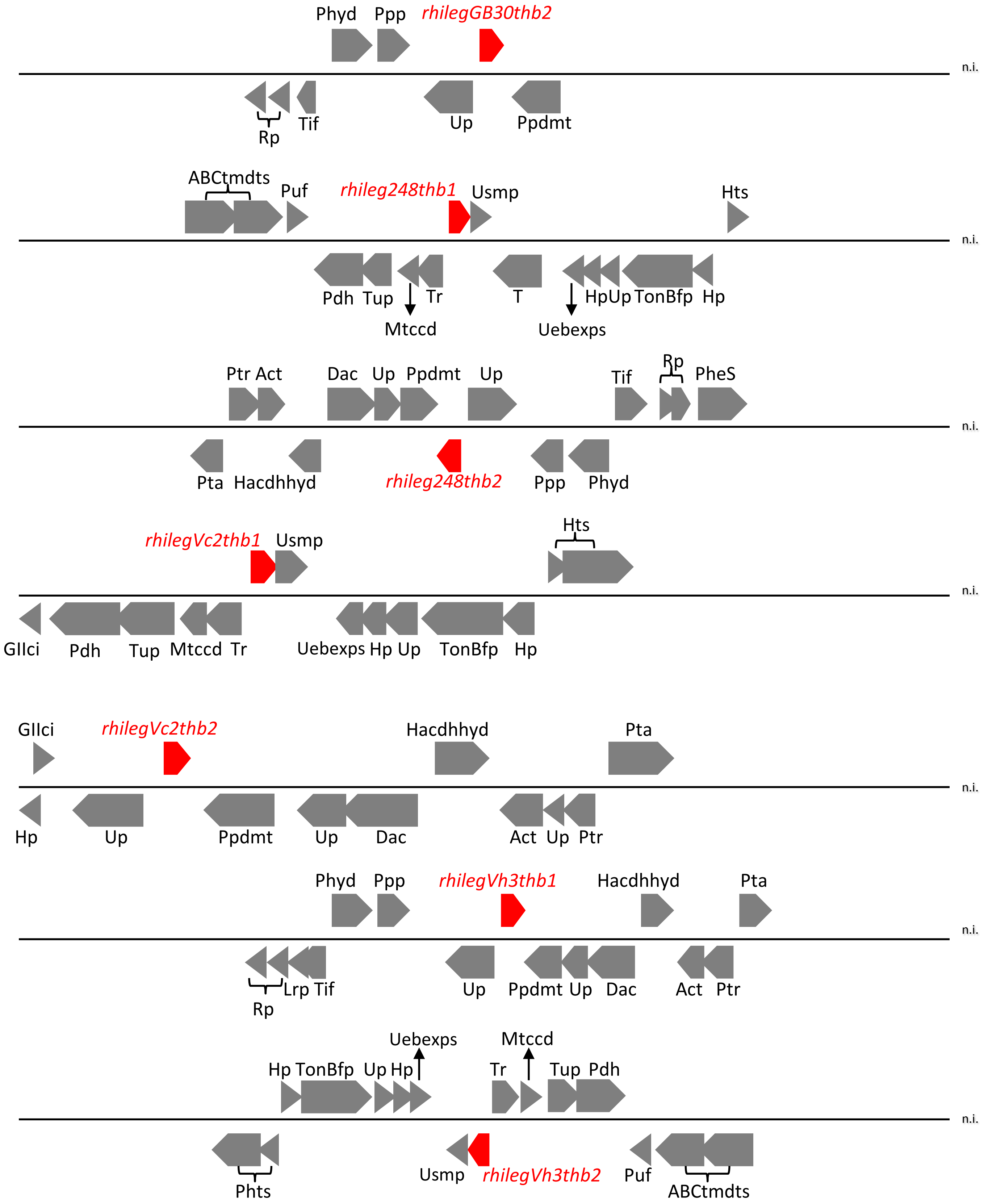
Phyd Ppp rhilegGB30thb2
n.i.
Rp Tif Up Ppdmt
ABCtmdts Puf rhileg248thb1 Usmp Hts
n.i.
Pdh Tup Tr T HpUp TonBfp Hp
Mtccd Uebexps
Ptr Act Dac Up Ppdmt Up Tif Rp PheS
n.i.
Pta Hacdhhyd rhileg248thb2 Ppp Phyd
rhilegVc2thb1 Usmp Hts
n.i.
GIIci Pdh Tup Mtccd Tr Uebexps Hp Up TonBfp Hp
GIIci rhilegVc2thb2 Hacdhhyd Pta
n.i.
Hp Up Ppdmt Up Dac Act Up Ptr
Phyd Ppp rhilegVh3thb1 Hacdhhyd Pta
n.i.
Lrp Tif Up Ppdmt Up Dac Act Ptr
Rp
Uebexps Mtccd
Hp TonBfp Up Hp Tr Tup Pdh
n.i.
Phts Usmp rhilegVh3thb2 Puf ABCtmdts

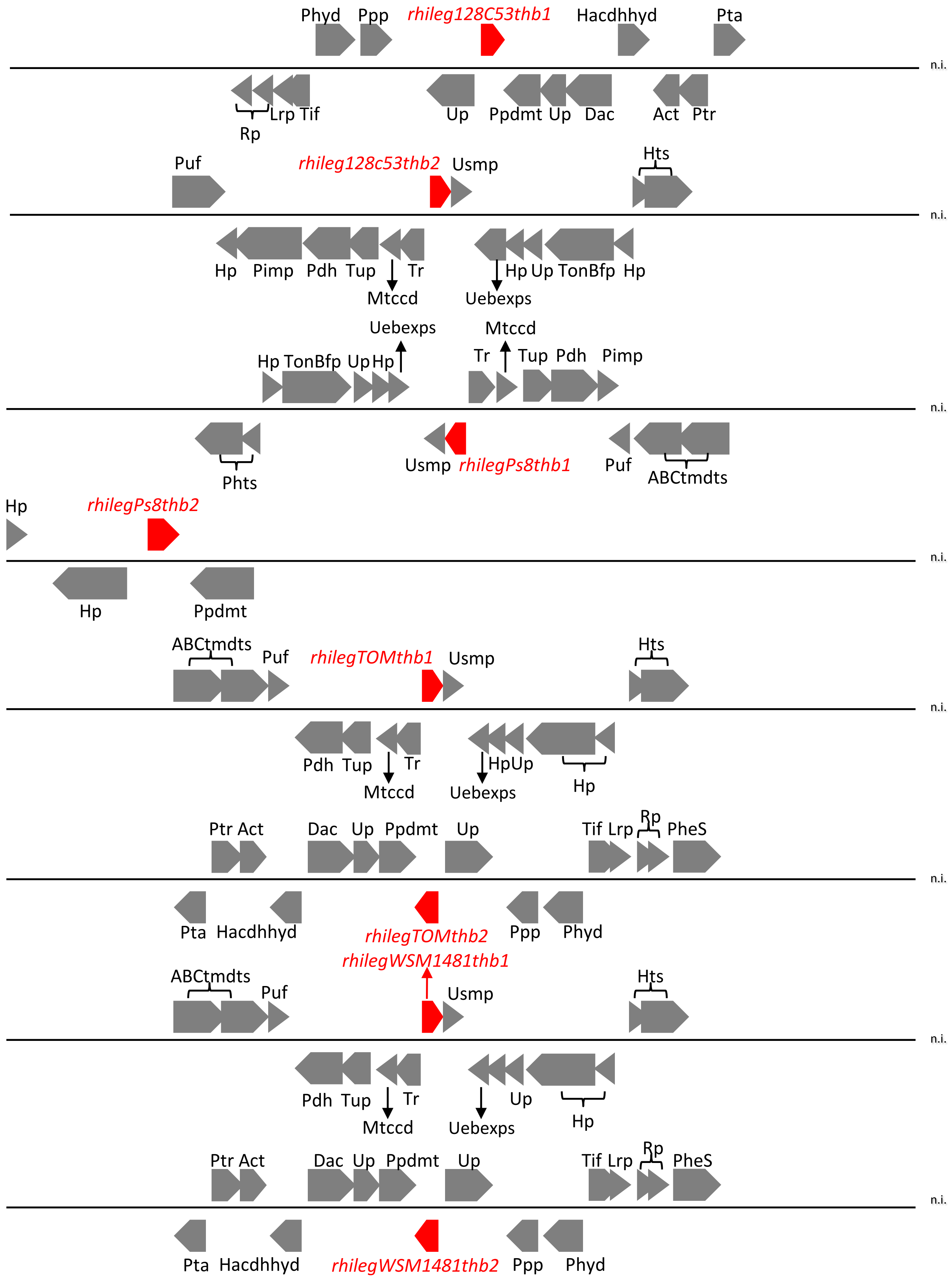
Phyd Ppp rhileg128C53thb1 Hacdhhyd Pta
n.i.
Rp Lrp Tif Up Ppdmt Up Dac Act Ptr
Puf rhileg128c53thb2 Usmp Hts
n.i.
Hp Pimp Pdh Tup Tr Mtccd Hp Up TonBfp Hp Uebexps
Uebexps Mtccd
Hp TonBfp Up Hp Tr Tup Pdh Pimp
n.i.
Phts Usmp rhilegPs8thb1 Puf ABCtmdts
Hp rhilegPs8thb2
n.i.
Hp Ppdmt
ABCtmdts Puf rhilegTOMthb1 Usmp Hts
n.i.
Pdh Tup Tr Mtccd HpUp Uebexps Hp
Ptr Act Dac Up Ppdmt Up Tif Lrp Rp PheS
n.i.
Pta Hacdhhyd rhilegTOMthb2 Ppp Phyd
rhilegWSM1481thb1
ABCtmdts Puf Usmp Hts
n.i.
Pdh Tup Tr Mtccd Up Uebexps Hp
Ptr Act Dac Up Ppdmt Up Tif Lrp Rp PheS
n.i.
Pta Hacdhhyd rhilegWSM1481thb2 Ppp Phyd

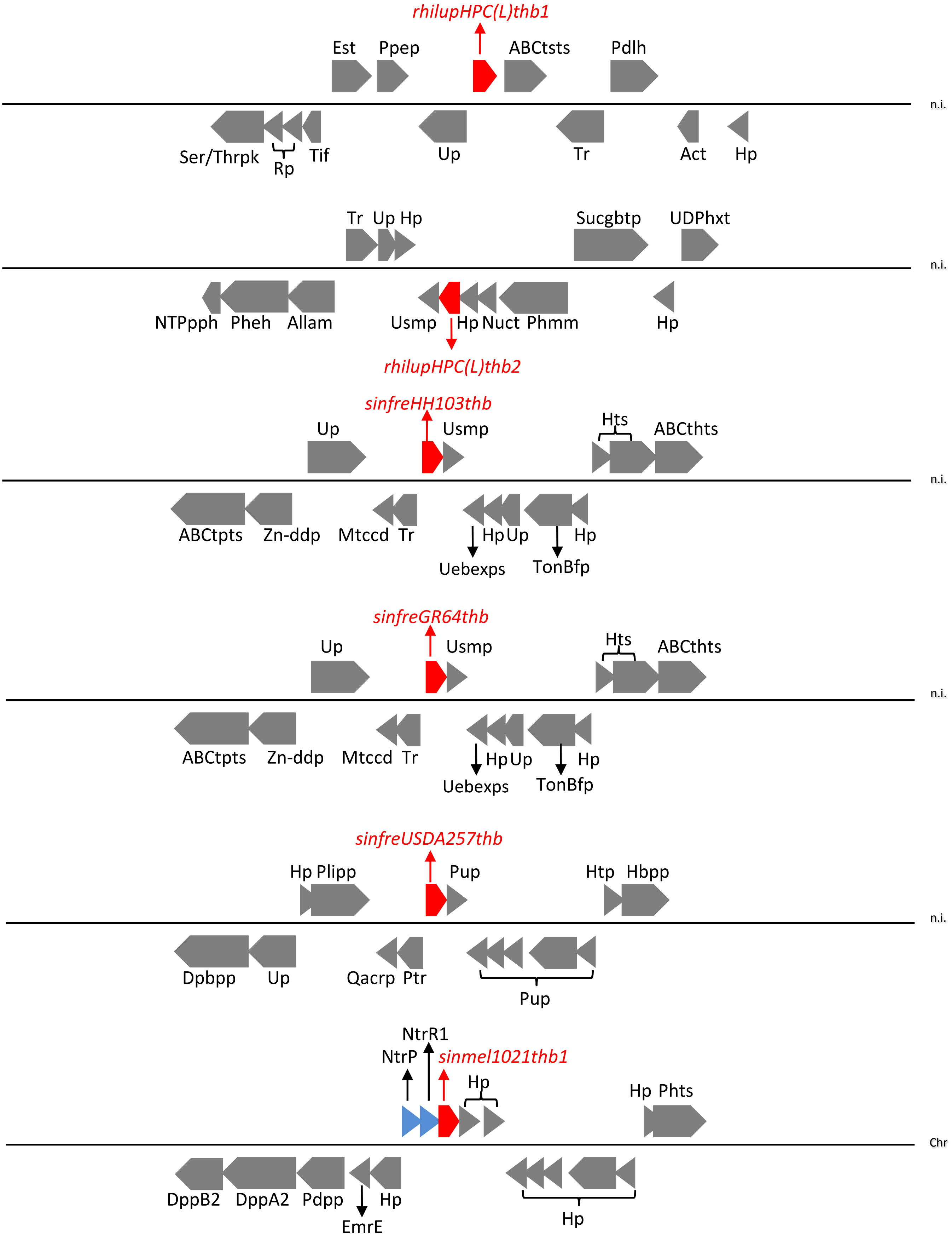

rhilupHPC(L)thb1
Est
Ppep
ABCtsts
Pdlh
n.i.
Ser/Thrpk
Rp
Tif
Up
Tr
Act
Hp
Tr
Up
Hp
Sucgbtp
UDPhxt
n.i.
NTPpph
Pheh
Allam
Usmp
Hp
Nuct
Phmm
Hp
rhilupHPC(L)thb2
sinfreHH103thb
Up
Usmp
Hts
ABCthts
n.i.
ABCtpts
Zn-ddp
Mtccd
Tr
Hp
Up
Hp
Uebexps
TonBfp
sinfreGR64thb
Up
Usmp
Hts
ABCthts
n.i.
ABCtpts
Zn-ddp
Mtccd
Tr
Hp
Up
Hp
Uebexps
TonBfp
sinfreUSDA257thb
Hp
Plipp
Pup
Htp
Hbpp
n.i.
Dpbpp
Up
Qacrp
Ptr
Pup
NtrR1
NtrP
sinmel1021thb1
Hp
Hp
Phts
Chr
DppB2
DppA2
Pdpp
Hp
EmrE
Hp

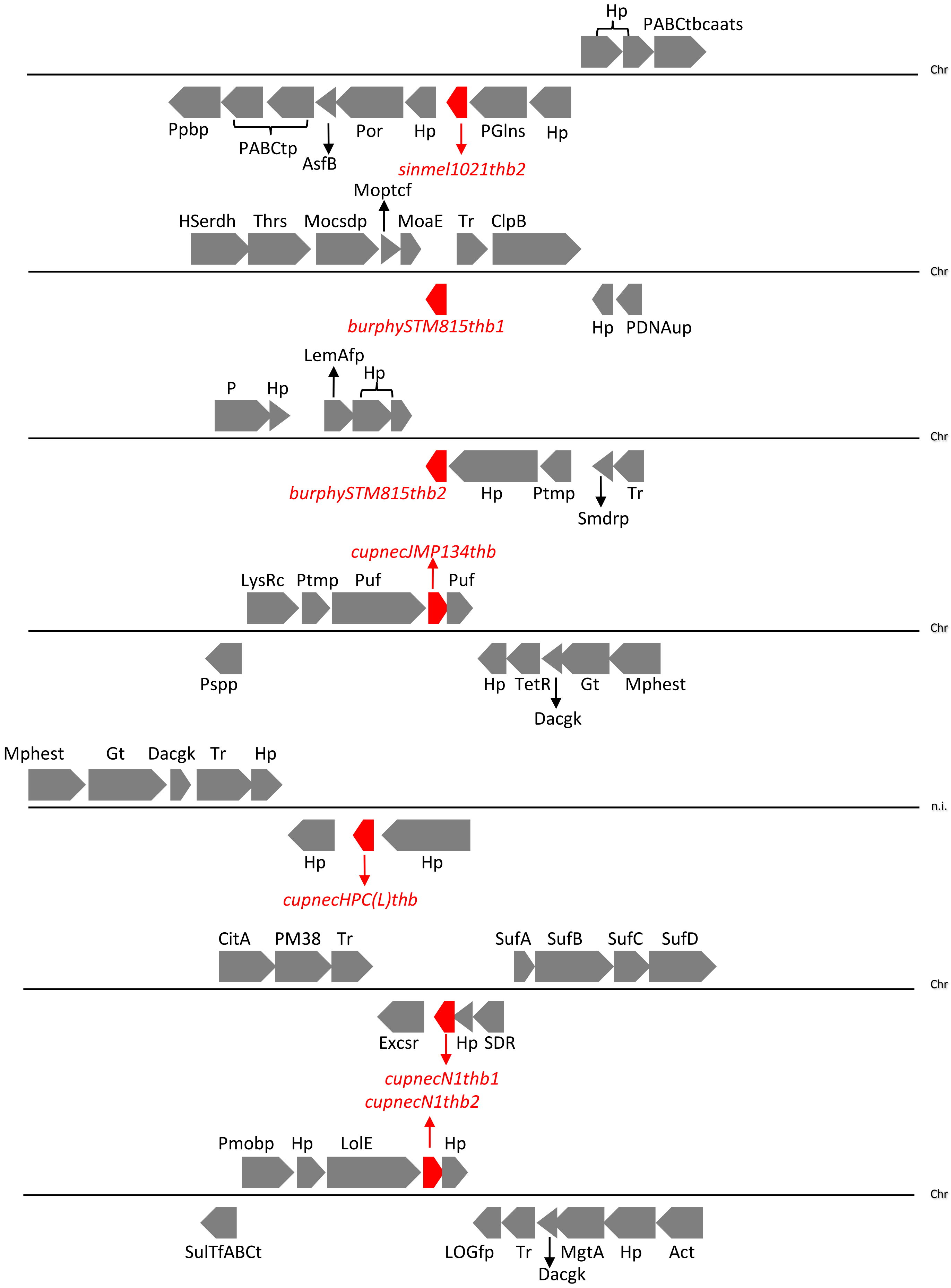

Hp
PABCtbcaats
Chr
Ppbp
PABCtp
AsfB
Por
Hp
PGlns
Hp
sinmel1021thb2
Moptcf
HSerdh
Thrs
Mocsdp
MoaE
Tr
ClpB
Chr
burphySTM815thb1
Hp
PDNAup
LemAfp
Hp
P
Hp
Chr
burphySTM815thb2
Hp
Ptmp
Smdrp
Tr
cupnecJMP134thb
LysRc
Ptmp
Puf
Puf
Chr
Pspp
Hp
TetR
Dacgk
Gt
Mphest
Mphest
Gt
Dacgk
Tr
Hp
n.i.
Hp
Hp
cupnecHPC(L)thb
CitA
PM38
Tr
SufA
SufB
SufC
SufD
Chr
Excsr
Hp
SDR
cupnecN1thb1
cupnecN1thb2
Pmobp
Hp
LolE
Hp
Chr
SulTfABCt
LOGfp
Tr
Dacgk
MgtA
Hp
Act

Abbreviations for predicted polypeptides:

2,3dhy2,3dhybeCoArce, 2,3 dihydro 2,3 dihydrobenzoyl CoA ring cleavage enzyme; **2dhp2r**, 2 dehydropantoate 2 reductase; **3haCoAdh**, 3 hydroxyacyl CoA dehydrogenase; **3imdh**, 3-isopropylmalate dehydrogenase; **4-a4-dox-L-arat**, 4-amino 4-deoxy-L-arabinose transferase; **Aaσrf**, Anti anti σ regulatory factor; **Abbmoxi**, Antibiotic biosynthesis monooxygenase; **ABCtaats**, ABC type amino acid transport system; **ABCtbcaats**, ABC type branch chain amino acid transport system; **ABCtCots**, ABC type Co transport system; **ABCthts**, ABC type hemin transport system; **ABCtmdts**, ABC type multidrug transport system; **ABCtnts**, ABC type nitrate transport system; **ABCtpts**, ABC type peptide transport system; **ABCtsts**, ABC, type sugar transport system; **ABCtts**, ABC type transport system; **ABCttts**, ABC type tungstate transport system; **ABCtuts**, ABC type uncharacterized transport system; **Acamhyd**, acetyl aminohydrolase; **ACoAdh,** acylCoA dehydrogenase; **ACoAs**, AcetylCoA synthetase; **Acpaahyd**, acetylpolyamine aminohydrolase; **Act**, acetyl transferase; **Adguac**, adenylate and guanylate cyclase; **Adh**, alcohol dehydrogenase; **Adsucl,** adenylosuccinate lyase; **Aep,** arabinose efflux permease; **Akr**, aldo/keto reductase; **Aldh**, aldehyde dehydrogenase; **Allam**, allantoate amidohydrolase; **Am**, amidase; **Amap**, Addition module antidote protein; **Amh,** amidohydrolase; **Anr**, Assimilatory nitrate reductase; **AphA**, acetylpolyamine aminohydrolase protein; **AraCtDNAbp**, AraC type DNA binding protein; **Arr**, Arsenate reductase; **AsfB**, putative ferredoxin protein; **Asp/Gluat**, Asp/Glu amidotransferase; **AtCOdh**, aerobic type carbon monoxide dehydrogenase; **ATPase chrp**, ATPases involved in chromosome partitioning; **BCoAdl**, benzoyl CoA dihydrodiol lyase ; **BCoAl**, benzoate CoA ligase; **BCoAox**, benzoyl CoA oxygenase, **βkadptr**, β ketoadipate pathway transcriptional regulator; **βlac**, β lactamase; **BNR/Aspbr**, BNR Asp box repeat; **BNRrld**, BNR repeat like domain; **BsrpRNA**, bacterial signal recognition particle RNA; **Cabefh**, Ca binding ef-hand; **cAMPbp**, cAmp-binding protein; **Cas**, Ca sensor; **CBS-dcmp**, CBS-domain containing membrane protein; **Ccdt**, cation/cationic drug transporter; **Ccox,** cytochrome oxidase; **Cd**, catechol 1,2-dioxygenase; **ChapGroI**, chaperonin GroI; **CheA**, chemotaxis two component sensor histidine kinase protein; **CheBch1**, protein glutamate methyltransferase; **CheD**, chemotaxis protein; **CheRch1**, chemotaxis methyltransferase protein; **CheW1**, chemotaxis signal transduction protein; **CheY2**, chemotaxis two component response regulator protein; **CitA**, citrate proton symporter; **ClpB**, ATP dependent chaperone; **CochapGroES**, co-chaperonin GroEs; **CoenzF420**, coenzyme F420; **Cp**, chemotaxis protein; **CpHk**, chemotaxis protein histidine kinase; **CpphIIIox,** coproporphyrinogen III oxidase; **Crr**, Chemotaxis response regulator; **Cstp**, chemotaxis signal transduction protein; **D2nd**, dioxygenase related to 2-nitropropane dioxygenase; **Dac**, deacetylase; **Dacgk**, diacylglycerol kinase; **Dadtphh**, diadenosine tetraphosphate hydrolase; **DapD**, N-succinyltransferase protein; **DapE**, putative succinyl diaminopimelate desuccinylase protein; **DapE2**, succinyl diaminopimelate desuccinylase protein; **Def1**, Peptide deformylase protein; **Dh,** dehydrogenase; **Dlh**, dienelactone hydrolase; **DMT,** DMT (drug/metabolite transporter) superfamily permease; **DNAbp,** DNA binding protein, **DNAlig**, DNA ligase; **DNApolIII**, DNA polymerase III; **DNArrp**, DNA repair regulator protein; **DNArtr,** DNA repair transcriptional regulator; **Dnr,** dissimilatory nitrite reductase; **Dpbpp**, dipeptide binding periplasmic protein; **DppA2,** putative dipeptide binding periplasmic protein; **DppbB2**, putative dipeptide transport system; **Duf**, domain of unknown function; **EamAltf**, EamA like transporter family; **ECoAhyd**, enoyl CoA hydratase; **EmrE**,

transmembrane protein; **Est**, esterase; **Excsr**, extracytoplasmic solute receptor; **FAD/FMNcdh**, FAD/FMN containing dehydrogenase; **Fe/Zn urp**, Fe/Zn uptake regulation protein; **Fer**, ferredoxin; **Fe-Scp**, Fe-S cluster protein; **Flif**, flagellar M-ring protein; **Fmt**, formyltransferase protein; **FNt**, formate/nitrite transporter; **Fsnr**, ferredoxin subunit of nitrite reductase; **Fthf**, formyltetrahydrofolate; **GAFdcp**, GAF domain containing protein; **GIIci**, group II catalytic intron; **Glns**, Gln synthetase; **Gluc**, gluconolactonase; **Gly/Daaox,** Gly/D amino acid oxidase; **Goxald,** glyoxalase like domain; **Gr**, glutathione reductase; **Grkes**, glutathione-regulated K-efflux system; **Gt**, glycosyl transferase; **GTPase tef**, GTPase translation elongation factor; **Guak**, guanylate kinase; **Guf**, glycerol uptake facilitator; **Hacdhhyd**, haloacid dehydrogenase hydrolase; **Hbpp**, hemin binding periplasmic protein; **Hcmp**, hypothetical conserved membrane protein; **Hcp**, hybrid cluster protein; **Hdac**, histone deacetylase; **Hlnsph-ns**, histone like nucleoid-structuring protein H-NS; **Hmrr**, heavy metal response regulator; **Hmsq**, heavy metal sensor quinase; **Hp**, hypothetical protein; **Hphat**, histidinol phosphate aminotransferase; **HSerdh**, homoserine dehydrogenase; **Htp**, hemin transport protein; **Hts**, Hemin transport system; **Hyd**, hydrolase, **Int**, integrase; **Ipfor**, indol pyruvate ferredoxin oxidoreductase; **IseppA**, intracellular septation protein A; **Jtp**, J transmembrane protein, **KeftKts**, kef type K transport system; **L-2at4cah**, L-2-amino-thiazoline-4-carboxylic acid hydrolase; **LemAfp**, LemA family protein; **Lgl**, lactoylgluthathione lyase; **L-Glns**, L-Gln synthetase; **Ligα-β-diox**, lignostilbene α-β-dioxygenase; **Lip**, lipase; **LoGfp**, LoG family protein; **LolE**, ABC transporter; **Lr1e**, L-rhamnose 1-epimerase; **Lrp**, long range pseudoknot; **LRVp**, LRV protein; **LysR**, regulatory protein; **MarRs**, transcriptional regulator; **Mcc**, muconate and chloromucanate cycloisomerase; **Mcp**, methyl accepting chemotaxis protein; **McpE**, methyl accepting chemotaxis protein; **McpX1**, methyl accepting chemotaxis protein; **Metl**, methylase; **Met-tRNAft**, Methionyl tRNA formyltransferase; **Mglr**, monothiol glutaredoxin; **MgtA**, mannosyltransferase; **Mi2dh**, myoinositol 2-dehydrogenase; **Mmcp**, methylase of methyl accepting chemotaxis protein; **MoaE**, molybdopterin biosynthesis protein; **Mocsdp**, Mo cofactor synthesis domain containing protein; **Moptcf**, molybdopterin converting factor; **Mphest**, metallophosphoesterase; **Mtccd**, membrane transporter of cation and cationic drugs; **Mtd**, methyltransferase domain; **MtrK**, methylthioribose kinase; **Mδi**, muconolactone δ-isomerase; **NADadh**, NAD dependent aldehyde dehydrogenases; **NAD(P)Hnr**, NAD(P)H nitrite reductase; **NAD(P)Hdh**, NAD(P)H dehydrogenase; **NADPHqr**, NADPH quinone reductase; **NasA**, nitrate reductase; **Nat**, N-acetyltransferase; **Nep**, nitrite extrusion protein; **NifA**, transcriptional regulator; **NifB**, responsible for formation of a functional Mo-Fe nitrogenase; **NifH**, Mo nitrogenase iron protein subunit; **NifQ**, responsible for formation of a functional Mo-Fe nitrogenase; **NifZ**, nitrogenase Mo-Fe maturation protein; **NIPSNAP**; **Nnt**, nitrate/nitrite transporter; **NosD**, periplasmic Cu-binding precursor; **NosF**, Cu-ABC transporter; **NosL**, N_2O reduction; **NosR**, regulatory protein for N_2O reductase; **NosX**, N_2O reduction; **NosY**, N_2O metabolic protein; **NosZ**, nitrous oxide reductase precursor; **Nr,** nitrite reductase; **NrpPtsN**, nitrogene regulatory protein PtsN; **NsrR**, transcriptional regulator; **Nstp**, N-succinyltransferase protein; **Nt**, nitrate transport; **Nterdtmd**, N-terminal double transmembrane domain; **NTPpph**, NTP

pyrophosphatase; **NTP-pphh**, NTP pyrophosphohydrolases; **NtrP**, nitrogen regulatory protein; **NtrR1**, nitrogen regulatory protein; **Nuct**, nucleotidyltransferase; **Omrp**, outer membrane receptor protein; **Oxr**, oxidoreductase; **OxrNADbd/FADbd**, oxidoreductase NAD binding domain/FAD binding domain; **P3Bs**, precorrin 3B synthase; **P3hyalks**, Poly (3-hydroxyalkanoate) synthetase; **PABCt**, probable ABC transporter; **PABCtbcaats**, Putative ABC type branch chain amino acid transport system; **PABCtp**, putative ABC transport protein; **Pacpaahyd**, Putative acetylpolyamine aminohydrolase; **Pact,** probable acetyltransferase; **PASd**, PAS domain; **PASdsb/dgc**, PAS domain S-box/diguanylate cyclase; **PASf**, PAS fold; **Pblrp**, putative bleomycin resistance protein; **Pc1,2dox**, protocatequate 1,2 dioxygenase; **Pcbp**, predicted cobalamin binding protein; **Pcbs**, protein involved in cellulose biosynthesis; **Pcd**, protocatechuate 3,4 dioxygenase; **PcK**, putative carbohydrate kinase; **PcSETd**, protein containing SET domain; **Pcthd**, protein containing a thioredoxin domain; **PCuep**, putative copper export protein; **Pdf**, peptide deformylase; **Pdh**, putative dehydrogenase; **Pdiox**, probable dioxygenase; **Pdlh**, predicted dienelactone hydrolase; **Pdmt**, permease of the drug/metabolite transporter; **PDNArp**, putative DNA recombination protein; **PDNAup**, putative DNA uptake protein; **Pdpp**, putative dipeptidase protein; **Pdthdsi**, predicted dithiol-disulfide isomerase; **Pemp**, protein export membrane protein; **Perll**, predicted enzyme related to lactoylglutatione lyase; **PG**, general secretion pathway proteins; **PGFO/IDH/MocAfox**, PGFO/IDH/MocA family oxidoreductase; **PGloxa**, probable glyoxalase; **PGlns**, probable Gln synthetase; **Pgt**, putative glycosyltransferase protein; **PH**, general secretion pathway proteins; **PhABCt**, phosphate ABC transporter; **Phacdhhyd**, putative haloacid dehydrogenase hydrolase; **Phediox**, phenylpropionate dioxygenase; **Pheh**, phenylhydantoinase; **PheS**, phenylalanyl-tRNA synthetase; **Phmm,** Phosphomannomutase; **Phts**, putative hemin transport system; **Phyd**, predicted hydrolase; **PI**, general secretion pathway proteins; **Pimp**, predicted integral membrane protein; **PJ**, general secretion pathway proteins; **PK**, general secretion pathway proteins; **PKes**, putative K efflux system; **Plipp**, putative lipoprotein; **PM38**, peptidase M38; **Pmbp**, periplasmic molybdate-binding protein; **Pmcp**, probable methyl accepting chemotaxis protein; **Pmobp**, periplasmic molybdate binding protein; **Pmp**, predicted membrane protein; **Pmscbe**, predicted metal sulfur cluster biosynthetic enzyme; **Pmskp**, plasmid maintenance system killer protein; **Pmv/erp**, probable methyl viologen/ethidium resistance protein; **Pmv/ertmp**, probable methyl viologen/ethidium resistance transmembrane protein; **Pnabp**, predicted nucleic acid binding protein; **Pnr**, putative nitrite reductase; **Pomp**, predicted outer membrane protein; **Por**, putative oxidoreductase; **Poxr**, predicted oxydereductase; **Ppbp**, putative periplasmic binding protein; **Ppdf**, putative peptide deformylase; **Ppdmt**, predicted permease of the drug/metabolite transporter**;** **Ppep**, putative permease protein; **Ppp**, predicted periplasmic protein; **Ppphde**, predicted pyridoxal phosphate dependent enzyme; **Ppsp**, probable plasmid stabilization protein; **Ppt**, preprotein translocase; **PQQddh**, PQQ dependent dehydrogenase; **PRCbd**, PRC barrel domain; **Psazu**, Pseudoazurin; **Psl**,

Pseudouridylate synthase I; **Psp**, plasmid stability protein; **Pspp**, probable signal peptide protein; **Pta**, probable transcriptional activator; **Ptcp**, putative transporter component protein; **Ptefp**, putative transmembrane efflux protein; **Ptep**, putative transmembrane exporting protein ; **Ptglcp**, putative transglutaminase-like cysteine peptidase; **Pthie**, predicted thioesterase; **Ptiesp**, putative transmembrane ion efflux system protein; **Ptif**, putative transcriptional initiation factor; **Ptii**, putative translation initiation inhibitor; **PtmcABCt**, putative transmembrane component ABC transporter; **Ptmp**, putative transmembrane protein; **Ptr**, probable transcriptional regulator; **PtsNch**, protein N(pi)-phosphohistidine-sugar phosphotransferase; **Puf**, protein of unknown function; **Pup**, putative uncharacterized protein; **Qacrp**, quaternary ammonium compound resistance protein; **Racbe**, regulator of the catabolism of benzoate; **RbsK**, ribose kinase protein; **RecR**, recombination protein; **RecX**, DNA repair transcriptional regulator; **Rieske FE-Sp**, Rieske Fe-S protein; **RNApolσf**, RNA polymerase σ factor; **RNDes**, RND efflux system outer membrane lipoprotein; **Rep**, regulatory protein; **Rp**, ribosomal protein; **Rr**, response regulator; **Rrf2fp**, Rrf2 family protein; **Rsip**, ribosomal subunit interfase protein; **Sdapdsp**, succinyl diaminopimelate desuccinylase protein; **SDR**, short chain dehydrogenase; **SecD1**, translocase; **Ser/Thrpk**, ser/thr protein kinase; **Shk**, shikimate kinase; **Sk**, sugar kinase; **Smdrp**, small multidrug resistance protein; **Sox**, Sulfite oxidase; **Sp**, secretion protein; **StdB**, plasmid stability protein; **StHk**, signal transduction histidine kinase; **Sucgbtp**, succinoglycan biosynthesis transport protein; **SufA**, iron sulfur cluster assembly transcriptional regulator; **SufB**, iron sulfur cluster assembly protein; **SufC**, iron sulfur cluster assembly protein; **SufD**, iron sulfur cluster assembly protein; **SulTfABCt**, SulT family ABC transporter; **T**, transposase; **Tatp**, transcription antitermination protein; **Tdsi**, thiol-disulfide isomerase; **Tef**, translation elongation factor; **TetR**, regulatory protein; **Tgap**, Transglycosylase associated protein; **Tgle,** Transglutaminase like enzyme; **Thir**, thioredoxin reductase; **Thrs**, threonine synthase; **ThuBch**, myoinositol dehydrogenase; **Tif**, translation initiation factor; **Tl**, translocase; **Tmup**, trehalose maltose utilization protein; **TonBfp**, TonB family protein; **Tr**, transcriptional regulator; **TrcB**, thiosulfate reductase cytochrome B; **tRNA-Arg**; **tRNApsI**, RNAt pseudouridine synthase I; **tRNA-Thr**; **tRNA-Trp**; **TruA**, tRNA pseudouridine synthase; **Tt**, transcriptional termination; **Tt/atf**, transcriptional termination/antitermination factor; **Tuf**, elongation factor, **Tup,** trehalose utilization protein; **Uadh**, Uncharacterized anaerobic dehydrogenase; **UDPhxt**, UDP hexose transferase; **Uebexps**, uncharacterized enzyme involved in biosynthesis of extracellular polysaccharides; **UIIIs**, uroporphyrinogen III synthase; **Ump**, uncharacterized membrane protein; **Up**, uncharacterized protein; **UrSAMp**, uncharacterized radical SAM protein; **Usmp**, uncharacterized small membrane protein; **Zn-ddp**, Zn-dependent dipeptidase.
