# Supplementary material for: A bioinformatics insight to rhizobial globins: gene identification and mapping, polypeptide sequence and phenetic analysis, and protein modeling
Source: F1000Res. 2015 May 13;4:117. [Version 1] doi: 10.12688/f1000research.6392.1 (PMC4648194; doi:10.12688/f1000research.6392.1)
Supplement: Supplementary file 5 [file f1000research-4-6858-s0004.tgz › 9c36283e-d557-4f76-b60e-5f9b3638f2b5.pdf]

Figure S2
Gesto-Borroto et al.

```
                                    10        20        30        40        50
                                     |         |         |         |         |
BraelkUSDA3254Hbt          --------------------------------------------------
BraelkUSDA3259Hbt          --------------------------------------------------
BraelkUSDA76Hbt2           --------------------------------------------------
Braelk587Hbt1              --------------------------------------------------
BraelkUSDA94Hbt2           --------------------------------------------------
BraelkWSM2783Hbt           --------------------------------------------------
BraelkWSM1741Hbt2          --------------------------------------------------
BrajapUSDA4Hbt1            --------------------------------------------------
BrajapWSM2793Hbt1          --------------------------------------------------
BrajapUSDA123Hbt1          --------------------------------------------------
BrajapUSDA6-8372Hbt1       --------------------------------------------------
BrajapUSDA6-7488Hbt1       --------------------------------------------------
BrajapUSDA38Hbt1           --------------------------------------------------
Brajapin8p8Hbt1            --------------------------------------------------
Brajapis5Hbt1              --------------------------------------------------
BrajapUSDA135Hbt1          --------------------------------------------------
BrajapUSDA122Hbt1          --------------------------------------------------
BrajapUSDA124Hbt1          --------------------------------------------------
BrajapWSM1743Hbt1          --------------------------------------------------
Brajap22Hbt1               --------------------------------------------------
AzodoeUFLA1-100Hbt1        --------------------------------------------------
MeslotNZP2037Hbt2          --------------------------------------------------
MeslotUSDA3471Hbt1         --------------------------------------------------
RhietlCIAT652Hbt2          --------------------------------------------------
RhietlCNPAF512Hbt          --------------------------------------------------
RhietlBrasil5Hbt           --------------------------------------------------
RhietlCFN42Hbt2            --------------------------------------------------
Rhileg3841Hbt2             --------------------------------------------------
RhilegWSM1455Hbt2          --------------------------------------------------
RhilegVF39Hbt1             --------------------------------------------------
RhilegWSM1481Hbt2          --------------------------------------------------
RhilegUPM1131Hbt2          --------------------------------------------------
Rhileg248Hbt2              --------------------------------------------------
RhilegTOMHbt2              --------------------------------------------------
Rhileg128C53Hbt1           --------------------------------------------------
RhilegPs8Hbt2              --------------------------------------------------
RhilegGB30Hbt2             --------------------------------------------------
RhilegUPM1137Hbt2          --------------------------------------------------
RhilegVc2Hbt2              --------------------------------------------------
RhilegVh3Hbt1              --------------------------------------------------
Sinmel1021Hbt2             --------------------------------------------------
RhilupHPC_L_Hbt1           --------------------------------------------------
RhilupHPC_L_Hbt2           --------------------------------------------------
AgrtumHbtC2                --------------------------------------------------
SinfreUSDA257Hbt           --------------------------------------------------
SinfreHH103Hbt             --------------------------------------------------
SinfreGR64Hbt              --------------------------------------------------
Sinmel1021Hbt1             --------------------------------------------------
RhilegUPM1137Hbt1          --------------------------------------------------
Rhileg128C53Hbt2           --------------------------------------------------
RhilegVc2Hbt1              --------------------------------------------------
RhilegVh3Hbt2              --------------------------------------------------
RhilegGB30Hbt1             --------------------------------------------------
RhilegPs8Hbt1              --------------------------------------------------
Rhileg3841Hbt1             --------------------------------------------------
Rhileg248Hbt1              --------------------------------------------------
RhilegWSM1455Hbt1          --------------------------------------------------
RhilegWSM1481Hbt1          --------------------------------------------------
RhilegVF39Hbt2             --------------------------------------------------
RhilegTOMHbt1              --------------------------------------------------
RhilegUPM1131Hbt1          --------------------------------------------------
RhietlKIM5Hbt              --------------------------------------------------
RhietlCFN42Hbt1            --------------------------------------------------
Rhietl8C3Hbt               --------------------------------------------------
```

```
RhietlCIAT652Hbt1          --------------------------------------------------
RhietlCIAT894Hbt           --------------------------------------------------
RhietlIE4771Hbt            --------------------------------------------------
AzodoeUFLA1-100Hbt2        --------------------------------------------------
Brajap22Hbt2               --------------------------------------------------
BrajapUSDA110Hbt           --------------------------------------------------
BrajapUSDA122Hbt2          --------------------------------------------------
BrajapUSDA124Hbt2          --------------------------------------------------
BrajapWSM1743Hbt2          --------------------------------------------------
BrajapUSDA6-8372Hbt2       --------------------------------------------------
BrajapUSDA6-7488Hbt2       --------------------------------------------------
BrajapUSDA38Hbt2           --------------------------------------------------
BrajapUSDA123Hbt2          --------------------------------------------------
BrajapWSM2793Hbt2          --------------------------------------------------
BrajapUSDA135Hbt2          --------------------------------------------------
BrajapUSDA4Hbt3            --------------------------------------------------
Brajap22Hbt3               --------------------------------------------------
Brajapin8p8Hbt2            --------------------------------------------------
Brajapis5Hbt2              --------------------------------------------------
CupnecHPC_L_Hbt            --------------------------------------------------
CupnecJMP134Hbt            --------------------------------------------------
CupnecN1Hbt2               --------------------------------------------------
BurphySTM815Hbt2           --------------------------------------------------
BraelkUSDA76Hbt1           MTDDNSAQGPSDKQQPRKHIVVRRNGPYEPDPDISIVDHLGVPIAAAAPA
Braelk587Hbt2              --------------------------------------------------
BraelkUSDA94Hbt1           MTDDNSAQGPSDKQQPRKRIVVRRNGPYEPDPDIPIVDHLGVPIAAKAAV
MescicCMG6Hbt              --------------------------------------------------
MescicWSM1271Hbt           --------------------------------------------------
MescicWSM4083Hbt           --------------------------------------------------
MeslotR7AHbt               --------------------------------------------------
MeslotMAFF303099Hbt        --------------------------------------------------
MeslotNZP2037Hbt1          --------------------------------------------------
MeslotR88bHbt              --------------------------------------------------
MeslotCJ3symHbt            --------------------------------------------------
MeslotUSDA3471Hbt2         --------------------------------------------------
BurphySTM815fHb            --------------------------------------------------
CupnecHPC_L_fHb            --------------------------------------------------
CupnecN1fHb2               --------------------------------------------------
Sinmel1021fHb              --------------------------------------------------
BacsubfHb                  --------------------------------------------------
VitSDgb                    --------------------------------------------------
EsccolfHb                  --------------------------------------------------
RhilegUPM1137fHb           --------------------------------------------------
CupnecN1fHb1               --------------------------------------------------
CupnecJMP134fHb            --------------------------------------------------
SaccerfHb                  --------------------------------------------------
BrajapUSDA110SDgb1         --------------------------------------------------
BrajapUSDA122SDgb1         --------------------------------------------------
Brajapin8p8SDgb            --------------------------------------------------
Brajapis5SDgb              --------------------------------------------------
BrajapUSDA135SDgb1         --------------------------------------------------
BrajapWSM1743SDgb          --------------------------------------------------
BrajapUSDA124SDgb1         --------------------------------------------------
Brajap22SDgb               --------------------------------------------------
BrajapUSDA6-7488SDgb1      --------------------------------------------------
BrajapUSDA6-8372SDgb2      --------------------------------------------------
BrajapUSDA123SDgb1         --------------------------------------------------
BrajapUSDA38SDgb1          --------------------------------------------------
BrajapUSDA4SDgb1           --------------------------------------------------
BrajapWSM2793SDgb1         --------------------------------------------------
BraelkUSDA3254SDgb4        --------------------------------------------------
BraelkUSDA3259SDgb1        --------------------------------------------------
BraelkUSDA94SDgb1          --------------------------------------------------
Braelk587SDgb1             --------------------------------------------------
BraelkUSDA76SDgb1          --------------------------------------------------
BraelkWSM2783SDgb1         --------------------------------------------------
AzodoeUFLA1-100SDgb        --------------------------------------------------
BraelkWSM1741SDgb1         --------------------------------------------------
```

```
BraelkWSM2783SDgb2          --------------------------------------------------
BraelkUSDA3254SDgb2         --------------------------------------------------
BraelkUSDA3259SDgb2         --------------------------------------------------
BraelkUSDA3254SDgb3         --------------------------------------------------
BraelkUSDA3259SDgb4         --------------------------------------------------
Braelk587SDgb2              --------------------------------------------------
BraelkUSDA76SDgb2           --------------------------------------------------
BraelkUSDA94SDgb2           --------------------------------------------------
BrajapUSDA135SDgb2          --------------------------------------------------
BraelkUSDA3254SDgb1         --------------------------------------------------
BraelkUSDA3259SDgb3         --------------------------------------------------
BraelkUSDA94SDgb3           --------------------------------------------------
BraelkUSDA76SDgb3           --------------------------------------------------
Braelk587SDgb3              --------------------------------------------------
BraelkWSM2783SDgb3          --------------------------------------------------
BraelkWSM1741SDgb2          --------------------------------------------------
BrajapUSDA110SDgb2          --------------------------------------------------
BrajapUSDA122SDgb2          --------------------------------------------------
BrajapUSDA4SDgb2            --------------------------------------------------
BrajapWSM2793SDgb2          --------------------------------------------------
BrajapUSDA6-7488SDgb2       --------------------------------------------------
BrajapUSDA6-8372SDgb1       --------------------------------------------------
BrajapUSDA38SDgb2           --------------------------------------------------
BrajapUSDA123SDgb2          --------------------------------------------------
BrajapUSDA124SDgb2          --------------------------------------------------
Brajapin8p8GCS              --------------------------------------------------
Brajapis5GCS                --------------------------------------------------
BraelkWSM2783GCS            --------------------------------------------------
RhilegWSM1481GCS2           --------------------------------------------------
Rhileg248GCS2               --------------------------------------------------
RhilegTOMGCS2               --------------------------------------------------
RhilegUPM1131GCS            --------------------------------------------------
RhilegVF39GCS2              --------------------------------------------------
RhietlCIAT894GCS            --------------------------------------------------
RhilegVh3GCS2               --------------------------------------------------
Rhileg128C53GCS2            --------------------------------------------------
RhilegPs8GCS2               --------------------------------------------------
RhilegGB30GCS2              --------------------------------------------------
RhilegUPM1137GCS2           --------------------------------------------------
RhilegWSM1455GCS2           --------------------------------------------------
Rhileg3841GCS2              --------------------------------------------------
RhilegVc2GCS2               --------------------------------------------------
Rhietl8C3GCS                --------------------------------------------------
RhietlCIAT652GCS2           --------------------------------------------------
RhietlCFN42GCS2             --------------------------------------------------
SinfreGR64GCS               --------------------------------------------------
SinfreHH103GCS              --------------------------------------------------
SinfreUSDA257GCS            --------------------------------------------------
Sinmel1021GCS               --------------------------------------------------
RhietlCIAT652GCS1           --------------------------------------------------
RhietlCNPAF512GCS           --------------------------------------------------
RhietlGR56GCS               --------------------------------------------------
Rhileg128C53GCS1            --------------------------------------------------
RhilegUPM1137GCS1           --------------------------------------------------
RhilegGB30GCS1              --------------------------------------------------
RhilegPs8GCS1               --------------------------------------------------
RhilegVc2GCS1               --------------------------------------------------
RhilegVh3GCS1               --------------------------------------------------
RhilegVF39GCS1              --------------------------------------------------
Rhileg3841GCS1              --------------------------------------------------
RhilegWSM1455GCS1           --------------------------------------------------
RhilegWSM1481GCS1           --------------------------------------------------
Rhileg248GCS1               --------------------------------------------------
RhilegTOMGCS1               --------------------------------------------------
RhietlCFN42GCS1             --------------------------------------------------
AgrtumGCS                   --------------------------------------------------
MyctubHbtC1                 --------------------------------------------------
CupnecN1Hbt1                --------------------------------------------------
```

```
BrajapUSDA4Hbt2            --------------------------------------------------
BrajapWSM2793Hbt3          --------------------------------------------------
MyctubHbtC2                --------------------------------------------------
BurphySTM815Hbt1           --------------------------------------------------
MycaviHbtC3                --------------------------------------------------
BraelkWSM1741Hbt1          --------------------------------------------------
BacsubGCS                  --------------------------------------------------

                                    60        70        80        90       100
                                    |         |         |         |         |
BraelkUSDA3254Hbt          --------------------------------------------------
BraelkUSDA3259Hbt          --------------------------------------------------
BraelkUSDA76Hbt2           --------------------------------------------------
Braelk587Hbt1              --------------------------------------------------
BraelkUSDA94Hbt2           --------------------------------------------------
BraelkWSM2783Hbt           --------------------------------------------------
BraelkWSM1741Hbt2          --------------------------------------------------
BrajapUSDA4Hbt1            --------------------------------------------------
BrajapWSM2793Hbt1          --------------------------------------------------
BrajapUSDA123Hbt1          --------------------------------------------------
BrajapUSDA6-8372Hbt1       --------------------------------------------------
BrajapUSDA6-7488Hbt1       --------------------------------------------------
BrajapUSDA38Hbt1           --------------------------------------------------
Brajapin8p8Hbt1            --------------------------------------------------
Brajapis5Hbt1              --------------------------------------------------
BrajapUSDA135Hbt1          --------------------------------------------------
BrajapUSDA122Hbt1          --------------------------------------------------
BrajapUSDA124Hbt1          --------------------------------------------------
BrajapWSM1743Hbt1          --------------------------------------------------
Brajap22Hbt1               --------------------------------------------------
AzodoeUFLA1-100Hbt1        --------------------------------------------------
MeslotNZP2037Hbt2          --------------------------------------------------
MeslotUSDA3471Hbt1         --------------------------------------------------
RhietlCIAT652Hbt2          --------------------------------------------------
RhietlCNPAF512Hbt          --------------------------------------------------
RhietlBrasil5Hbt           --------------------------------------------------
RhietlCFN42Hbt2            --------------------------------------------------
Rhileg3841Hbt2             --------------------------------------------------
RhilegWSM1455Hbt2          --------------------------------------------------
RhilegVF39Hbt1             --------------------------------------------------
RhilegWSM1481Hbt2          --------------------------------------------------
RhilegUPM1131Hbt2          --------------------------------------------------
Rhileg248Hbt2              --------------------------------------------------
RhilegTOMHbt2              --------------------------------------------------
Rhileg128C53Hbt1           --------------------------------------------------
RhilegPs8Hbt2              --------------------------------------------------
RhilegGB30Hbt2             --------------------------------------------------
RhilegUPM1137Hbt2          --------------------------------------------------
RhilegVc2Hbt2              --------------------------------------------------
RhilegVh3Hbt1              --------------------------------------------------
Sinmel1021Hbt2             --------------------------------------------------
RhilupHPC_L_Hbt1           --------------------------------------------------
RhilupHPC_L_Hbt2           --------------------------------------------------
AgrtumHbtC2                --------------------------------------------------
SinfreUSDA257Hbt           --------------------------------------------------
SinfreHH103Hbt             --------------------------------------------------
SinfreGR64Hbt              --------------------------------------------------
Sinmel1021Hbt1             --------------------------------------------------
RhilegUPM1137Hbt1          --------------------------------------------------
Rhileg128C53Hbt2           --------------------------------------------------
RhilegVc2Hbt1              --------------------------------------------------
RhilegVh3Hbt2              --------------------------------------------------
RhilegGB30Hbt1             --------------------------------------------------
RhilegPs8Hbt1              --------------------------------------------------
Rhileg3841Hbt1             --------------------------------------------------
Rhileg248Hbt1              --------------------------------------------------
RhilegWSM1455Hbt1          --------------------------------------------------
RhilegWSM1481Hbt1          --------------------------------------------------
```

RhilegVF39Hbt2	--
RhilegTOMHbt1	--
RhilegUPM1131Hbt1	--
RhietlKIM5Hbt	--
RhietlCFN42Hbt1	--
Rhietl8C3Hbt	--
RhietlCIAT652Hbt1	--
RhietlCIAT894Hbt	--
RhietlIE4771Hbt	--
AzodoeUFLA1-100Hbt2	--
Brajap22Hbt2	--
BrajapUSDA110Hbt	--
BrajapUSDA122Hbt2	--
BrajapUSDA124Hbt2	--
BrajapWSM1743Hbt2	--
BrajapUSDA6-8372Hbt2	--
BrajapUSDA6-7488Hbt2	--
BrajapUSDA38Hbt2	--
BrajapUSDA123Hbt2	--
BrajapWSM2793Hbt2	--
BrajapUSDA135Hbt2	--
BrajapUSDA4Hbt3	--
Brajap22Hbt3	--
Brajapin8p8Hbt2	--
Brajapis5Hbt2	--
CupnecHPC_L_Hbt	--
CupnecJMP134Hbt	--
CupnecN1Hbt2	--
BurphySTM815Hbt2	--
BraelkUSDA76Hbt1	RLCRCGQSQSQPFCDDSHVARGFTDARDPRRVPDRLDVYAGQQAYVFDNR
Braelk587Hbt2	--
BraelkUSDA94Hbt1	RLCRCGQSQTKPFCDDSHVARGFTDARDPRRVPDRLEVYAGQQAYVFDNR
MescicCMG6Hbt	--
MescicWSM1271Hbt	--
MescicWSM4083Hbt	--
MeslotR7AHbt	--
MeslotMAFF303099Hbt	--
MeslotNZP2037Hbt1	--
MeslotR88bHbt	--
MeslotCJ3symHbt	--
MeslotUSDA3471Hbt2	--
BurphySTM815fHb	--
CupnecHPC_L_fHb	--
CupnecN1fHb2	--
Sinmel1021fHb	--
BacsubfHb	--
VitSDgb	--
EsccolfHb	--
RhilegUPM1137fHb	--
CupnecN1fHb1	--
CupnecJMP134fHb	--
SaccerfHb	--
BrajapUSDA110SDgb1	--
BrajapUSDA122SDgb1	--
Brajapin8p8SDgb	--
Brajapis5SDgb	--
BrajapUSDA135SDgb1	--
BrajapWSM1743SDgb	--
BrajapUSDA124SDgb1	--
Brajap22SDgb	--
BrajapUSDA6-7488SDgb1	--
BrajapUSDA6-8372SDgb2	--
BrajapUSDA123SDgb1	--
BrajapUSDA38SDgb1	--
BrajapUSDA4SDgb1	--
BrajapWSM2793SDgb1	--
BraelkUSDA3254SDgb4	--
BraelkUSDA3259SDgb1	--

```
BraelkUSDA94SDgb1          --------------------------------------------------
Braelk587SDgb1             --------------------------------------------------
BraelkUSDA76SDgb1          --------------------------------------------------
BraelkWSM2783SDgb1         --------------------------------------------------
AzodoeUFLA1-100SDgb        --------------------------------------------------
BraelkWSM1741SDgb1         --------------------------------------------------
BraelkWSM2783SDgb2         --------------------------------------------------
BraelkUSDA3254SDgb2        --------------------------------------------------
BraelkUSDA3259SDgb2        --------------------------------------------------
BraelkUSDA3254SDgb3        --------------------------------------------------
BraelkUSDA3259SDgb4        --------------------------------------------------
Braelk587SDgb2             --------------------------------------------------
BraelkUSDA76SDgb2          --------------------------------------------------
BraelkUSDA94SDgb2          --------------------------------------------------
BrajapUSDA135SDgb2         --------------------------------------------------
BraelkUSDA3254SDgb1        --------------------------------------------------
BraelkUSDA3259SDgb3        --------------------------------------------------
BraelkUSDA94SDgb3          --------------------------------------------------
BraelkUSDA76SDgb3          --------------------------------------------------
Braelk587SDgb3             --------------------------------------------------
BraelkWSM2783SDgb3         --------------------------------------------------
BraelkWSM1741SDgb2         --------------------------------------------------
BrajapUSDA110SDgb2         --------------------------------------------------
BrajapUSDA122SDgb2         --------------------------------------------------
BrajapUSDA4SDgb2           --------------------------------------------------
BrajapWSM2793SDgb2         --------------------------------------------------
BrajapUSDA6-7488SDgb2      --------------------------------------------------
BrajapUSDA6-8372SDgb1      --------------------------------------------------
BrajapUSDA38SDgb2          --------------------------------------------------
BrajapUSDA123SDgb2         --------------------------------------------------
BrajapUSDA124SDgb2         --------------------------------------------------
Brajapin8p8GCS             --------------------------------------------------
Brajapis5GCS               --------------------------------------------------
BraelkWSM2783GCS           --------------------------------------------------
RhilegWSM1481GCS2          --------------------------------------------------
Rhileg248GCS2              --------------------------------------------------
RhilegTOMGCS2              --------------------------------------------------
RhilegUPM1131GCS           --------------------------------------------------
RhilegVF39GCS2             --------------------------------------------------
RhietlCIAT894GCS           --------------------------------------------------
RhilegVh3GCS2              --------------------------------------------------
Rhileg128C53GCS2           --------------------------------------------------
RhilegPs8GCS2              --------------------------------------------------
RhilegGB30GCS2             --------------------------------------------------
RhilegUPM1137GCS2          --------------------------------------------------
RhilegWSM1455GCS2          --------------------------------------------------
Rhileg3841GCS2             --------------------------------------------------
RhilegVc2GCS2              --------------------------------------------------
Rhietl8C3GCS               --------------------------------------------------
RhietlCIAT652GCS2          --------------------------------------------------
RhietlCFN42GCS2            --------------------------------------------------
SinfreGR64GCS              --------------------------------------------------
SinfreHH103GCS             --------------------------------------------------
SinfreUSDA257GCS           --------------------------------------------------
Sinmel1021GCS              --------------------------------------------------
RhietlCIAT652GCS1          --------------------------------------------------
RhietlCNPAF512GCS          --------------------------------------------------
RhietlGR56GCS              --------------------------------------------------
Rhileg128C53GCS1           --------------------------------------------------
RhilegUPM1137GCS1          --------------------------------------------------
RhilegGB30GCS1             --------------------------------------------------
RhilegPs8GCS1              --------------------------------------------------
RhilegVc2GCS1              --------------------------------------------------
RhilegVh3GCS1              --------------------------------------------------
RhilegVF39GCS1             --------------------------------------------------
Rhileg3841GCS1             --------------------------------------------------
RhilegWSM1455GCS1          --------------------------------------------------
RhilegWSM1481GCS1          --------------------------------------------------
```

```
Rhileg248GCS1              --------------------------------------------------
RhilegTOMGCS1              --------------------------------------------------
RhietlCFN42GCS1            --------------------------------------------------
AgrtumGCS                  --------------------------------------------------
MyctubHbtC1                --------------------------------------------------
CupnecN1Hbt1               --------------------------------------------------
BrajapUSDA4Hbt2            --------------------------------------------------
BrajapWSM2793Hbt3          --------------------------------------------------
MyctubHbtC2                --------------------------------------------------
BurphySTM815Hbt1           --------------------------------------------------
MycaviHbtC3                --------------------------------------------------
BraelkWSM1741Hbt1          --------------------------------------------------
BacsubGCS                  --------------------------------------------------

                                   110       120       130       140       150
                                    |         |         |         |         |
BraelkUSDA3254Hbt          --------------------------------------------------
BraelkUSDA3259Hbt          --------------------------------------------------
BraelkUSDA76Hbt2           --------------------------------------------------
Braelk587Hbt1              --------------------------------------------------
BraelkUSDA94Hbt2           --------------------------------------------------
BraelkWSM2783Hbt           --------------------------------------------------
BraelkWSM1741Hbt2          --------------------------------------------------
BrajapUSDA4Hbt1            --------------------------------------------------
BrajapWSM2793Hbt1          --------------------------------------------------
BrajapUSDA123Hbt1          --------------------------------------------------
BrajapUSDA6-8372Hbt1       --------------------------------------------------
BrajapUSDA6-7488Hbt1       --------------------------------------------------
BrajapUSDA38Hbt1           --------------------------------------------------
Brajapin8p8Hbt1            --------------------------------------------------
Brajapis5Hbt1              --------------------------------------------------
BrajapUSDA135Hbt1          --------------------------------------------------
BrajapUSDA122Hbt1          --------------------------------------------------
BrajapUSDA124Hbt1          --------------------------------------------------
BrajapWSM1743Hbt1          --------------------------------------------------
Brajap22Hbt1               --------------------------------------------------
AzodoeUFLA1-100Hbt1        --------------------------------------------------
MeslotNZP2037Hbt2          --------------------------------------------------
MeslotUSDA3471Hbt1         --------------------------------------------------
RhietlCIAT652Hbt2          --------------------------------------------------
RhietlCNPAF512Hbt          --------------------------------------------------
RhietlBrasil5Hbt           --------------------------------------------------
RhietlCFN42Hbt2            --------------------------------------------------
Rhileg3841Hbt2             --------------------------------------------------
RhilegWSM1455Hbt2          --------------------------------------------------
RhilegVF39Hbt1             --------------------------------------------------
RhilegWSM1481Hbt2          --------------------------------------------------
RhilegUPM1131Hbt2          --------------------------------------------------
Rhileg248Hbt2              --------------------------------------------------
RhilegTOMHbt2              --------------------------------------------------
Rhileg128C53Hbt1           --------------------------------------------------
RhilegPs8Hbt2              --------------------------------------------------
RhilegGB30Hbt2             --------------------------------------------------
RhilegUPM1137Hbt2          --------------------------------------------------
RhilegVc2Hbt2              --------------------------------------------------
RhilegVh3Hbt1              --------------------------------------------------
Sinmel1021Hbt2             --------------------------------------------------
RhilupHPC_L_Hbt1           --------------------------------------------------
RhilupHPC_L_Hbt2           --------------------------------------------------
AgrtumHbtC2                --------------------------------------------------
SinfreUSDA257Hbt           --------------------------------------------------
SinfreHH103Hbt             --------------------------------------------------
SinfreGR64Hbt              --------------------------------------------------
Sinmel1021Hbt1             --------------------------------------------------
RhilegUPM1137Hbt1          --------------------------------------------------
Rhileg128C53Hbt2           --------------------------------------------------
RhilegVc2Hbt1              --------------------------------------------------
RhilegVh3Hbt2              --------------------------------------------------
```

```
RhilegGB30Hbt1           --------------------------------------------------
RhilegPs8Hbt1            --------------------------------------------------
Rhileg3841Hbt1           --------------------------------------------------
Rhileg248Hbt1            --------------------------------------------------
RhilegWSM1455Hbt1        --------------------------------------------------
RhilegWSM1481Hbt1        --------------------------------------------------
RhilegVF39Hbt2           --------------------------------------------------
RhilegTOMHbt1            --------------------------------------------------
RhilegUPM1131Hbt1        --------------------------------------------------
RhietlKIM5Hbt            --------------------------------------------------
RhietlCFN42Hbt1          --------------------------------------------------
Rhietl8C3Hbt             --------------------------------------------------
RhietlCIAT652Hbt1        --------------------------------------------------
RhietlCIAT894Hbt         --------------------------------------------------
RhietlIE4771Hbt          --------------------------------------------------
AzodoeUFLA1-100Hbt2      --------------------------------------------------
Brajap22Hbt2             --------------------------------------------------
BrajapUSDA110Hbt         --------------------------------------------------
BrajapUSDA122Hbt2        --------------------------------------------------
BrajapUSDA124Hbt2        --------------------------------------------------
BrajapWSM1743Hbt2        --------------------------------------------------
BrajapUSDA6-8372Hbt2     --------------------------------------------------
BrajapUSDA6-7488Hbt2     --------------------------------------------------
BrajapUSDA38Hbt2         --------------------------------------------------
BrajapUSDA123Hbt2        --------------------------------------------------
BrajapWSM2793Hbt2        --------------------------------------------------
BrajapUSDA135Hbt2        --------------------------------------------------
BrajapUSDA4Hbt3          --------------------------------------------------
Brajap22Hbt3             --------------------------------------------------
Brajapin8p8Hbt2          --------------------------------------------------
Brajapis5Hbt2            --------------------------------------------------
CupnecHPC_L_Hbt          --------------------------------------------------
CupnecJMP134Hbt          --------------------------------------------------
CupnecN1Hbt2             --------------------------------------------------
BurphySTM815Hbt2         --------------------------------------------------
BraelkUSDA76Hbt1         GTCAHSGFCTDRLASVFRLDEEPFIAPSGARLDDLINAVRRCPSGALGIG
Braelk587Hbt2            ------------------LDEEPFIAPSGARLDDLINAVRRCPSGALGIG
BraelkUSDA94Hbt1         GTCAHSGFCTNRLASVFRLGEQPFIAPSGARLDDLINAVRRCPSGALGIG
MescicCMG6Hbt            --------------------------------------------------
MescicWSM1271Hbt         --------------------------------------------------
MescicWSM4083Hbt         --------------------------------------------------
MeslotR7AHbt             --------------------------------------------------
MeslotMAFF303099Hbt      --------------------------------------------------
MeslotNZP2037Hbt1        --------------------------------------------------
MeslotR88bHbt            --------------------------------------------------
MeslotCJ3symHbt          --------------------------------------------------
MeslotUSDA3471Hbt2       --------------------------------------------------
BurphySTM815fHb          --------------------------------------------------
CupnecHPC_L_fHb          --------------------------------------------------
CupnecN1fHb2             --------------------------------------------------
Sinmel1021fHb            --------------------------------------------------
BacsubfHb                --------------------------------------------------
VitSDgb                  --------------------------------------------------
EsccolfHb                --------------------------------------------------
RhilegUPM1137fHb         --------------------------------------------------
CupnecN1fHb1             --------------------------------------------------
CupnecJMP134fHb          --------------------------------------------------
SaccerfHb                --------------------------------------------------
BrajapUSDA110SDgb1       --------------------------------------------------
BrajapUSDA122SDgb1       --------------------------------------------------
Brajapin8p8SDgb          --------------------------------------------------
Brajapis5SDgb            --------------------------------------------------
BrajapUSDA135SDgb1       --------------------------------------------------
BrajapWSM1743SDgb        --------------------------------------------------
BrajapUSDA124SDgb1       --------------------------------------------------
Brajap22SDgb             --------------------------------------------------
BrajapUSDA6-7488SDgb1    --------------------------------------------------
BrajapUSDA6-8372SDgb2    --------------------------------------------------
```

```
BrajapUSDA123SDgb1         --------------------------------------------------
BrajapUSDA38SDgb1          --------------------------------------------------
BrajapUSDA4SDgb1           --------------------------------------------------
BrajapWSM2793SDgb1         --------------------------------------------------
BraelkUSDA3254SDgb4        --------------------------------------------------
BraelkUSDA3259SDgb1        --------------------------------------------------
BraelkUSDA94SDgb1          --------------------------------------------------
Braelk587SDgb1             --------------------------------------------------
BraelkUSDA76SDgb1          --------------------------------------------------
BraelkWSM2783SDgb1         --------------------------------------------------
AzodoeUFLA1-100SDgb        --------------------------------------------------
BraelkWSM1741SDgb1         --------------------------------------------------
BraelkWSM2783SDgb2         --------------------------------------------------
BraelkUSDA3254SDgb2        --------------------------------------------------
BraelkUSDA3259SDgb2        --------------------------------------------------
BraelkUSDA3254SDgb3        --------------------------------------------------
BraelkUSDA3259SDgb4        --------------------------------------------------
Braelk587SDgb2             --------------------------------------------------
BraelkUSDA76SDgb2          --------------------------------------------------
BraelkUSDA94SDgb2          --------------------------------------------------
BrajapUSDA135SDgb2         --------------------------------------------------
BraelkUSDA3254SDgb1        --------------------------------------------------
BraelkUSDA3259SDgb3        --------------------------------------------------
BraelkUSDA94SDgb3          --------------------------------------------------
BraelkUSDA76SDgb3          --------------------------------------------------
Braelk587SDgb3             --------------------------------------------------
BraelkWSM2783SDgb3         --------------------------------------------------
BraelkWSM1741SDgb2         --------------------------------------------------
BrajapUSDA110SDgb2         --------------------------------------------------
BrajapUSDA122SDgb2         --------------------------------------------------
BrajapUSDA4SDgb2           --------------------------------------------------
BrajapWSM2793SDgb2         --------------------------------------------------
BrajapUSDA6-7488SDgb2      --------------------------------------------------
BrajapUSDA6-8372SDgb1      --------------------------------------------------
BrajapUSDA38SDgb2          --------------------------------------------------
BrajapUSDA123SDgb2         --------------------------------------------------
BrajapUSDA124SDgb2         --------------------------------------------------
Brajapin8p8GCS             --------------------------------------------------
Brajapis5GCS               --------------------------------------------------
BraelkWSM2783GCS           --------------------------------------------------
RhilegWSM1481GCS2          --------------------------------------------------
Rhileg248GCS2              --------------------------------------------------
RhilegTOMGCS2              --------------------------------------------------
RhilegUPM1131GCS           --------------------------------------------------
RhilegVF39GCS2             --------------------------------------------------
RhietlCIAT894GCS           --------------------------------------------------
RhilegVh3GCS2              --------------------------------------------------
Rhileg128C53GCS2           --------------------------------------------------
RhilegPs8GCS2              --------------------------------------------------
RhilegGB30GCS2             --------------------------------------------------
RhilegUPM1137GCS2          --------------------------------------------------
RhilegWSM1455GCS2          --------------------------------------------------
Rhileg3841GCS2             --------------------------------------------------
RhilegVc2GCS2              --------------------------------------------------
Rhietl8C3GCS               --------------------------------------------------
RhietlCIAT652GCS2          --------------------------------------------------
RhietlCFN42GCS2            --------------------------------------------------
SinfreGR64GCS              --------------------------------------------------
SinfreHH103GCS             --------------------------------------------------
SinfreUSDA257GCS           --------------------------------------------------
Sinmel1021GCS              --------------------------------------------------
RhietlCIAT652GCS1          --------------------------------------------------
RhietlCNPAF512GCS          --------------------------------------------------
RhietlGR56GCS              --------------------------------------------------
Rhileg128C53GCS1           --------------------------------------------------
RhilegUPM1137GCS1          --------------------------------------------------
RhilegGB30GCS1             --------------------------------------------------
RhilegPs8GCS1              --------------------------------------------------
```

```
RhilegVc2GCS1              --------------------------------------------------
RhilegVh3GCS1              --------------------------------------------------
RhilegVF39GCS1             --------------------------------------------------
Rhileg3841GCS1             --------------------------------------------------
RhilegWSM1455GCS1          --------------------------------------------------
RhilegWSM1481GCS1          --------------------------------------------------
Rhileg248GCS1              --------------------------------------------------
RhilegTOMGCS1              --------------------------------------------------
RhietlCFN42GCS1            --------------------------------------------------
AgrtumGCS                  --------------------------------------------------
MyctubHbtC1                --------------------------------------------------
CupnecN1Hbt1               --------------------------------------------------
BrajapUSDA4Hbt2            --------------------------------------------------
BrajapWSM2793Hbt3          --------------------------------------------------
MyctubHbtC2                --------------------------------------------------
BurphySTM815Hbt1           --------------------------------------------------
MycaviHbtC3                --------------------------------------------------
BraelkWSM1741Hbt1          --------------------------------------------------
BacsubGCS                  --------------------------------------------------


                                   160       170       180       190       200
                                    |         |         |         |         |
BraelkUSDA3254Hbt          --------------------------------------------------
BraelkUSDA3259Hbt          --------------------------------------------------
BraelkUSDA76Hbt2           --------------------------------------------------
Braelk587Hbt1              --------------------------------------------------
BraelkUSDA94Hbt2           --------------------------------------------------
BraelkWSM2783Hbt           --------------------------------------------------
BraelkWSM1741Hbt2          --------------------------------------------------
BrajapUSDA4Hbt1            --------------------------------------------------
BrajapWSM2793Hbt1          --------------------------------------------------
BrajapUSDA123Hbt1          ----------------------------MATRLTANTVAHPSRRFAASHP
BrajapUSDA6-8372Hbt1       --------------------------------------------------
BrajapUSDA6-7488Hbt1       --------------------------------------------------
BrajapUSDA38Hbt1           --------------------------------------------------
Brajapin8p8Hbt1            --------------------------------------------------
Brajapis5Hbt1              --------------------------------------------------
BrajapUSDA135Hbt1          ------------------------------------LRCDNDAARNWPDN
BrajapUSDA122Hbt1          --------------------------------------------------
BrajapUSDA124Hbt1          --------------------------------------------------
BrajapWSM1743Hbt1          --------------------------------------------------
Brajap22Hbt1               --------------------------------------------------
AzodoeUFLA1-100Hbt1        --------------------------------------------------
MeslotNZP2037Hbt2          --------------------------------------------------
MeslotUSDA3471Hbt1         --------------------------------------------------
RhietlCIAT652Hbt2          --------------------------------------------------
RhietlCNPAF512Hbt          --------------------------------------------------
RhietlBrasil5Hbt           --------------------------------------------------
RhietlCFN42Hbt2            --------------------------------------------------
Rhileg3841Hbt2             --------------------------------------------------
RhilegWSM1455Hbt2          --------------------------------------------------
RhilegVF39Hbt1             --------------------------------------------------
RhilegWSM1481Hbt2          --------------------------------------------------
RhilegUPM1131Hbt2          --------------------------------------------------
Rhileg248Hbt2              --------------------------------------------------
RhilegTOMHbt2              --------------------------------------------------
Rhileg128C53Hbt1           --------------------------------------------------
RhilegPs8Hbt2              --------------------------------------------------
RhilegGB30Hbt2             --------------------------------------------------
RhilegUPM1137Hbt2          --------------------------------------------------
RhilegVc2Hbt2              --------------------------------------------------
RhilegVh3Hbt1              --------------------------------------------------
Sinmel1021Hbt2             --------------------------------------------------
RhilupHPC_L_Hbt1           --------------------------------------------------
RhilupHPC_L_Hbt2           --------------------------------------------------
AgrtumHbtC2                --------------------------------------------------
SinfreUSDA257Hbt           --------------------------------------------------
```

```
SinfreHH103Hbt          --------------------------------------------------
SinfreGR64Hbt           --------------------------------------------------
Sinmel1021Hbt1          --------------------------------------------------
RhilegUPM1137Hbt1       --------------------------------------------------
Rhileg128C53Hbt2        --------------------------------------------------
RhilegVc2Hbt1           --------------------------------------------------
RhilegVh3Hbt2           --------------------------------------------------
RhilegGB30Hbt1          --------------------------------------------------
RhilegPs8Hbt1           --------------------------------------------------
Rhileg3841Hbt1          --------------------------------------------------
Rhileg248Hbt1           --------------------------------------------------
RhilegWSM1455Hbt1       --------------------------------------------------
RhilegWSM1481Hbt1       --------------------------------------------------
RhilegVF39Hbt2          --------------------------------------------------
RhilegTOMHbt1           --------------------------------------------------
RhilegUPM1131Hbt1       --------------------------------------------------
RhietlKIM5Hbt           --------------------------------------------------
RhietlCFN42Hbt1         --------------------------------------------------
Rhietl8C3Hbt            --------------------------------------------------
RhietlCIAT652Hbt1       --------------------------------------------------
RhietlCIAT894Hbt        --------------------------------------------------
RhietlIE4771Hbt         --------------------------------------------------
AzodoeUFLA1-100Hbt2     --------------------------------------------------
Brajap22Hbt2            --------------------------------------------------
BrajapUSDA110Hbt        --------------------------------------------------
BrajapUSDA122Hbt2       --------------------------------------------------
BrajapUSDA124Hbt2       --------------------------------------------------
BrajapWSM1743Hbt2       --------------------------------------------------
BrajapUSDA6-8372Hbt2    --------------------------------------------------
BrajapUSDA6-7488Hbt2    --------------------------------------------------
BrajapUSDA38Hbt2        --------------------------------------------------
BrajapUSDA123Hbt2       --------------------------------------------------
BrajapWSM2793Hbt2       --------------------------------------------------
BrajapUSDA135Hbt2       --------------------------------------------------
BrajapUSDA4Hbt3         --------------------------------------------------
Brajap22Hbt3            --------------------------------------------------
Brajapin8p8Hbt2         --------------------------------------------------
Brajapis5Hbt2           --------------------------------------------------
CupnecHPC_L_Hbt         --------------------------------------------------
CupnecJMP134Hbt         --------------------------------------------------
CupnecN1Hbt2            --------------------------------------------------
BurphySTM815Hbt2        --------------------------------------------------
BraelkUSDA76Hbt1        IGPERDDNLSDVNRPPQIEVSKDGPYRVTGHVELVDEDGASIARNAGASQ
Braelk587Hbt2           IGPERDDNLSDVNRPPQIEVSKDGPYRVTGHVELVDEDGASIARNAGASQ
BraelkUSDA94Hbt1        IGPERNANLSDVNRPPQIEVSKDGPYRVTGHVELVDEDGASITRNAGASQ
MescicCMG6Hbt           --------------------------------------------------
MescicWSM1271Hbt        --------------------------------------------------
MescicWSM4083Hbt        --------------------------------------------------
MeslotR7AHbt            --------------------------------------------------
MeslotMAFF303099Hbt     --------------------------------------------------
MeslotNZP2037Hbt1       --------------------------------------------------
MeslotR88bHbt           --------------------------------------------------
MeslotCJ3symHbt         --------------------------------------------------
MeslotUSDA3471Hbt2      --------------------------------------------------
BurphySTM815fHb         --------------------------------------------------
CupnecHPC_L_fHb         --------------------------------------------------
CupnecN1fHb2            --------------------------------------------------
Sinmel1021fHb           --------------------------------------------------
BacsubfHb               --------------------------------------------------
VitSDgb                 --------------------------------------------------
EsccolfHb               --------------------------------------------------
RhilegUPM1137fHb        --------------------------------------------------
CupnecN1fHb1            --------------------------------------------------
CupnecJMP134fHb         --------------------------------------------------
SaccerfHb               --------------------------------------------------
BrajapUSDA110SDgb1      --------------------------------------------------
BrajapUSDA122SDgb1      --------------------------------------------------
Brajapin8p8SDgb        --------------------------------------------------
```

```
Brajapis5SDgb              --------------------------------------------------
BrajapUSDA135SDgb1         --------------------------------------------------
BrajapWSM1743SDgb          --------------------------------------------------
BrajapUSDA124SDgb1         --------------------------------------------------
Brajap22SDgb               --------------------------------------------------
BrajapUSDA6-7488SDgb1      --------------------------------------------------
BrajapUSDA6-8372SDgb2      --------------------------------------------------
BrajapUSDA123SDgb1         --------------------------------------------------
BrajapUSDA38SDgb1          --------------------------------------------------
BrajapUSDA4SDgb1           --------------------------------------------------
BrajapWSM2793SDgb1         --------------------------------------------------
BraelkUSDA3254SDgb4        --------------------------------------------------
BraelkUSDA3259SDgb1        --------------------------------------------------
BraelkUSDA94SDgb1          --------------------------------------------------
Braelk587SDgb1             --------------------------------------------------
BraelkUSDA76SDgb1          --------------------------------------------------
BraelkWSM2783SDgb1         --------------------------------------------------
AzodoeUFLA1-100SDgb        --------------------------------------------------
BraelkWSM1741SDgb1         --------------------------------------------------
BraelkWSM2783SDgb2         --------------------------------------------------
BraelkUSDA3254SDgb2        --------------------------------------------------
BraelkUSDA3259SDgb2        --------------------------------------------------
BraelkUSDA3254SDgb3        --------------------------------------------------
BraelkUSDA3259SDgb4        --------------------------------------------------
Braelk587SDgb2             --------------------------------------------------
BraelkUSDA76SDgb2          --------------------------------------------------
BraelkUSDA94SDgb2          --------------------------------------------------
BrajapUSDA135SDgb2         --------------------------------------------------
BraelkUSDA3254SDgb1        --------------------------------------------------
BraelkUSDA3259SDgb3        --------------------------------------------------
BraelkUSDA94SDgb3          --------------------------------------------------
BraelkUSDA76SDgb3          --------------------------------------------------
Braelk587SDgb3             --------------------------------------------------
BraelkWSM2783SDgb3         --------------------------------------------------
BraelkWSM1741SDgb2         -------------MVYQFGLRLADRQSLVVQVSGGALTRTSSVASTIKIK
BrajapUSDA110SDgb2         --------------------------------------------------
BrajapUSDA122SDgb2         --------------------------------------------------
BrajapUSDA4SDgb2           --------------------------------------------------
BrajapWSM2793SDgb2         --------------------------------------------------
BrajapUSDA6-7488SDgb2      --------------------------------------------------
BrajapUSDA6-8372SDgb1      --------------------------------------------------
BrajapUSDA38SDgb2          --------------------------------------------------
BrajapUSDA123SDgb2         --------------------------------------------------
BrajapUSDA124SDgb2         --------------------------------------------------
Brajapin8p8GCS             --------------------------------------------------
Brajapis5GCS               --------------------------------------------------
BraelkWSM2783GCS           --------------------------------------------------
RhilegWSM1481GCS2          --------------------------------------------------
Rhileg248GCS2              --------------------------------------------------
RhilegTOMGCS2              --------------------------------------------------
RhilegUPM1131GCS           --------------------------------------------------
RhilegVF39GCS2             --------------------------------------------------
RhietlCIAT894GCS           --------------------------------------------------
RhilegVh3GCS2              --------------------------------------------------
Rhileg128C53GCS2           --------------------------------------------------
RhilegPs8GCS2              --------------------------------------------------
RhilegGB30GCS2             --------------------------------------------------
RhilegUPM1137GCS2          --------------------------------------------------
RhilegWSM1455GCS2          --------------------------------------------------
Rhileg3841GCS2             --------------------------------------------------
RhilegVc2GCS2              --------------------------------------------------
Rhietl8C3GCS               --------------------------------------------------
RhietlCIAT652GCS2          --------------------------------------------------
RhietlCFN42GCS2            --------------------------------------------------
SinfreGR64GCS              --------------------------------------------------
SinfreHH103GCS             --------------------------------------------------
SinfreUSDA257GCS           --------------------------------------------------
Sinmel1021GCS              --------------------------------------------------
```

```
RhietlCIAT652GCS1         --------------------------------------------------
RhietlCNPAF512GCS         --------------------------------------------------
RhietlGR56GCS             --------------------------------------------------
Rhileg128C53GCS1          --------------------------------------------------
RhilegUPM1137GCS1         --------------------------------------------------
RhilegGB30GCS1            --------------------------------------------------
RhilegPs8GCS1             --------------------------------------------------
RhilegVc2GCS1             --------------------------------------------------
RhilegVh3GCS1             --------------------------------------------------
RhilegVF39GCS1            --------------------------------------------------
Rhileg3841GCS1            --------------------------------------------------
RhilegWSM1455GCS1         --------------------------------------------------
RhilegWSM1481GCS1         --------------------------------------------------
Rhileg248GCS1             --------------------------------------------------
RhilegTOMGCS1             --------------------------------------------------
RhietlCFN42GCS1           ------------------------------------MFNHIDQNLCRRIP
AgrtumGCS                 --------------------------------------------------
MyctubHbtC1               --------------------------------------------------
CupnecN1Hbt1              --------------------------------------------------
BrajapUSDA4Hbt2           -------------------------------------------MARPRST
BrajapWSM2793Hbt3         -------------------------------------------MARPRST
MyctubHbtC2               --------------------------------------------------
BurphySTM815Hbt1          --------------------------------------------------
MycaviHbtC3               --------------------------------------------------
BraelkWSM1741Hbt1         --------------------------------------------------
BacsubGCS                 -------------------------------------MLFKKDRKQETAY

                                   210       220       230       240       250
                                     |         |         |         |         |
BraelkUSDA3254Hbt         ------------------------MTAAERREQITAGIVARTGITEAMIE
BraelkUSDA3259Hbt         ------------------------MTAAERREQITAGIVARTGITEAMIE
BraelkUSDA76Hbt2          ------------------------MTAAERREQITAGIVARTGINEAMIE
Braelk587Hbt1             ------------------------MTAAERREQITAGIVARTGISEAMIE
BraelkUSDA94Hbt2          ------------------------MTGAERREQITAGIVARTGITEAMIE
BraelkWSM2783Hbt          ------------------------MTGAERREQITADIVAKTGITEAMIE
BraelkWSM1741Hbt2         --------------------MEAIVAGPERRERLVAEIMERTGIDEAMIE
BrajapUSDA4Hbt1           ---------------MSDRLKAEREAAAARRNLLTQDAIERTGITEEMIG
BrajapWSM2793Hbt1         ---------------MSDRLKAEREAAAARRNLLTQDAIERTGITEEMIG
BrajapUSDA123Hbt1         LPQGERGRQNGRRKSMSDRLKAEREAAAARRNLLTQDAIERTGITEEMIG
BrajapUSDA6-8372Hbt1      ---------------MSDRLKAEREAAAARRNLLTQDAIERTGITEEMIG
BrajapUSDA6-7488Hbt1      -------------------MKAEREAAAARRNLLTQDAIERTGITEEMIG
BrajapUSDA38Hbt1          ---------------MSDRLKAEREAAAARRNLLTQDAIERTGITEEMIG
Brajapin8p8Hbt1           ---------------MSDRLKAEREAAAARRSLLTQDAIERTGITEEMIG
Brajapis5Hbt1             ---------------MSDRLKAEREAAAARRSLLTQDAIERTGITEEMIG
BrajapUSDA135Hbt1         VERAARGNNGTDTEDMSDRLKAEREAAAARRSLLTQDAIERTGINEEMIG
BrajapUSDA122Hbt1         ---------------MSDRLKAEREAAAARRNLLTQDAIERTGITEEMIA
BrajapUSDA124Hbt1         ---------------MSDRLKAEREAAAVRRNLLTQDAIERTGITEEMIG
BrajapWSM1743Hbt1         ---------------MSDRLKAEREAAAARRSLLTQDAIERTGITEEMIG
Brajap22Hbt1              ---------------MSDRLKAEREAAAARRSLLTQDAIERTGITEEMIA
AzodoeUFLA1-100Hbt1       --------------------------------------MKHARIDEPAIA
MeslotNZP2037Hbt2         --------------------MTLKSSLADHARPAPEKPLHHAGVDRAAIG
MeslotUSDA3471Hbt1        --------------------MTFNSAPIERVVPVAEEPLDKAGVDRQSID
RhietlCIAT652Hbt2         ----------------MDNDIQGRPAHVAAIRERAEAEMRAMGVDEAFIG
RhietlCNPAF512Hbt         ----------------MDNDIQGRPAHVAAIRERAEAEMRAMGVDEAFIG
RhietlBrasil5Hbt          ----------------MDNDIQGRPAHVAAIRERAEAEMRAMGVDEAFIG
RhietlCFN42Hbt2           ----------------MNNDIQGRPAHVAAIREKAEAEMRAMGVDEAFIG
Rhileg3841Hbt2            ----------------MDNDIQGRPAHVAAIRERAEAEMREMGVDAAFID
RhilegWSM1455Hbt2         ----------------MDNDIQGRPAHVAAIRERAEAEMREMGVDAAFID
RhilegVF39Hbt1            --------------------------------------MREMGVDAAFID
RhilegWSM1481Hbt2         ----------------MDNEIQGRPAHVAAIRERAEAEMREMGVDAAFID
RhilegUPM1131Hbt2         --------------------------------------MREMGVDAAFID
Rhileg248Hbt2             ----------------MDNEIQGRPAHVAAIRERAEAEMREMGVDAAFID
RhilegTOMHbt2             ----------------MDNENQGRPAHVAAIRERAEAEMREMGVDAAFID
Rhileg128C53Hbt1          --------------------------------------MRDMGVDAAFID
RhilegPs8Hbt2             --------------------------------------MRDMGVDAAFID
RhilegGB30Hbt2            --------------------------------------MRDMGVDAAFID
RhilegUPM1137Hbt2         --------------------------------------MRDMGVDAAFID
RhilegVc2Hbt2             --------------------------------------MRDMGVDGAFID
```

```
RhilegVh3Hbt1               --------------------------------------MRDMGVDGAFID
Sinmel1021Hbt2              ----------------MSDELKGHAVQSAAMRERAEAEMKALGIDEAFIG
RhilupHPC_L_Hbt1            ---------------MMQNPAAARAAHSAEIQDRAEKAMAAIGVDARFID
RhilupHPC_L_Hbt2            -------------------------------MTGETITLYEAIGGDATVR
AgrtumHbtC2                 -------------------------------MSGETITLYEAIGGDATVR
SinfreUSDA257Hbt            ----------MGGHCARCGAAIRSRSKDTDMTETNTTTLYEAIGGDAAVR
SinfreHH103Hbt              ------------------------------MTETKTTTLYEAIGGDAAVR
SinfreGR64Hbt               ------------------------------MTETKTTTLYEAIGGDAAVR
Sinmel1021Hbt1              ------------------------------MTEPQTTTLYEAIGGDATVR
RhilegUPM1137Hbt1           ------------------MKGLGIRMASDGFVTEKVTTLYEAIGGDPVVR
Rhileg128C53Hbt2            -------------------------MAWDGFVTEKITTLYQAVGGDPVVR
RhilegVc2Hbt1               -------MTVAIIFRPVHEEAGVGPGSGDGFVTEKVTTLYQAIGGDPVVR
RhilegVh3Hbt2               -------MTVAIIFRPVHEEAGVGPGSGDGFVTEKVTTLYQAIGGDPVVR
RhilegGB30Hbt1              -------MTVAIISPPVHEEAGVGPGSGDGFVTEKVTTLYQAIGGDPVVR
RhilegPs8Hbt1               -------------------------------VTEKVTTLYQAIGGDPVVR
Rhileg3841Hbt1              -------------------------------MTEKVTTLYQAIGGDPVVR
Rhileg248Hbt1               -------------------------------MTEKVTTLYQAIGGDPVVR
RhilegWSM1455Hbt1           -------------------------------MTEKVTTLYQAIGGDPVVR
RhilegWSM1481Hbt1           -------------------------------MTEKVTTLYQAIGGDPVVR
RhilegVF39Hbt2              -------------------------------VTEKVTTLYQAIGGDPVVR
RhilegTOMHbt1               -------------------------------MTEKVTTLYQAIGGDPVVR
RhilegUPM1131Hbt1           -------------------------------VTEKVTTLYQAIGGDPAVR
RhietlKIM5Hbt               -------------------------------MTEKVTTLYQAIGGDHAVR
RhietlCFN42Hbt1             -------------------------------MTEKVTTLYEAIGGDPAVR
Rhietl8C3Hbt                --------MTVAIAFRPVHEGSGDGNDIGDGFVTENTTLYEAIGGDPAVR
RhietlCIAT652Hbt1           --------------------------------MTENTTLYEAIGGDPAVR
RhietlCIAT894Hbt            -------MTVAIIFHQVHEGSGNGDDIGDGFVTEKVTTLYEAIGGDPTVR
RhietlIE4771Hbt             ------------------MKVADGKCIWDGFVTEKVTTLYQAIGGDPAVR
AzodoeUFLA1-100Hbt2         -----------------------------MSEADVQVSIFERIGGPVTID
Brajap22Hbt2                -----------------------------MSDTAITVSMFERIGGPVVID
BrajapUSDA110Hbt            ----------------------------MMTDSDVAISMFERIGGSATID
BrajapUSDA122Hbt2           -----------------------------MTDSDVAISMFERIGGSATID
BrajapUSDA124Hbt2           -----------------------------MTDSDVAISMFERIGGSATID
BrajapWSM1743Hbt2           -----------------------------MTDSTTAISMFERIGGSATID
BrajapUSDA6-8372Hbt2        -----------------------------MTTSDVTTSMFERIGGSTTID
BrajapUSDA6-7488Hbt2        -----------------------------MTTSDVTTSMFERIGGSTTID
BrajapUSDA38Hbt2            -----------------------------MTSSDVTTSMFERIGGSATID
BrajapUSDA123Hbt2           -----------------------------MTSSDVTTSMFERIGGSGTID
BrajapWSM2793Hbt2           -----------------------------MTSTDVTTSMFERIGGSATID
BrajapUSDA135Hbt2           -----------------------------MSSTDVTTSVFERIGGSATID
BrajapUSDA4Hbt3             ------------------------------MTSTDVTTMFERIGGSATID
Brajap22Hbt3                -----------------------------MTDGTVTTSLFERIGGSAAIE
Brajapin8p8Hbt2             -----------------------------MTNIDTSVSLFDRIGGSATID
Brajapis5Hbt2               -----------------------------MTNIDTSVSLFDRIGGSATID
CupnecHPC_L_Hbt             ------------------------------------------------MR
CupnecJMP134Hbt             ----------------------MNTEPESTGQAGTEVTAFDLVGGEARVR
CupnecN1Hbt2                -----------------------MSTESNDKPGTAEVTAFELVGGEARVR
BurphySTM815Hbt2            -------------------------MTDPIDEAPSQPTAFELVGGEARVR
BraelkUSDA76Hbt1            EHVSLCRCGASLNKPFCSGMHWSVAFRDPIPDPMREPTLFEWAGGYPALL
Braelk587Hbt2               EHVSLCRCGASLNKPFCSGMHWSVAFRDPIPDPMREPTLFEWAGGYPALL
BraelkUSDA94Hbt1            EHVSLCRCGASLNKPFCSGMHWNVEFRDPVPDPMREPTLFEWAGGYPALL
MescicCMG6Hbt               -------------------------------MGQDIPTLYEWAGGSEALN
MescicWSM1271Hbt            -------------------------------MGQDIPTLYEWAGGSEALN
MescicWSM4083Hbt            -------------------------------MSQDIPTLYEWAGGSEALN
MeslotR7AHbt                -------------------------------MSRDVPTLYEWAGGSDALN
MeslotMAFF303099Hbt         -------------------------------MSRDVPTLYEWAGGSDALN
MeslotNZP2037Hbt1           -------------------------------MSRDVPTLYEWAGGSDALN
MeslotR88bHbt               -------------------------------MRKDVPTLYEWAGGSDALN
MeslotCJ3symHbt             --------------------------------MRKDPTLYEWAGGSDALN
MeslotUSDA3471Hbt2          -------------------------------MRKDVPTLYEWAGGSDALN
BurphySTM815fHb             ----------------------------MLSAEHRAIVKATVPLLESGGE
CupnecHPC_L_fHb             ----------------------------MLSPNTIALVKATVPVLTQHGE
CupnecN1fHb2                ----------------------------MLTQQTKDIVKATAPVLAAHGY
Sinmel1021fHb               ----------------------------MLTQKTKDIVKATAPVLAQHGY
BacsubfHb                   ----------------------------MLDNKTIEIIKSTVPVLQQHGE
VitSDgb                     ----------------------------MLDQQTINIIKATVPVLKEHGV
EsccolfHb                   ----------------------------MLDAQTIATVKATIPLLVETGP
RhilegUPM1137fHb            -------------------------MPKTLSSETVAAVKATISALDEHGA
```

```
CupnecN1fHb1                ----------------------------MLSAASRPYIDASVPVLREHGL
CupnecJMP134fHb             ----------------------------MLSAASRPYIDASVPVLREHGL
SaccerfHb                   ----------------------------MLAEKTRSIIKATVPVLEQQGT
BrajapUSDA110SDgb1          -----------------------------MTPEQITLIQQSFAKVAPISE
BrajapUSDA122SDgb1          -----------------------------MTPEQITLVQQSFAKVAPISE
Brajapin8p8SDgb             -----------------------------MTPEQVTLIQQSFSKVAPISE
Brajapis5SDgb               -----------------------------MTPEQVTLIQQSFSKVAPISE
BrajapUSDA135SDgb1          -----------------------------MTPEQIALIQQSFAKVAPISE
BrajapWSM1743SDgb           -----------------------------MTPEQITLIQQSFSKVAPISE
BrajapUSDA124SDgb1          -----------------------------MTPEQITLIQQSFAKVAPISE
Brajap22SDgb                -----------------------------MTPEQIALIQQSFSKVAPISE
BrajapUSDA6-7488SDgb1       -----------------------------MTSEQIALVQQSFSKVAPISE
BrajapUSDA6-8372SDgb2       -----------------------------MTSEQIALVQQSFSKVAPISE
BrajapUSDA123SDgb1          -----------------------------MTSEQITLVQQSFSKVAPISE
BrajapUSDA38SDgb1           -----------------------------MTPEQITLIQQSFSKVAPISE
BrajapUSDA4SDgb1            -----------------------------MTPEQITLIQQSFAKVAPISE
BrajapWSM2793SDgb1          -----------------------------MTPEQITLIQQSFAKVAPISQ
BraelkUSDA3254SDgb4         -----------------------------MNPAQIKLVQDSFGKVAPISE
BraelkUSDA3259SDgb1         -----------------------------MNPAQIKLVQDSFGKVAPISE
BraelkUSDA94SDgb1           -----------------------------MNPAQIKLVQESFGKVAPISE
Braelk587SDgb1              -----------------------------MNPAQIKLVQESFGKVAPISE
BraelkUSDA76SDgb1           -----------------------------MNPAQIKLVQESFGKVAPISE
BraelkWSM2783SDgb1          -----------------------------MTPEQVKLVQESFSKVAPISE
AzodoeUFLA1-100SDgb         -----------------------------MTPSQVELVQSSFAKVAPIAD
BraelkWSM1741SDgb1          -----------------------------MSPETKELLKSTWAKVIPISD
BraelkWSM2783SDgb2          -----------------------------VDAVQQELVQTTFARLAAMPE
BraelkUSDA3254SDgb2         -----------------------------MTPEQVDLIGISFDAMWPIRR
BraelkUSDA3259SDgb2         -----------------------------MTPEQVDLIGISFDAMWPIRR
BraelkUSDA3254SDgb3         --------------------------------------------MWPIRR
BraelkUSDA3259SDgb4         --------------------------------------------MWPIRR
Braelk587SDgb2              -----------------------------MTPEQVDLIRTSFDAMWPIRR
BraelkUSDA76SDgb2           -----------------------------MTPEQVDLIRTSFDAMWPIRR
BraelkUSDA94SDgb2           --------------------------------------------------
BrajapUSDA135SDgb2          ----------------------------MLTVEEIVRVRSSFDRVFANAT
BraelkUSDA3254SDgb1         ------------------------------MTPSSNPIERSFELAAAACD
BraelkUSDA3259SDgb3         ------------------------------MTPSSNPIERSFELAAAACD
BraelkUSDA94SDgb3           ------------------------------MTPSSNPIERSFELAAAACD
BraelkUSDA76SDgb3           ------------------------------MTPSSNPIERSFELAAAACD
Braelk587SDgb3              --------------------------------------------------
BraelkWSM2783SDgb3          -------------------------------MSSANPIERSFELAAARCD
BraelkWSM1741SDgb2          SQFSRKLRVDTIFEQGLVPCSFFSLRSQTTMTASSNPIERSFELAAARCE
BrajapUSDA110SDgb2          -------------------------MAPTLMTISPNPIEQSFERAASRCA
BrajapUSDA122SDgb2          ------------------------------MTISPNPIEQSFERAASRCA
BrajapUSDA4SDgb2            ------------------------------MNMPSNPVELSFELAASRCA
BrajapWSM2793SDgb2          ------------------------------MNMPSNPVELSFELAASRCA
BrajapUSDA6-7488SDgb2       --------------------------------------------------
BrajapUSDA6-8372SDgb1       ----------------------------MPMSAPVNSIENSFELAASRCA
BrajapUSDA38SDgb2           ----------------------------MPMSAPLNSIENSFELAASRCA
BrajapUSDA123SDgb2          -------------------------MASMPMSAPSNPIENSFDLAASRCA
BrajapUSDA124SDgb2          --------------------------------------------------
Brajapin8p8GCS              -------MTLAQTPNDENQDRDGRMRFMRVGTETGELLCEFWSVAEPALP
Brajapis5GCS                -------MTLAQTPNDENQDRDGRMRFMRVGTETGELLCEFWSVAEPALP
BraelkWSM2783GCS            --------------VNSAGSLATRLAFHRIDQKTTALLREARSLVIDALP
RhilegWSM1481GCS2           -------MPSDQARGAQAGSLRDRLRFAGLDADQCELVRRNRPALEAYLK
Rhileg248GCS2               -------MPSDQARGAQAGSLRDRLRFAGLDADQCELVRRNRPALEAHLK
RhilegTOMGCS2               -------MPSDQARGAQAGSLRDRLRFAGLDADQCELVRRNRPALEAHLK
RhilegUPM1131GCS            -------MPSDQARGAQAGSLRDRLRFAGLDADQCELVRRNRPALEAHLK
RhilegVF39GCS2              -------MPSDQARGAQAGSLRDRLRFAGLDADQCELVRRNRPALEAHLK
RhietlCIAT894GCS            -------------------------------------MRRNRPALEAHLK
RhilegVh3GCS2               -------MPSDQARGAQAGSLRDRLRFAGLDADQCELVRRNRPALEAHLK
Rhileg128C53GCS2            -------MPSDQARGAQAGSLRDRLRFAGLDADQCDLVRRNRPALEAHLK
RhilegPs8GCS2               -------MPSDQARGAQAGSLRDRLRFAGLDADQCELVRRNRPALEAHLK
RhilegGB30GCS2              -------MPSDQARGAQAGSLRDRLRFAGLDADQCELVRRNRPALEAHLK
RhilegUPM1137GCS2           -------MPSDQARGAQAGSLRDRLRFAGLDADQCELVRRNRPALEAHLK
RhilegWSM1455GCS2           -------MPSDQARGAQAGSLRDRLRFAGLDADQCELVRRNRPALEAHLK
Rhileg3841GCS2              ---MRQDMPSDQARGAQAGSLRDRLRFAGLDADQCELVRRNRPALEAHLK
RhilegVc2GCS2               -------MPSDQARGAQAGSLRDRLRFAGLDAEQCELVRRNRPALEAHLK
Rhietl8C3GCS                --------------------MRDRLRFAGLDADQCELVRRNRPALEAHLK
```

```
RhietlCIAT652GCS2          -------MPSDQARGAQAGSLRDRLRFAGLDADQCELVRRNRPALEAHLK
RhietlCFN42GCS2            -------MPSDQARGAQAGSLRDRLRFAGLDADQCELVRRNRPALKAHLK
SinfreGR64GCS              ----VQQEASEQARKGQAGSLLERLRFAGLDEAACALLRDHRQVLSPRIE
SinfreHH103GCS             ----MQQEASEQARKGQAGSLLERLRFAGLDEAACALLRDHRQVLSPRIE
SinfreUSDA257GCS           ----MQQEASEQARKSQAGSLLERLRFAGLDEAACALLRDHRQALSPRIE
Sinmel1021GCS              ----MKQEASEQARKGQAGSLLERLRFAGLDEDACALLRDHRQALSPRIE
RhietlCIAT652GCS1          -----------MTSQQTDSALKQRLDFIELDEAARKSMRDLRPVISELVG
RhietlCNPAF512GCS          -----------MTSQQTDSALKQRLDFIELDEAARKSMRDLRPVISELVG
RhietlGR56GCS              -----------MTSQQTDSALKQRLDFIELDEAARKSMRELRPVIAELIG
Rhileg128C53GCS1           -----------MTAQQTDNALRQRLDFIELDEAARKSMRDLRPVISELIG
RhilegUPM1137GCS1          -----------MTAQQTDNALRQRLDFIELDEAARKSMRDLRPVISELIG
RhilegGB30GCS1             -----------MTAQQTDNALRQRLDFIELDEAARKSMRDLRPVISELIG
RhilegPs8GCS1              -----------MTAQQTDNALRQRLDFIELDEAARKSMRDLRPVISELIG
RhilegVc2GCS1              -----------MTAQQTDNALRQRLDFIELDEAARKSMRDLRPVISELIG
RhilegVh3GCS1              -----------MTAQQTDNALRQRLDFIELDEAARKSMRDLRPVISELIG
RhilegVF39GCS1             -----------MTAQQTDSALRQRLDFIELDEAARKSMRDLRPVISELIG
Rhileg3841GCS1             -----------MTAQQTDSALRQRLDFIELDEAARKSMRDLRPVISELIG
RhilegWSM1455GCS1          -----------MTAQQTDSALRQRLDFIELDEAARKSMRDLRPVISELIG
RhilegWSM1481GCS1          -----------MTAQQTDSALRQRLDFIELDEAARKSMRDLRPVISELIG
Rhileg248GCS1              -----------MTAQQTDSALRQRLDFIELDEAARKSMRDLRPVISELIG
RhilegTOMGCS1              -----------MTAQQTDSALRQRLDFIELDEAALKSMRDLRPVISELIG
RhietlCFN42GCS1            SRAVAPSTEISMTSQQTDSALRQRLDFIELDEAARRSMRDLSPVISELIG
AgrtumGCS                  ----------MHGQAKTDRQLDERLNFLGLGHGERQNLSDMKGVITGSLD
MyctubHbtC1                --------------------MGLLSRLRKREP----ISIYDKIGGHEAIE
CupnecN1Hbt1               -----MRIPPSLYGVLLAALIGLGGCAMPDKPKPTMPSLYERLGGIQAIT
BrajapUSDA4Hbt2            SISRRSVVAGLALTAAPVALAGANQARAQAQQTTPDQSLYERLGGVFAIA
BrajapWSM2793Hbt3          SISRRSVVAGLALTAAPVALAGANQARAQAQQTTPDQSLYERLGGVFAIA
MyctubHbtC2                ----------------------------------MPKSFYDAVGGAKTFD
BurphySTM815Hbt1           -------------------------MKSDISSDISAAERLATRYAEPSDD
MycaviHbtC3                ----------------------------------------MPDLRDRADVE
BraelkWSM1741Hbt1          ----------------------------------VDKSLYERLGGATGVA
BacsubGCS                  FSDSNGQQKNRIQLTNKHADVKKQLKMVRLGDAELYVLEQLQPLIQENIV

                                                     ➲
2/2-folding helix                                                 [-A-][-------
3/3-folding helix                                          [-------A-------][--

                                    260       270       280       290       300
                                     |         |         |         |         |
BraelkUSDA3254Hbt          QLVHAFYAK---VRKDPMIGPVF---------ETRISN-WEPHLAQMCAF
BraelkUSDA3259Hbt          QLVHAFYAK---VRKDPMIGPVF---------ETRISN-WEPHLAQMCAF
BraelkUSDA76Hbt2           QLVHAFYAK---VRKDPMIGPVF---------GSRISN-WEPHLAQMCAF
Braelk587Hbt1              QLVHAFYAK---VRKDPMIGPVF---------GSRISN-WEPHLAQMCAF
BraelkUSDA94Hbt2           QLVHAFYAE---VRKDPMIGPVF---------ENRISN-WAPHLAQMCAF
BraelkWSM2783Hbt           HLVHGFYAK---VRADAVLGPVF---------EARIRD-WGPHLSQMCAF
BraelkWSM1741Hbt2          RLVRGFYAK---VREDAVLGPIF---------DARIKD-WEPHLNQMCAF
BrajapUSDA4Hbt1            ELVTRFYGR---VREDARLGPVF---------GIVQN--WDEHLAKLRDF
BrajapWSM2793Hbt1          ELVTRFYGR---VREDARLGPVF---------GIVQN--WDEHLAKLRDF
BrajapUSDA123Hbt1          ELVTRFYGR---VREDALLGPVF---------AIVQN--WDEHLAKLRDF
BrajapUSDA6-8372Hbt1       ELVTRFYGR---VREDALLGPVF---------AIVQN--WDEHLAKLREF
BrajapUSDA6-7488Hbt1       ELVTRFYGR---VREDALLGPVF---------AIVQN--WDEHLAKLREF
BrajapUSDA38Hbt1           ELVTRFYGR---VREDALLGPVF---------AIVQN--WDEHLAKLREF
Brajapin8p8Hbt1            ELVTRFYGR---VREDALLGPVF---------AVVQN--WDEHLAKLRDF
Brajapis5Hbt1              ELVTRFYGR---VREDALLGPVF---------AVVQN--WDEHLAKLRDF
BrajapUSDA135Hbt1          ELVTRFYGR---VREDTLLGPVF---------AIVQN--WDEHLAKLRDF
BrajapUSDA122Hbt1          ELVTRFYGR---VREDALLGPVF---------AVVQN--WDEHLAKLGDF
BrajapUSDA124Hbt1          ELVTRFYGR---VRKDALLGPVF---------AIVQN--WDEHLAKLTDF
BrajapWSM1743Hbt1          ELVTRFYGR---VREDALLGPVF---------AIVQN--WDEHFAKLKDF
Brajap22Hbt1               ELVTRFYGR---VREDAKLGPVF---------GVVQN--WDEHLARLRDF
AzodoeUFLA1-100Hbt1        ELVERFYGY---ARADARLGPVF---------AAAVHD-WPGHMATLKDF
MeslotNZP2037Hbt2          RLVREFYAR---LRKNERLGPIF---------AREIPGDWEPHLEKMTDF
MeslotUSDA3471Hbt1         KLVREFYAR---VRNDERLGPIF---------DREISSDWKPHLEKMTDF
RhietlCIAT652Hbt2          RLVETFYGR---VLAHADLGPVF---------DARLSGRWPEHMEKMKSF
RhietlCNPAF512Hbt          RLVETFYGR---VLAHADLGPVF---------DARLSGRWPEHMKKMKSF
RhietlBrasil5Hbt           RLVETFYGR---VLAHADLGPVF---------DARLSGRWPEHMKKMKSF
RhietlCFN42Hbt2            RLVETFYGR---VLEHPDLGAVF---------DARLSGRWPEHMEKMKSF
Rhileg3841Hbt2             RLVETFYGR---VLAHPDLGPVF---------DARLSGRWPEHMAKMKSF
RhilegWSM1455Hbt2          RLVETFYGR---VLAHPDLGPVF---------DARLSGRWPEHMAKMKSF
```

RhilegVF39Hbt1	RLVETFYGR---VLAHPDLGPVF---------DARLSGRWPEHMAKMKSF
RhilegWSM1481Hbt2	RLVETFYGR---VLAHPDLGPVF---------DARLSGRWPEHMAKMKSF
RhilegUPM1131Hbt2	RLVETFYGR---VLTHPDLGPVF---------DARLSGRWPEHMAKMKSF
Rhileg248Hbt2	RLVETFYGR---VLTHPDLGPVF---------DARLSGRWPEHMAKMKSF
RhilegTOMHbt2	RLVETFYGR---VLTHPDLGPVF---------DAKLSGRWPEHMAKMKSF
Rhileg128C53Hbt1	RLVETFYGR---VLTHPDLGPVF---------DAKLSGRWPEHMAKMKSF
RhilegPs8Hbt2	RLVETFYGR---VLTHPDLGPVF---------DAKLSGRWPEHMTKMKSF
RhilegGB30Hbt2	RLVETFYGR---VLTHPDLGPVF---------DARLSGRWPEHMTKMKSF
RhilegUPM1137Hbt2	RLVETFYGR---VLTHPDLGPVF---------DAKLAGRWPEHMAKMKGF
RhilegVc2Hbt2	RLVETFYAR---VLAHPDLGPVF---------DARLSGRWPEHMTKMKSF
RhilegVh3Hbt1	RLVETFYAR---VLAHPDLGPVF---------DARLSGRWPAHMAKMKSF
Sinmel1021Hbt2	KLVDTFYAR---VLAHPELGPVF---------DARLSGRWPEHMEKMKSF
RhilupHPC_L_Hbt1	LLVETFYGR---VLKHPALGPVF---------DARLAGRWPEHMARMKQF
RhilupHPC_L_Hbt2	ALTRRFYQLMDTLPEAARCRAIH---------PADLSGSEAKFYDYMTGY
AgrtumHbtC2	ALTRRFYELMDTLPEAARCRAIH---------PADLSSSEAKFYDYLTGY
SinfreUSDA257Hbt	ALTRRFYELMGSLPEAARCRAVH---------PPDLSGSEEKFYEYLTGW
SinfreHH103Hbt	ALTQRFYELMDSLPEAARCRAVH---------PPDLSGSEEKLYEYLTGW
SinfreGR64Hbt	ALTRRFYELMGSLPEAARCRAVH---------PPDLSGSEEKLYEYLTGW
Sinmel1021Hbt1	ALTQRFYELMDSLPEAARCRAVH---------PPDLTGSEEKFYEYLTGW
RhilegUPM1137Hbt1	ALTHRFYELMDTLPEARNVRAVH---------PPSLTGSEEKFYEYMSGY
Rhileg128C53Hbt2	ALTHRFYELMDTLPEARNVRAVH---------PPSLTGSEEKFYEYMSGY
RhilegVc2Hbt1	ALTHRFYALMDRLPEARNVRAVH---------PPSLTGSEEKFYEYMSGY
RhilegVh3Hbt2	ALTHRFYALMDRLPEARNVRAVH---------PPSLTGSEEKFYEYMSGY
RhilegGB30Hbt1	ALTHRFYALMDTLPEARNVRAMY---------PPSLTGSEEKFYEYMSGY
RhilegPs8Hbt1	ALTHRFYALMDALPEARNVRAVH---------PPSLTGSEEKFYEYMSGY
Rhileg3841Hbt1	ALTHRFYELMDTLPEASNVRAVH---------PPSLEGSEEKFYEYMTGY
Rhileg248Hbt1	ALTHRFYELMDRLPEARNLRAVH---------PPSLTGSEEKFYEYMTGY
RhilegWSM1455Hbt1	ALTHRFYELMDSLPEASNVRAVH---------PPSLVGSEEKFYEYMTGY
RhilegWSM1481Hbt1	ALTHRFYELMDSLPEASNVRAVH---------PPSLVGSEEKFYEYMTGY
RhilegVF39Hbt2	ALTHRFYELMDSLPEARNVRAVH---------PPSLVGSEEKFYEYMTGY
RhilegTOMHbt1	ALTHRFYELMDSLPEARNVRAVH---------PSSLVGSEEKFYEYMTGY
RhilegUPM1131Hbt1	ALTRRFYELMDSLSEVRNVRAVH---------PPSLVGSEEKFYEYMTGY
RhietlKIM5Hbt	ALTRRFYELMDRLPEASNVRAVH---------PPSLQESEEKFYEYMSGY
RhietlCFN42Hbt1	ALTHRFYALMDSLQEAKNVRAVH---------PPSLQGSEEKFYEYMSGY
Rhietl8C3Hbt	ALTHRFYVLMDTLPEASNVRAVH---------PSSLEGSEEKFYEYMSGY
RhietlCIAT652Hbt1	ALTHRFYVLMDTLPEASNVRAVH---------PSSLEGSEEKFYEYMSGY
RhietlCIAT894Hbt	ALTHRFYALMDKLPEASNVRAVH---------PPSLEGSEEKFYEYMTGY
RhietlIE4771Hbt	ALTHRFYELMDTLPQASNVRAVH---------PPTLRESEEKFYEYMSGY
AzodoeUFLA1-100Hbt2	RLVEAFYRRMDSEAEAAHIRTMH---------APDLTNTKSVLKRYLTEW
Brajap22Hbt2	RLVESFYRRMDALPEAAGIRAMH---------ADDLTSTKQVLKRYLSEW
BrajapUSDA110Hbt	LLVDRFYERMDTLPEAKVIRAMH---------AADLGLIRDVLKRYLTEW
BrajapUSDA122Hbt2	LLVDRFYERMDTLPEAQVIRAMH---------AADLGLIRDVLKRYLTEW
BrajapUSDA124Hbt2	RLVDRFYDRMDTLPEAKIIRAMH---------AADLGLIRDVLKRYLTEW
BrajapWSM1743Hbt2	RLVDRFYDRMDTLPEAQVIRAMH---------ADDLGLIRDVLKRYLTEW
BrajapUSDA6-8372Hbt2	ALVDRFYDRMDTLPEAQMIRAMH---------ADDLGLIRDVLKRYLTEW
BrajapUSDA6-7488Hbt2	ALVDRFYDRMDTLPEAQMIRAMH---------ADDLGLIRDVLKRYLTEW
BrajapUSDA38Hbt2	ALVDRFYDRMDTLPEAQLIRTMH---------ADDLGLIRDVLKRYLTEW
BrajapUSDA123Hbt2	LLVDRFYDRMDTLPEAQMIRVMH---------ADDLGLIRDVLKRYLNEW
BrajapWSM2793Hbt2	ALVDRFYERMDTLPEAQVIRAMH---------ADDLGLIRDVLKRYLTEW
BrajapUSDA135Hbt2	LLVDRFYDRMDRLPEAKVIRAMH---------ADDLGLIRDVLKRYLTEW
BrajapUSDA4Hbt3	LLVDRFYKRMDTLPEAQIIRAMH---------ADDLGLIRDVLKRYLTEW
Brajap22Hbt3	ALVDRFYDRMETLPEAKVIRAMH---------ADDLGLIRDVLKRYLTEW
Brajapin8p8Hbt2	ALVDRFYDRMDTLPEAKIIRAMH---------ADDLGPIRDVLKRYLTEW
Brajapis5Hbt2	ALVDRFYDRMDTLPEAKIIRAMH---------ADDLGPIRDVLKRYLTEW
CupnecHPC_L_Hbt	ELVDRFYDLMDLEPQFAGLRALH---------PASMDGSRDKLFWFLCGW
CupnecJMP134Hbt	ELVDRFYDLMDLEPEFAGLRALH---------PASLDGSRDKLFWFLCGW
CupnecN1Hbt2	ELVDRFYDLMDLETQFAGLRALH---------PPSLEGSRDKLFWFLCGW
BurphySTM815Hbt2	ELVDRFYDLMDLEADFARIRKLH---------PPTLEGSRDKLFWFLCGW
BraelkUSDA76Hbt1	DMTRIFYSR--YVPEDPLLGPLF---------AEMSPDHPERVAAWLSEV
Braelk587Hbt2	DMTRIFYSR--YVPEDPLLGPLF---------AEMSPDHPERVAAWLSEV
BraelkUSDA94Hbt1	DMTRIFYSR--YVPEDPLLGPLF---------AEMSPDHPERVAAWLSEV
MescicCMG6Hbt	RLTQTFYD---KVAQDPIVGPVF---------KTMSPDHPAHVAAFIGEV
MescicWSM1271Hbt	RLTQTFYD---KVAQDPIVGPVF---------KTMSPDHPAHVAAFIGEV
MescicWSM4083Hbt	RLTQTFYD---TVAQDPIVGPVF---------KTMSPDHPTHVAAFIGEV
MeslotR7AHbt	RLTD--
MeslotMAFF303099Hbt	RLTQTFYD---KVAKDPVVGPVF---------KAMSPDHPSHVAAFIGEV
MeslotNZP2037Hbt1	RLTQTFYD---KVAKDPVVGPVF---------KTMSPDHPAHVAAFIGEV
MeslotR88bHbt	RLTQTFYD---KVAQDPVVGPVF---------KAMSPDHPAHVAAFIGEV

```
MeslotCJ3symHbt          RLTQTFYD---QVAQDPIVGPVF---------KAMSPDHPAHVAAFIGEV
MeslotUSDA3471Hbt2       RLTQTFYD---KVAQDPIIGPVF---------KTMSPDHPAHVAAFIGEV
BurphySTM815fHb          ALTTHFYKVMLSEYPSVRPLFNQA----HQQSGDQPRALANAVLMYARHI
CupnecHPC_L_fHb          AITQHFYRLLLTQHPELKAFFNEA----HQVHGTQARALAGAVLAFASHV
CupnecN1fHb2             DIIKCFYKRMFEAHPELKNVFNMA----HQEQGQQQQALARAVYAYAENI
Sinmel1021fHb            AIIQHFYKRMFQAHPELKDIFNMA----HQERGEQQQALARAVYAYAANI
BacsubfHb                TITGRFYDLMFQDHPELLNIFNQT----NQKKKTQRAALANAVIAAAANI
VitSDgb                  TITTTFYKNLFAKHPEVRPLFDMG----RQESLEQPKALAMTVLAAAQNI
EsccolfHb                KLTAHFYDRMFTHNPELKEIFNMS----NQRNGDQREALFNAIAAYASNI
RhilegUPM1137fHb         VITAAMYRRLFED-AEIAALFNQS----NQKSGTQIHALAAAILAYARNI
CupnecN1fHb1             AITTHFYREMFADRPELTQMFNMG----NQANGSQQQSLASAVFAYAANI
CupnecJMP134fHb          AITTHFYREMFAARPELTQLFNMG----NQANGSQQQSLASAVFAYAANI
SaccerfHb                VITRTFYKNMLTEHTELLNIFNRT----NQKVGAQPNALATTVLAAAKNI
BrajapUSDA110SDgb1       TAAVLFYDRLFEVAPSVRAMFP-------EDMTEQRKKLMGMLAAVVGGL
BrajapUSDA122SDgb1       TAAVLFYDRLFEVAPSVRAMFP-------EDMTEQRKKLMGMLAAVVGGL
Brajapin8p8SDgb          QAAVLFYDRLFEVAPSVRAMFP-------EDMTEQRKKLMGMLAAVVGGL
Brajapis5SDgb            QAAVLFYDRLFEVAPSVRAMFP-------EDMTEQRKKLMGMLAAVVGGL
BrajapUSDA135SDgb1       QAAVLFYDRLFEVAPSVRAMFP-------EDMTEQRKKLMGMLAAVVGGL
BrajapWSM1743SDgb        QAAVLFYDRLFEVAPSVRAMFP-------EDMTEQRKKLMGMLAAVVGGL
BrajapUSDA124SDgb1       QAAVLFYDRLFEVAPPVRAMFP-------EDMTEQRKKLMGMLAAVVGGL
Brajap22SDgb             QAAVLFYDRLFEVAPSVRAMFP-------EDMTEQRKKLMGMLAAVVGGL
BrajapUSDA6-7488SDgb1    AAAVLFYDRLFEVAPSVRAMFP-------EDMTEQRKKLMGMLAAVVGGL
BrajapUSDA6-8372SDgb2    AAAVLFYDRLFEVAPSVRAMFP-------EDMTEQRKKLMGMLAAVVGGL
BrajapUSDA123SDgb1       AAAVLFYDRLFEVAPSVRAMFP-------EDMTEQRKKLMGMLAAVVGGL
BrajapUSDA38SDgb1        AAAVLFYDRLFEVAPSVRAMFP-------EDMTEQRKKLMGMLAAVVGGL
BrajapUSDA4SDgb1         AASVLFYDRLFEVAPSVRAMFP-------EDMTEQRKKLMGMLAAVVSGL
BrajapWSM2793SDgb1       AASVLFYDRLFEVAPSVRAMFP-------EDMTEQRKKLMGMLAAVVSGL
BraelkUSDA3254SDgb4      QAAVIFYDRLFEVAPAVKAMFP-------VDMKEQRKKLMTTLAVVVNGL
BraelkUSDA3259SDgb1      QAAVIFYDRLFEVAPAVKAMFP-------VDMKEQRKKLMTTLAVVVNGL
BraelkUSDA94SDgb1        QAAVIFYDRLFEVAPAVRAMFP-------ADMKEQRKKLMTTLAVVVNGL
Braelk587SDgb1           QAAVIFYDRLFEVAPAVKAMFP-------ADMKEQRRKLMTTLAVVVNGL
BraelkUSDA76SDgb1        QAAVIFYDRLFEVAPAVKAMFP-------ADMKEQRRKLMTTLAVVVNGL
BraelkWSM2783SDgb1       QAAILFYDRLFEVAPTVRAMFP-------DDMTEQRKKLMAVLAVVVNGL
AzodoeUFLA1-100SDgb      TAAGLFYGRLFETAPEVKPLFK-------GDIAEQGRKLMATLAVVVNGL
BraelkWSM1741SDgb1       VAAGLFYERLFTLDPSLQRLFKN------ADMKVQRRKLVQALSAVINSV
BraelkWSM2783SDgb2       VTGALFYERLFASNPNFRPLFR-------NNMRIQGVKLMTMLAMVVYNL
BraelkUSDA3254SDgb2      DIADLCYSRFVELDPDAKDMFA-------GDIERRRMKVLDMITALVASL
BraelkUSDA3259SDgb2      DIADLCYSRFVELDPDAKDMFA-------GDIERRRMKVLDMITALVASL
BraelkUSDA3254SDgb3      DLADLCYNRFVELAPDARQMFG-------GDTEKQRMKVLDMITALVASL
BraelkUSDA3259SDgb4      DLADLCYNRFVELAPDARQMFG-------GDTEKQRMKVLDMITALVASL
Braelk587SDgb2           DLADLCYNRFVELAPDARSLFG-------GDAEKQRMKMLDMIIALVASL
BraelkUSDA76SDgb2        DLADLCYNRFVELAPDARSLFG-------GDAEKQRMKMLDMIIALVASL
BraelkUSDA94SDgb2        -------------------------------------MKMLDMITALVASL
BrajapUSDA135SDgb2       DMTTTFYDRVFELAPEIRPMFP-------ADMTNLKHDFISKLAVLVGSL
BraelkUSDA3254SDgb1      DLTPSVYRRLFRDHPEAQAMFRT-----EGSEPVKGSMLQLTIEAILDFA
BraelkUSDA3259SDgb3      DLTPSVYRRLFRDHPEAQAMFRT-----EGSEPVKGSMLQLTIEAILDFA
BraelkUSDA94SDgb3        DLTPFVYRRLFREHPETQAMFRT-----EGSEPVKGSMLQLTIEALLDFA
BraelkUSDA76SDgb3        DLTPFVYRRLFREHPETQAMFRT-----EGSEPVKGAMLQLTIEAILDFA
Braelk587SDgb3           -------------------MFRT-----EGSEPVKGAMLQLTIEAILDFA
BraelkWSM2783SDgb3       DLTPLIYRRLFEAHPETVAMFRT-----EGSELVKGSMLALTIEAILDFA
BraelkWSM1741SDgb2       DLTPLVYRRLFEAHPEARTMFRT-----EGGELVKGSMLALTINAILDFA
BrajapUSDA110SDgb2       DLTPLVYRRLFDEHPETRAMFRS-----QGSELVKGSMLALTIEAILDFA
BrajapUSDA122SDgb2       DLTPLVYRRLFDEHPETRTMFRS-----QGSDLVKGSMLALTIEAILDFA
BrajapUSDA4SDgb2         DLTPLVYKRLFDEHPETQAMFRS-----KGGDLVKGSMLALTIEAILDFA
BrajapWSM2793SDgb2       DLTPLVYQRLFDEHPETQAMFRS-----EGADLVKGSMLALTIEAILDFA
BrajapUSDA6-7488SDgb2    -------------------MFRS-----QGSEPVMGSMLALTIEAILDFA
BrajapUSDA6-8372SDgb1    DLTPLVYQRLFEQHPETRTMFRS-----QGSEPVMGSMLALTIEAILDFA
BrajapUSDA38SDgb2        DLTPLVYQRLFEQHPETRTMFRS-----QGSEPVMGSMLALTIEAILDFA
BrajapUSDA123SDgb2       DLTPLVYQRLFEQHPETQTMFRS-----QGSELVMGSMLALTIEAILDFA
BrajapUSDA124SDgb2       -------------------MFRT-----QGSELVKGSVLALTIEAILDFA
Brajapin8p8GCS           AILDEFYRHVLSEPQLAALIG---------DDMRRLKTAQSSHWARLFNG
Brajapis5GCS             AILDEFYRHVLSEPQLAALIG---------DDMRRLKTAQSSHWARLFNG
BraelkWSM2783GCS         GILDRFYDHVSRFAETARFFRSR-------EHMMHAKQMQMKHWSIILEG
RhilegWSM1481GCS2        AGLRDLFHRFQSFPDASRNFESE-------RQVERLHDLQSSHWDVLTDA
Rhileg248GCS2            AGLRDLFHRFQSFPDASRNFESE-------RQVERLHDLQSSHWDVLTDA
RhilegTOMGCS2            AGLRDLFHRFQSFPDASRNFESE-------RQVERLHDLQSSHWDVLTDA
RhilegUPM1131GCS         AGLRDLFHRFQSFPDASRNFESE-------RQVERLHDLQSSHWDVLTDA
RhilegVF39GCS2           AGLRDLFHRFQSFPDASRNFESE-------RQVERLHDLQSSHWDVLTDA
```

```
RhietlCIAT894GCS        AGLRDLFHRFQSFPDASRNFESE-------RQVERLHDLQSSHWDVLTDA
RhilegVh3GCS2           AGLRDLFHRFQSFPDASRNFESE-------RQIERLHDLQSSHWDVLTDA
Rhileg128C53GCS2        AGLRDLFHRFQSFPDASRNFESE-------RQVDRLHDLQSSHWDVLTDA
RhilegPs8GCS2           AGLRDLFHRFQSFPDASRNFESE-------RQVERLHDLQSSHWDVLTDA
RhilegGB30GCS2          AGLRDLFHRFQSFPDASRNFESE-------RQVERLHDLQSSHWDVLTDA
RhilegUPM1137GCS2       AGIRDLFHRFQSFPDASRNFESE-------RQVDRLHDLQSSHWDVLTDA
RhilegWSM1455GCS2       AGLRDLFHRFQSFPDASRNFESE-------RQVERLHDLQSSHWDVLTDA
Rhileg3841GCS2          AGLRDLFHRFQTFPDASRNFESE-------RQVERLHDLQSSHWDVLTDA
RhilegVc2GCS2           AGLRDLFHRFQSFPDASRNFESE-------RQIERLHDLQSSHWDVLTDA
Rhietl8C3GCS            AGLRDIFHRFQSFPDASRNFESE-------RQVERLHDLQSSHWDVLTDA
RhietlCIAT652GCS2       AGLRDIFHRFQSFPDASRNFESE-------RQVERLHDLQSSHWDVLTDA
RhietlCFN42GCS2         AGLRDLFHRFQSYPDAARNFESD-------RQVDRLQDLQSSHWDVLTDA
SinfreGR64GCS           LALRDLFQRLQANPDAVRHFDSD-------RQLDRLHDLQSSHWNVLTDA
SinfreHH103GCS          LALRDLFQRLQANPDAVRHFDSD-------RQLDRLHDLQSSHWNVLTDA
SinfreUSDA257GCS        LALRDLFHRLQTNPDAIRHFDND-------RQLDRLHDLQSSHWNVLTDA
Sinmel1021GCS           LALRALSHRLQASPDAARHFDSD-------RQIDRLHDLQSSHWNVLTDA
RhietlCIAT652GCS1       GALDKFYAKIARTPAVSSFFTDK-------SQVAHAKKRQQDHWANLAGG
RhietlCNPAF512GCS       GALDKFYAKIARPPAVSSFFTDK-------SQVAHAKKRQQDHWANLAGG
RhietlGR56GCS           GALDKFYAKIARTPAVSSFFTDR-------NHVGHAKKRQQDHWANLASG
Rhileg128C53GCS1        GALDKFYAKIAKTPAVSGFFADK-------SHVDHAKKRQQDHWANLAGG
RhilegUPM1137GCS1       GALDKFYAKIAKTPAVSGFFADK-------SHVDHAKKRQQDHWTNLAGG
RhilegGB30GCS1          GALDKFYAKIAKTPAVAGFFADK-------NHVGHAKKRQQDHWANLAGG
RhilegPs8GCS1           GALDKFYAKIAKTPAVAGFFADK-------NHVGHARKRQQDHWANLAGG
RhilegVc2GCS1           GALDKFYAKIAKTPAVAGFFADK-------NHVGHAKKRQQDHWANLAGG
RhilegVh3GCS1           GALDKFYAKIAKTPAVAGFFADK-------NHVGHAKKRQQDHWANLAGG
RhilegVF39GCS1          GALDKFYAKIAKTPAVAGFFADK-------NHVGHAKKRQQDHWANLAGG
Rhileg3841GCS1          GALDKFYAKIAKTPAVAGFFADK-------NHVGHAKKRQQDHWANLAGG
RhilegWSM1455GCS1       GALDKFYAKIAKTPAVAGFFADK-------NHVGHAKKRQQDHWANLAGG
RhilegWSM1481GCS1       GALDKFYAKIAKTPAVAGFFADK-------NHVGHAKKRQQDHWANLAGG
Rhileg248GCS1           GALDKFYAKIAKTPAVAGFFADK-------NHVGHAKKRQQDHWANLAGG
RhilegTOMGCS1           GALDKFYAKIAKTPAVAGFFADK-------NHVGHAKKRQQDHWANLAGG
RhietlCFN42GCS1         AALDKFYAKVARTPAVSGFFSDK-------NHVAHAKKRQQDHWGKLASG
AgrtumGCS               ASLDRFYTKVRAVPETAKFFSSE-------AHIHHAKSMQLKHWSRIASG
MyctubHbtC1             VVVEDFYVRVLADDQLSAFFSG-----------TNMSRLKGKQVEFFAAA
CupnecN1Hbt1            AVVDDFVGNVAADSRINAKFAT-----------ANIPRLKTRLVEQICAG
BrajapUSDA4Hbt2         AVVDHFSDAVVKNAVVGQKSKNPQLREWHTKNLKRLPGLKFMRTLWVCDV
BrajapWSM2793Hbt3       AVVDHFSDAVVKNAVVGQKSKNPQLREWHTKNLKRLPGLKFMRTLWVCDV
MyctubHbtC2             AIVSRFYAQVAEDEVLRRVYPED-----------DLAGAEERLRMFLEQY
BurphySTM815Hbt1        NIRELVYAFYDRVRADALIGPVF---------EKKLVGRWDEHLPKMCVF
MycaviHbtC3             ALLRRFYGRALDDEVLAEPFARLR------------ATGLDDHVPTMCDF
BraelkWSM1741Hbt1       TLVDDIVEAHMSNPTIKARFLPYR------EDPDRLAKVKQHLRNFLGAG
BacsubGCS               NIVDAFYKNLDHESSLMDIINDHS-------SVDRLKQTLKRHIQEMFAG

2/2-folding helix       -B----------]  [-C-]  [----------------------E------
3/3-folding helix       -----B--------][---C---]      [-----------E---------

                                   310       320       330       340       350
                                     |         |         |         |         |
BraelkUSDA3254Hbt       WSSVALMTGRYH---GTPMVKHMPLP-IDAAHFDRWLELFEATAAELCP-
BraelkUSDA3259Hbt       WSSVALMTGRYH---GTPMVKHMPLP-IDAAHFDRWLELFEATAAELCP-
BraelkUSDA76Hbt2        WSSVALMTGRYH---GTPMVKHMPLP-IDAAHFDRWLELFEATAAELCP-
Braelk587Hbt1           WSSVALMTGRYH---GTPMVKHMPLP-IDAAHFDRWLELFEATAAELCP-
BraelkUSDA94Hbt2        WSSVALMTGRYH---GTPMVKHMPLP-IDAAHFDRWLELFEATAAELCP-
BraelkWSM2783Hbt        WSSVALMTGRYH---GTPMAKHLPLP-VDAGHFDRWLELFEQTAQELCP-
BraelkWSM1741Hbt2       WSSVALMTGRYH---GTPMAKHLPLS-VDAAHFDRWLALFEATAREICP-
BrajapUSDA4Hbt1         WSSVVLMSGRYH---GSPMRAHLPLS-LVGDHFDRWLDLFEQTAREVCP-
BrajapWSM2793Hbt1       WSSVVLMSGRYH---GSPMRAHLPLS-LVGDHFDRWLDLFEQTAREVCP-
BrajapUSDA123Hbt1       WSSVVLMSGRYH---GSPMRAHVPLS-LVGDHFDRWLDLFEQTAREVCP-
BrajapUSDA6-8372Hbt1    WSSVVLMSGRYH---GSPMRAHVPLS-LVGDHFDRWLDLFEQTAREVCP-
BrajapUSDA6-7488Hbt1    WSSVVLMSGRYH---GSPMRAHVPLS-LVGDHFDRWLDLFEQTAREVCP-
BrajapUSDA38Hbt1        WSSVVLMSGRYH---GSPMRAHVPLS-LIGNHFDRWLDLFEQTAREVCP-
Brajapin8p8Hbt1         WSSVVLMSGRYH---GSPMRAHLPLS-LAGDHFDRWLDLFEQTAREVCP-
Brajapis5Hbt1           WSSVVLMSGRYH---GSPMRAHLPLS-LAGDHFDRWLDLFEQTAREVCP-
BrajapUSDA135Hbt1       WSSVVLMSGRYH---GSPMRAHLPLN-LVGDHFDRWLDLFEQTARKVCP-
BrajapUSDA122Hbt1       WSSVVLMSGRYH---GSPMRAHLPLG-LVGDHFDRWLDLFEQTARDVCP-
BrajapUSDA124Hbt1       WSSVVLMSGRYH---GSPMRAHLPLS-LVGGHFDRWLDLFEQTAREVCP-
BrajapWSM1743Hbt1       WSSVVLMSGRYH---GSPMRAHLPLS-LVGGHFDRWLDLFEQTAREVCP-
Brajap22Hbt1            WSSVVLMSGRYH---GSPMRAHLPLG-LAGDHFDRWLDLFEQTAREVCP-
```

```
AzodoeUFLA1-100Hbt1         WSSVMLGSGRYK---GNPFAVHTALP-LEPGLFQDWLGLWHKTTAELFE-
MeslotNZP2037Hbt2           WCSVILKTGDYH---GRPVPAHLKLGDVTEADFDIWLAMFSQTAAELFG-
MeslotUSDA3471Hbt1          WCSVILKSGDYH---GRPVPAHTRLRGVTEADFEIWQALFATTAAELFA-
RhietlCIAT652Hbt2           WSAVAFRSGAYG---GKPVQAHTGVADLTPDLFPKWLALFAATLDDVAPS
RhietlCNPAF512Hbt           WSAVAFRSGAYG---GKPVQAHTGVADLTPDLFPKWLALFAATLDDIAPS
RhietlBrasil5Hbt            WSAVAFRSGAYG---GKPVQAHTGVADMTPDLFPKWLALFAATLDDVAPS
RhietlCFN42Hbt2             WSAVTFRSGAYG---GKPVQAHTGVQNLTPDLFPKWLSLFAATLDDVAPS
Rhileg3841Hbt2              WSAVAFRSGAYG---GKPVQAHTGVQNLTPDLFPKWLSLFAATLDDIAPT
RhilegWSM1455Hbt2           WSAVAFRSGAYG---GKPVQAHTGVQNLTPDLFPKWLSLFAATLDDIAPT
RhilegVF39Hbt1              WSAVAFRSGAYG---GKPVQAHTGVQNLTPDLFPKWLSLFAATLDDIAPT
RhilegWSM1481Hbt2           WSAVAFRSGVYG---GKPVQAHTGVQNLTPDLFPKWLSLFAATLDDIAPT
RhilegUPM1131Hbt2           WSAVAFRSGAYG---GKPVQAHTGVQNLTPDLFPKWLSLFAATLDDIAPT
Rhileg248Hbt2               WSAVAFRSGAYG---GKPVQAHTGVQNLTPDLFPKWLSLFAATLDDIAPT
RhilegTOMHbt2               WSAVAFRSGAYG---GKPVQAHTGVQNLTPDLFPKWLSLFAATLDDIAPT
Rhileg128C53Hbt1            WSAVAFRSGAYG---GKPVQAHTHVANLTPDLFPKWLSLFAATLDDIAPS
RhilegPs8Hbt2               WSAVAFRSGAYG---GKPVQAHTHVANLTPDLFPKWLSLFAATLDDIAPT
RhilegGB30Hbt2              WSAVAFRSGAYG---GKPVQAHTHVANLTPDLFPKWLSLFAATLDDIAPS
RhilegUPM1137Hbt2           WSAVAFRSGAYG---GKPVQAHTHVANLTPDLFPKWLSLFAATLDGIAPS
RhilegVc2Hbt2               WSAVAFRSGAYG---GKPVQAHTHVANLTPDLFPKWLSLFAATLDDIAPS
RhilegVh3Hbt1               WSAVAFRSGAYG---GKPAQAHAHVANLTPDLFPKWLSLFAATLDDIAPS
Sinmel1021Hbt2              WSAVAFRSGAYG---GKPVQAHLGVANMSPELFPKWLGLFAATLDDIAPN
RhilupHPC_L_Hbt1            WAAVAFKNGGYG---GKPVQAHLGVKGMTAELFPQWLALFSATLDDIAP-
RhilupHPC_L_Hbt2            LGGPPVYVEKHG---HPMLRRRHFVAPIGPAERDEWLLCFRRAMEETIE-
AgrtumHbtC2                 LGGPPVYVEKHG---HPMLRRRHFVAPIGPAERDEWLLCFRRAMDETIE-
SinfreUSDA257Hbt            LGGPPIYVEKRG---HPMLRRRHFVAEIGSAERDEWLLCFTRALEETVS-
SinfreHH103Hbt              LGGPPIYVEKRG---HPMLRRRHFIAEIGPAERDEWLLCFTRALEETVA-
SinfreGR64Hbt               LGGPPIYVEKRG---HPMLRRRHFIAGIGPAERDEWLLCFTRALEEAVA-
Sinmel1021Hbt1              LGGPPIYVQKRG---HPMLRRRHFIAGIGPAERDEWLFCFTRALEETVS-
RhilegUPM1137Hbt1           LGGPPLYTDKRG---HPRLRSRHFVAEIGPVERDEWLLCFRRALDETIA-
Rhileg128C53Hbt2            LGGPPLYTDKRG---HPRLRSRHFVAEIGPVERDEWLLCFRRALDETIA-
RhilegVc2Hbt1               LGGPPLYTDKRG---HPRLRSRHFVAEIGPVERDEWLLCFRRALDETIA-
RhilegVh3Hbt2               LGGPPLYTDKRG---HPRLRSRHFVAEIGPVERDEWLLCFRRALDETIA-
RhilegGB30Hbt1              LGGPPLYTDKRG---HPRLRSRHFVAEIGPVERDEWLLCFRRALDETIA-
RhilegPs8Hbt1               LGGPPLYTDKRG---HPRLRSRHFVAEIGPVERDEWLLCFRRALDETIA-
Rhileg3841Hbt1              LGGPPLYTDKRG---HPRLRSRHFVAEIGPVERNEWLLCFRRALDETIS-
Rhileg248Hbt1               LGGPPLYTDKRG---HPRLRSRHFVAEIGPVERDEWLLCFRRALDETIS-
RhilegWSM1455Hbt1           LGGPPLYTDKRG---HPRLRSRHFVAEIGPVERDEWLLCFRRALDETVA-
RhilegWSM1481Hbt1           LGGPPLYTDKRG---HPRLRSRHFVAEIGPVERDEWLLCFRRALDETVA-
RhilegVF39Hbt2              LGGPPLYTDKRG---HPRLRSRHFVADIGPVERDEWLLCFRRALDETIS-
RhilegTOMHbt1               LGGPPLYTDKRG---HPRLRSRHFVAEIGPVERDEWLLCFRRALDETIS-
RhilegUPM1131Hbt1           LGGPPLYTDKRG---HPRLRSRHFVAEIGPVERDEWLLCFRRALDETIS-
RhietlKIM5Hbt               LGGPPLYTDKRG---HPRLRSRHFVAEIGPVERDEWLLCFRRALDETIA-
RhietlCFN42Hbt1             LGGPPLYTNKRG---HPRLRSRHFVADIGPVERDEWLLCFRRALDETIA-
Rhietl8C3Hbt                LGGPPLYTDKRG---HPRLRSRHFVAEIGPVERDEWLLCFRRALDETIA-
RhietlCIAT652Hbt1           LGGPPLYTDKRG---HPRLRSRHFVAEIGPVERDEWLLCFRRALDETIA-
RhietlCIAT894Hbt            LGGPPLYTDKRG---HPRLRSRHFVAEIGPVERDEWLLCFRRALDET---
RhietlIE4771Hbt             LGGPPLYTDKRG---HPRLRSRHFVAEIGPVERDEWLLCFRRALTRRLR-
AzodoeUFLA1-100Hbt2         LGGPKLYSAEKG---HPRLRQRHLGFAIGNAERDAWMHCMRGALAETVA-
Brajap22Hbt2                TGGPKLYSPEKG---HPRLRQRHMGFPIGGAERDAWLLCMRGALGETVA-
BrajapUSDA110Hbt            TGGPRLYSVEKG---HPRLRQRHIGFAIGDAERDAWLTCMRGALEETVA-
BrajapUSDA122Hbt2           TGGPRLYSVEKG---HPRLRQRHIGFAIGDAERDAWLTCMRGALGETVA-
BrajapUSDA124Hbt2           TGGPRLFTPEKG---HPRLRQRHIGFAIGDAERDAWMLCMRGAIEETVT-
BrajapWSM1743Hbt2           TGGPRLYTPEKG---HPRLRQRHFGFAIGDAERDAWLRCMRGALEETVT-
BrajapUSDA6-8372Hbt2        TGGPKLYTPEKG---HPRLRQRHIGFAIGDAERDAWLLCMRGAMEETVT-
BrajapUSDA6-7488Hbt2        TGGPKLYTPEKG---HPRLRQRHIGFAIGDAERDAWLLCMRGAMEETVT-
BrajapUSDA38Hbt2            TGGPKLYTPEKG---HPRLRQRHIGFAIGDAERDAWLFCMRGAMEETVV-
BrajapUSDA123Hbt2           TGGPKLYTPEKG---HPRLRQRHIGFAIGDAERDAWLLCMRGALEETIT-
BrajapWSM2793Hbt2           TGGPRLYTPEKG---HPRLRQRHIGFAIGDAERDAWMLCMRGAMEETVT-
BrajapUSDA135Hbt2           TGGPRLYTPEKG---HPRLRQRHIGFAIGDAERDAWMLCMRGALEETVT-
BrajapUSDA4Hbt3             TGGPRLYSVEKG---HPRLRQRHIGFAIGDAERNAWMVCMRGALEETVV-
Brajap22Hbt3                TGGPRLYTPEKG---HPRLRQRHFGFAIGDAERDAWMLCMRGAMEETIT-
Brajapin8p8Hbt2             TGGPKLYSPEKG---HPRLRQRHFGFAIGDAERDAWMLCMRGALAETIT-
Brajapis5Hbt2               TGGPKLYSPEKG---HPRLRQRHFGFAIGDAERDAWMLCMRGALAETIT-
CupnecHPC_L_Hbt             LGGPNHFIERFG---HPRLRARHLPFEIGISERDQWMRCMAMAMHDVGL-
CupnecJMP134Hbt             LGGPNHFIERFG---HPRLRARHMPFEIGISERDQWMRCMALAMQDVGL-
CupnecN1Hbt2                LGGPNHFIERFG---HPRLRARHMPFEIGVSERDQWMRCMALAMQDIGL-
BurphySTM815Hbt2            LGGPDHYISRFG---HPRLRARHLPFPIASVERDQWLRCMAWAMEDVGL-
BraelkUSDA76Hbt1            FGGPRFYTERYGG--YRRMVSQHIGKEIRPEQRALWAAYMVQSADDAGLP
```

Braelk587Hbt2	FGGPRFYTERYGG--YRRMVSQHIGKEIRPEQRALWAAYMVQSADDAGLP
BraelkUSDA94Hbt1	FGGPRFYTERYGG--YRRMVSQHIGKEIRPEQRALWATYMVQSADDAGLP
MescicCMG6Hbt	FGGPKTYSEQFGG--HREMVMHHLGKHLSEEQRRRWISLLADAADAVGLP
MescicWSM1271Hbt	FGGPKTYSEQFGG--HREMVMHHLGKHLTEEQRRRWISLLADAADAVGLP
MescicWSM4083Hbt	FGGPKTYGEQFGG--HREMVMHHLGKHLSEEQRRRWISLLADAADAVGLP
MeslotR7AHbt	LGGPKTYSEKFGG--HREMVMHHLGKHLTEEQRRRWINLLADAADEVGLP
MeslotMAFF303099Hbt	FGGPKTYSEKFGG--HREMVMHHLGKHLTEEQRRRWINLLADAADEVGLP
MeslotNZP2037Hbt1	FGGPKIYSEKFGG--HREMVMHHLGKHLTEEQRRRWINLLADAADEVGLP
MeslotR88bHbt	FGGPKTYSEKFGG--HREMVMHHLGKHLTEEQRRRWINLLADAADTVGLP
MeslotCJ3symHbt	FGGPKTYSEKFGG--HREMVMHHLGKHLTEEQRRRWINLLADAADTVGLP
MeslotUSDA3471Hbt2	FGGPKTYSEKFGG--HREMVMHHLGKHLTEAQRRRWINLLADAADEVGLP
BurphySTM815fHb	EQLEQLGGLVS------QIVNKHVALNILPEHYPIVGTCLLRAIREVLGP
CupnecHPC_L_fHb	DELEALAGALP------RIVQKHAALGVQPEHYPIVGGCLLQAIRDVLG-
CupnecN1fHb2	EDPSSLLAVLK------NIANKHASLGVRPEQYPIVGEHLLAAIKEVLG-
Sinmel1021fHb	ENPESLSAVLK------DIAHKHASIGVRPEQYPIVGEHLLASIKEVLG-
BacsubfHb	DQLGTIIPVVK------QIGHKHRSIGIKAEHYPIVGKYLLIAIKDVLG-
VitSDgb	ENLPAILPAVK------KIAVKHCQAGVAAAHYPIVGQELLGAIKEVLG-
EsccolfHb	ENLPALLPAVE------KIAQKHTSFQIKPEQYNIVGEHLLATLDEMFS-
RhilegUPM1137fHb	ESLAALGPAVE------RIAQKHIGYAILPDHYPHVATALLGAIEEVLG-
CupnecN1fHb1	DNAAALGPVLE------RIVHKHAAVGLTPAHYPIVGRHLLGAISAVLG-
CupnecJMP134fHb	DNANALAPVVE------RIVHKHAAVGLKPAHYPIVGRHLLGAISAVLG-
SaccerfHb	DDLSVLMDHVK------QIGHKHRALQIKPEHYPIVGEYLLKAIKEVLG-
BrajapUSDA110SDgb1	SNLDSILPAAS------ALAKRHVAYGAKAEHYPVVGATLLWTLEKGLG-
BrajapUSDA122SDgb1	SNLDSILPAAS------ALAKRHVAYGAKAEHYPVVGATLLWTLEKGLG-
Brajapin8p8SDgb	SNLESILPAAS------ALAKRHVDYGAKAEHYPVVGATLLWTLEKGLG-
Brajapis5SDgb	SNLESILPAAS------ALAKRHVDYGAKAEHYPVVGATLLWTLEKGLG-
BrajapUSDA135SDgb1	SNLESILPAAS------ALAKRHVAYGAKAEHYPVVGATLLWTLEKGLG-
BrajapWSM1743SDgb	SNLETILPAAS------ALAKRHVAYGATAEHYPVVGATLLWTLETGLG-
BrajapUSDA124SDgb1	SNLETILPAAS------ALAKRHVAYGAKAEHYPVVGATLLWTLEKGLG-
Brajap22SDgb	SNLDSILPAAS------ALAKRHVAYGAAPEHYPVVGATLLWTLEKGLG-
BrajapUSDA6-7488SDgb1	SKLETILPAAS------ALAKRHVAYGAKAEHYPVVGATLLWTLEKGLG-
BrajapUSDA6-8372SDgb2	SKLETILPAAS------ALAKRHVAYGAKAEHYPVVGATLLWTLEKGLG-
BrajapUSDA123SDgb1	SKLETILPAAS------ALAKRHVAYGAKAEHYPVVGATLLWTLEKGLG-
BrajapUSDA38SDgb1	SKLETILPAAS------TLAKRHVAYGAKAEHYPVVGATLLWTLEKGIG-
BrajapUSDA4SDgb1	SNLETVLPAAS------ALAKRHVAYGATAEHYPVVGATLLWTLEKGLG-
BrajapWSM2793SDgb1	SNLETILPAAS------ALAKRHVAYGATAEHYPVVGASLLWTLEKGLG-
BraelkUSDA3254SDgb4	SNLDTILPAAS------ALAKRHVGYGAKAEHYPVVGGALLWTLEKGLG-
BraelkUSDA3259SDgb1	SNLDTILPAAS------ALAKRHVGYGAKAEHYPVVGGALLWTLEKGLG-
BraelkUSDA94SDgb1	SNLDTILPAAS------ALAKRHVGYGAKPEHYPVVGGALLWTLEKGLG-
Braelk587SDgb1	SNLDTILPAAS------ALAKRHVNYGARPEHYPVVGGALLWTLEKGLG-
BraelkUSDA76SDgb1	SNLDTILPAAS------ALAKRHVNYGARPEHYPVVGGALLWTLEKGLG-
BraelkWSM2783SDgb1	SNLQSVLPAAS------ALAKRHVSYGTKAEHYPVVGDALLWTLQKGLG-
AzodoeUFLA1-100SDgb	TKLEVIVPAAQ------TLARRHVAYGVRPEHYAPVGAALLWTLEQGLG-
BraelkWSM1741SDgb1	DDLPSLIPTLE------ILGRNHIRYGVEDRHYDTVGAALLWTLEQGLK-
BraelkWSM2783SDgb2	PAQDQLSPAIH------DLALRHVEYGVKLEDYDAMREALLWTLEQALG-
BraelkUSDA3254SDgb2	DERPIFQSLIT------LSGHKHARLGVQLSHYVAMGEALMWSLERKLG-
BraelkUSDA3259SDgb2	DERPIFQSLIT------LSGHKHARLGVQLSHYVAMGEALMWSLERKLG-
BraelkUSDA3254SDgb3	DERPMFQSLIA------ISGHKHAILGVQPSHFVAMGEALMWSFERKFG-
BraelkUSDA3259SDgb4	DERPMFQSLIA------ISGHKHAILGVQPSHFVAMGEALMWSFERKFG-
Braelk587SDgb2	DERPMFQSLIT------LSGHKHARLGVQPSHFVAMGEALMWSFERKFG-
BraelkUSDA76SDgb2	DERPMFQSLIT------LSGHKHARLGVQPSHFVAMGEALMWSFERKFG-
BraelkUSDA94SDgb2	DERPMFQSLIT------LSGHKHARLGVQPSHFVAMGEALMWSFERKFG-
BrajapUSDA135SDgb2	DETTGLLSGAD------ILGRNHLRYGVRPEHYPVVAEALIWSFARGLG-
BraelkUSDA3254SDgb1	GERRGHFRLIE------SEVFSHDAYGTPRELFVAFFAVIADSLREILG-
BraelkUSDA3259SDgb3	GERRGHFRLIE------SEVFSHDAYGTPRELFVAFFAVIADSLREILG-
BraelkUSDA94SDgb3	GERRGHFRLIE------SEVFSHDAYGTPRELFVAFFAVIADSLREILG-
BraelkUSDA76SDgb3	GERRGHFRLIE------SEVFSHDAYGTPRELFVAFFAMIADSLRDILG-
Braelk587SDgb3	GERRGHFRLIE------SEVFSHDAYGTPRELFVAFFAMIADSLRDILG-
BraelkWSM2783SDgb3	GERRGNFRMIE------CEVASHDAYGTPRDLFVAFFAVIAGTLQEILG-
BraelkWSM1741SDgb2	GQRTGHFRLIA------SEVSSHDSYGTPRELFVAFFGIIAQTLREIVG-
BrajapUSDA110SDgb2	GTRSGHFRLIA------CEVVSHDAYGTPRELFIAFFAVIRDTLRDLLG-
BrajapUSDA122SDgb2	GTRSGHFRLIA------CEIVSHDAYGTPRELFIAFFAIIRDTLRDLLG-
BrajapUSDA4SDgb2	GMRHGHFRLIA------CELASHDAYGTSRQLFTAFFTIIRDTLRDLLG-
BrajapWSM2793SDgb2	GMRHGHFRLIA------CEVASHDAYGTSPQLFTAFFTIIRDTLRDLLG-
BrajapUSDA6-7488SDgb2	GERRGHFRLIA------CEVASHDGYGTPRELFIAFFVVIRDALRDLLG-
BrajapUSDA6-8372SDgb1	GERRGHFRLIA------CEVASHDGYGTPRELFIAFFVVIRDALRDLLG-
BrajapUSDA38SDgb2	GERRGHFRLIA------CEFASHDGYGTPRELFIAFFAVIRDTLRDLLG-
BrajapUSDA123SDgb2	GERRGHFRLIA------CEVASHDGYGTPRELFIAFFAVIRNALRDLLG-

```
BrajapUSDA124SDgb2      GERRGHFRLIA------CEVASHDAYGTSRALFIAFFAIVRDTLRDLLG-
Brajapin8p8GCS          RFDHDYMRGVR------MIGLMHNKIGLEPRWYIGGYNFVLCRLLALAVR
Brajapis5GCS            RFDHDYMRGVR------MIGLMHNKIGLEPRWYIGGYNFVLCRLLALAVR
BraelkWSM2783GCS        RFDADYEASVA------RIGEIHNKLGLEPGWYIGGYNALLSDLVSEVAI
RhilegWSM1481GCS2       RFDSLYAERVK------VLSDTESKMGLDPRWHVAGHGVMLEHVVSGLAE
Rhileg248GCS2           RFDSLYAERVK------VLSDTESKMGLDPRWHVAGHGVMLEHVVSGLAE
RhilegTOMGCS2           RFDSLYAERVK------VLSDTESKMGLDPRWHVAGHGVMLEHVVSGLAE
RhilegUPM1131GCS        RFDSLYAERVK------VLSDTESKMGLDPRWHVAGHGVMLEHVVSGLAE
RhilegVF39GCS2          RFDSLYAERVK------VLSDTESKMGLDPRWHVAGHGVMLEHVVSGLAE
RhietlCIAT894GCS        RFDSLYAERVK------VLSDTESKIGLDPRWHVAGHGVMLEHVVSGLAE
RhilegVh3GCS2           RFDSLYAERVK------VLSDTESKMGLDPRWHVAGHGVMLEHVVSGLAE
Rhileg128C53GCS2        RFDSLYAERVK------VLSDTESKMGLDPRWHVAGHAVMLEHVVSGLAE
RhilegPs8GCS2           RFDSLYAERVK------VLSDTESKMGLDPRWHVAGHGVMLEHVVSGLAE
RhilegGB30GCS2          RFDSLYAERVK------VLSDTESKMGLDPRWHVAGHGVMLEHVVSGLAE
RhilegUPM1137GCS2       RFDSLYAERVK------VLSDTESKMGLDPRWHVAGHAVMLEHVVSGLAE
RhilegWSM1455GCS2       RFDSLYAERVK------VLSDTESKMGLDPRWHVAGHGVMLEHVVSGLAE
Rhileg3841GCS2          RFDSLYAERVK------VLSDTESKMGLDPRWHVAGHGVMLEHVVSGLAE
RhilegVc2GCS2           RFDSLYAERVK------VLSDTESKMGLDPRWHVAGHGVMLEHVVSGLAD
Rhietl8C3GCS            RFDSLYAERVK------VLSDTESKMGLDPRWHVAGHGVMLEHVLSGLAA
RhietlCIAT652GCS2       RFDSLYAERVK------VLSDTESKMGLDPRWHVAGHGVMLEHVLSGLAA
RhietlCFN42GCS2         RFDSLYAERVK------VLSDTESKMGLDPRWHVAGHGVMLEHVLSGLAE
SinfreGR64GCS           RFDGLYAERVK------VLADTEARMGLDPRWQLASHAVVLEHLIVGAIE
SinfreHH103GCS          RFDALYAERVK------VLADTEARMGLDPRWQLASHAVVLEHLIVGAIG
SinfreUSDA257GCS        RFDGLYAERVK------VLADTEARMGLDPRWQLASHAVVLEHLIVGAIE
Sinmel1021GCS           RFDGLYAERVK------VLADTESRMGLDPRWQIASHAIVLEHLILGAIE
RhietlCIAT652GCS1       SFDESYVDGVT------AVGRTHARIGLEPRWYIGGYALVMSELVKGIME
RhietlCNPAF512GCS       SFDESYVDGVT------AVGRTHARIGLEPRWYIGGYALVMSELVKGIME
RhietlGR56GCS           TFDERYVEGVT------AVGRTHARIGLEPRWYIGSYAIVMSELVKGIME
Rhileg128C53GCS1        AFDESYVNGVT------AVGRTHARIGLEPRWYIGGYAIVMSELVKGIME
RhilegUPM1137GCS1       AFDESYVNGVT------AVGRTHARIGLEPRWYIGGYAIVMSELVKGIME
RhilegGB30GCS1          AFDESYVNGVT------AVGRTHARIGLEPRWYIGGYAIVMSELVKGIME
RhilegPs8GCS1           AFDESYVNGVT------AVGRTHARIGLEPRWYIGGYAIVMSELVKGIME
RhilegVc2GCS1           AFDESYVNGVT------AVGRTHARIGLEPRWYIGGYAIVMSELVKGIME
RhilegVh3GCS1           AFDESYVNGVT------AVGRTHARIGLEPRWYIGGYAIVMSELVKGIME
RhilegVF39GCS1          AFDESYVNGVT------AVGRTHARIGLEPRWYIGGYAIVMSELVKGIME
Rhileg3841GCS1          AFDESYVNGVT------AVGRTHARIGLEPRWYIGGYAIVMSELVKGIME
RhilegWSM1455GCS1       AFDESYVNGVT------AVGRTHARIGLEPRWYIGGYAIVMSELVKGIME
RhilegWSM1481GCS1       AFDESYVNGVT------AVGRTHARIGLEPRWYIGGYAIVMSELVKGIME
Rhileg248GCS1           AFDESYVDGVT------AVGRTHARIGLEPRWYIGGYAIVMSELVKGIME
RhilegTOMGCS1           AFDESYVNGVT------AVGRTHARIGLEPRWYIGGYTIVMSELVKGIME
RhietlCFN42GCS1         AFDETYVDGVT------AVGRTHARIGLEPRWYIGGYALVMSELVKGIME
AgrtumGCS               TFNEDYTNAVT------AIGRTHARLGLEPRWYIGGYALMLDGIVKAVIE
MyctubHbtC1             LGGPEPYTGA-------PMKQVHQGRGITMHHFSLVAGHLADALTAAGVP
CupnecN1Hbt1            AGGPCTYSGR-------DMRTAHAGMGITNGEFDALVEDLVKSLAKFKVS
BrajapUSDA4Hbt2         SGGPFQFKATRPGTTPLGLEEAHRALRISPAEFDEVAAELGRTLDFAKVP
BrajapWSM2793Hbt3       SGGPFQFKATRPGTTPLGLEEAHRGLRISPAEFDEVAAELGRTLDFAKVP
MyctubHbtC2             WGGPRTYSEQRG---HPRLRMRHAPFRISLIERDAWLRCMHTAVASIDSE
BurphySTM815Hbt1        WGSLVLGAKQYRG----NVQQAHPLEGLEPRHFSRWLYLFLDTVESRYEP
MycaviHbtC3             WETVLFRAGRYRGSALQAHRDIHRRAPLSDRHFRRWLTLWHNTVDEMYRG
BraelkWSM1741Hbt1       SGGPEQYSGRS-------MCDAHRGMNVNAQEYMAAIDDIMSTLEKHNID
BacsubGCS               VIDDEFIEKRNR------IASIHLRIGLLPKWYMGAFQELLLSMIDIYEA

2/2-folding helix       -------------][----F----]  [--------G---------]   [
3/3-folding helix       -] [------------F-----------]   [----------G-------

                                  360       370       380       390       400
                                   |         |         |         |         |
BraelkUSDA3254Hbt       --PDAAAHFIDRARRIASSLEMGVASGQGVMLAVGERYRRSEAGAVQ---
BraelkUSDA3259Hbt       --PDAAAHFIDRARRIASSLEMGVASGQGVMLAVGERYRRSEAGAVQ---
BraelkUSDA76Hbt2        --PDAAAHFIDRARRIASSLEMGVASGQGVMLGVGERYRRSEAGAVQ---
Braelk587Hbt1           --PEAAAHFIDRARRIASSLEMGVASGQGVMLGVGERYRRSEAGAVQ---
BraelkUSDA94Hbt2        --PEAAAHFIDRARRIASSLEMGVASGQGVMLAVGERYRRSEAGAVQ---
BraelkWSM2783Hbt        --PEAAAHLIERAQRIAASLELGIAGAQGVMLGKGERFRRGEDANA----
BraelkWSM1741Hbt2       --PEAEAHFVERARRIAASLELGIAGVNGVMLANGERFRRNQAGAV----
BrajapUSDA4Hbt1         --PPAAALFIDKARRIADSFEMASATVAGSIAAPRHVLRS----------
BrajapWSM2793Hbt1       --PPAASLFIDKARRIADSFEMASATVSGSIAAPRHVLRS----------
BrajapUSDA123Hbt1       --PPAAALFIDKARRIADSFEMASATIAGRIAAPRHVLRS----------
BrajapUSDA6-8372Hbt1    --PPAAALFIDKARRIADSFEMASATIAGRIAAPRHVLRS----------
```

```
BrajapUSDA6-7488Hbt1       --PPAAALFIDKARRIADSFEMASATIAGRIAAPRHVLRS----------
BrajapUSDA38Hbt1           --PPAAALFIDKARRIADSFEMASATIAGRIAAPRHVLRS----------
Brajapin8p8Hbt1            --PTAAALFIDKARRIADSFEMASATVAGRIASPRHVLRS----------
Brajapis5Hbt1              --PTAAALFIDKARRIADSFEMASATVAGRIASPRHVLRS----------
BrajapUSDA135Hbt1          --PAAAALFIDKARRIADSFEMASATVAGRIASPRHVLRS----------
BrajapUSDA122Hbt1          --PAAAALFIDKARRIADSFEMASATVAGRIASPRHVLRS----------
BrajapUSDA124Hbt1          --PPAAALFIDKARRIADSFEMASATVAGRIASPRHVLRS----------
BrajapWSM1743Hbt1          --PAAAALFIDKARRIADSFEMASATVAGRIASPRHVLRS----------
Brajap22Hbt1               --PPAASLFIDKARRIADSFEMASATVAGSIAAPRHVLRP----------
AzodoeUFLA1-100Hbt1        --PEVAAQFDARAERIADSLKAGLFFNPSRPAR-----------------
MeslotNZP2037Hbt2          --PEIAAVFVDRAERIATSLKLAMFFRLGDRA------------------
MeslotUSDA3471Hbt1         --PDTAAVFVARAERIAKSLRLAMFFRLGPPDVGQCHPGDGAL-------
RhietlCIAT652Hbt2          --PEAKAWFMATAERIAKSLTLSLFYNPALDDPAKKAG------------
RhietlCNPAF512Hbt          --PEAKAWFMATAERIAKSLTLSLFYNPALDDPAKKAG------------
RhietlBrasil5Hbt           --PEAKAWFMAPAERIAKSLTLSLFYNPALDGPAKKAG------------
RhietlCFN42Hbt2            --PEAKVWFMATAERIAKSLTLSLFYNPALDDPAKKVG------------
Rhileg3841Hbt2             --PEAKAWFMATAERIAKSLILSLFYNPALDDPARKPA------------
RhilegWSM1455Hbt2          --PEAKAWFMATAERIAKSLILSLFYNPALDDPARKPA------------
RhilegVF39Hbt1             --PEAKAWFMATAERIAKSLILSLFYNPALDDPARKPA------------
RhilegWSM1481Hbt2          --PEAKAWFMATAERIAKSLILSLFYNPALDDPARKPA------------
RhilegUPM1131Hbt2          --PEAKAWFMATAERIAKSLTLSLFYNPALDDPARKLA------------
Rhileg248Hbt2              --PEAKAWFMATAERIAKSLTLSLFYNPALDDPARKLA------------
RhilegTOMHbt2              --PEAKAWFMATAERIAKSLTLSLFYNPALDDPARKLA------------
Rhileg128C53Hbt1           --PEAKAWFMATAERIAKSLVLSLFYNPALDDPKRKPA------------
RhilegPs8Hbt2              --PEAKAWFMATAERIAKSLVLSLFYNPALDDPTRKPA------------
RhilegGB30Hbt2             --PEAKAWFMTTAERIAKSLTLSLFYNPALDDPKRKPA------------
RhilegUPM1137Hbt2          --PEAKAWFMATAERIAKSLVLSLFYNPALDDPKRKPA------------
RhilegVc2Hbt2              --PEAKAWFMATAERIAKSLVLSLFYNPALDDPTRKPA------------
RhilegVh3Hbt1              --PEAKAWFMATAERIAKSLVLSLFYNPALDDPARKPA------------
Sinmel1021Hbt2             --EEAKAWFMATAERIARSLTLSLFYNPALDDPALKRS------------
RhilupHPC_L_Hbt1           ------------AERRTTGLWKPLNASPEA--------------------
RhilupHPC_L_Hbt2           -NPKLREIIWPPIERLAFHMQNREADSQ----------------------
AgrtumHbtC2                -NPKLREIIWTPVERLAFHMQNQEADSQ----------------------
SinfreUSDA257Hbt           -HPQLRQVILEPITRLAHHMQNKE--------------------------
SinfreHH103Hbt             -HPQLRQVILEPITRLAHHMQNKE--------------------------
SinfreGR64Hbt              -HPQLREIILEPITRLAHHMQNKE--------------------------
Sinmel1021Hbt1             -HPKLREIILEPITRLAHHMQNKE--------------------------
RhilegUPM1137Hbt1          -SQALRDLIWAPVERLAYHMQNKAPDHKEQP-------------------
Rhileg128C53Hbt2           -SQALRDLIWAPVERLAYHMQNRAPDHKEQP-------------------
RhilegVc2Hbt1              -SQALRDLIWPPVERLAYHMQNKASDNKEQP-------------------
RhilegVh3Hbt2              -SQALRDLIWPPVERLAYHMQNKASDNKEQP-------------------
RhilegGB30Hbt1             -SQALRDLIWPPVERLAYHMQNKASDNKEQP-------------------
RhilegPs8Hbt1              -SQALRDLIWAPVERLAYHMQNKE--------------------------
Rhileg3841Hbt1             -SQELRDLIWAPVERLAYHMQNKAPDDKEQP-------------------
Rhileg248Hbt1              -SQALRDLIWAPVERLAYHMQNKAPDNKEQP-------------------
RhilegWSM1455Hbt1          -SQALRDLIWAPVERLAYHMQNKATDNKEQS-------------------
RhilegWSM1481Hbt1          -SQALRDLIWAPVERLAYHMQNKATDNKEQS-------------------
RhilegVF39Hbt2             -SQALRDLIWAPVERLAYHMQNKATDNKERS-------------------
RhilegTOMHbt1              -SQALRDLIWAPVERLAYHMQNKATDNKEQS-------------------
RhilegUPM1131Hbt1          -SQALRDLIWAPVERLAYHMQNKAPDNREQP-------------------
RhietlKIM5Hbt              -TQALRDLIWAPVERLAYHMQNKEQP------------------------
RhietlCFN42Hbt1            -SQALRDLIWAPVERLAYHMQNKAPDEEQP--------------------
Rhietl8C3Hbt               -SQALRDLIWAPVERLAYHMQNKAPDTEEQP-------------------
RhietlCIAT652Hbt1          -SQALRDLIWAPVERLAYHMQNKAPDTEEQP-------------------
RhietlCIAT894Hbt           --------------------------------------------------
RhietlIE4771Hbt            -ASTARSHLG----------------------------------------
AzodoeUFLA1-100Hbt2        -DAEARQSIDDAMTKLADWMRNTAGNPHDTGHGHRP--------------
Brajap22Hbt2               -DEAARGELDAALTKLADWMRNQAGNPHDARGRHG---------------
BrajapUSDA110Hbt           -DAAARQDLDKALSGLADWMRNR---------------------------
BrajapUSDA122Hbt2          -DAAARQDLDKALSGLADWMRNR---------------------------
BrajapUSDA124Hbt2          -DSAARKDLDSAISGLADWMRNRT--------------------------
BrajapWSM1743Hbt2          -DSAARRDLDNALSGLADWMRNRP--------------------------
BrajapUSDA6-8372Hbt2       -DSAARQDLDRAISGLADWMRNRS--------------------------
BrajapUSDA6-7488Hbt2       -DSAARQDLDRAISGLADWMRNRS--------------------------
BrajapUSDA38Hbt2           -DAAARQDLDRAISGLADWMRNRS--------------------------
BrajapUSDA123Hbt2          -DAAARQDLDKAISGLADWMRNRS--------------------------
BrajapWSM2793Hbt2          -DTAARLELDKAISGLADWMRNRQ--------------------------
BrajapUSDA135Hbt2          -DSAARQDLDAALSGLADWMRNRN--------------------------
```

BrajapUSDA4Hbt3	-DTIARQDLDRALSGLADWMRNRQ--------------------------
Brajap22Hbt3	-DAAARQELDKALSGLADWMRNRQ--------------------------
Brajapin8p8Hbt2	-DAAARQHLDRTMSGLADWMRNWP--------------------------
Brajapis5Hbt2	-DAAARQHLDRTMSGLADWMRNWP--------------------------
CupnecHPC_L_Hbt	-SEDLQMRLLQALFQTADWMRNVAR-------------------------
CupnecJMP134Hbt	-SEDLQMRLMQAFFQTADWMRNVAR-------------------------
CupnecN1Hbt2	-SEDLQLRLMQAFFQTADWMRNVAR-------------------------
BurphySTM815Hbt2	-PEPLRERLLHSFFDTADWMINQHSAVTKR--------------------
BraelkUSDA76Hbt1	SDPEFRAAFVAYIEWGSRIAVENSGAGATPPPN-MPVPRWWWVCNATPAA
Braelk587Hbt2	SDPEFRAAFVAYIEWGSRIAVENSGAGATPPPN-MPVPRWWWVCNATPAA
BraelkUSDA94Hbt1	TDPEFRAAFVAYIEWGSRIAVENSGAGATPPPN-MPVPRWWWVCNATPAA
MescicCMG6Hbt	DDPEFRSAFMGYVEWGSRLAKMNSNLGETCDPQTEPMPAWGWGVPGGPYQ
MescicWSM1271Hbt	DDPEFRSAFMGYVEWGSRLAKMNSNLGETCDPQTEPMPAWGWGVPGGPYQ
MescicWSM4083Hbt	DDPEFRSAFMGYVEWGSRLAKMNSNLGQTCDPQTEPMPAWGWGVPGGPYR
MeslotR7AHbt	DDPEFRSAFMGYVEWGSRLAKMNSNLGETCDPETEPMPAWGWGVPGGPYT
MeslotMAFF303099Hbt	DDPEFRSAFMGYVEWGSRLAKMNSNLGETCDPETEPMPAWGWGVPGGPYT
MeslotNZP2037Hbt1	DDPEFRSAFMGYVEWGSRLAKMNSNLGQTCDPETEPMPAWGWGVPGGPYT
MeslotR88bHbt	DDPEFRSAFMGYVEWGSRLAKMNSNLGETCDPETEPMPAWGWGVPGGPYT
MeslotCJ3symHbt	DDPEFRSAFMGYVEWGSRLAKMNSNLGETCDPKTEPMPAWGWGVPGGPYT
MeslotUSDA3471Hbt2	DDPEFRSAFTGYVEWGSRLAKMNSNLGETCDPQTEPMPAWGWGVPGGPYT
BurphySTM815fHb	EIATDAVIEAWGAAYGQLADLLIGLEEKVYVEKETSKGGWRGTR------
CupnecHPC_L_fHb	EAATDEIIGAWGEAYGVLAKILIDAEEAVYRDNAAQPGGWRGTR------
CupnecN1fHb2	DAATDDIISAWAQAYGNLADVLMGMESELYERSAEQPGGWKGWR------
Sinmel1021fHb	DAATDEIISAWAQAYGNLADILAGMESELYGRSEERAGGWAGWR------
BacsubfHb	DAATPDIMQAWEKAYGVIADAFIGIEKEMYEQAEEQAGGWKEYK------
VitSDgb	DAATDDILDAWGKAYGVIADVFIQVEADLYAQAVE---------------
EsccolfHb	PGQ--EVLDAWGKAYGVLANVFINREAEIYNENASKAGGWEGTR------
RhilegUPM1137fHb	GAATPDVLTAWGEAYWFLADILKGREAAIRDDLLSKAGGWTGWR------
CupnecN1fHb1	EAATPPLLAAWDEAYWLLAGELIAAEARLYQRTGVAAGELTPVR------
CupnecJMP134fHb	EAATPDLIAAWDEAYWLLAGELIAAEARLYQSTGMAAGERIAVR------
SaccerfHb	DAATPEIINAWGEAYQAIADIFITVEKKMYEEAL-----WPGWK------
BrajapUSDA110SDgb1	EAWTPELATAWTDAYGVLSGYMISEAYGAQAQAAE---------------
BrajapUSDA122SDgb1	EAWTPELATAWTDAYGVLSGYMISEAYGAQAQAAE---------------
Brajapin8p8SDgb	EAWTPELAKAWTDAYGVLSGYMISEAYGAQAQAAE---------------
Brajapis5SDgb	EAWTPELAKAWTDAYGVLSGYMISEAYGAQAQAAE---------------
BrajapUSDA135SDgb1	EAWTPELAKAWTDAYGVLSGYMISEAYGAQAQAAE---------------
BrajapWSM1743SDgb	EAWTPELATAWTDAYGVLSGYMISEAYGAQAQAAE---------------
BrajapUSDA124SDgb1	EAWTPELAAAWTDAYGVLSGYMMSEAYGAQAQAAE---------------
Brajap22SDgb	EAWTPELAKAWTDAYGVLSGYMMSEAYGAQPQAAE---------------
BrajapUSDA6-7488SDgb1	EAWTPELATAWTDAYGVLSGYMISEAYGAQAQAAE---------------
BrajapUSDA6-8372SDgb2	EAWTPELATAWTDAYGVLSGYMISEAYGAQAQAAE---------------
BrajapUSDA123SDgb1	EAWTPELAAAWTDAYGVLSGYMISEAYGAQAQAAE---------------
BrajapUSDA38SDgb1	EAWTPELATAWTDAYGVLSSYMISEAYGAQTQAAE---------------
BrajapUSDA4SDgb1	EAWTPELAKAWTDAYGVLSGFMMSEAYGAQAQAAE---------------
BrajapWSM2793SDgb1	DAWTPELAKAWTDAYGVLSGYMMSEAYGAQAQAAE---------------
BraelkUSDA3254SDgb4	EAWTPDVAAAWTAAYGTLSGYMISEAYGPVQPVE----------------
BraelkUSDA3259SDgb1	EAWTPDVAAAWTAAYGTLSGYMISEAYGPVQPVE----------------
BraelkUSDA94SDgb1	EAWTPDVAAAWTAAYGTLSGYMISEAYGSAQPAE----------------
Braelk587SDgb1	PAWTPDVAAAWTAAYGTLSGYMISEAYGGPRAAE----------------
BraelkUSDA76SDgb1	PAWTPDVAAAWTAAYGTLSGYMISEAYGGPRAAE----------------
BraelkWSM2783SDgb1	EAWTPEVAAAWTAAYGTLSGYMISEAYGSPQAAE----------------
AzodoeUFLA1-100SDgb	PDFTPETKAAWAEAYTLLSSVMIEAAADAAPVA-----------------
BraelkWSM1741SDgb1	EAWTPAAKSAWVVAYSTVSGVMRDAAAASRENATAQAV------------
BraelkWSM2783SDgb2	EDLTPAVREAWTVCYDELAGEMKAAAGV----------------------
BraelkUSDA3254SDgb2	ASFTQELQEAWRTLYATAQTEMLRSAAKT---------------------
BraelkUSDA3259SDgb2	ASFTQELQEAWRTLYATAQTEMLRSAAKT---------------------
BraelkUSDA3254SDgb3	ASFTPELRESWHTLYATAQNEMLRATGRHSSF------------------
BraelkUSDA3259SDgb4	ASFTPELRESWHTLYATAQNEMLRATGRHSSF------------------
Braelk587SDgb2	AFFTPELRDSWRALYATAQNEMLRAAGRPSSF------------------
BraelkUSDA76SDgb2	AFFTPELRDSWRALYATAQNEMLRAAGRPSSF------------------
BraelkUSDA94SDgb2	AFFTPELRDSWRTLYATAQNEMLRAAGRPSSF------------------
BrajapUSDA135SDgb2	AHWTDEVEQAWRKVYGIIAQRMIATDSSPPPSLSSPITSPPRRA------
BraelkUSDA3254SDgb1	EQWTAEIDAAWHKLLGDIEAIVLQQKHLVDERP-----------------
BraelkUSDA3259SDgb3	EQWTAEIDAAWHKLLGDIEAIVLQQKHLVDERP-----------------
BraelkUSDA94SDgb3	EQWTAEIDAAWHKLLGDIEAIVLQQKHLVDGRP-----------------
BraelkUSDA76SDgb3	EQWTAEIDAAWHTLLGDIEAIVLQQKHLVDERP-----------------
Braelk587SDgb3	EQWTAEIDAAWHTLLGDIEAIVLQQKHLVDERP-----------------
BraelkWSM2783SDgb3	VEWTAEIDAAWQKLLREIESMVIPDVA-----------------------

```
BraelkWSM1741SDgb2        PDWSDEIDAAWQELLGEIEGLVAAQ-------------------------
BrajapUSDA110SDgb2        DAWSAEIAQAWDTLLTDIEAYVTVPA------------------------
BrajapUSDA122SDgb2        DAWSAEIAQAWDTLLTDIEAYVAVPA------------------------
BrajapUSDA4SDgb2          DEWSIEIAQAWDKLLVDIDALAGSTA------------------------
BrajapWSM2793SDgb2        DEWSIEIAQAWDTLLVDIDALAGTTA------------------------
BrajapUSDA6-7488SDgb2     DEWSPEIAQAWDQLLVEIDAFATIPA------------------------
BrajapUSDA6-8372SDgb1     DEWSPEIAQAWDQLLVEIDAFATIPA------------------------
BrajapUSDA38SDgb2         DEWSPEIAQAWDQLLVEIDAFATIPA------------------------
BrajapUSDA123SDgb2        DEWSPEIAQAWDQLLVEIDAFATIPA------------------------
BrajapUSDA124SDgb2        DEWTPDMALAWDALLVEIDTYAGIPA------------------------
Brajapin8p8GCS            KY---------RWMPTRLASVLTALNTAVMLDIDIAISAYQEAMFAEKQK
Brajapis5GCS              KY---------RWMPTRLASVLTALNTAVMLDIDIAISAYQEAMFAEKQK
BraelkWSM2783GCS          RMPTR---MLSRGSAARQAAMQRAIVNAALLDMDIALAVYTEAERRDRR-
RhilegWSM1481GCS2         EIAGRPLLPSAKRRAREISDLMTAIIRIVMVDVEIAVSLRFNALRAAQSR
Rhileg248GCS2             QIAGRPLLPSAKRRTREISDLMTAIIRIVMVDVEIAVSLRFNALRAAQSR
RhilegTOMGCS2             EIAGRPLLPSAKRRTREISDLMTAIIRIVMVDVEIAVSLRFNALRAAQSR
RhilegUPM1131GCS          EIAGRPLLPSAKRRTREISDLMTAIIRIVMVDVEIAVSLRFNALRAAQSR
RhilegVF39GCS2            EIAGRPLLPSAKRRAREISDLMTAIIRIVMVDVEIAVSLRFNALRAGEQR
RhietlCIAT894GCS          EIAGRPLLPSAKRRAREISDLMTAIIRIVMVDVEIAVSLRFNAQRAGEQR
RhilegVh3GCS2             QIAGRPLLPSAKRRTREISDLMTAIIRIVMVDVEIAVSLRFNALRAGEQR
Rhileg128C53GCS2          EIAGRPLLPSAKRRTREISDLMTAIIRIVMVDVEIAVSLRFNALRAGEQR
RhilegPs8GCS2             QIAGRPLLPSAKRRTREISDLMTAIIRIVMVDVEIAVSLRFNALRAGEQR
RhilegGB30GCS2            QIAGRPLLPSAKRRTREISDLMTAIIRIVMVDVEIAVSLRFNALRAGEQR
RhilegUPM1137GCS2         EIAGRPLLPSAKRRTREISDLMTAIIRIVMVDVEIAVSLRFNALRAGEQR
RhilegWSM1455GCS2         EIAGRPLLPSAKRRTREISDLMTAIIRIVMVDVEIAVSLRFNALRAAQSR
Rhileg3841GCS2            EIAGRPLLPSAKRRAREISDLMTAIIRIVMVDVEIAVSLRFNALRAAQSR
RhilegVc2GCS2             EIAGRPLLPSAKRRTREISALMTAIIRIVMVDVEIAVSLRFNALRAGEQR
Rhietl8C3GCS              EIAGRPLLPSAKRRSREISELMTAIIRIVMVDVEIAVSLRFNALRAAQNR
RhietlCIAT652GCS2         EIAGRPLLPSAKRRSREISELMTAIIRIVMVDVEIAVSLRFNALRAAQNR
RhietlCFN42GCS2           EIAGRPLLPSARRRTREISDLMTAIIRIVMVDVEIAVSLRFNALRAAQSR
SinfreGR64GCS             NAWPKSLLPFGKARRNELCRLVAALVRAAFVDGEIAVSLRFNALRQQHQR
SinfreHH103GCS            NAWPKSLLPFGKARRNELCRLVAALVRAAFVDSEIAVSLRFNALRQQHQR
SinfreUSDA257GCS          EAWPKSLLPFGKARRNELCRLVAALVRAACVDSEIAVSLRFNALRQQQQR
Sinmel1021GCS             DAWPKSILSLGKARRRELRDLVAALVRAAFVDTEIAVSLRFNALRQQHQR
RhietlCIAT652GCS1         KQWPS---VFARQQGRQLAEKLSAVIKSAMLDMDYSISVYLETLEAKRRA
RhietlCNPAF512GCS         KQWPS---VFARQQGRQLAEKLSAVIKSAMLDMDYSISVYLETLEAKRRA
RhietlGR56GCS             KQWPS---VFARQQGRQLAEKLSAVIKSGMLDMDYSISVYLETLEAKRRA
Rhileg128C53GCS1          KQWPS---IFARQQGKALAEKLSAVIKAGMLDVDYSISVYLETLEAKRRA
RhilegUPM1137GCS1         KQWPS---IFARREGKALAEKLSAVIKAGMLDMDYSISVYLETLEAKRRA
RhilegGB30GCS1            KQWPS---IFARQQGKALAEKLSAVIKAGMLDMDYSISVYLETLEAKRRA
RhilegPs8GCS1             KQWPS---IFARQQGKALAEKLSAVIKAGMLDMDYSISVYLETLEAKRRA
RhilegVc2GCS1             KQWPS---IFARQQGKALAEKLSAVIKAGMLDMDYSISVYLETLEAKRRA
RhilegVh3GCS1             KQWPS---IFARQQGKALAEKLSAVIKVGMLDMDYSISVYLETLEAKRRA
RhilegVF39GCS1            KQWPS---IFARQQGKALAEKLSAVIKSGMLDMDYSISVYLETLEAKRRA
Rhileg3841GCS1            KQWPS---IFARQQGKALAEKLSAVIKSGMLDMDYSISVYLETLEAKRRA
RhilegWSM1455GCS1         KQWPS---IFARREGKALAQKLSAVIKAGMLDMDYSISVYLETLEAKRRA
RhilegWSM1481GCS1         KQWPS---IFARREGKALAQKLSAVIKAGMLDMDYSISVYLETLEAKRRA
Rhileg248GCS1             KQWPS---IFARREGKALAEKLSAVIKAGMLDMDYSISVYLETLEAKRRA
RhilegTOMGCS1             KQWPS---IFARREGKALAEKLSAVIKSGMLDMDYSISVYLDTLEAKRRV
RhietlCFN42GCS1           KQWPS---VFARQQGRQLAEKLSAVIKSGMLDIDYSISVYLETLEAKRQA
AgrtumGCS                 SELKG---LFMEKKAKKVKDALSATIKAALLDMDYSISVYLDVLATERQK
MyctubHbtC1               SETITEILGVIAPLAVDVTSGESTTAPV----------------------
CupnecN1Hbt1              PQEQKDLLGMLGPMRKDIVSR-----------------------------
BrajapUSDA4Hbt2           KREKTEVLAAFASHKDEVTAGYVSRAKRG---------------------
BrajapWSM2793Hbt3         KREKTEVLAAFASHKDEVTAGYVSRAKRG---------------------
MyctubHbtC2               TLDDEHRRELLDYLEMAAHSLVNSPF------------------------
BurphySTM815Hbt1          VAAIRFMEPALRIAQSLQLSKFGWDYPIPAEQQALLERIAPRRRPRD---
MycaviHbtC3               PAADRAKIQAARIAWAMHRRLTGADSPELLVTQRVRDDRAPDRTGSDDR-
BraelkWSM1741Hbt1         EQTRKDVLAIAYSLKNEIIHV-----------------------------
BacsubGCS                 SITNQQELLKAIKATTKILNLEQQLVLEAFQSEYNQTRDEQEEKK-----

2/2-folding helix         -------H--------]
3/3-folding helix         ---][--------------H-------------]
```

```
                                       410       420       430       440       450
                                         |         |         |         |         |
BraelkUSDA3254Hbt           --------------------------------------------------
BraelkUSDA3259Hbt           --------------------------------------------------
BraelkUSDA76Hbt2            --------------------------------------------------
Braelk587Hbt1               --------------------------------------------------
BraelkUSDA94Hbt2            --------------------------------------------------
BraelkWSM2783Hbt            --------------------------------------------------
BraelkWSM1741Hbt2           --------------------------------------------------
BrajapUSDA4Hbt1             --------------------------------------------------
BrajapWSM2793Hbt1           --------------------------------------------------
BrajapUSDA123Hbt1           --------------------------------------------------
BrajapUSDA6-8372Hbt1        --------------------------------------------------
BrajapUSDA6-7488Hbt1        --------------------------------------------------
BrajapUSDA38Hbt1            --------------------------------------------------
Brajapin8p8Hbt1             --------------------------------------------------
Brajapis5Hbt1               --------------------------------------------------
BrajapUSDA135Hbt1           --------------------------------------------------
BrajapUSDA122Hbt1           --------------------------------------------------
BrajapUSDA124Hbt1           --------------------------------------------------
BrajapWSM1743Hbt1           --------------------------------------------------
Brajap22Hbt1                --------------------------------------------------
AzodoeUFLA1-100Hbt1         --------------------------------------------------
MeslotNZP2037Hbt2           --------------------------------------------------
MeslotUSDA3471Hbt1          --------------------------------------------------
RhietlCIAT652Hbt2           --------------------------------------------------
RhietlCNPAF512Hbt           --------------------------------------------------
RhietlBrasil5Hbt            --------------------------------------------------
RhietlCFN42Hbt2             --------------------------------------------------
Rhileg3841Hbt2              --------------------------------------------------
RhilegWSM1455Hbt2           --------------------------------------------------
RhilegVF39Hbt1              --------------------------------------------------
RhilegWSM1481Hbt2           --------------------------------------------------
RhilegUPM1131Hbt2           --------------------------------------------------
Rhileg248Hbt2               --------------------------------------------------
RhilegTOMHbt2               --------------------------------------------------
Rhileg128C53Hbt1            --------------------------------------------------
RhilegPs8Hbt2               --------------------------------------------------
RhilegGB30Hbt2              --------------------------------------------------
RhilegUPM1137Hbt2           --------------------------------------------------
RhilegVc2Hbt2               --------------------------------------------------
RhilegVh3Hbt1               --------------------------------------------------
Sinmel1021Hbt2              --------------------------------------------------
RhilupHPC_L_Hbt1            --------------------------------------------------
RhilupHPC_L_Hbt2            --------------------------------------------------
AgrtumHbtC2                 --------------------------------------------------
SinfreUSDA257Hbt            --------------------------------------------------
SinfreHH103Hbt              --------------------------------------------------
SinfreGR64Hbt               --------------------------------------------------
Sinmel1021Hbt1              --------------------------------------------------
RhilegUPM1137Hbt1           --------------------------------------------------
Rhileg128C53Hbt2            --------------------------------------------------
RhilegVc2Hbt1               --------------------------------------------------
RhilegVh3Hbt2               --------------------------------------------------
RhilegGB30Hbt1              --------------------------------------------------
RhilegPs8Hbt1               --------------------------------------------------
Rhileg3841Hbt1              --------------------------------------------------
Rhileg248Hbt1               --------------------------------------------------
RhilegWSM1455Hbt1           --------------------------------------------------
RhilegWSM1481Hbt1           --------------------------------------------------
RhilegVF39Hbt2              --------------------------------------------------
RhilegTOMHbt1               --------------------------------------------------
RhilegUPM1131Hbt1           --------------------------------------------------
RhietlKIM5Hbt               --------------------------------------------------
RhietlCFN42Hbt1             --------------------------------------------------
Rhietl8C3Hbt                --------------------------------------------------
RhietlCIAT652Hbt1           --------------------------------------------------
RhietlCIAT894Hbt            --------------------------------------------------
```

```
RhietlIE4771Hbt          --------------------------------------------------
AzodoeUFLA1-100Hbt2      --------------------------------------------------
Brajap22Hbt2             --------------------------------------------------
BrajapUSDA110Hbt         --------------------------------------------------
BrajapUSDA122Hbt2        --------------------------------------------------
BrajapUSDA124Hbt2        --------------------------------------------------
BrajapWSM1743Hbt2        --------------------------------------------------
BrajapUSDA6-8372Hbt2     --------------------------------------------------
BrajapUSDA6-7488Hbt2     --------------------------------------------------
BrajapUSDA38Hbt2         --------------------------------------------------
BrajapUSDA123Hbt2        --------------------------------------------------
BrajapWSM2793Hbt2        --------------------------------------------------
BrajapUSDA135Hbt2        --------------------------------------------------
BrajapUSDA4Hbt3          --------------------------------------------------
Brajap22Hbt3             --------------------------------------------------
Brajapin8p8Hbt2          --------------------------------------------------
Brajapis5Hbt2            --------------------------------------------------
CupnecHPC_L_Hbt          --------------------------------------------------
CupnecJMP134Hbt          --------------------------------------------------
CupnecN1Hbt2             --------------------------------------------------
BurphySTM815Hbt2         --------------------------------------------------
BraelkUSDA76Hbt1         RPSAIAGDAQATNDDIEAPPGSDETVQFEQHIRPLFRPMDRSSMLFTFDL
Braelk587Hbt2            RPSAIAGDAQATNDDIEAPPGSDETVQFEQHIRPLFRPMDRSSMLFTFDL
BraelkUSDA94Hbt1         RPSATASDAQAANEISVALPGPDEAVQFEHHIRPLFRPMDRSSMLFAFDL
MescicCMG6Hbt            PPGTN---------------------------------------------
MescicWSM1271Hbt         PPGTN---------------------------------------------
MescicWSM4083Hbt         PLKTK---------------------------------------------
MeslotR7AHbt             PPETN---------------------------------------------
MeslotMAFF303099Hbt      PPETN---------------------------------------------
MeslotNZP2037Hbt1        PPETK---------------------------------------------
MeslotR88bHbt            PPETK---------------------------------------------
MeslotCJ3symHbt          PPETK---------------------------------------------
MeslotUSDA3471Hbt2       PPAGKS--------------------------------------------
BurphySTM815fHb          ---------------------------------PFVVARKVKESDEITSF
CupnecHPC_L_fHb          ---------------------------------GLRIARKVQESEIITSF
CupnecN1fHb2             ---------------------------------NFVVREKRPESDVITSF
Sinmel1021fHb            ---------------------------------RFIVREKNPESDVITSF
BacsubfHb                ---------------------------------PFVIAKKERESAEITSF
VitSDgb                  --------------------------------------------------
EsccolfHb                ---------------------------------DFRIVAKTPRSALITSF
RhilegUPM1137fHb         ---------------------------------RFVFAERRQESETITSF
CupnecN1fHb1             ------------------------------------VVRREAQGDQVVAL
CupnecJMP134fHb          ------------------------------------VDRREVQSDTVVAL
SaccerfHb                ---------------------------------PFDITAKEYVASDIVEF
BrajapUSDA110SDgb1       --------------------------------------------------
BrajapUSDA122SDgb1       --------------------------------------------------
Brajapin8p8SDgb          --------------------------------------------------
Brajapis5SDgb            --------------------------------------------------
BrajapUSDA135SDgb1       --------------------------------------------------
BrajapWSM1743SDgb        --------------------------------------------------
BrajapUSDA124SDgb1       --------------------------------------------------
Brajap22SDgb             --------------------------------------------------
BrajapUSDA6-7488SDgb1    --------------------------------------------------
BrajapUSDA6-8372SDgb2    --------------------------------------------------
BrajapUSDA123SDgb1       --------------------------------------------------
BrajapUSDA38SDgb1        --------------------------------------------------
BrajapUSDA4SDgb1         --------------------------------------------------
BrajapWSM2793SDgb1       --------------------------------------------------
BraelkUSDA3254SDgb4      --------------------------------------------------
BraelkUSDA3259SDgb1      --------------------------------------------------
BraelkUSDA94SDgb1        --------------------------------------------------
Braelk587SDgb1           --------------------------------------------------
BraelkUSDA76SDgb1        --------------------------------------------------
BraelkWSM2783SDgb1       --------------------------------------------------
AzodoeUFLA1-100SDgb      --------------------------------------------------
BraelkWSM1741SDgb1       --------------------------------------------------
BraelkWSM2783SDgb2       --------------------------------------------------
BraelkUSDA3254SDgb2      --------------------------------------------------
```

```
BraelkUSDA3259SDgb2        --------------------------------------------------
BraelkUSDA3254SDgb3        --------------------------------------------------
BraelkUSDA3259SDgb4        --------------------------------------------------
Braelk587SDgb2             --------------------------------------------------
BraelkUSDA76SDgb2          --------------------------------------------------
BraelkUSDA94SDgb2          --------------------------------------------------
BrajapUSDA135SDgb2         --------------------------------------------------
BraelkUSDA3254SDgb1        --------------------------------------------------
BraelkUSDA3259SDgb3        --------------------------------------------------
BraelkUSDA94SDgb3          --------------------------------------------------
BraelkUSDA76SDgb3          --------------------------------------------------
Braelk587SDgb3             --------------------------------------------------
BraelkWSM2783SDgb3         --------------------------------------------------
BraelkWSM1741SDgb2         --------------------------------------------------
BrajapUSDA110SDgb2         --------------------------------------------------
BrajapUSDA122SDgb2         --------------------------------------------------
BrajapUSDA4SDgb2           --------------------------------------------------
BrajapWSM2793SDgb2         --------------------------------------------------
BrajapUSDA6-7488SDgb2      --------------------------------------------------
BrajapUSDA6-8372SDgb1      --------------------------------------------------
BrajapUSDA38SDgb2          --------------------------------------------------
BrajapUSDA123SDgb2         --------------------------------------------------
BrajapUSDA124SDgb2         --------------------------------------------------
Brajapin8p8GCS             ---------------------------QQEAIAAAILDFDGRMKVVLERL
Brajapis5GCS               ---------------------------QQEAIAAAILDFDGRMKVVLERL
BraelkWSM2783GCS           -----------------------------QTLDRLATDFEGAIGGVVDIV
RhilegWSM1481GCS2          ALAD--QRADNEAEITRIFGDVIESLSARDLTRRAPVDGDGAYGGIAVAL
Rhileg248GCS2              ALAD--QRADNEAEIIRIFGDVIESLSARDLTRRAPVDGDGAYGGIAVAL
RhilegTOMGCS2              ALAD--QRADSEAEIARIFGDVIEGLTARDLTRRAPVDGDGAHAGIAAAL
RhilegUPM1131GCS           ALAD--QRADNEAEIIRIFGDVIEGLSARDLTRRAPVDGDGAYGGIAVAL
RhilegVF39GCS2             ALAA--QRADNEAEIIRIFGDVIEGLSARDLTRRAPVDGDGAYGGIAVAL
RhietlCIAT894GCS           ALAS--QRADNEAEIARIFGDVIEGLSARDLTRRAPVEGDGAYAGIAAAL
RhilegVh3GCS2              ALAD--QRADNEAEIARIFGDVIEGLSARDLTRRAPVDGDGAYGGIAVAL
Rhileg128C53GCS2           ALAD--QRADNEAEIARIFGDVIDGLSARDLTRRAPVDG--VYAGIAAAL
RhilegPs8GCS2              ALAD--QRADNEAEIARIFGDVIEGLSARDLTRRAPVDGDGAYGGIAVAL
RhilegGB30GCS2             ALAD--QRADNEAEIARIFGDVIEGLSARDLTRRAPVDGDGAYGGIAVAL
RhilegUPM1137GCS2          ALAE--QRADNEAEIARIFGDVIDGLSARDLTRRAPVDG--AYAGIAAAL
RhilegWSM1455GCS2          ALAD--QRADNEAEIMRIFGDVIEGLSARDLTRRAPVDGDGAYGGIAVAL
Rhileg3841GCS2             ALAD--QRADNEAEITRIFGDVIESLSARDLTRRAPVDGDGAYGGIAVAL
RhilegVc2GCS2              ALAD--QRADNEAEIARIFGDVIEGLSARDLTRRAPVDGDGAYGGIAAAL
Rhietl8C3GCS               ALAD--QRADNEAEIVRIFGDVIEGLSARDLTRRAPVDVDGAYAGIAAAL
RhietlCIAT652GCS2          ALAD--QRADNEAEIVRIFGDVIEGLSARDLTRRAPVDVDGAYAGIAAAL
RhietlCFN42GCS2            ALAD--QRAGSEAEIARIFGEVIEGLADRDLTRRAPVDADGAYAGIAAAL
SinfreGR64GCS              QLSD--QRKGDESEVTALFSDFLQALGTGDLGARLPTDAPDAYRPIVAGL
SinfreHH103GCS             QLSD--QRKGDESEVTALFSDFLQALGTGDLGARLPTDAPDAYRPIVAGL
SinfreUSDA257GCS           QLSE--QRKGDESEVTALFSDFIQALGTGDLGARLPTDAPDVYRPIVAGL
Sinmel1021GCS              QLSE--QRRDDESELKTLFADFLRALGEGDLTARLPEDAPDTYQPIVAGL
RhietlCIAT652GCS1          LEEERAKAQSDQAIALEQLRRGLEALSNGDLEATLPCDLPGDFRQMAEHY
RhietlCNPAF512GCS          LEEERAKAQSDQAIALEQLRRGLEALSNGDLEATLPSDLPGDFRQMAEHY
RhietlGR56GCS              LEEERAKAQSDQAIALEQLRRGLEALSNGDLEATLPSDLPGDFRQMAEDY
Rhileg128C53GCS1           LEEERAQAESDQAIALEQLRHGLEALSNGDLEATLPSDLPGNFRQMAEDY
RhilegUPM1137GCS1          LEEERAQAESDQAIALEQLRRGLEALSNGDLEATLPSDLPGNFRQMAEDY
RhilegGB30GCS1             LEEERAQAESDQAIALEQLRRGLEALSKGDLEATLPSDLPGNFRHMAEDY
RhilegPs8GCS1              LEEERAQAESDQAIALEQLRRGLEALSKGDLEATLPSDLPGNFRHMAEDY
RhilegVc2GCS1              LEEERAQAESDQAIALEQLRRGLEALSKGDLEATLPSDLPGNFRQMAEDY
RhilegVh3GCS1              LEEERAQAESDQAIALEQLRRGLEALSKGDLEATLPSDLPGNFRQMAEDY
RhilegVF39GCS1             LEEERAEAESDQAIALEQLRRGLEALSKGDLEATLPTDLPGNFRQMAEDY
Rhileg3841GCS1             LEEERAEAESDQAIALEQLRRGLEALSKGDLEATLPTDLPGNFRQMAEDY
RhilegWSM1455GCS1          LEEQRAEAESDQAIALEQLRRGLEALSNGDLEATLPSDLPGNFRQMAEDY
RhilegWSM1481GCS1          LEEQRAEAESDQAIALEQLRRGLEALSNGDLEATLPSDLPGNFRQMAEDY
Rhileg248GCS1              LEEERAQAESDQAIALEQLRRGLEALSNGDLEATLPSDLPGNFRQMAEDY
RhilegTOMGCS1              LEEQRAQAESDQAIALEQLRRGLEALSNGDLEATLPSDLPGNFRQMAEDY
RhietlCFN42GCS1            LEEQCAQAEADQAMALDQLRRGLEALSSGDLEATLPSDLPGNFRQMAEDY
AgrtumGCS                  VEAEQAQMKKEQDHVLELLNNALDRLANGDLTSSIAEKTAPQFEGLIANF
MyctubHbtC1                --------------------------------------------------
CupnecN1Hbt1               --------------------------------------------------
BrajapUSDA4Hbt2            --------------------------------------------------
BrajapWSM2793Hbt3          --------------------------------------------------
```

```
MyctubHbtC2          --------------------------------------------------
BurphySTM815Hbt1     --------------------------------DDAAEHARPRGEPFPAKI
MycaviHbtC3          --------------------------------------------------
BraelkWSM1741Hbt1    --------------------------------------------------
BacsubGCS            --------------------------------------------------

                              460       470       480       490       500
                                |         |         |         |         |
BraelkUSDA3254Hbt    --------------------------------------------------
BraelkUSDA3259Hbt    --------------------------------------------------
BraelkUSDA76Hbt2     --------------------------------------------------
Braelk587Hbt1        --------------------------------------------------
BraelkUSDA94Hbt2     --------------------------------------------------
BraelkWSM2783Hbt     --------------------------------------------------
BraelkWSM1741Hbt2    --------------------------------------------------
BrajapUSDA4Hbt1      --------------------------------------------------
BrajapWSM2793Hbt1    --------------------------------------------------
BrajapUSDA123Hbt1    --------------------------------------------------
BrajapUSDA6-8372Hbt1 --------------------------------------------------
BrajapUSDA6-7488Hbt1 --------------------------------------------------
BrajapUSDA38Hbt1     --------------------------------------------------
Brajapin8p8Hbt1      --------------------------------------------------
Brajapis5Hbt1        --------------------------------------------------
BrajapUSDA135Hbt1    --------------------------------------------------
BrajapUSDA122Hbt1    --------------------------------------------------
BrajapUSDA124Hbt1    --------------------------------------------------
BrajapWSM1743Hbt1    --------------------------------------------------
Brajap22Hbt1         --------------------------------------------------
AzodoeUFLA1-100Hbt1  --------------------------------------------------
MeslotNZP2037Hbt2    --------------------------------------------------
MeslotUSDA3471Hbt1   --------------------------------------------------
RhietlCIAT652Hbt2    --------------------------------------------------
RhietlCNPAF512Hbt    --------------------------------------------------
RhietlBrasil5Hbt     --------------------------------------------------
RhietlCFN42Hbt2      --------------------------------------------------
Rhileg3841Hbt2       --------------------------------------------------
RhilegWSM1455Hbt2    --------------------------------------------------
RhilegVF39Hbt1       --------------------------------------------------
RhilegWSM1481Hbt2    --------------------------------------------------
RhilegUPM1131Hbt2    --------------------------------------------------
Rhileg248Hbt2        --------------------------------------------------
RhilegTOMHbt2        --------------------------------------------------
Rhileg128C53Hbt1     --------------------------------------------------
RhilegPs8Hbt2        --------------------------------------------------
RhilegGB30Hbt2       --------------------------------------------------
RhilegUPM1137Hbt2    --------------------------------------------------
RhilegVc2Hbt2        --------------------------------------------------
RhilegVh3Hbt1        --------------------------------------------------
Sinmel1021Hbt2       --------------------------------------------------
RhilupHPC_L_Hbt1     --------------------------------------------------
RhilupHPC_L_Hbt2     --------------------------------------------------
AgrtumHbtC2          --------------------------------------------------
SinfreUSDA257Hbt     --------------------------------------------------
SinfreHH103Hbt       --------------------------------------------------
SinfreGR64Hbt        --------------------------------------------------
Sinmel1021Hbt1       --------------------------------------------------
RhilegUPM1137Hbt1    --------------------------------------------------
Rhileg128C53Hbt2     --------------------------------------------------
RhilegVc2Hbt1        --------------------------------------------------
RhilegVh3Hbt2        --------------------------------------------------
RhilegGB30Hbt1       --------------------------------------------------
RhilegPs8Hbt1        --------------------------------------------------
Rhileg3841Hbt1       --------------------------------------------------
Rhileg248Hbt1        --------------------------------------------------
RhilegWSM1455Hbt1    --------------------------------------------------
RhilegWSM1481Hbt1    --------------------------------------------------
RhilegVF39Hbt2       --------------------------------------------------
RhilegTOMHbt1        --------------------------------------------------
```

```
RhilegUPM1131Hbt1          --------------------------------------------------
RhietlKIM5Hbt              --------------------------------------------------
RhietlCFN42Hbt1            --------------------------------------------------
Rhietl8C3Hbt               --------------------------------------------------
RhietlCIAT652Hbt1          --------------------------------------------------
RhietlCIAT894Hbt           --------------------------------------------------
RhietlIE4771Hbt            --------------------------------------------------
AzodoeUFLA1-100Hbt2        --------------------------------------------------
Brajap22Hbt2               --------------------------------------------------
BrajapUSDA110Hbt           --------------------------------------------------
BrajapUSDA122Hbt2          --------------------------------------------------
BrajapUSDA124Hbt2          --------------------------------------------------
BrajapWSM1743Hbt2          --------------------------------------------------
BrajapUSDA6-8372Hbt2       --------------------------------------------------
BrajapUSDA6-7488Hbt2       --------------------------------------------------
BrajapUSDA38Hbt2           --------------------------------------------------
BrajapUSDA123Hbt2          --------------------------------------------------
BrajapWSM2793Hbt2          --------------------------------------------------
BrajapUSDA135Hbt2          --------------------------------------------------
BrajapUSDA4Hbt3            --------------------------------------------------
Brajap22Hbt3               --------------------------------------------------
Brajapin8p8Hbt2            --------------------------------------------------
Brajapis5Hbt2              --------------------------------------------------
CupnecHPC_L_Hbt            --------------------------------------------------
CupnecJMP134Hbt            --------------------------------------------------
CupnecN1Hbt2               --------------------------------------------------
BurphySTM815Hbt2           --------------------------------------------------
BraelkUSDA76Hbt1           WKEEDVARHGQQILARLKAGTMPCDGAWPAARVALFARWANGQTPPT---
Braelk587Hbt2              WKEEDVARHGQQILARLKAGTMPCDGAWPAARVALFARWANGQTPPT---
BraelkUSDA94Hbt1           WKEEDVARHGQQILARLEAGTMPCDGAWPAERVALFARWANALKTVT---
MescicCMG6Hbt              --------------------------------------------------
MescicWSM1271Hbt           --------------------------------------------------
MescicWSM4083Hbt           --------------------------------------------------
MeslotR7AHbt               --------------------------------------------------
MeslotMAFF303099Hbt        --------------------------------------------------
MeslotNZP2037Hbt1          --------------------------------------------------
MeslotR88bHbt              --------------------------------------------------
MeslotCJ3symHbt            --------------------------------------------------
MeslotUSDA3471Hbt2         --------------------------------------------------
BurphySTM815fHb            YLRPADGG----DVLEFQPGQYIGLRLI--VDGE--EIRRNYSLSAAANG
CupnecHPC_L_fHb            YLEPADGG----VLPAFRPGQYLTLLLT--IDGA--PTRRHYSLSDAPGK
CupnecN1fHb2               ILEPVDGG----PLLNFEPGQYTSVAID--VPALGLQQIRQYSLSDMPNG
Sinmel1021fHb              VLEPADGG----PVADFEPGQYTSVAVQ--VPKLGYQQIRQYSLSDSPNG
BacsubfHb                  YLKPEDGK----PLPDFQAGQYISIKVQ--IPDSEYTQIRQYSLSDMPGK
VitSDgb                    --------------------------------------------------
EsccolfHb                  ELEPVDGG----AVAEYRPGQYLGVWLK--PEGFPHQEIRQYSLTRKPDG
RhilegUPM1137fHb           ILRPQDGG----RVLRHKPGQYLTFRFD--AAGRE-GLKRNYSISCAPND
CupnecN1fHb1               TLAAADGQ----PLRAFRPGQYISVEAR--LDDGQ-RQLRQYSLSAESGL
CupnecJMP134fHb            TLSAVDGQ----PLRDFRPGQYVSVEVT--LDDGN-RQQRQYSLSAERGL
SaccerfHb                  TVKPKFGSGIELESLPITPGQYITVNTHPIRQENQYDALRHYSLCSASTK
BrajapUSDA110SDgb1         --------------------------------------------------
BrajapUSDA122SDgb1         --------------------------------------------------
Brajapin8p8SDgb            --------------------------------------------------
Brajapis5SDgb              --------------------------------------------------
BrajapUSDA135SDgb1         --------------------------------------------------
BrajapWSM1743SDgb          --------------------------------------------------
BrajapUSDA124SDgb1         --------------------------------------------------
Brajap22SDgb               --------------------------------------------------
BrajapUSDA6-7488SDgb1      --------------------------------------------------
BrajapUSDA6-8372SDgb2      --------------------------------------------------
BrajapUSDA123SDgb1         --------------------------------------------------
BrajapUSDA38SDgb1          --------------------------------------------------
BrajapUSDA4SDgb1           --------------------------------------------------
BrajapWSM2793SDgb1         --------------------------------------------------
BraelkUSDA3254SDgb4        --------------------------------------------------
BraelkUSDA3259SDgb1        --------------------------------------------------
BraelkUSDA94SDgb1          --------------------------------------------------
Braelk587SDgb1             --------------------------------------------------
```

```
BraelkUSDA76SDgb1        --------------------------------------------------
BraelkWSM2783SDgb1       --------------------------------------------------
AzodoeUFLA1-100SDgb      --------------------------------------------------
BraelkWSM1741SDgb1       --------------------------------------------------
BraelkWSM2783SDgb2       --------------------------------------------------
BraelkUSDA3254SDgb2      --------------------------------------------------
BraelkUSDA3259SDgb2      --------------------------------------------------
BraelkUSDA3254SDgb3      --------------------------------------------------
BraelkUSDA3259SDgb4      --------------------------------------------------
Braelk587SDgb2           --------------------------------------------------
BraelkUSDA76SDgb2        --------------------------------------------------
BraelkUSDA94SDgb2        --------------------------------------------------
BrajapUSDA135SDgb2       --------------------------------------------------
BraelkUSDA3254SDgb1      --------------------------------------------------
BraelkUSDA3259SDgb3      --------------------------------------------------
BraelkUSDA94SDgb3        --------------------------------------------------
BraelkUSDA76SDgb3        --------------------------------------------------
Braelk587SDgb3           --------------------------------------------------
BraelkWSM2783SDgb3       --------------------------------------------------
BraelkWSM1741SDgb2       --------------------------------------------------
BrajapUSDA110SDgb2       --------------------------------------------------
BrajapUSDA122SDgb2       --------------------------------------------------
BrajapUSDA4SDgb2         --------------------------------------------------
BrajapWSM2793SDgb2       --------------------------------------------------
BrajapUSDA6-7488SDgb2    --------------------------------------------------
BrajapUSDA6-8372SDgb1    --------------------------------------------------
BrajapUSDA38SDgb2        --------------------------------------------------
BrajapUSDA123SDgb2       --------------------------------------------------
BrajapUSDA124SDgb2       --------------------------------------------------
Brajapin8p8GCS           GSSATGLQGVADVLSASAERSTRQSSVVAAGSEEASTNVQAVAASTEELA
Brajapis5GCS             GSSATGLQGVADVLSASAERSTRQSSVVAAGSEEASTNVQAVAASTEELA
BraelkWSM2783GCS         ASASTELQASAQSLQGATDRTSAKSGLVTVASREATTNVNAVAAAVEQLT
RhilegWSM1481GCS2        NGALDGLQAEFTTITERTVKAEAATGSLAGLSRQFAGTASGQADRLQLSA
Rhileg248GCS2            NGALDGLQAEFTTITERTAKAEAATGSLAGLSRQFAGTASAQADRLQLSA
RhilegTOMGCS2            NGALDGLQAEFTTITERTAKAEAATGSLAGMSRQFAGTASGQADRLQLSA
RhilegUPM1131GCS         NGALDGLQAEFTAITERTVKAEAATGSLAGLSRQFAGTASGQADRLQLSA
RhilegVF39GCS2           NGALDGLQAEFTTITERTVKAEAATGSLAGLSRQFAGTASGQADRLQLSA
RhietlCIAT894GCS         NGALDGLQAEFAAISERTLKAEAATGSLAGLSRQFAGTASGHADRLQLSA
RhilegVh3GCS2            NGALDGLQAEFTAITERTVKAEAATGSLAGLSRQFAGTASGQADRLQLSA
Rhileg128C53GCS2         NGALDGLQAEFAAITERTVKAEAATGSLAGLSRQFASTASGQADRLQLSA
RhilegPs8GCS2            NGALDGLQAEFTAITERTVKAEAATGSLAGLSRQFAGTASGQADRLQLSA
RhilegGB30GCS2           NGALDGLQAEFTAITERTVKAEAATGSLAGLSRQFAGTASGQADRLQLSA
RhilegUPM1137GCS2        NGALDGLQAEFTAITERTVKAEAATGSLAGLSRQFAGTASGQADRLLLSA
RhilegWSM1455GCS2        NGALDGLQAEFTTITERTVKAEAATGSLAGLSRQFAGTASGQADRLQLSA
Rhileg3841GCS2           NGALDGLQAEFTTITERTVKAEAATGSLAGLSRQFAGTASGQADRLQLSA
RhilegVc2GCS2            NGALDGLQAEFTAITERTVTAEAATGSLAGLARQFAGTASGQADRLQRSA
Rhietl8C3GCS             NGALDGLQAEFADITGRTVKAEAATASLADLSRQFAGTASGQADRLQLSA
RhietlCIAT652GCS2        NGALDGLQAEFADITGRTVKAEAATASLADLSRQFAGTASGQADRLQLSA
RhietlCFN42GCS2          NDALDGLQAEFAAMTERTDKAETATSSLAGQSRQFAGTASSQAERLQLSA
SinfreGR64GCS            NAALDQIQNALLSGDKRAAAAESMVESLRGRTSEFSGTASDEAEALTGQV
SinfreHH103GCS           NAALDQIQNALLSGDKRAAAAESMVKTLRGRTSEFSGTASDEAEALTGQV
SinfreUSDA257GCS         NAALDQIQQALLSGDKRAAAAESMVESLRSRTSEFSGSAGGEAEALTGQV
Sinmel1021GCS            NTALGQIRDALLAADKRATAAEAIVSDLRGSAAEFSGNAGAEAKAIDQHL
RhietlCIAT652GCS1        NRAVSALRQSFASVRDTSGQIMSGADVISNATNDLALRTAQQAAGVEESS
RhietlCNPAF512GCS        NRAVSALRQSFASVRDTSGQIMSGADVISNATNDLALRTAQQAAGVEESS
RhietlGR56GCS            NRAVSALRQSFASVRDTSGQIMSGADVISNATNDLALRTAQQAAGVEESS
Rhileg128C53GCS1         NRAVSALRQSFASVRETSGHIMSGADVISNATNDLALRTAQQAAGVEESS
RhilegUPM1137GCS1        NRAVSALRQSFASVRETSGHIMSGADVISNATNDLALRTAQQAAGVEESS
RhilegGB30GCS1           NRAVRALSQSFASVRETSGHIMSGADVISNATNDLALRTAQQAAGVEESS
RhilegPs8GCS1            NRAVRALSQSFASVRETSGHIMSGADVISNATNDLALRTAQQAAGVEESS
RhilegVc2GCS1            NRAVRALSQSFASVRETSGHIMSGADVISNATNDLALRTAQQAAGVEESS
RhilegVh3GCS1            NRAVRALSQSFASVRETSGHIMSGADVISNATNDLALRTAQQAAGVEESS
RhilegVF39GCS1           NRAVRALSQSFASVRETSGHIMSGADVISNATNDLALRTAQQAAGVEESS
Rhileg3841GCS1           NRAVRALSQSFASVRETSGHIMSGADVISNATNDLALRTAQQAAGVEESS
RhilegWSM1455GCS1        NRAVRALSQSFASVRETSGHIMSGADVISNATNDLALRTAQQAAGVEESS
RhilegWSM1481GCS1        NRAVRALSQSFASVRETSGHIMSGADVISNATNDLALRTAQQAAGVEESS
Rhileg248GCS1            NRAVRALSQSFASVRETSGHIMSGADVISNATNDLALRTAQQAAGVEESS
RhilegTOMGCS1            NRAVRALSQSFASVRETSGHIMSGADVISNATNDLALRTAQQAAGVEESS
```

```
RhietlCFN42GCS1           NRAVKALRQSFASVRDTSGHIMNGADVISNATNDLALRTAQQAAGVEESS
AgrtumGCS                 NAAVGNLSGAFAQIVEEANKISGNTRELTAATDDMARRTEQQAAALEETA
MyctubHbtC1               --------------------------------------------------
CupnecN1Hbt1              --------------------------------------------------
BrajapUSDA4Hbt2           --------------------------------------------------
BrajapWSM2793Hbt3         --------------------------------------------------
MyctubHbtC2               --------------------------------------------------
BurphySTM815Hbt1          IGKSRDD-------------------------------------------
MycaviHbtC3               --------------------------------------------------
BraelkWSM1741Hbt1         --------------------------------------------------
BacsubGCS                 --------------NLLHQKIQETSGSIANLFSETSRSVQELVDKSEGIS

                                   510       520       530       540       550
                                     |         |         |         |         |
BraelkUSDA3254Hbt         --------------------------------------------------
BraelkUSDA3259Hbt         --------------------------------------------------
BraelkUSDA76Hbt2          --------------------------------------------------
Braelk587Hbt1             --------------------------------------------------
BraelkUSDA94Hbt2          --------------------------------------------------
BraelkWSM2783Hbt          --------------------------------------------------
BraelkWSM1741Hbt2         --------------------------------------------------
BrajapUSDA4Hbt1           --------------------------------------------------
BrajapWSM2793Hbt1         --------------------------------------------------
BrajapUSDA123Hbt1         --------------------------------------------------
BrajapUSDA6-8372Hbt1      --------------------------------------------------
BrajapUSDA6-7488Hbt1      --------------------------------------------------
BrajapUSDA38Hbt1          --------------------------------------------------
Brajapin8p8Hbt1           --------------------------------------------------
Brajapis5Hbt1             --------------------------------------------------
BrajapUSDA135Hbt1         --------------------------------------------------
BrajapUSDA122Hbt1         --------------------------------------------------
BrajapUSDA124Hbt1         --------------------------------------------------
BrajapWSM1743Hbt1         --------------------------------------------------
Brajap22Hbt1              --------------------------------------------------
AzodoeUFLA1-100Hbt1       --------------------------------------------------
MeslotNZP2037Hbt2         --------------------------------------------------
MeslotUSDA3471Hbt1        --------------------------------------------------
RhietlCIAT652Hbt2         --------------------------------------------------
RhietlCNPAF512Hbt         --------------------------------------------------
RhietlBrasil5Hbt          --------------------------------------------------
RhietlCFN42Hbt2           --------------------------------------------------
Rhileg3841Hbt2            --------------------------------------------------
RhilegWSM1455Hbt2         --------------------------------------------------
RhilegVF39Hbt1            --------------------------------------------------
RhilegWSM1481Hbt2         --------------------------------------------------
RhilegUPM1131Hbt2         --------------------------------------------------
Rhileg248Hbt2             --------------------------------------------------
RhilegTOMHbt2             --------------------------------------------------
Rhileg128C53Hbt1          --------------------------------------------------
RhilegPs8Hbt2             --------------------------------------------------
RhilegGB30Hbt2            --------------------------------------------------
RhilegUPM1137Hbt2         --------------------------------------------------
RhilegVc2Hbt2             --------------------------------------------------
RhilegVh3Hbt1             --------------------------------------------------
Sinmel1021Hbt2            --------------------------------------------------
RhilupHPC_L_Hbt1          --------------------------------------------------
RhilupHPC_L_Hbt2          --------------------------------------------------
AgrtumHbtC2               --------------------------------------------------
SinfreUSDA257Hbt          --------------------------------------------------
SinfreHH103Hbt            --------------------------------------------------
SinfreGR64Hbt             --------------------------------------------------
Sinmel1021Hbt1            --------------------------------------------------
RhilegUPM1137Hbt1         --------------------------------------------------
Rhileg128C53Hbt2          --------------------------------------------------
RhilegVc2Hbt1             --------------------------------------------------
RhilegVh3Hbt2             --------------------------------------------------
RhilegGB30Hbt1            --------------------------------------------------
RhilegPs8Hbt1             --------------------------------------------------
```

```
Rhileg3841Hbt1            --------------------------------------------------
Rhileg248Hbt1             --------------------------------------------------
RhilegWSM1455Hbt1         --------------------------------------------------
RhilegWSM1481Hbt1         --------------------------------------------------
RhilegVF39Hbt2            --------------------------------------------------
RhilegTOMHbt1             --------------------------------------------------
RhilegUPM1131Hbt1         --------------------------------------------------
RhietlKIM5Hbt             --------------------------------------------------
RhietlCFN42Hbt1           --------------------------------------------------
Rhietl8C3Hbt              --------------------------------------------------
RhietlCIAT652Hbt1         --------------------------------------------------
RhietlCIAT894Hbt          --------------------------------------------------
RhietlIE4771Hbt           --------------------------------------------------
AzodoeUFLA1-100Hbt2       --------------------------------------------------
Brajap22Hbt2              --------------------------------------------------
BrajapUSDA110Hbt          --------------------------------------------------
BrajapUSDA122Hbt2         --------------------------------------------------
BrajapUSDA124Hbt2         --------------------------------------------------
BrajapWSM1743Hbt2         --------------------------------------------------
BrajapUSDA6-8372Hbt2      --------------------------------------------------
BrajapUSDA6-7488Hbt2      --------------------------------------------------
BrajapUSDA38Hbt2          --------------------------------------------------
BrajapUSDA123Hbt2         --------------------------------------------------
BrajapWSM2793Hbt2         --------------------------------------------------
BrajapUSDA135Hbt2         --------------------------------------------------
BrajapUSDA4Hbt3           --------------------------------------------------
Brajap22Hbt3              --------------------------------------------------
Brajapin8p8Hbt2           --------------------------------------------------
Brajapis5Hbt2             --------------------------------------------------
CupnecHPC_L_Hbt           --------------------------------------------------
CupnecJMP134Hbt           --------------------------------------------------
CupnecN1Hbt2              --------------------------------------------------
BurphySTM815Hbt2          --------------------------------------------------
BraelkUSDA76Hbt1          --------------------------------------------------
Braelk587Hbt2             --------------------------------------------------
BraelkUSDA94Hbt1          --------------------------------------------------
MescicCMG6Hbt             --------------------------------------------------
MescicWSM1271Hbt          --------------------------------------------------
MescicWSM4083Hbt          --------------------------------------------------
MeslotR7AHbt              --------------------------------------------------
MeslotMAFF303099Hbt       --------------------------------------------------
MeslotNZP2037Hbt1         --------------------------------------------------
MeslotR88bHbt             --------------------------------------------------
MeslotCJ3symHbt           --------------------------------------------------
MeslotUSDA3471Hbt2        --------------------------------------------------
BurphySTM815fHb           REYRISVKREPN------GKGSNYLHDVVKEGDTLDLYAPSGDFTLE---
CupnecHPC_L_fHb           PWYRISVKREPG------GRASNWLHDHAAVGDVLQALQPCGDFVLE---
CupnecN1fHb2              RSYRISVKREAGGTQP-PGYVSNLLHDHVNVGDEVRLAAPYGSFHID---
Sinmel1021fHb             RSYRISVKREDGGLGT-PGYVSSLLHDEINVGDEPKLAAPYGNFYID---
BacsubfHb                 DYYRISVKKD--------GVVSSYLHDGLQEGDSVEISAPAGDFVLD---
VitSDgb                   --------------------------------------------------
EsccolfHb                 KGYRIAVKREEG------GQVSNWLHNHANVGDVVKLVAPAGDFFMA---
RhilegUPM1137fHb          EHYRISVKREPQ------GDASVYLHDEASAGTVVECTPPAGDFFLS---
CupnecN1fHb1              PTWRISVKREAGDRTTPAGAVSNWLHANAQVGTELKVSAPFGEFTPA---
CupnecJMP134fHb           PTWQISVKREDGDHATPAGAVSNWLHANAQPGTELSVSAPFGDFAPR---
SaccerfHb                 NGLRFAVKMEAARENFPAGLVSEYLHKDAKVGDEIKLSAPAGDFAINKEL
BrajapUSDA110SDgb1        --------------------------------------------------
BrajapUSDA122SDgb1        --------------------------------------------------
Brajapin8p8SDgb           --------------------------------------------------
Brajapis5SDgb             --------------------------------------------------
BrajapUSDA135SDgb1        --------------------------------------------------
BrajapWSM1743SDgb         --------------------------------------------------
BrajapUSDA124SDgb1        --------------------------------------------------
Brajap22SDgb              --------------------------------------------------
BrajapUSDA6-7488SDgb1     --------------------------------------------------
BrajapUSDA6-8372SDgb2     --------------------------------------------------
BrajapUSDA123SDgb1        --------------------------------------------------
BrajapUSDA38SDgb1         --------------------------------------------------
```

```
BrajapUSDA4SDgb1          --------------------------------------------------
BrajapWSM2793SDgb1        --------------------------------------------------
BraelkUSDA3254SDgb4       --------------------------------------------------
BraelkUSDA3259SDgb1       --------------------------------------------------
BraelkUSDA94SDgb1         --------------------------------------------------
Braelk587SDgb1            --------------------------------------------------
BraelkUSDA76SDgb1         --------------------------------------------------
BraelkWSM2783SDgb1        --------------------------------------------------
AzodoeUFLA1-100SDgb       --------------------------------------------------
BraelkWSM1741SDgb1        --------------------------------------------------
BraelkWSM2783SDgb2        --------------------------------------------------
BraelkUSDA3254SDgb2       --------------------------------------------------
BraelkUSDA3259SDgb2       --------------------------------------------------
BraelkUSDA3254SDgb3       --------------------------------------------------
BraelkUSDA3259SDgb4       --------------------------------------------------
Braelk587SDgb2            --------------------------------------------------
BraelkUSDA76SDgb2         --------------------------------------------------
BraelkUSDA94SDgb2         --------------------------------------------------
BrajapUSDA135SDgb2        --------------------------------------------------
BraelkUSDA3254SDgb1       --------------------------------------------------
BraelkUSDA3259SDgb3       --------------------------------------------------
BraelkUSDA94SDgb3         --------------------------------------------------
BraelkUSDA76SDgb3         --------------------------------------------------
Braelk587SDgb3            --------------------------------------------------
BraelkWSM2783SDgb3        --------------------------------------------------
BraelkWSM1741SDgb2        --------------------------------------------------
BrajapUSDA110SDgb2        --------------------------------------------------
BrajapUSDA122SDgb2        --------------------------------------------------
BrajapUSDA4SDgb2          --------------------------------------------------
BrajapWSM2793SDgb2        --------------------------------------------------
BrajapUSDA6-7488SDgb2     --------------------------------------------------
BrajapUSDA6-8372SDgb1     --------------------------------------------------
BrajapUSDA38SDgb2         --------------------------------------------------
BrajapUSDA123SDgb2        --------------------------------------------------
BrajapUSDA124SDgb2        --------------------------------------------------
Brajapin8p8GCS            SSIKEIGRQVSESTRMTGEAVDQAARSGAAIES--------------LAR
Brajapis5GCS              SSIKEIGRQVSESTRMTGEAVDQAARSGAAIES--------------LAR
BraelkWSM2783GCS          GSIREIGERVTDSARIATEATAAINAAGDKMQK--------------LSA
RhilegWSM1481GCS2         AALAGIAGSVRDGAADSRAAEQAAATTRAAVEESGEVVGRAISAMADIEQ
Rhileg248GCS2             AALACIAGSVRNGAADSRAAEQAAASTRAAIEESGEVVGRAISAMADIEH
RhilegTOMGCS2             AALAGIAGGVRDGAADSRAAEQAAASTRAAVEESGEVVGRAISAMADIEQ
RhilegUPM1131GCS          AALAGIAGSVRDGAADSRAAEQAAATTRAAVEESGEVVGRAISAMADIEQ
RhilegVF39GCS2            TALAGIAGSVRDGAADSRAAEQAAASTRAAVEESGEVVGRAISAMADIEQ
RhietlCIAT894GCS          AALAGIADSVRDGAAESRAAEQAAASTRAAVEESGEVVGRAISAMADIEQ
RhilegVh3GCS2             AALAGIAGSVRDGAADSRAAEQAAATTRAAVEESGEVVGRAISAMADIEQ
Rhileg128C53GCS2          AALAGIAGSVRDGAADSRAAEQAAATTRAAVEASGEVVGRAISAMADIEQ
RhilegPs8GCS2             AALAGIAGSVRHGAADSRAAEQAAATTRAAVEESGEVVGRAISAMADIEQ
RhilegGB30GCS2            AALAGIAGSVRHGAADSRAAEQAAATTRAAVEESGEVVGRAISAMADIEQ
RhilegUPM1137GCS2         AALAGIAGSVRDGAADSRAAEQAAATTRAAVEASGEVVGRAISAMADIEQ
RhilegWSM1455GCS2         AALAGIAGSVRDGAADSRAAEQAAATTRAAVEESGEVVGRAISAMADIEQ
Rhileg3841GCS2            AALAGIAGSVRDGAADSRAAEQAAATTRAAVEESGEVVGRAISAMADIEQ
RhilegVc2GCS2             AALAGIAGSVRDGAADSRAAEQAAATTRAAVEESGEVVGRAISAMADIEQ
Rhietl8C3GCS              ATLAGIAGSVRDGAADSRAAEQAAASTRATVEESGEVVGRAISAMADIEQ
RhietlCIAT652GCS2         ATLAGIAGSVRDGAADSRAAEQAAASTRATVEESGEVVGRAISAMADIEQ
RhietlCFN42GCS2           GALAGIAASVRNAAADSRAAEQAAASTRAAVAQSGEVVGRAISAMADIEQ
SinfreGR64GCS             AALGAMTERMRSGSIRISETEAKASETRQAGERSGEIAGQAISAMADIEA
SinfreHH103GCS            AALGAMTERMRSGSIRISETEAKASETRQAAERSGEIAGQAISAMADIEA
SinfreUSDA257GCS          AALGAMTDRMRSGAMRISAAEAKTNETRHAAERSGEIAGQAISAMADIEA
Sinmel1021GCS             VSLGTVTERIRTGSIRIGETEAQASRTRIAVERSGEIAGQAISAMADIEA
RhietlCIAT652GCS1         AALQQLSVSVGQTAANAEKASDAVRETQQKAKNSGELVTSAVSAMAGIEK
RhietlCNPAF512GCS         AALQQLSVSVGQTAANAEKASDAVRETQQKAKNSGELVTSAVSAMAGIEK
RhietlGR56GCS             AALQQLSVSVGQTAANAEKASGAVRETQQKAKNSGELVTSAVSAMAGIEK
Rhileg128C53GCS1          AALQQLSVSVGQTAANAEKASDAVRETQEKAKNSGELVTSAVSAMAGIEK
RhilegUPM1137GCS1         AALQQLSVSVGQTAANAEKASAAVRETQEKAKNSGELVTSAVSAMAGIEK
RhilegGB30GCS1            AALQQLSVSVGQTAANAEKASAAVRETQEKAKNSGELVTSAVSAMAGIEK
RhilegPs8GCS1             AALQQLSVSVGQTAANAEKASAAVRETQEKAKNSGELVTSAVSAMAGIEK
RhilegVc2GCS1             AALQQLSVSVGQTAANAEKASAAVRETQEKAKNSGELVTSAVSAMAGIEK
RhilegVh3GCS1             AALQQLSVSVGQTAANAEKASAAVRETQEKAKNSGELVTSAVSAMAGIEK
```

```
RhilegVF39GCS1           AALQQLSVSVGQTAANAEKASAAVRETQEKAKNSGELVTSAVSAMAGIEK
Rhileg3841GCS1           AALQQLSVSVGQTAANAEKASAAVRETQEKAKNSGELVTSAVSAMAGIEK
RhilegWSM1455GCS1        AALQQLSVSVGQTAANAEKASDAVRETQEKAKNSGELVTSAVSAMAGIEK
RhilegWSM1481GCS1        AALQQLSVSVGQTAANAEKASDAVRETQEKAKNSGELVTSAVSAMAGIEK
Rhileg248GCS1            AALQQLSVSVGQTAANAEKASDAVRETQQKAKNSGELVTSAVSAMAGIEK
RhilegTOMGCS1            AALQQLSVSVGQTAANAEKASDAMRETQQKAKNSGELVTSAVSAMAGIEK
RhietlCFN42GCS1          AALQQLSVSVGQTAANAEKASDAVRETQQKAKNSGELVTSAVSAMAGIEK
AgrtumGCS                AAVEEITTISKLSAQRSEEAKAIVESSAVEAARSRDVVTDAVKAMGAIEE
MyctubHbtC1              --------------------------------------------------
CupnecN1Hbt1             --------------------------------------------------
BrajapUSDA4Hbt2          --------------------------------------------------
BrajapWSM2793Hbt3        --------------------------------------------------
MyctubHbtC2              --------------------------------------------------
BurphySTM815Hbt1         --------------------------------------------------
MycaviHbtC3              --------------------------------------------------
BraelkWSM1741Hbt1        --------------------------------------------------
BacsubGCS                QASKAGTVTSSTVEEKSIGGKKELEVQQKQMNKIDTSLVQIEKEMVKLDE

                                   560       570       580       590       600
                                     |         |         |         |         |
BraelkUSDA3254Hbt        --------------------------------------------------
BraelkUSDA3259Hbt        --------------------------------------------------
BraelkUSDA76Hbt2         --------------------------------------------------
Braelk587Hbt1            --------------------------------------------------
BraelkUSDA94Hbt2         --------------------------------------------------
BraelkWSM2783Hbt         --------------------------------------------------
BraelkWSM1741Hbt2        --------------------------------------------------
BrajapUSDA4Hbt1          --------------------------------------------------
BrajapWSM2793Hbt1        --------------------------------------------------
BrajapUSDA123Hbt1        --------------------------------------------------
BrajapUSDA6-8372Hbt1     --------------------------------------------------
BrajapUSDA6-7488Hbt1     --------------------------------------------------
BrajapUSDA38Hbt1         --------------------------------------------------
Brajapin8p8Hbt1          --------------------------------------------------
Brajapis5Hbt1            --------------------------------------------------
BrajapUSDA135Hbt1        --------------------------------------------------
BrajapUSDA122Hbt1        --------------------------------------------------
BrajapUSDA124Hbt1        --------------------------------------------------
BrajapWSM1743Hbt1        --------------------------------------------------
Brajap22Hbt1             --------------------------------------------------
AzodoeUFLA1-100Hbt1      --------------------------------------------------
MeslotNZP2037Hbt2        --------------------------------------------------
MeslotUSDA3471Hbt1       --------------------------------------------------
RhietlCIAT652Hbt2        --------------------------------------------------
RhietlCNPAF512Hbt        --------------------------------------------------
RhietlBrasil5Hbt         --------------------------------------------------
RhietlCFN42Hbt2          --------------------------------------------------
Rhileg3841Hbt2           --------------------------------------------------
RhilegWSM1455Hbt2        --------------------------------------------------
RhilegVF39Hbt1           --------------------------------------------------
RhilegWSM1481Hbt2        --------------------------------------------------
RhilegUPM1131Hbt2        --------------------------------------------------
Rhileg248Hbt2            --------------------------------------------------
RhilegTOMHbt2            --------------------------------------------------
Rhileg128C53Hbt1         --------------------------------------------------
RhilegPs8Hbt2            --------------------------------------------------
RhilegGB30Hbt2           --------------------------------------------------
RhilegUPM1137Hbt2        --------------------------------------------------
RhilegVc2Hbt2            --------------------------------------------------
RhilegVh3Hbt1            --------------------------------------------------
Sinmel1021Hbt2           --------------------------------------------------
RhilupHPC_L_Hbt1         --------------------------------------------------
RhilupHPC_L_Hbt2         --------------------------------------------------
AgrtumHbtC2              --------------------------------------------------
SinfreUSDA257Hbt         --------------------------------------------------
SinfreHH103Hbt           --------------------------------------------------
SinfreGR64Hbt            --------------------------------------------------
Sinmel1021Hbt1           --------------------------------------------------
```

```
RhilegUPM1137Hbt1          --------------------------------------------------
Rhileg128C53Hbt2           --------------------------------------------------
RhilegVc2Hbt1              --------------------------------------------------
RhilegVh3Hbt2              --------------------------------------------------
RhilegGB30Hbt1             --------------------------------------------------
RhilegPs8Hbt1              --------------------------------------------------
Rhileg3841Hbt1             --------------------------------------------------
Rhileg248Hbt1              --------------------------------------------------
RhilegWSM1455Hbt1          --------------------------------------------------
RhilegWSM1481Hbt1          --------------------------------------------------
RhilegVF39Hbt2             --------------------------------------------------
RhilegTOMHbt1              --------------------------------------------------
RhilegUPM1131Hbt1          --------------------------------------------------
RhietlKIM5Hbt              --------------------------------------------------
RhietlCFN42Hbt1            --------------------------------------------------
Rhietl8C3Hbt               --------------------------------------------------
RhietlCIAT652Hbt1          --------------------------------------------------
RhietlCIAT894Hbt           --------------------------------------------------
RhietlIE4771Hbt            --------------------------------------------------
AzodoeUFLA1-100Hbt2        --------------------------------------------------
Brajap22Hbt2               --------------------------------------------------
BrajapUSDA110Hbt           --------------------------------------------------
BrajapUSDA122Hbt2          --------------------------------------------------
BrajapUSDA124Hbt2          --------------------------------------------------
BrajapWSM1743Hbt2          --------------------------------------------------
BrajapUSDA6-8372Hbt2       --------------------------------------------------
BrajapUSDA6-7488Hbt2       --------------------------------------------------
BrajapUSDA38Hbt2           --------------------------------------------------
BrajapUSDA123Hbt2          --------------------------------------------------
BrajapWSM2793Hbt2          --------------------------------------------------
BrajapUSDA135Hbt2          --------------------------------------------------
BrajapUSDA4Hbt3            --------------------------------------------------
Brajap22Hbt3               --------------------------------------------------
Brajapin8p8Hbt2            --------------------------------------------------
Brajapis5Hbt2              --------------------------------------------------
CupnecHPC_L_Hbt            --------------------------------------------------
CupnecJMP134Hbt            --------------------------------------------------
CupnecN1Hbt2               --------------------------------------------------
BurphySTM815Hbt2           --------------------------------------------------
BraelkUSDA76Hbt1           --------------------------------------------------
Braelk587Hbt2              --------------------------------------------------
BraelkUSDA94Hbt1           --------------------------------------------------
MescicCMG6Hbt              --------------------------------------------------
MescicWSM1271Hbt           --------------------------------------------------
MescicWSM4083Hbt           --------------------------------------------------
MeslotR7AHbt               --------------------------------------------------
MeslotMAFF303099Hbt        --------------------------------------------------
MeslotNZP2037Hbt1          --------------------------------------------------
MeslotR88bHbt              --------------------------------------------------
MeslotCJ3symHbt            --------------------------------------------------
MeslotUSDA3471Hbt2         --------------------------------------------------
BurphySTM815fHb            HSD--KPLVLISGGVGITPTLAMLNAALQT--SRPIHFIHATRHGGVHAF
CupnecHPC_L_fHb            PAADERPLVLVTGGVGITPAISMLEAAAPA--GRPIQFIHAARHGGVHAF
CupnecN1fHb2               VNAR-TPIVLISGGVGLTPMISMLKNALQE-PPRQVVFVHGARNSAVHAM
Sinmel1021fHb              VSAT-TPIVLISGGVGLTPMVSMLKKALQT-PPRKVVFVHGARNSAVHAM
BacsubfHb                  ASSQ-KDLVLISAGVGITPMISMLKTSVSK-QPKRQILFIHAAKNSEYHA
VitSDgb                    --------------------------------------------------
EsccolfHb                  VADD-TPVTLISAGVGQTPMLAMLDTLAKAGHTAQVNWFHAAENGDVHAF
RhilegUPM1137fHb           DPPQ-RPVVLLSGGVGLTPMVSILEALAEKHAGHPTFYIHGTASRATHAF
CupnecN1fHb1               LDGR-RPLVLLSAGIGITPMLSVLRTLAAQGSQRQVLFAHAARDGRHHAH
CupnecJMP134fHb            LDNH-RPIVLLSAGIGITPMLSVLRTLAAQGSRREILFAHAARDGRHHAH
SaccerfHb                  IHQNEVPLVLLSSGVGVTPLLAMLEEQVKCNPNRPIYWIQSSYDEKTQAF
BrajapUSDA110SDgb1         --------------------------------------------------
BrajapUSDA122SDgb1         --------------------------------------------------
Brajapin8p8SDgb            --------------------------------------------------
Brajapis5SDgb              --------------------------------------------------
BrajapUSDA135SDgb1         --------------------------------------------------
BrajapWSM1743SDgb          --------------------------------------------------
```

```
BrajapUSDA124SDgb1          --------------------------------------------------
Brajap22SDgb                --------------------------------------------------
BrajapUSDA6-7488SDgb1       --------------------------------------------------
BrajapUSDA6-8372SDgb2       --------------------------------------------------
BrajapUSDA123SDgb1          --------------------------------------------------
BrajapUSDA38SDgb1           --------------------------------------------------
BrajapUSDA4SDgb1            --------------------------------------------------
BrajapWSM2793SDgb1          --------------------------------------------------
BraelkUSDA3254SDgb4         --------------------------------------------------
BraelkUSDA3259SDgb1         --------------------------------------------------
BraelkUSDA94SDgb1           --------------------------------------------------
Braelk587SDgb1              --------------------------------------------------
BraelkUSDA76SDgb1           --------------------------------------------------
BraelkWSM2783SDgb1          --------------------------------------------------
AzodoeUFLA1-100SDgb         --------------------------------------------------
BraelkWSM1741SDgb1          --------------------------------------------------
BraelkWSM2783SDgb2          --------------------------------------------------
BraelkUSDA3254SDgb2         --------------------------------------------------
BraelkUSDA3259SDgb2         --------------------------------------------------
BraelkUSDA3254SDgb3         --------------------------------------------------
BraelkUSDA3259SDgb4         --------------------------------------------------
Braelk587SDgb2              --------------------------------------------------
BraelkUSDA76SDgb2           --------------------------------------------------
BraelkUSDA94SDgb2           --------------------------------------------------
BrajapUSDA135SDgb2          --------------------------------------------------
BraelkUSDA3254SDgb1         --------------------------------------------------
BraelkUSDA3259SDgb3         --------------------------------------------------
BraelkUSDA94SDgb3           --------------------------------------------------
BraelkUSDA76SDgb3           --------------------------------------------------
Braelk587SDgb3              --------------------------------------------------
BraelkWSM2783SDgb3          --------------------------------------------------
BraelkWSM1741SDgb2          --------------------------------------------------
BrajapUSDA110SDgb2          --------------------------------------------------
BrajapUSDA122SDgb2          --------------------------------------------------
BrajapUSDA4SDgb2            --------------------------------------------------
BrajapWSM2793SDgb2          --------------------------------------------------
BrajapUSDA6-7488SDgb2       --------------------------------------------------
BrajapUSDA6-8372SDgb1       --------------------------------------------------
BrajapUSDA38SDgb2           --------------------------------------------------
BrajapUSDA123SDgb2          --------------------------------------------------
BrajapUSDA124SDgb2          --------------------------------------------------
Brajapin8p8GCS              AAQRIGDVVELINTIAAQTNLLALNATIEAARAGEAGRGFAVVASEVKAL
Brajapis5GCS                AAQRIGDVVELINTIAAQTNLLALNATIEAARAGEAGRGFAVVASEVKAL
BraelkWSM2783GCS            AAQQIGTIVGLITDIAGQTNLLALNATIEAARAGDAGRGFAVVAQEVKSL
RhilegWSM1481GCS2           SAEKIGQIIGAIDEIAFQTNLLALNAGIEAARAGDSGRGFAVVAQEVRAL
Rhileg248GCS2               SAEKIGQIIGAIDEIAFQTNLLALNAGIEAARAGDSGRGFAVVAQEVRAL
RhilegTOMGCS2               SAEKIGQIIGAIDEIAFQTNLLALNAGIEAARAGDSGRGFAVVAQEVRAL
RhilegUPM1131GCS            SAERIGQIIGAIDEIAFQTNLLALNAGIEAARAGDSGRGFAVVAQEVRAL
RhilegVF39GCS2              SAEKIGQIIGAIDEIAFQTNLLALNAGIEAARAGDSGRGFAVVAQEVRAL
RhietlCIAT894GCS            SAEKIGQIIGAIDEIAFQTNLLALNAGIEAARAGDSGRGFAVVAQEVRAL
RhilegVh3GCS2               SAEKIGQIIGAIDEIAFQTNLLALNAGIEAARAGDSGRGFAVVAQEVRAL
Rhileg128C53GCS2            SAEKIGQIIGAIDEIAFQTNLLALNAGIEAARAGDSGRGFAVVAQEVRAL
RhilegPs8GCS2               SAEKIGQIIGAIDEIAFQTNLLALNAGIEAARAGDSGRGFAVVAQEVRAL
RhilegGB30GCS2              SAEKIGQIIGAIDEIAFQTNLLALNAGIEAARAGDSGRGFAVVAQEVRAL
RhilegUPM1137GCS2           SAEKIGQIIGAIDEIAFQTNLLALNAGIEAARAGDSGRGFAVVAQEVRAL
RhilegWSM1455GCS2           SAEKIGQIIGAIDEIAFQTNLLALNAGIEAARAGDSGRGFAVVAQEVRAL
Rhileg3841GCS2              SAEKIGQIIGAIDEIAFQTNLLALNAGIEAARAGDSGRGFAVVAQEVRAL
RhilegVc2GCS2               SAEKIGQIIGAIDEIAFQTNLLALNAGIEAARAGDSGRGFAVVAQEVRAL
Rhietl8C3GCS                SAEKIGQIHRGRSTRSPFQTQSSGAECRHRSGA-----------------
RhietlCIAT652GCS2           SAEKIGQIIGTIDEIAFQTNLLALNAGIEAARAGDSGRGFAVVAQEVRAL
RhietlCFN42GCS2             SAEKIGQIIGAIDEIAFQTNLLALNAGIEAARAGDSGRGFAVVAQEVRAL
SinfreGR64GCS               SAEKIGQIIGVIDEIAFQTNLLALNAGIEAARAGESGRGFAVVAQEVRAL
SinfreHH103GCS              SAEKIGQIIGVIDEIAFQTNLLALNAGIEAARAGESGRGFAVVAQEVRAL
SinfreUSDA257GCS            SAEKIGQIIGVIDEIAFQTNLLALNAGIEAARAGDSGRGFAVVAQEVRAL
Sinmel1021GCS               SAEKIGQIIGVIDEIAFQTNLLALNAGIEAARAGESGRGFAVVAQEVRAL
RhietlCIAT652GCS1           SSTEISKIIGVIDEIAFQTNLLALNAGVEAARAGDAGKGFAVVAQEVRQL
RhietlCNPAF512GCS           SSTEISKIIGVIDEIAFQTNLLALNAGVEAARAGDAGKGFAVVAQEVRQL
RhietlGR56GCS               SSTEISKIIGVIDEIAFQTNLLALNAGVEAARAGDAGKGFAVVAQEVRQL
```

```
Rhileg128C53GCS1          SSTGISKIIGVIDEIAFQTNLLALNAGVEAARAGDAGKGFAVVAQEVRQL
RhilegUPM1137GCS1         SSTGISKIIGVIDEIAFQTNLLALNAGVEAARAGDAGKGFAVVAQEVRQL
RhilegGB30GCS1            SSTGISKIIGVIDEIAFQTNLLALNAGVEAARAGDAGKGFAVVAQEVRQL
RhilegPs8GCS1             SSTGISKIIGVIDEIAFQTNLLALNAGVEAARAGDAGKGFAVVAQEVRQL
RhilegVc2GCS1             SSTGISKIIGVIDEIAFQTNLLALNAGVEAARAGDAGKGFAVVAQEVRQL
RhilegVh3GCS1             SSTGISKIIGVIDEIAFQTNLLALNAGVEAARAGDAGKGFAVVAQEVRQL
RhilegVF39GCS1            SSTGISKIIGVIDEIAFQTNLLALNAGVEAARAGDAGKGFAVVAQEVRQL
Rhileg3841GCS1            SSTGISKIIGVIDEIAFQTNLLALNAGVEAARAGDAGKGFAVVAQEVRQL
RhilegWSM1455GCS1         SSTGISKIIGVIDEIAFQTNLLALNAGVEAARAGDAGKGFAVVAQEVRQL
RhilegWSM1481GCS1         SSTGISKIIGVIDEIAFQTNLLALNAGVEAARAGDAGKGFAVVAQEVRQL
Rhileg248GCS1             SSTGISKIIGVIDEIAFQTNLLALNAGVEAARAGDAGKGFAVVAQEVRQL
RhilegTOMGCS1             SSTGISKIIGVIDEIAFQTNLLALNAGVEAARAGDAGKGFAVVAQEVRQL
RhietlCFN42GCS1           SSTEISKIIGVIDEIAFQTNLLALNAGVEAARAGDAGKGFAVVAQEVRQL
AgrtumGCS                 SSQKITQIISVIDEISFQTNLLALNAGVEAARAGEAGKGFAVVAQEVREL
MyctubHbtC1               --------------------------------------------------
CupnecN1Hbt1              --------------------------------------------------
BrajapUSDA4Hbt2           --------------------------------------------------
BrajapWSM2793Hbt3         --------------------------------------------------
MyctubHbtC2               --------------------------------------------------
BurphySTM815Hbt1          --------------------------------------------------
MycaviHbtC3               --------------------------------------------------
BraelkWSM1741Hbt1         --------------------------------------------------
BacsubGCS                 IAQQIEKIFGIVTGIAEQTNLLSLNASIESARAGEHGKGFAVVANEVRKL

                                   610       620       630       640       650
                                     |         |         |         |         |
BraelkUSDA3254Hbt         --------------------------------------------------
BraelkUSDA3259Hbt         --------------------------------------------------
BraelkUSDA76Hbt2          --------------------------------------------------
Braelk587Hbt1             --------------------------------------------------
BraelkUSDA94Hbt2          --------------------------------------------------
BraelkWSM2783Hbt          --------------------------------------------------
BraelkWSM1741Hbt2         --------------------------------------------------
BrajapUSDA4Hbt1           --------------------------------------------------
BrajapWSM2793Hbt1         --------------------------------------------------
BrajapUSDA123Hbt1         --------------------------------------------------
BrajapUSDA6-8372Hbt1      --------------------------------------------------
BrajapUSDA6-7488Hbt1      --------------------------------------------------
BrajapUSDA38Hbt1          --------------------------------------------------
Brajapin8p8Hbt1           --------------------------------------------------
Brajapis5Hbt1             --------------------------------------------------
BrajapUSDA135Hbt1         --------------------------------------------------
BrajapUSDA122Hbt1         --------------------------------------------------
BrajapUSDA124Hbt1         --------------------------------------------------
BrajapWSM1743Hbt1         --------------------------------------------------
Brajap22Hbt1              --------------------------------------------------
AzodoeUFLA1-100Hbt1       --------------------------------------------------
MeslotNZP2037Hbt2         --------------------------------------------------
MeslotUSDA3471Hbt1        --------------------------------------------------
RhietlCIAT652Hbt2         --------------------------------------------------
RhietlCNPAF512Hbt         --------------------------------------------------
RhietlBrasil5Hbt          --------------------------------------------------
RhietlCFN42Hbt2           --------------------------------------------------
Rhileg3841Hbt2            --------------------------------------------------
RhilegWSM1455Hbt2         --------------------------------------------------
RhilegVF39Hbt1            --------------------------------------------------
RhilegWSM1481Hbt2         --------------------------------------------------
RhilegUPM1131Hbt2         --------------------------------------------------
Rhileg248Hbt2             --------------------------------------------------
RhilegTOMHbt2             --------------------------------------------------
Rhileg128C53Hbt1          --------------------------------------------------
RhilegPs8Hbt2             --------------------------------------------------
RhilegGB30Hbt2            --------------------------------------------------
RhilegUPM1137Hbt2         --------------------------------------------------
RhilegVc2Hbt2             --------------------------------------------------
RhilegVh3Hbt1             --------------------------------------------------
Sinmel1021Hbt2            --------------------------------------------------
RhilupHPC_L_Hbt1          --------------------------------------------------
```

```
RhilupHPC_L_Hbt2           --------------------------------------------------
AgrtumHbtC2                --------------------------------------------------
SinfreUSDA257Hbt           --------------------------------------------------
SinfreHH103Hbt             --------------------------------------------------
SinfreGR64Hbt              --------------------------------------------------
Sinmel1021Hbt1             --------------------------------------------------
RhilegUPM1137Hbt1          --------------------------------------------------
Rhileg128C53Hbt2           --------------------------------------------------
RhilegVc2Hbt1              --------------------------------------------------
RhilegVh3Hbt2              --------------------------------------------------
RhilegGB30Hbt1             --------------------------------------------------
RhilegPs8Hbt1              --------------------------------------------------
Rhileg3841Hbt1             --------------------------------------------------
Rhileg248Hbt1              --------------------------------------------------
RhilegWSM1455Hbt1          --------------------------------------------------
RhilegWSM1481Hbt1          --------------------------------------------------
RhilegVF39Hbt2             --------------------------------------------------
RhilegTOMHbt1              --------------------------------------------------
RhilegUPM1131Hbt1          --------------------------------------------------
RhietlKIM5Hbt              --------------------------------------------------
RhietlCFN42Hbt1            --------------------------------------------------
Rhietl8C3Hbt               --------------------------------------------------
RhietlCIAT652Hbt1          --------------------------------------------------
RhietlCIAT894Hbt           --------------------------------------------------
RhietlIE4771Hbt            --------------------------------------------------
AzodoeUFLA1-100Hbt2        --------------------------------------------------
Brajap22Hbt2               --------------------------------------------------
BrajapUSDA110Hbt           --------------------------------------------------
BrajapUSDA122Hbt2          --------------------------------------------------
BrajapUSDA124Hbt2          --------------------------------------------------
BrajapWSM1743Hbt2          --------------------------------------------------
BrajapUSDA6-8372Hbt2       --------------------------------------------------
BrajapUSDA6-7488Hbt2       --------------------------------------------------
BrajapUSDA38Hbt2           --------------------------------------------------
BrajapUSDA123Hbt2          --------------------------------------------------
BrajapWSM2793Hbt2          --------------------------------------------------
BrajapUSDA135Hbt2          --------------------------------------------------
BrajapUSDA4Hbt3            --------------------------------------------------
Brajap22Hbt3               --------------------------------------------------
Brajapin8p8Hbt2            --------------------------------------------------
Brajapis5Hbt2              --------------------------------------------------
CupnecHPC_L_Hbt            --------------------------------------------------
CupnecJMP134Hbt            --------------------------------------------------
CupnecN1Hbt2               --------------------------------------------------
BurphySTM815Hbt2           --------------------------------------------------
BraelkUSDA76Hbt1           --------------------------------------------------
Braelk587Hbt2              --------------------------------------------------
BraelkUSDA94Hbt1           --------------------------------------------------
MescicCMG6Hbt              --------------------------------------------------
MescicWSM1271Hbt           --------------------------------------------------
MescicWSM4083Hbt           --------------------------------------------------
MeslotR7AHbt               --------------------------------------------------
MeslotMAFF303099Hbt        --------------------------------------------------
MeslotNZP2037Hbt1          --------------------------------------------------
MeslotR88bHbt              --------------------------------------------------
MeslotCJ3symHbt            --------------------------------------------------
MeslotUSDA3471Hbt2         --------------------------------------------------
BurphySTM815fHb            RDAIDELAARHPQLKRFYVYEKPRQQDDAHHAEG--FIDEDRLIEWMPAT
CupnecHPC_L_fHb            RERVDAIAANYDNVSVCYVYDTPRD-GDNPHAVG--FVTRELLASRLPAD
CupnecN1fHb2               RDRLREAAKAYENFDLFVFYDQPLSEDVQGRDYDYPGLVDVKLIEKSILL
Sinmel1021fHb              RDRLKEASRTYPDFKLFIFYDEPLPTDIEGRDYDFAGLVDVENVKDSILL
BacsubfHb                  LRHEVEEAANHSSVKTAFVYREPTEEDRAGDLQFHEGQIDQQFLKELIAN
VitSDgb                    --------------------------------------------------
EsccolfHb                  ADEVKELGQSLPRFTAHTWYRQPSEADRAKGQFDSEGLMDLSKLEGAFSD
RhilegUPM1137fHb           DSHVKILAARQQATSVATFYD-----QSSDEAEVHSGYISFEWLLANTPF
CupnecN1fHb1               RADLQWARERLPQLATHISYETPQAGDVAGRDYDHAGTMPVAELLRQPDL
CupnecJMP134fHb            RADVAWARERLPQLRTHISYEQPQAADVAGRDYDHAGTMPVAALLDAPDN
SaccerfHb                  KKHVDELLAECANVDKIIVHTDTEP------------LINAAFLKEKSPA
```

```
BrajapUSDA110SDgb1        --------------------------------------------------
BrajapUSDA122SDgb1        --------------------------------------------------
Brajapin8p8SDgb           --------------------------------------------------
Brajapis5SDgb             --------------------------------------------------
BrajapUSDA135SDgb1        --------------------------------------------------
BrajapWSM1743SDgb         --------------------------------------------------
BrajapUSDA124SDgb1        --------------------------------------------------
Brajap22SDgb              --------------------------------------------------
BrajapUSDA6-7488SDgb1     --------------------------------------------------
BrajapUSDA6-8372SDgb2     --------------------------------------------------
BrajapUSDA123SDgb1        --------------------------------------------------
BrajapUSDA38SDgb1         --------------------------------------------------
BrajapUSDA4SDgb1          --------------------------------------------------
BrajapWSM2793SDgb1        --------------------------------------------------
BraelkUSDA3254SDgb4       --------------------------------------------------
BraelkUSDA3259SDgb1       --------------------------------------------------
BraelkUSDA94SDgb1         --------------------------------------------------
Braelk587SDgb1            --------------------------------------------------
BraelkUSDA76SDgb1         --------------------------------------------------
BraelkWSM2783SDgb1        --------------------------------------------------
AzodoeUFLA1-100SDgb       --------------------------------------------------
BraelkWSM1741SDgb1        --------------------------------------------------
BraelkWSM2783SDgb2        --------------------------------------------------
BraelkUSDA3254SDgb2       --------------------------------------------------
BraelkUSDA3259SDgb2       --------------------------------------------------
BraelkUSDA3254SDgb3       --------------------------------------------------
BraelkUSDA3259SDgb4       --------------------------------------------------
Braelk587SDgb2            --------------------------------------------------
BraelkUSDA76SDgb2         --------------------------------------------------
BraelkUSDA94SDgb2         --------------------------------------------------
BrajapUSDA135SDgb2        --------------------------------------------------
BraelkUSDA3254SDgb1       --------------------------------------------------
BraelkUSDA3259SDgb3       --------------------------------------------------
BraelkUSDA94SDgb3         --------------------------------------------------
BraelkUSDA76SDgb3         --------------------------------------------------
Braelk587SDgb3            --------------------------------------------------
BraelkWSM2783SDgb3        --------------------------------------------------
BraelkWSM1741SDgb2        --------------------------------------------------
BrajapUSDA110SDgb2        --------------------------------------------------
BrajapUSDA122SDgb2        --------------------------------------------------
BrajapUSDA4SDgb2          --------------------------------------------------
BrajapWSM2793SDgb2        --------------------------------------------------
BrajapUSDA6-7488SDgb2     --------------------------------------------------
BrajapUSDA6-8372SDgb1     --------------------------------------------------
BrajapUSDA38SDgb2         --------------------------------------------------
BrajapUSDA123SDgb2        --------------------------------------------------
BrajapUSDA124SDgb2        --------------------------------------------------
Brajapin8p8GCS            AEQTARATGEIGQQVLSIQEATTESVGIIRQIGETIAAVDEVATAIAAAV
Brajapis5GCS              AEQTARATGEIGQQVLSIQEATTESVGIIRQIGETIAAVDEVATAIAAAV
BraelkWSM2783GCS          AEQTTKATADIAAQIGDMQQSTTESTSAIGDIVGVIRSMGEVSNAIASAV
RhilegWSM1481GCS2         AQRSAEAAREIKTLVTTTKAQVDAGVQMVGRTQDSIGSIVRQVTDINAAI
Rhileg248GCS2             AQRSAEAAREIKTLVTTTKAQVDAGVQMVGRTQDSIGSIVRQVTDINAAI
RhilegTOMGCS2             AQRSAEAAREIKTLVTTTKAQVNAGVQMVGRTQDSIGSIVRQVTDINAAM
RhilegUPM1131GCS          AQRSAEAAREIKTLVTTTKAQVNAGVQMVGRTQDSIGSIVRQVTDINAAI
RhilegVF39GCS2            AQRSAEAAREIKTLVTTTKAQVDAGVQMVGRTQDSIGSIVRQVTDINAAI
RhietlCIAT894GCS          AQRSAEAAREIKTL------------------------------------
RhilegVh3GCS2             AQRSAEAAREIKTLVTTTKAQVDAGVQMVGRTQDSIGSIVRQVTDINAAI
Rhileg128C53GCS2          AQRSAEAAREIKTLVTTTKAQVDAGVQMVGRTQDSIGSIVRQVTDINAAI
RhilegPs8GCS2             AQRSAEAAREIKTLVTTTKAQVDAGVQMVGRTQDSIGSIVRQVTDINAAI
RhilegGB30GCS2            AQRSAEAAREIKTLVTTTKAQVDAGVQMVGRTQDSIGSIVRQVTDINAAI
RhilegUPM1137GCS2         AQRSAEAAREIKTLVTTTKAQVDAGVQMVGRTQDSIGSIVRQVTDINAAI
RhilegWSM1455GCS2         AQRSAEAAREIKTLVTTTKAQVDAGVQMVGRTQDSIGSIVRQVTDINAAI
Rhileg3841GCS2            AQRSAEAAREIKTLVTTTKAQVDAGVQMVGRTQDSIGSIVRQVTDINAAI
RhilegVc2GCS2             AQRSAEAAREIKTLVTTTKAQVDAGVQMVGRTQDSIGSIVRQVTDINAAI
Rhietl8C3GCS              --------------------------------------------------
RhietlCIAT652GCS2         AQRSAEAAREIKTLVTTTKAQVDAGVQMVGRTQDSIGSIVRQVTDINAAI
RhietlCFN42GCS2           AQRSADAAREIKTLVTTTKAQVDAGVQMVGRTQDSIGSIVRQVTDINAAI
SinfreGR64GCS             AQRSGEAAREIKQLVNGTKTQVEAGVEIVGQTQNAISNIVDQVISINAAV
```

```
SinfreHH103GCS             AQRSGEAAREIKQLVNGTKTQVEAGVEIVGQTQNAISSIVDQVISINAAV
SinfreUSDA257GCS           AQRSGEAAREIKQLVNGTKSQVEAGVEIVGQTQNAISSIVDQVISINAAV
Sinmel1021GCS              AQRSGEAAREIKQLVTGTKGQVVAGVEIVGRTQHAISSIAEQVVSINEAV
RhietlCIAT652GCS1          AQRTAEAAKEIKNLISQSSTQVNEGVGIVSSTGEALNDMISRIDIINRFV
RhietlCNPAF512GCS          AQRTAEAAKEIKNLISQSSTQVNEGVGIVSSTGEALNDMISRIDIINRFV
RhietlGR56GCS              AQRTAEAAKEIKNLISQSSTQVNQGVGIVSSTGEALNDMISRIDIINRFV
Rhileg128C53GCS1           AQRTAEAAKEIKNLISQSSTQVNQGVGIVSSTGEALNDMISRIDIINRFV
RhilegUPM1137GCS1          AQRTAEAAKEIKNLISQSSTQVNQGVGIVSSTGEALNDMISRIVIINRFV
RhilegGB30GCS1             AQRTAEAAKEIKNLISQSSTQVNQGVGIVSSTGEALNDMISRIDIINRFV
RhilegPs8GCS1              AQRTAEAAKEIKNLISQSSTQVNQGVGIVSSTGEALNDMISRIDIINRFV
RhilegVc2GCS1              AQRTAEAAKEIKNLISQSSTQVNQGVGIVSSTGEALNDMISRIDIINRFV
RhilegVh3GCS1              AQRTAEAAKEIKNLISQSSTQVNQGVGIVSSTGEALNDMISRIDIINRFV
RhilegVF39GCS1             AQRTAEAAKEIKNLISQSSTQVNQGVGIVSSTGEALNDMISRIDIINRFV
Rhileg3841GCS1             AQRTAEAAKEIKNLISQSSTQVNQGVGIVSSTGEALNDMISRIDIINRFV
RhilegWSM1455GCS1          AQRTAEAAKEIKNLISQSSTQVNQGVGIVSSTGEALNDMISRIDIINRFV
RhilegWSM1481GCS1          AQRTAEAAKEIKNLISQSSTQVNQGVGIVSSTGEALNDMISRIDIINRFV
Rhileg248GCS1              AQRTAEAAKEIKNLISQSSTQVNQGVGIVSSTGEALNDMISRIDIINRFV
RhilegTOMGCS1              AQRTAEAAKEIKNLISQSSTQVNQGVSIVSSTGEALNDMISRIDIINRFV
RhietlCFN42GCS1            AQRTAEAAKEIKNLISQSSTQVNEGVGIVSSTGEALNDMIGRIDIINRFV
AgrtumGCS                  AQRSANAAKEIKTLIAKSSEDVTQGVSLVNKTGESLNTIGNKVDHIKDHI
MyctubHbtC1                --------------------------------------------------
CupnecN1Hbt1               --------------------------------------------------
BrajapUSDA4Hbt2            --------------------------------------------------
BrajapWSM2793Hbt3          --------------------------------------------------
MyctubHbtC2                --------------------------------------------------
BurphySTM815Hbt1           --------------------------------------------------
MycaviHbtC3                --------------------------------------------------
BraelkWSM1741Hbt1          --------------------------------------------------
BacsubGCS                  SEDTKKTVSTVSELVNNTNTQINIVSKHIKDVNELVSESKEKMTQINRLF

                                     660       670       680       690       700
                                       |         |         |         |         |
BraelkUSDA3254Hbt          --------------------------------------------------
BraelkUSDA3259Hbt          --------------------------------------------------
BraelkUSDA76Hbt2           --------------------------------------------------
Braelk587Hbt1              --------------------------------------------------
BraelkUSDA94Hbt2           --------------------------------------------------
BraelkWSM2783Hbt           --------------------------------------------------
BraelkWSM1741Hbt2          --------------------------------------------------
BrajapUSDA4Hbt1            --------------------------------------------------
BrajapWSM2793Hbt1          --------------------------------------------------
BrajapUSDA123Hbt1          --------------------------------------------------
BrajapUSDA6-8372Hbt1       --------------------------------------------------
BrajapUSDA6-7488Hbt1       --------------------------------------------------
BrajapUSDA38Hbt1           --------------------------------------------------
Brajapin8p8Hbt1            --------------------------------------------------
Brajapis5Hbt1              --------------------------------------------------
BrajapUSDA135Hbt1          --------------------------------------------------
BrajapUSDA122Hbt1          --------------------------------------------------
BrajapUSDA124Hbt1          --------------------------------------------------
BrajapWSM1743Hbt1          --------------------------------------------------
Brajap22Hbt1               --------------------------------------------------
AzodoeUFLA1-100Hbt1        --------------------------------------------------
MeslotNZP2037Hbt2          --------------------------------------------------
MeslotUSDA3471Hbt1         --------------------------------------------------
RhietlCIAT652Hbt2          --------------------------------------------------
RhietlCNPAF512Hbt          --------------------------------------------------
RhietlBrasil5Hbt           --------------------------------------------------
RhietlCFN42Hbt2            --------------------------------------------------
Rhileg3841Hbt2             --------------------------------------------------
RhilegWSM1455Hbt2          --------------------------------------------------
RhilegVF39Hbt1             --------------------------------------------------
RhilegWSM1481Hbt2          --------------------------------------------------
RhilegUPM1131Hbt2          --------------------------------------------------
Rhileg248Hbt2              --------------------------------------------------
RhilegTOMHbt2              --------------------------------------------------
Rhileg128C53Hbt1           --------------------------------------------------
RhilegPs8Hbt2              --------------------------------------------------
```

```
RhilegGB30Hbt2              --------------------------------------------------
RhilegUPM1137Hbt2           --------------------------------------------------
RhilegVc2Hbt2               --------------------------------------------------
RhilegVh3Hbt1               --------------------------------------------------
Sinmel1021Hbt2              --------------------------------------------------
RhilupHPC_L_Hbt1            --------------------------------------------------
RhilupHPC_L_Hbt2            --------------------------------------------------
AgrtumHbtC2                 --------------------------------------------------
SinfreUSDA257Hbt            --------------------------------------------------
SinfreHH103Hbt              --------------------------------------------------
SinfreGR64Hbt               --------------------------------------------------
Sinmel1021Hbt1              --------------------------------------------------
RhilegUPM1137Hbt1           --------------------------------------------------
Rhileg128C53Hbt2            --------------------------------------------------
RhilegVc2Hbt1               --------------------------------------------------
RhilegVh3Hbt2               --------------------------------------------------
RhilegGB30Hbt1              --------------------------------------------------
RhilegPs8Hbt1               --------------------------------------------------
Rhileg3841Hbt1              --------------------------------------------------
Rhileg248Hbt1               --------------------------------------------------
RhilegWSM1455Hbt1           --------------------------------------------------
RhilegWSM1481Hbt1           --------------------------------------------------
RhilegVF39Hbt2              --------------------------------------------------
RhilegTOMHbt1               --------------------------------------------------
RhilegUPM1131Hbt1           --------------------------------------------------
RhietlKIM5Hbt               --------------------------------------------------
RhietlCFN42Hbt1             --------------------------------------------------
Rhietl8C3Hbt                --------------------------------------------------
RhietlCIAT652Hbt1           --------------------------------------------------
RhietlCIAT894Hbt            --------------------------------------------------
RhietlIE4771Hbt             --------------------------------------------------
AzodoeUFLA1-100Hbt2         --------------------------------------------------
Brajap22Hbt2                --------------------------------------------------
BrajapUSDA110Hbt            --------------------------------------------------
BrajapUSDA122Hbt2           --------------------------------------------------
BrajapUSDA124Hbt2           --------------------------------------------------
BrajapWSM1743Hbt2           --------------------------------------------------
BrajapUSDA6-8372Hbt2        --------------------------------------------------
BrajapUSDA6-7488Hbt2        --------------------------------------------------
BrajapUSDA38Hbt2            --------------------------------------------------
BrajapUSDA123Hbt2           --------------------------------------------------
BrajapWSM2793Hbt2           --------------------------------------------------
BrajapUSDA135Hbt2           --------------------------------------------------
BrajapUSDA4Hbt3             --------------------------------------------------
Brajap22Hbt3                --------------------------------------------------
Brajapin8p8Hbt2             --------------------------------------------------
Brajapis5Hbt2               --------------------------------------------------
CupnecHPC_L_Hbt             --------------------------------------------------
CupnecJMP134Hbt             --------------------------------------------------
CupnecN1Hbt2                --------------------------------------------------
BurphySTM815Hbt2            --------------------------------------------------
BraelkUSDA76Hbt1            --------------------------------------------------
Braelk587Hbt2               --------------------------------------------------
BraelkUSDA94Hbt1            --------------------------------------------------
MescicCMG6Hbt               --------------------------------------------------
MescicWSM1271Hbt            --------------------------------------------------
MescicWSM4083Hbt            --------------------------------------------------
MeslotR7AHbt                --------------------------------------------------
MeslotMAFF303099Hbt         --------------------------------------------------
MeslotNZP2037Hbt1           --------------------------------------------------
MeslotR88bHbt               --------------------------------------------------
MeslotCJ3symHbt             --------------------------------------------------
MeslotUSDA3471Hbt2          --------------------------------------------------
BurphySTM815fHb             ---RDVDVYFLGPKPFMKAVKRHLKAIGVPEKQSRFEFFGPAAALD----
CupnecHPC_L_fHb             ---RDVDFYLLGPKAFMRAVHADGRALGIAPERLRFEFFGPLEDLQAA--
CupnecN1fHb2                ---PDADYYICGPIPFMRMQHDALKKLGVHEGCIHYEVFGPDLFAE----
Sinmel1021fHb               ---DDADYYICGPVPFMRMQHDKLLGLGITEARIHYEVFGPDLFAE----
BacsubfHb                   ---TDADYYICGSSSFITAMHKLVSELGSAPESIHYELFGPQLSMAQSV-
```

```
VitSDgb                    --------------------------------------------------
EsccolfHb                  ---PTMQFYLCGPVGFMQFAAKQLVDLGVKQENIHYECFGPHKVL-----
RhilegUPM1137fHb           ---MEADFYICGPRPFMRFFVSGLTQAGVSADRIHYEFFGPTDEVLAA--
CupnecN1fHb1               QRFVDGSFYLCGPLGFMQEQRHALVSAGVPVAHIEREVFGPDLLDDLL--
CupnecJMP134fHb            RLFIDGDFHLCGPLGFMQAQRHALISAGVPVGHIHREVFGPDLLDDLL--
SaccerfHb                  ----HADVYTCGSLAFMQAMIGHLKELEHRDDMIHYEPFGPKMSTVQV--
BrajapUSDA110SDgb1         --------------------------------------------------
BrajapUSDA122SDgb1         --------------------------------------------------
Brajapin8p8SDgb            --------------------------------------------------
Brajapis5SDgb              --------------------------------------------------
BrajapUSDA135SDgb1         --------------------------------------------------
BrajapWSM1743SDgb          --------------------------------------------------
BrajapUSDA124SDgb1         --------------------------------------------------
Brajap22SDgb               --------------------------------------------------
BrajapUSDA6-7488SDgb1      --------------------------------------------------
BrajapUSDA6-8372SDgb2      --------------------------------------------------
BrajapUSDA123SDgb1         --------------------------------------------------
BrajapUSDA38SDgb1          --------------------------------------------------
BrajapUSDA4SDgb1           --------------------------------------------------
BrajapWSM2793SDgb1         --------------------------------------------------
BraelkUSDA3254SDgb4        --------------------------------------------------
BraelkUSDA3259SDgb1        --------------------------------------------------
BraelkUSDA94SDgb1          --------------------------------------------------
Braelk587SDgb1             --------------------------------------------------
BraelkUSDA76SDgb1          --------------------------------------------------
BraelkWSM2783SDgb1         --------------------------------------------------
AzodoeUFLA1-100SDgb        --------------------------------------------------
BraelkWSM1741SDgb1         --------------------------------------------------
BraelkWSM2783SDgb2         --------------------------------------------------
BraelkUSDA3254SDgb2        --------------------------------------------------
BraelkUSDA3259SDgb2        --------------------------------------------------
BraelkUSDA3254SDgb3        --------------------------------------------------
BraelkUSDA3259SDgb4        --------------------------------------------------
Braelk587SDgb2             --------------------------------------------------
BraelkUSDA76SDgb2          --------------------------------------------------
BraelkUSDA94SDgb2          --------------------------------------------------
BrajapUSDA135SDgb2         --------------------------------------------------
BraelkUSDA3254SDgb1        --------------------------------------------------
BraelkUSDA3259SDgb3        --------------------------------------------------
BraelkUSDA94SDgb3          --------------------------------------------------
BraelkUSDA76SDgb3          --------------------------------------------------
Braelk587SDgb3             --------------------------------------------------
BraelkWSM2783SDgb3         --------------------------------------------------
BraelkWSM1741SDgb2         --------------------------------------------------
BrajapUSDA110SDgb2         --------------------------------------------------
BrajapUSDA122SDgb2         --------------------------------------------------
BrajapUSDA4SDgb2           --------------------------------------------------
BrajapWSM2793SDgb2         --------------------------------------------------
BrajapUSDA6-7488SDgb2      --------------------------------------------------
BrajapUSDA6-8372SDgb1      --------------------------------------------------
BrajapUSDA38SDgb2          --------------------------------------------------
BrajapUSDA123SDgb2         --------------------------------------------------
BrajapUSDA124SDgb2         --------------------------------------------------
Brajapin8p8GCS             EQQGVATAEIARNVQEAAQGTHDVSSNIGGVSQAARETGITASELLAASK
Brajapis5GCS               EQQGVATAEIARNVQEAAQGTHDVSSNIGGVSQAARETGITASELLAASK
BraelkWSM2783GCS           EQQDAATSEISRNIQQAAVGTRDVSENIEDVMHAAKESGGSASNVLDAAG
RhilegWSM1481GCS2          SGIATRTGEHAASLDSVTSDVKGLGSEVAGSADLAERSAEGADHLHTVIL
Rhileg248GCS2              SGIATRTGEHAASLDSVTSDVKGLGGEVADSAGLAERSAEGADHLHTVIL
RhilegTOMGCS2              SGIATRTGEHAASLDSVAADVKGLGGEVADSAGLAERSAEGADHLHSVIL
RhilegUPM1131GCS           SGIATRTGEHAASLDSVTADVRGLGSEVADSAGLAERSADGADHLHTVIL
RhilegVF39GCS2             AGIATRTGEHAASLDSVTSDVKGLGGEVAASAGLAERSAEGADHLHTVIL
RhietlCIAT894GCS           --------------------------------------------------
RhilegVh3GCS2              AGIATRTGEHAASLDSVTSDVKGLGGEVADSAGLAERSAEGADHLHTVIL
Rhileg128C53GCS2           AGIATRTGEHAASLDSVTSDVKGLGGEVADSAGLAERSAEGADHLHTVIL
RhilegPs8GCS2              AGIATRTGEHAASLDSVTSDVKGLGGEVADSAGLAERSAEGADHLHTVIL
RhilegGB30GCS2             AGIATRTGEHAASLDSVTSDVKGLGGEVADSAGLAERSAEGADHLHTVIL
RhilegUPM1137GCS2          AGIATRTGEHAASLDSVTSDVKGLGGEVADSAGLAERSAEGADHLHTVIL
RhilegWSM1455GCS2          SGIATRTGEHAASLDSVTSDVKGLGHEVADSAGLAERSAEGADHLQTVIL
```

```
Rhileg3841GCS2          SGIATRTGEHAASLDSVTSDVKGLGSEVADSAGLAERSAEGADHLHTVIL
RhilegVc2GCS2           AGIATRTGEHAASLDSVTSDVKGLGGEVADSAGLAERSAEGADHLHTVIL
Rhietl8C3GCS            --------------------------------------------------
RhietlCIAT652GCS2       SGIATRTGEHAASLDGVTAEVKGLGAEVADSAGFAGRSADGADDLHTVIV
RhietlCFN42GCS2         SGIATRTGEHAACLDGVAADVKELGAEVADNAGLAERSAEDADHLHTVIL
SinfreGR64GCS           SGIARDAETQVNDLAAATSEIGGISKAMTRSATLAAEAAASTEDLRGTIL
SinfreHH103GCS          SGIARDAETQVNDLAAATSEIGGISKAMTRSATLAGEAAASTEDLRGTIL
SinfreUSDA257GCS        SGIAREAESQVGDLAAAASDIGGISKAMSRSAALAGEAAASTEDLHGTIL
Sinmel1021GCS           TGIAREAECQVNDLASATSEIGGVAQAMNRSAALAGAALASTDDLHGVIV
RhietlCIAT652GCS1       ADIAAAARDQATGVNEVSVAIRSMGEITQQNSGMVEHTSAETRRLKDEVE
RhietlCNPAF512GCS       ADIAAAARDQATGVNEVSVAIRSMGEITQQNSGMVEHTSAETRRLTDEVE
RhietlGR56GCS           ADIAAAARDQATGRQRRSAWRSAA--------------------------
Rhileg128C53GCS1        ADIAAAARDQATGVNEVSVAVRNMGAITQQNSDMVEQSSAETRRLKDEVE
RhilegUPM1137GCS1       ADIAAAARDQATGVNEVSVAVRNMGAITQQNSDMVEQSSAETRRLKDEVE
RhilegGB30GCS1          ADIAAAARDQATGVNEVSLAVRNMGAITQQNSDMVEHSSAETRRLKDEVE
RhilegPs8GCS1           ADIAAAARDQATGVNEVSLAVRNMGAITQQNSDMVEHSSAETRRLKDEVE
RhilegVc2GCS1           ADIAAAARDQATGVNEVSVAVRNMGAITQQNSDMVEHSSAETRRLKDEVE
RhilegVh3GCS1           ADIAAAARDQATGVNEVSVAVRNMGAITQQNSDMVEHSSAETRRLKDEVE
RhilegVF39GCS1          ADIAAAARDQATGVNEVSVAVRNMGAITQQNSDMVEHSSAETRRLKDEVE
Rhileg3841GCS1          ADIAAAARDQATGVNEVSVAVRNMGAITQQNSDMVEHSSAETRRLKDEVE
RhilegWSM1455GCS1       ADIASAARDQATGVNEVSLAVRNMGAITQQNSDMVEHSSAETRRLKDEVE
RhilegWSM1481GCS1       ADIASAARDQATGVNEVSLAVRNMGAITQQNSDMVEHSSAETRRLKDEVE
Rhileg248GCS1           ADIAAAARDQATGVNEVSIAVRNMGAITQQNSDMVEHSSAETRRLKDEVE
RhilegTOMGCS1           ADIAAAARDQATGVNEVSLAVRNMGAITQQNSDMVEQSSAETRRLKDEVE
RhietlCFN42GCS1         ADIAAAARDQATGVNEVSVAIRSMGEITQQNSGMVERTSAETGRLKEEVD
AgrtumGCS               TSLTKAAQEQSVGIQEISAAINSMDNLTQKNAAMVEETNAATHNLSDVSA
MyctubHbtC1             --------------------------------------------------
CupnecN1Hbt1            --------------------------------------------------
BrajapUSDA4Hbt2         --------------------------------------------------
BrajapWSM2793Hbt3       --------------------------------------------------
MyctubHbtC2             --------------------------------------------------
BurphySTM815Hbt1        --------------------------------------------------
MycaviHbtC3             --------------------------------------------------
BraelkWSM1741Hbt1       --------------------------------------------------
BacsubGCS               DEIVHSMKISKEQSGKIDVDLQAFLGGLQEVSRAVSHVAASVDSLVILTE
```

```
                                  710       720       730       740       750
                                    |         |         |         |         |
BraelkUSDA3254Hbt       --------------------------------------------------
BraelkUSDA3259Hbt       --------------------------------------------------
BraelkUSDA76Hbt2        --------------------------------------------------
Braelk587Hbt1           --------------------------------------------------
BraelkUSDA94Hbt2        --------------------------------------------------
BraelkWSM2783Hbt        --------------------------------------------------
BraelkWSM1741Hbt2       --------------------------------------------------
BrajapUSDA4Hbt1         --------------------------------------------------
BrajapWSM2793Hbt1       --------------------------------------------------
BrajapUSDA123Hbt1       --------------------------------------------------
BrajapUSDA6-8372Hbt1    --------------------------------------------------
BrajapUSDA6-7488Hbt1    --------------------------------------------------
BrajapUSDA38Hbt1        --------------------------------------------------
Brajapin8p8Hbt1         --------------------------------------------------
Brajapis5Hbt1           --------------------------------------------------
BrajapUSDA135Hbt1       --------------------------------------------------
BrajapUSDA122Hbt1       --------------------------------------------------
BrajapUSDA124Hbt1       --------------------------------------------------
BrajapWSM1743Hbt1       --------------------------------------------------
Brajap22Hbt1            --------------------------------------------------
AzodoeUFLA1-100Hbt1     --------------------------------------------------
MeslotNZP2037Hbt2       --------------------------------------------------
MeslotUSDA3471Hbt1      --------------------------------------------------
RhietlCIAT652Hbt2       --------------------------------------------------
RhietlCNPAF512Hbt       --------------------------------------------------
RhietlBrasil5Hbt        --------------------------------------------------
RhietlCFN42Hbt2         --------------------------------------------------
Rhileg3841Hbt2          --------------------------------------------------
RhilegWSM1455Hbt2       --------------------------------------------------
RhilegVF39Hbt1          --------------------------------------------------
```

```
RhilegWSM1481Hbt2          --------------------------------------------------
RhilegUPM1131Hbt2          --------------------------------------------------
Rhileg248Hbt2              --------------------------------------------------
RhilegTOMHbt2              --------------------------------------------------
Rhileg128C53Hbt1           --------------------------------------------------
RhilegPs8Hbt2              --------------------------------------------------
RhilegGB30Hbt2             --------------------------------------------------
RhilegUPM1137Hbt2          --------------------------------------------------
RhilegVc2Hbt2              --------------------------------------------------
RhilegVh3Hbt1              --------------------------------------------------
Sinmel1021Hbt2             --------------------------------------------------
RhilupHPC_L_Hbt1           --------------------------------------------------
RhilupHPC_L_Hbt2           --------------------------------------------------
AgrtumHbtC2                --------------------------------------------------
SinfreUSDA257Hbt           --------------------------------------------------
SinfreHH103Hbt             --------------------------------------------------
SinfreGR64Hbt              --------------------------------------------------
Sinmel1021Hbt1             --------------------------------------------------
RhilegUPM1137Hbt1          --------------------------------------------------
Rhileg128C53Hbt2           --------------------------------------------------
RhilegVc2Hbt1              --------------------------------------------------
RhilegVh3Hbt2              --------------------------------------------------
RhilegGB30Hbt1             --------------------------------------------------
RhilegPs8Hbt1              --------------------------------------------------
Rhileg3841Hbt1             --------------------------------------------------
Rhileg248Hbt1              --------------------------------------------------
RhilegWSM1455Hbt1          --------------------------------------------------
RhilegWSM1481Hbt1          --------------------------------------------------
RhilegVF39Hbt2             --------------------------------------------------
RhilegTOMHbt1              --------------------------------------------------
RhilegUPM1131Hbt1          --------------------------------------------------
RhietlKIM5Hbt              --------------------------------------------------
RhietlCFN42Hbt1            --------------------------------------------------
Rhietl8C3Hbt               --------------------------------------------------
RhietlCIAT652Hbt1          --------------------------------------------------
RhietlCIAT894Hbt           --------------------------------------------------
RhietlIE4771Hbt            --------------------------------------------------
AzodoeUFLA1-100Hbt2        --------------------------------------------------
Brajap22Hbt2               --------------------------------------------------
BrajapUSDA110Hbt           --------------------------------------------------
BrajapUSDA122Hbt2          --------------------------------------------------
BrajapUSDA124Hbt2          --------------------------------------------------
BrajapWSM1743Hbt2          --------------------------------------------------
BrajapUSDA6-8372Hbt2       --------------------------------------------------
BrajapUSDA6-7488Hbt2       --------------------------------------------------
BrajapUSDA38Hbt2           --------------------------------------------------
BrajapUSDA123Hbt2          --------------------------------------------------
BrajapWSM2793Hbt2          --------------------------------------------------
BrajapUSDA135Hbt2          --------------------------------------------------
BrajapUSDA4Hbt3            --------------------------------------------------
Brajap22Hbt3               --------------------------------------------------
Brajapin8p8Hbt2            --------------------------------------------------
Brajapis5Hbt2              --------------------------------------------------
CupnecHPC_L_Hbt            --------------------------------------------------
CupnecJMP134Hbt            --------------------------------------------------
CupnecN1Hbt2               --------------------------------------------------
BurphySTM815Hbt2           --------------------------------------------------
BraelkUSDA76Hbt1           --------------------------------------------------
Braelk587Hbt2              --------------------------------------------------
BraelkUSDA94Hbt1           --------------------------------------------------
MescicCMG6Hbt              --------------------------------------------------
MescicWSM1271Hbt           --------------------------------------------------
MescicWSM4083Hbt           --------------------------------------------------
MeslotR7AHbt               --------------------------------------------------
MeslotMAFF303099Hbt        --------------------------------------------------
MeslotNZP2037Hbt1          --------------------------------------------------
MeslotR88bHbt              --------------------------------------------------
MeslotCJ3symHbt            --------------------------------------------------
```

```
MeslotUSDA3471Hbt2          --------------------------------------------------
BurphySTM815fHb             --------------------------------------------------
CupnecHPC_L_fHb             --------------------------------------------------
CupnecN1fHb2                --------------------------------------------------
Sinmel1021fHb               --------------------------------------------------
BacsubfHb                   --------------------------------------------------
VitSDgb                     --------------------------------------------------
EsccolfHb                   --------------------------------------------------
RhilegUPM1137fHb            --------------------------------------------------
CupnecN1fHb1                --------------------------------------------------
CupnecJMP134fHb             --------------------------------------------------
SaccerfHb                   --------------------------------------------------
BrajapUSDA110SDgb1          --------------------------------------------------
BrajapUSDA122SDgb1          --------------------------------------------------
Brajapin8p8SDgb             --------------------------------------------------
Brajapis5SDgb               --------------------------------------------------
BrajapUSDA135SDgb1          --------------------------------------------------
BrajapWSM1743SDgb           --------------------------------------------------
BrajapUSDA124SDgb1          --------------------------------------------------
Brajap22SDgb                --------------------------------------------------
BrajapUSDA6-7488SDgb1       --------------------------------------------------
BrajapUSDA6-8372SDgb2       --------------------------------------------------
BrajapUSDA123SDgb1          --------------------------------------------------
BrajapUSDA38SDgb1           --------------------------------------------------
BrajapUSDA4SDgb1            --------------------------------------------------
BrajapWSM2793SDgb1          --------------------------------------------------
BraelkUSDA3254SDgb4         --------------------------------------------------
BraelkUSDA3259SDgb1         --------------------------------------------------
BraelkUSDA94SDgb1           --------------------------------------------------
Braelk587SDgb1              --------------------------------------------------
BraelkUSDA76SDgb1           --------------------------------------------------
BraelkWSM2783SDgb1          --------------------------------------------------
AzodoeUFLA1-100SDgb         --------------------------------------------------
BraelkWSM1741SDgb1          --------------------------------------------------
BraelkWSM2783SDgb2          --------------------------------------------------
BraelkUSDA3254SDgb2         --------------------------------------------------
BraelkUSDA3259SDgb2         --------------------------------------------------
BraelkUSDA3254SDgb3         --------------------------------------------------
BraelkUSDA3259SDgb4         --------------------------------------------------
Braelk587SDgb2              --------------------------------------------------
BraelkUSDA76SDgb2           --------------------------------------------------
BraelkUSDA94SDgb2           --------------------------------------------------
BrajapUSDA135SDgb2          --------------------------------------------------
BraelkUSDA3254SDgb1         --------------------------------------------------
BraelkUSDA3259SDgb3         --------------------------------------------------
BraelkUSDA94SDgb3           --------------------------------------------------
BraelkUSDA76SDgb3           --------------------------------------------------
Braelk587SDgb3              --------------------------------------------------
BraelkWSM2783SDgb3          --------------------------------------------------
BraelkWSM1741SDgb2          --------------------------------------------------
BrajapUSDA110SDgb2          --------------------------------------------------
BrajapUSDA122SDgb2          --------------------------------------------------
BrajapUSDA4SDgb2            --------------------------------------------------
BrajapWSM2793SDgb2          --------------------------------------------------
BrajapUSDA6-7488SDgb2       --------------------------------------------------
BrajapUSDA6-8372SDgb1       --------------------------------------------------
BrajapUSDA38SDgb2           --------------------------------------------------
BrajapUSDA123SDgb2          --------------------------------------------------
BrajapUSDA124SDgb2          --------------------------------------------------
Brajapin8p8GCS              ELSGQTDVLRSEVEGFFAAIRAA---------------------------
Brajapis5GCS                ELSGQTDVLRSEVEGFFAAIRAA---------------------------
BraelkWSM2783GCS            ELSRQSELLRNEVSKFLATVKAA---------------------------
RhilegWSM1481GCS2           ELGQTIREFRIARENATAGRPAPVRVTPPRALEGASRPAPVVDEYENDDF
Rhileg248GCS2               ELGQTIREFRIARENATAGRPAPVRVTSPRAVEGATRPAPVVDEYENDDF
RhilegTOMGCS2               ELGQTIREFRIARENAIAGRPAPVRMTPPRALEGATRPAPVVDEYENDDF
RhilegUPM1131GCS            ELGQTIREFRIARENATAGRPATVRVTAPRALEGATRPAPVVDEYENDDF
RhilegVF39GCS2              ELGQTIREFRIARENATAGRPAQVRMTPPRALEGTTRPAPVVDEYENDDF
RhietlCIAT894GCS            --------------------------------------------------
```

```
RhilegVh3GCS2              ELGQTIREFRIARENATAGRPAPVRVTPPRAIEVAARPALTEDGYENDDF
Rhileg128C53GCS2           ELGQTIREFRIARENATAGRQAPVRVTPPRAIEAAARPAPAVDEYENDDF
RhilegPs8GCS2              ELGQTIREFRIARENATAGRPAPVRVTPPRAIEVAARPALTEDGYENDDF
RhilegGB30GCS2             ELGQTIREFRIARENATAGRPAPVRVTPPRAIEVAARPALTEDGYENDDF
RhilegUPM1137GCS2          ELGQTIREFRIARENATAGRPAPVRVTPPRAIEAAARPAPAVDEYENDDF
RhilegWSM1455GCS2          ELGQTIREFRIARENATAGRPAPVRVTSPRALEDATRPAPVVDEYESDDF
Rhileg3841GCS2             ELGQTIREFRIARENATAGRPAPVRATPPRALEGTTRPAPVADEYENDDF
RhilegVc2GCS2              ELGQTIREFRIARENATAGRPAPVRVTPPRAIEVAGRPALAEDGYENDDF
Rhietl8C3GCS               --------------------------------------------------
RhietlCIAT652GCS2          ELGRTVREFRVARENAHAGRSAPVRVAPSRPIDAAARAAAVEGEYENDDF
RhietlCFN42GCS2            ELGQTIREFRIARENAHSGRLGSVRATPSRGIETAGR-PAVEDEYENEDF
SinfreGR64GCS              ELGQTIRRFRFERQAGLRASTFSPPPALLPQVED-EAAMPFSDDAALGRH
SinfreHH103GCS             ELGQTIRRFRFERQAGLRASTFSPPPALLPQVED-EAAMPFSDDAALGRH
SinfreUSDA257GCS           ELGQTIRRFRFERQAGLRASTFALPPALPPQIDEDEAAMPFSDDAASGRQ
Sinmel1021GCS              ELGETIRRFHLDRQARSAASFAPRMRIEAPEDETTSPFG----EVTSERH
RhietlCIAT652GCS1          KLIELLRRFRARPEGRSVAATRRAA-------------------------
RhietlCNPAF512GCS          KLIELLRRFRARPEGRSVAATRRAA-------------------------
RhietlGR56GCS              --------------------------------------------------
Rhileg128C53GCS1           TLIELLRRFRARPEGRSTGAARRAA-------------------------
RhilegUPM1137GCS1          TLIELLRRFRARPEGRSTGAARRAA-------------------------
RhilegGB30GCS1             SLIELLRRFRARPEGHSASAARRAA-------------------------
RhilegPs8GCS1              SLIELLRRFRARPEGHSASAARRAA-------------------------
RhilegVc2GCS1              SLIELLRRFRARPEGHSIVARRAA--------------------------
RhilegVh3GCS1              SLIELLRRFRARPEGHSIVARRAA--------------------------
RhilegVF39GCS1             SLIELLRRFRARPEGHSASAARRAA-------------------------
Rhileg3841GCS1             SLIELLRRFRARPEGHSASAARRAA-------------------------
RhilegWSM1455GCS1          TLIELLRRFRARPEGSSASAARRAA-------------------------
RhilegWSM1481GCS1          TLIELLRRFRARPEGSSAGAARRAA-------------------------
Rhileg248GCS1              TLIELLRRFRARPEGRSAGAARQAA-------------------------
RhilegTOMGCS1              TLIELLRRFRARPEGRSAGAARRAA-------------------------
RhietlCFN42GCS1            HLIELLRRFRARPEAHAAITARRAA-------------------------
AgrtumGCS                  NLAALVSRFSVSATRAHVERTYRAA-------------------------
MyctubHbtC1                --------------------------------------------------
CupnecN1Hbt1               --------------------------------------------------
BrajapUSDA4Hbt2            --------------------------------------------------
BrajapWSM2793Hbt3          --------------------------------------------------
MyctubHbtC2                --------------------------------------------------
BurphySTM815Hbt1           --------------------------------------------------
MycaviHbtC3                --------------------------------------------------
BraelkWSM1741Hbt1          --------------------------------------------------
BacsubGCS                  E-------------------------------------------------

                                   760    766
                                    |      |
BraelkUSDA3254Hbt          ----------------
BraelkUSDA3259Hbt          ----------------
BraelkUSDA76Hbt2           ----------------
Braelk587Hbt1              ----------------
BraelkUSDA94Hbt2           ----------------
BraelkWSM2783Hbt           ----------------
BraelkWSM1741Hbt2          ----------------
BrajapUSDA4Hbt1            ----------------
BrajapWSM2793Hbt1          ----------------
BrajapUSDA123Hbt1          ----------------
BrajapUSDA6-8372Hbt1       ----------------
BrajapUSDA6-7488Hbt1       ----------------
BrajapUSDA38Hbt1           ----------------
Brajapin8p8Hbt1            ----------------
Brajapis5Hbt1              ----------------
BrajapUSDA135Hbt1          ----------------
BrajapUSDA122Hbt1          ----------------
BrajapUSDA124Hbt1          ----------------
BrajapWSM1743Hbt1          ----------------
Brajap22Hbt1               ----------------
AzodoeUFLA1-100Hbt1        ----------------
MeslotNZP2037Hbt2          ----------------
MeslotUSDA3471Hbt1         ----------------
RhietlCIAT652Hbt2          ----------------
```

```
RhietlCNPAF512Hbt          ----------------
RhietlBrasil5Hbt           ----------------
RhietlCFN42Hbt2            ----------------
Rhileg3841Hbt2             ----------------
RhilegWSM1455Hbt2          ----------------
RhilegVF39Hbt1             ----------------
RhilegWSM1481Hbt2          ----------------
RhilegUPM1131Hbt2          ----------------
Rhileg248Hbt2              ----------------
RhilegTOMHbt2              ----------------
Rhileg128C53Hbt1           ----------------
RhilegPs8Hbt2              ----------------
RhilegGB30Hbt2             ----------------
RhilegUPM1137Hbt2          ----------------
RhilegVc2Hbt2              ----------------
RhilegVh3Hbt1              ----------------
Sinmel1021Hbt2             ----------------
RhilupHPC_L_Hbt1           ----------------
RhilupHPC_L_Hbt2           ----------------
AgrtumHbtC2                ----------------
SinfreUSDA257Hbt           ----------------
SinfreHH103Hbt             ----------------
SinfreGR64Hbt              ----------------
Sinmel1021Hbt1             ----------------
RhilegUPM1137Hbt1          ----------------
Rhileg128C53Hbt2           ----------------
RhilegVc2Hbt1              ----------------
RhilegVh3Hbt2              ----------------
RhilegGB30Hbt1             ----------------
RhilegPs8Hbt1              ----------------
Rhileg3841Hbt1             ----------------
Rhileg248Hbt1              ----------------
RhilegWSM1455Hbt1          ----------------
RhilegWSM1481Hbt1          ----------------
RhilegVF39Hbt2             ----------------
RhilegTOMHbt1              ----------------
RhilegUPM1131Hbt1          ----------------
RhietlKIM5Hbt              ----------------
RhietlCFN42Hbt1            ----------------
Rhietl8C3Hbt               ----------------
RhietlCIAT652Hbt1          ----------------
RhietlCIAT894Hbt           ----------------
RhietlIE4771Hbt            ----------------
AzodoeUFLA1-100Hbt2        ----------------
Brajap22Hbt2               ----------------
BrajapUSDA110Hbt           ----------------
BrajapUSDA122Hbt2          ----------------
BrajapUSDA124Hbt2          ----------------
BrajapWSM1743Hbt2          ----------------
BrajapUSDA6-8372Hbt2       ----------------
BrajapUSDA6-7488Hbt2       ----------------
BrajapUSDA38Hbt2           ----------------
BrajapUSDA123Hbt2          ----------------
BrajapWSM2793Hbt2          ----------------
BrajapUSDA135Hbt2          ----------------
BrajapUSDA4Hbt3            ----------------
Brajap22Hbt3               ----------------
Brajapin8p8Hbt2            ----------------
Brajapis5Hbt2              ----------------
CupnecHPC_L_Hbt            ----------------
CupnecJMP134Hbt            ----------------
CupnecN1Hbt2               ----------------
BurphySTM815Hbt2           ----------------
BraelkUSDA76Hbt1           ----------------
Braelk587Hbt2              ----------------
BraelkUSDA94Hbt1           ----------------
MescicCMG6Hbt              ----------------
MescicWSM1271Hbt           ----------------
```

MescicWSM4083Hbt	----------------
MeslotR7AHbt	----------------
MeslotMAFF303099Hbt	----------------
MeslotNZP2037Hbt1	----------------
MeslotR88bHbt	----------------
MeslotCJ3symHbt	----------------
MeslotUSDA3471Hbt2	----------------
BurphySTM815fHb	----------------
CupnecHPC_L_fHb	----------------
CupnecN1fHb2	----------------
Sinmel1021fHb	----------------
BacsubfHb	----------------
VitSDgb	----------------
EsccolfHb	----------------
RhilegUPM1137fHb	----------------
CupnecN1fHb1	----------------
CupnecJMP134fHb	----------------
SaccerfHb	----------------
BrajapUSDA110SDgb1	----------------
BrajapUSDA122SDgb1	----------------
Brajapin8p8SDgb	----------------
Brajapis5SDgb	----------------
BrajapUSDA135SDgb1	----------------
BrajapWSM1743SDgb	----------------
BrajapUSDA124SDgb1	----------------
Brajap22SDgb	----------------
BrajapUSDA6-7488SDgb1	----------------
BrajapUSDA6-8372SDgb2	----------------
BrajapUSDA123SDgb1	----------------
BrajapUSDA38SDgb1	----------------
BrajapUSDA4SDgb1	----------------
BrajapWSM2793SDgb1	----------------
BraelkUSDA3254SDgb4	----------------
BraelkUSDA3259SDgb1	----------------
BraelkUSDA94SDgb1	----------------
Braelk587SDgb1	----------------
BraelkUSDA76SDgb1	----------------
BraelkWSM2783SDgb1	----------------
AzodoeUFLA1-100SDgb	----------------
BraelkWSM1741SDgb1	----------------
BraelkWSM2783SDgb2	----------------
BraelkUSDA3254SDgb2	----------------
BraelkUSDA3259SDgb2	----------------
BraelkUSDA3254SDgb3	----------------
BraelkUSDA3259SDgb4	----------------
Braelk587SDgb2	----------------
BraelkUSDA76SDgb2	----------------
BraelkUSDA94SDgb2	----------------
BrajapUSDA135SDgb2	----------------
BraelkUSDA3254SDgb1	----------------
BraelkUSDA3259SDgb3	----------------
BraelkUSDA94SDgb3	----------------
BraelkUSDA76SDgb3	----------------
Braelk587SDgb3	----------------
BraelkWSM2783SDgb3	----------------
BraelkWSM1741SDgb2	----------------
BrajapUSDA110SDgb2	----------------
BrajapUSDA122SDgb2	----------------
BrajapUSDA4SDgb2	----------------
BrajapWSM2793SDgb2	----------------
BrajapUSDA6-7488SDgb2	----------------
BrajapUSDA6-8372SDgb1	----------------
BrajapUSDA38SDgb2	----------------
BrajapUSDA123SDgb2	----------------
BrajapUSDA124SDgb2	----------------
Brajapin8p8GCS	----------------
Brajapis5GCS	----------------
BraelkWSM2783GCS	----------------

RhilegWSM1481GCS2	GLPQALASVGGGRNVY
Rhileg248GCS2	GLPQAFASVGGGRNVY
RhilegTOMGCS2	GLPQAFASVGGGRNVY
RhilegUPM1131GCS	GLPQAFANVGGGRNVY
RhilegVF39GCS2	GLPQALASVGGGRNVY
RhietlCIAT894GCS	----------------
RhilegVh3GCS2	GLPQPFASVGGGRNVY
Rhileg128C53GCS2	GLPQPFAGVGGGRNVY
RhilegPs8GCS2	GLPQPFASVGGGRNVY
RhilegGB30GCS2	GLPQPFASVGGGRNVY
RhilegUPM1137GCS2	GLPQPFAGVGGGRNVY
RhilegWSM1455GCS2	GLPQVFASVGGGRNVY
Rhileg3841GCS2	GLPQAFASVGGGRNVY
RhilegVc2GCS2	GLPQPFASVGGGRNVY
Rhietl8C3GCS	----------------
RhietlCIAT652GCS2	GLPQAFASVGGGRNVY
RhietlCFN42GCS2	GLPQSLASVGGGRNVY
SinfreGR64GCS	VAGWRR----------
SinfreHH103GCS	VAGWRR----------
SinfreUSDA257GCS	VAAWRR----------
Sinmel1021GCS	LAGWRR----------
RhietlCIAT652GCS1	----------------
RhietlCNPAF512GCS	----------------
RhietlGR56GCS	----------------
Rhileg128C53GCS1	----------------
RhilegUPM1137GCS1	----------------
RhilegGB30GCS1	----------------
RhilegPs8GCS1	----------------
RhilegVc2GCS1	----------------
RhilegVh3GCS1	----------------
RhilegVF39GCS1	----------------
Rhileg3841GCS1	----------------
RhilegWSM1455GCS1	----------------
RhilegWSM1481GCS1	----------------
Rhileg248GCS1	----------------
RhilegTOMGCS1	----------------
RhietlCFN42GCS1	----------------
AgrtumGCS	----------------
MyctubHbtC1	----------------
CupnecN1Hbt1	----------------
BrajapUSDA4Hbt2	----------------
BrajapWSM2793Hbt3	----------------
MyctubHbtC2	----------------
BurphySTM815Hbt1	----------------
MycaviHbtC3	----------------
BraelkWSM1741Hbt1	----------------
BacsubGCS	----------------
