# Supplementary material for: A bioinformatics insight to rhizobial globins: gene identification and mapping, polypeptide sequence and phenetic analysis, and protein modeling
Source: F1000Res. 2015 May 13;4:117. [Version 1] doi: 10.12688/f1000research.6392.1 (PMC4648194; doi:10.12688/f1000research.6392.1)
Supplement: Peer review history

# Peer review: Referee response for version 1

**DOI:** 10.5256/f1000research.6858.r9023

**Published:** 2015-07-08

**Authors:** Paul Twigg

**Affiliations:** 1 Department of Biology, University of Nebraska at Kearney, Kearney, NE, USA

I find this paper by Gesto-Borroto *et al. *to be well written and analyzed.  This paper fills a gap in the knowledge base for what is known about bacterial or more specifically rhizobial globins.  The authors seem to have taken great care to analyze all available globins from various rhizobial species and biovars.  I have no major revisions for this work.  It is comprehensive and demonstrates some interesting points about globins in rhizobia.  The coexistence of various globins in the bacteria begs questions about their control and functions that undoubtedly other researchers will address.  The work with the promoter analysis was particularly interesting to me indicating more than once that the globin expression is likely tied to nitrate/nitrogen metabolism.  The absence of the -10 promoter area was also unexpected.  The amino acid sequence alignment data also shows interesting information about the conserved positions in the sequence.  The phenetic relationships are also interesting and appropriately analyzed.  The structural modeling is also well done and reveals interesting points about the structure and function of the various globins.  Lastly, the authors back up some of their proposals with spectroscopic data from globing extracts.  Again, overall I thought that the work was well done and needs no major revisions.

I have read this submission. I believe that I have an appropriate level of expertise to confirm that it is of an acceptable scientific standard.

# Response

**Published:** 2015-07-08

**Authors:** Raul Arredondo-Peter

**Affiliations:** Universidad Autonoma del Estado de Morelos, Mexico

We thank Dr. Twigg for evaluating this article and constructive comments.

# Peer review: Referee response for version 1

**DOI:** 10.5256/f1000research.6858.r8649

**Published:** 2015-05-26

**Authors:** Manuel Becana

**Affiliations:** 1 Department of Plant Nutrition, Spanish National Research Council, Zaragoza, Spain

This is a well-written paper on a subject of great interest. There is very little information of rhizobial globins and the authors have done a good job by systematically analyzing the composition of globin genes of 62 genomes in various genera, species and biovars of rhizobia. The authors are experts in the phylogeny and evolution of plant hemoglobins, and I have no major comments to improve this work. It will nevertheless be of interest for future work to address the issue of why several types of hemoglobins coexist in rhizobia. For example, both truncated hemoglobins and flavohemoglobins seem to be present within the same species and strain, although this would have to be verified by identifying the proteins themselves rather than by only gene sequencing or by analyzing differential spectra (reduced + CO vs reduced) in bacterial extracts. Both classes of hemoglobins have been proposed to act as modulators of NO concentration, but they are unlikely to have redundant functions. An interesting, additional aspect of the work is the mapping analysis, including the report of flanking sequences. This hints to a role of at least some rhizobial globins in nitrogen metabolism. This observation is very timing because of the recent discovery that truncated hemoglobins of Chlamydomonas regulate nitrate reductase.

I have read this submission. I believe that I have an appropriate level of expertise to confirm that it is of an acceptable scientific standard.

# Response

**Published:** 2015-05-26

**Authors:** Raul Arredondo-Peter

**Affiliations:** Universidad Autonoma del Estado de Morelos, Mexico

We thank Dr. Becana for evaluating this article and constructive comments.
